# Overview of Ethnobotanical–Pharmacological Studies Carried Out on Medicinal Plants from the Serra da Estrela Natural Park: Focus on Their Antidiabetic Potential

**DOI:** 10.3390/pharmaceutics16040454

**Published:** 2024-03-25

**Authors:** Radhia Aitfella Lahlou, Filomena Carvalho, Maria João Pereira, João Lopes, Luís R. Silva

**Affiliations:** 1 SPRINT Sport Physical Activity and Health Research & Innovation Center, Instituto Politécnico da Guarda, 6300-559 Guarda, Portugal; radhialahlou@ipg.pt (R.A.L.); filomenacarvalho@ipg.pt (F.C.); 2 CERENA/DER, Instituto Superior Técnico, Universidade de Lisboa, Av. Rovisco Pais, 1049-001 Lisbon, Portugal; maria.pereira@tecnico.ulisboa.pt; 3 iMed.ULisboa, Research Institute for Medicines, Faculdade de Farmácia, University of Lisboa, 1649-003 Lisboa, Portugal; jlopes@ff.ulisboa.pt; 4 CICS-UBI—Health Sciences Research Center, University of Beira Interior, 6201-506 Covilhã, Portugal; 5 CERES, Department of Chemical Engineering, University of Coimbra, 3030-790 Coimbra, Portugal

**Keywords:** Natural Park of Serra da Estrela, botanical diversity, medicinal plants, diabetes prevention, pharmacological applications, chemical composition

## Abstract

The Serra da Estrela Natural Park (NPSE) in Portugal stands out as a well-preserved region abundant in medicinal plants, particularly known for their pharmaceutical applications in diabetes prevention and treatment. This comprehensive review explores these plants’ botanical diversity, traditional uses, pharmacological applications, and chemical composition. The NPSE boast a rich diversity with 138 medicinal plants across 55 families identified as traditionally and pharmacologically used against diabetes globally. Notably, the *Asteraceae* and *Lamiaceae* families are prevalent in antidiabetic applications. In vitro studies have revealed their significant inhibition of carbohydrate-metabolizing enzymes, and certain plant co-products regulate genes involved in carbohydrate metabolism and insulin secretion. In vivo trials have demonstrated antidiabetic effects, including glycaemia regulation, insulin secretion, antioxidant activity, and lipid profile modulation. Medicinal plants in NPSE exhibit various activities beyond antidiabetic, such as antioxidant, anti-inflammatory, antibacterial, anti-cancer, and more. Chemical analyses have identified over fifty compounds like phenolic acids, flavonoids, terpenoids, and polysaccharides responsible for their efficacy against diabetes. These findings underscore the potential of NPSE medicinal plants as antidiabetic candidates, urging further research to develop effective plant-based antidiabetic drugs, beverages, and supplements.

## 1. Introduction

Type 2 diabetes mellitus (T2DM) is a chronic metabolic disease associated with multiple dysfunctions in the endocrine system [1], affecting the metabolism of carbohydrates, lipids, and proteins [2]. This disorder is related to a defect in insulin secretion and/or a progressive alteration in its function in the body [3,4]. The onset of hyperglycemia and insulin resistance leads to the accumulation of free fatty acids, lipid peroxidation, the disruption of cellular antioxidant defences, and inflammatory reactions [5,6,7,8]. Although some of the underlying mechanisms are uncertain, all these factors contribute to the disturbance of the integrity of physiological barriers [9], mainly by altering the vascular integrity of tissues, and may contribute to the clinically recognised complications of diabetes (hypertension, diabetic peripheral neuropathy, chronic kidney disease, retinopathy, cardiovascular disease, and others) [10,11,12,13,14,15]. T2DM is often associated with fatty liver, sleep apnea syndrome, depression, cognitive decline, and dementia [16].

According to current diabetes statistics, more than 90% of cases worldwide are T2DM; the older generation is the most affected of the 500 million people suffering from the disease [17]. About 422 million people worldwide have T2DM, most living in low–middle-income countries, and 1.5 million deaths are directly attributed to diabetes yearly [18]. In Portugal, the incidence of T2DM is much higher than in other types of diabetes [19,20]. Epidemiological studies show that Portugal (9.1%) is among the countries with the highest rates in Europe, alongside Turkey (14.5%), Spain (10.3%), Andorra (9.7%), and Serbia (9.1%) [17]. It is estimated that T2DM affects 13.6% of the Portuguese population aged between 20 and 79; an equivalent rate of 5.9% of people are unaware that they have the disease [21]. The data for 2021 already show an increase in the number of new cases identified [19]. Around 857,272 people with T2DM have been registered with the Portuguese National Health Service, including 74,396 new diagnoses [19]. Approximately 200 new patients are diagnosed with diabetes daily in Portugal [19]. 

T2DM can occur for a variety of reasons. Hyperglycaemia, obesity, hypertriglyceridaemia, poor eating habits, lack of exercise, ageing, family history, alcohol consumption, smoking, stress, anxiety, and depression are the main risk factors for the onset of the disease in adults [22,23,24,25,26]. Statistics show it is present in young people due to poor diet and lifestyle [17,27,28]. Multiple studies have shown that people from different ethnic backgrounds may have specific phenotypes that increase susceptibility to hypertension, insulin resistance, and dyslipidaemia [29]. People of Latin American, East, Southeast and South Asian, sub-Saharan African, Middle Eastern, and North African origin living in Europe are 1.3 to 3.7 times more likely to have T2DM than white European populations [30]. In the United States, 14.7% of American Indians and Alaska Natives have been diagnosed with diabetes, compared with 7.5% of non-Hispanic white Americans [31]. Regarding the gender factor, both men and women are affected, but worldwide, an estimated 17.7 million more men than women have T2DM [17,32,33]. Insulin sensitivity and response capacity are significantly higher in women than men [34]. Genetic polymorphisms between the two sexes, differences in the mechanism of action of sex hormones, visceral and hepatic adiposity, hypoadiponectinemia, adiponectin, insulin-sensitive hormones, resting energy expenditure, and lipid metabolism may contribute to higher insulin sensitivity in men than in women [35,36,37,38,39].

The accumulation of excessive body fat (obesity) triggers a broad spectrum of metabolic issues and diseases, comprising insulin resistance, atherogenic dyslipidaemia [high plasma triglyceride and low HDL (high-density lipoprotein) cholesterol content], non-alcoholic fatty liver disease (NAFLD), -cell dysfunction, pre-diabetes, and T2DM [40,41]. Obesity strongly influences T2DM in adults, children, and adolescents [42,43]. It is a serious concern associated with poorer mental outcomes, reduced quality of life, and the leading cause of death worldwide [44]. Obesity increases the risk of developing T2DM by at least a factor of six [17,45]. The prevalence of obesity in adults (age ≥ 20 years) was 38.8% between 2013 and 2016 [46]. If the obesity trends continue, projections are that one in three adults will have type 2 by 2050 [47,48]. The prevalence of T2DM is also positively correlated with the duration of obesity and body mass index (BMI) in childhood [43,49,50]. The proportion of T2DM is higher in people who were obese in childhood and of normal weight in adulthood than in people of normal weight in childhood [43,51]. Obesity and type T2DM represent the greatest threat to the development of atherosclerosis and CAD (coronary artery disease) [52]. These two health conditions are oxidative damage, inflammation, and insulin resistance [5]. Indeed, under diabetic or hyperglycaemic conditions, excess reactive oxygen species (ROS) are released in various tissues and may play a role in developing many complications [53]. This state can persist even when hyperglycaemia is controlled. A disequilibrium occurs with the antioxidant defence systems. This modification scenario is known as oxidative stress [54]. It mainly causes endothelial dysfunction, leading to vascular lesions, the abnormal production of plasma lipids, the activation of platelets and increased coagulation, and the activation of inflammatory processes [54,55]. This damage can be prevented when adequate glycaemic control is established early but is not easily reversed if poor control is maintained over a prolonged period [56]. Oxidative stress causes potential damage to lipids, DNA, and proteins and is responsible for altering intracellular signalling pathways, leading to insulin resistance [57]. The hyperglycemic environment and free fatty acids lead to the appearance of metabolic stress because of an increase in ROS and a change in the mitochondrial electron transport chain [58,59,60]. ROS are considered key signalling molecules that play an important role in the progression of inflammatory disorders, contributing to the development of insulin resistance and the predicted long-term complications of T2DM [57,61]. The activation of the immune system and increased circulating acute-phase inflammatory markers can significantly and directly impact insulin resistance or blood glucose levels [62].

Postprandial hyperglycaemia in people with T2DM can be managed by several approaches, including lifestyle modification, i.e., regular physical activity and a healthy diet [63]. The administration of pharmacological drugs accompanies these measures. Some of these can delay carbohydrate absorption by reducing the digestion of polysaccharides and their bioavailability (e.g., α-glucosidase inhibitors) [64,65]. Others are mainly used to increase the availability of endogenous insulin. These include sulphonylureas, such as Glibenclamide, and glinides, insulin analogues that act on the sulphonylurea receptor in the pancreas to promote insulin secretion. Glucagon-like peptide-1 (GLP-1) agonists and dipeptidyl peptidase-IV (DPP-IV) inhibitors can also increase endogenous insulin by acting on ileal cells in the small intestine. Other drugs used to improve insulin sensitivity include thiazolidinediones, peroxisome proliferator-activated receptor gamma (PPAR*γ*) agonists, and metformin, a biguanide [66,67]. All these drug treatments are prescribed either as monotherapy or with other hypoglycaemic agents [68]. The administration of exogenous insulin remains the primary treatment for some patients with T2DM who are unable to control their blood glucose with oral hypoglycaemic agents [68,69]. If all types of oral hypoglycaemic agents and insulin are administered correctly, and with a healthy lifestyle, people with T2DM can manage and reduce the side effects of the disease. However, certain iterations linked to the risk of hypoglycaemia or co-morbidities have been observed [70]. These occur following a progressive decline in *β*-cell function and a reduction in therapeutic efficacy due to inappropriate or ineffective dosing regimens, altered drug metabolism, lack of target specificity, and solubility and permeability problems [68]. In treated patients, weight gain, weakness, fatigue, lactic acidosis, nausea or diarrhoea, abdominal discomfort, and a metallic taste have been observed [71,72]. 

In this context, medicinal plants have a well-established record of circumventing the problems mentioned about the conventional use of drugs [73]. Medicinal compounds derived from plants could provide new, straightforward approaches to preventing and treating T2DM and its complications [74,75,76]. Traditional knowledge and practices have enabled the development of most modern medicines [73,77]. Many natural resources have been used to develop almost 25% of the major pharmaceutical compounds and their derivatives currently on the market [78,79]. These plant resources have great potential as alternative hypoglycaemic medicines because of their safety, efficacy, affordability, and availability. They constitute an almost unlimited source of bioactive compounds, and their use as antidiabetic agents has been exploited in various ways [74,75]. Secondary metabolites, such as flavonoids, phenolic acids, alkaloids, tannins, terpenoids, saponins, triterpenoids, steroidal glycosides, etc., have shown innumerable promising results against T2DM [80,81,82,83]. They are effective in different stages of diabetes. They can control insulin resistance, impact glucose absorption, and regulate multiple glucose and lipid metabolism stages and the inhibition and/or activation of the expression of genes involved in glucose homeostasis [81]. These natural antidiabetic agents can act alone or with conventional treatments to strengthen the body’s ability to cope with the disease [84]. However, some of these compounds have not yet been studied in depth. As some of the antidiabetic actions of many medicinal plants are still unexplored, researchers are focusing more and more on finding new treatments that work quickly and at lower costs [85]. 

The information needed to assess the efficacy of potentially important medicinal plants and to prove their antidiabetic value must be effective and well-validated [74,86]. One method of sourcing information on medicinal plants is ethnopharmacological studies [87]. They provide rich information from the local community and contribute to discovering and developing natural medicines [86]. An analysis of the medicinal literature concerning the NPSE (the Serra da Estrela Natural Park) shows that documentation on local medicinal plants is weak and almost non-existent, hence the importance of an in-depth study [88,89,90,91,92,93,94,95,96,97,98]. Therefore, to obtain a complete perspective on the potential use of medicinal plants from the NPSE as alternative solutions for combating diabetes, the most relevant studies concerning the botanical diversity, the known traditional uses of local plants, the validation of their antidiabetic activities (in vitro and in vivo studies), the underlying mechanisms of action, their pharmacological activities, the plant-derived chemical compounds that may be responsible for these activities, and the challenges and prospects for the antidiabetic activity of medicinal plants from the NPSE have been critically analysed in this review. 

## 2. Materials and Methods

### 2.1. Geographical and Climate Features of the Serra da Estrela Natural Park

**Figure 1:**
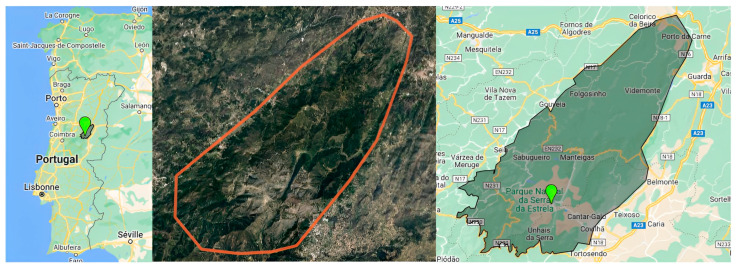
Geolocalization of Serra da Estrela Natural Park [106].

Continental Portugal has several mountain ranges. The highest is in the centre-east, the “Serra da Estrela” (40°20′ N, 7°35′ W) (Figure 1) [99]. Its massif forms the western part of the Cordillera Central, with its highest point called “La Torre” at an altitude of 1993 m [88,89,90,91]. Part of the Iberian Peninsula is traversed by this mountain range, over 500 km long, stretching from almost the Atlantic coast to just north of Madrid [88,89,90,91]. Most of these mountains lie within the boundaries of the NPSE, created in 1976 [100,101,102] and covering around 100,000 hectares [88,89,90,91]. This area is a biological and community interest site integral to the Natura 2000 network [100,101]. Six municipalities (Seia, Gouveia, Celorico da Beira, Guarda, Manteigas, and Covilha) (Figure 1) and two districts (in the north, the district of Guarda, and the south, the community of Castelo Branco) have joined forces to draw up this project [88,89,90,91,100,101,102]. The mountains are mainly composed of granite in the central part and schist in the periphery, dominating the Mondego and the Zezere plains (a tributary of the Tagus) [88,89,90,91]. In the north-east, the landscape is characterized by the watersheds of three major basins: the Douro (the largest river on the Iberian Peninsula), the Tagus (the longest river on the Iberian Peninsula), and the Mondego (the largest river in Portugal) [88,89,90,91].

The climate of Serra da Estrela is influenced by several factors: temperature, atmospheric pressure, wind, humidity, and precipitation, as well as geographical factors [103,104]. Its high altitude among the surrounding land, the general organisation of the relief, and the relative proximity of the Atlantic Ocean, some one hundred kilometres away, play a decisive role in the complex mosaic of local climates that characterise the region [88,89,90,91]. Thus, all the climate factors are controlled by the overall latitudinal position of the mountains and influenced by the north–south temperate climate and the southeast-northwest Mediterranean macroclimate [99,103,104]. They are also controlled by the Atlantic’s longitudinal position and the Iberian Peninsula’s interior (maritime influences mainly to the west and continental influences to the east and west) [88,89,90,91]. Average annual rainfall is around 2500 mm at the summit, while the plateaux record is more than 2000 mm [88,89,99,105]. The highest number of precipitation days is observed in the western part of the mountains, while the lowest values are in the basal areas, in the north-western and south-eastern sectors, with around 1000 to 1200 mm [88,89,90,99,105]. The region is characterized by hot, dry summers and wet winters, with snowfall more frequent between December and March [99,103,104]. The most striking aspects of the relief are the glacial forms and deposits. The snowfall is heaviest in the higher mountain areas but lightest and most irregular in the lower regions [88,89,90,91].

### 2.2. Ethnobotanical Data Collection and Selection Criteria

The information on the plant species of the Serra da Estrela region has been collected from the databases of the World Checklist of Vascular Plants [107]. It is an international collaborative programme with editors, compilers, and reviewers from all over the world. The main objective of the database is to provide high-quality, expertly reviewed taxonomic data on all vascular plants based on the nomenclatural data provided by the International Plant Name Index (IPNI). 

**Figure 2:**
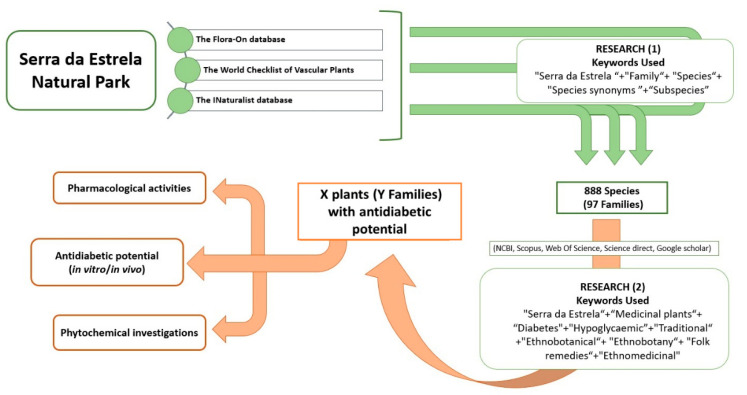
Diagram of the systematic literature review.

The Flora-On database is also used; it is a portal coordinated by the Portuguese Botanical Society containing photographic, geographical, morphological, and ecological information for all vascular plant species in Portugal [108]. The search was supported by the INaturalist database [109]. It is a joint initiative of the California Academy of Sciences and the National Geographic Society. It is also a species identification system and a tool for recording the occurrence of organisms. It can be used to record sightings, get help with identifications, collaborate with others to collect information for a common purpose, or access sighting data collected by iNaturalist users [109]. All databases were screened using a combination of the keywords “Family”, “Species”, “Species Synonyms”, and “Subspecies”. This approach enabled us to find 97 different families. The total number of species is 888 (a complete list of the taxa is given in Table S1). Based on this list of plants, a systematic literature search on their traditional and diabetic uses was conducted. Data were obtained from scientific databases, including NCBI, Scopus, Web of Science, and Google Scholar (Figure 2). The preliminary selection was initially performed using the search terms “Serra da Estrela” and “medicinal plants” to cover the maximum range of medicinal plants used against diabetes. As the number of studies was small, we carried out another selection, but universal, by searching by keyword for all medicinal plants from the Serra da Estrela region and their possible worldwide uses. The Boolean operator “AND” followed by the keyword “Diabetes” or “Hypoglycaemic” was used for this search to cover literature reports dealing exclusively with T2DM anywhere in the world. This search was carried out specifically for each plant on the databases, using the leading taxonomic designation of the species and other botanical synonym names, followed by the keywords mentioned. The names of each plant and combinations of the terms “Traditional”, “Ethnobotanical”, “Ethnobotany”, “Folk remedies”, and “Ethnomedicinal” were used to search the above databases (Figure 2). 

## 3. Results

### 3.1. Botanical Diversity of NPSE and Ethnopharmacological Uses of Medicinal Plants with Antidiabetic Potential

Mountains have always been an excellent challenge for humankind, who have never ceased climbing, cultivating, and domesticating. They are open-air laboratories of knowledge, home to species and communities that have adapted to their environment in various ways. They provide fertile ground for observing and understanding the evolution of species and the distribution of organisms in similar contexts, from one mountain to another thousands of kilometres away. They are, therefore, important ecosystems because they harbour high levels of biodiversity and endemicity [110]. They provide essential services such as climate regulation, freshwater supply and purification, and nutrient cycling [111,112,113,114,115]. 

The inaccessible NPSE vegetation is the best preserved in the region [88,89]. The isolation of the summits and the extreme conditions that prevail there have encouraged the appearance of new species and facilitated species isolation, speciation, extinction, and migration [114]. According to Jansen et al. [88], the flora of this mountainous region shows significant contrasts as you go up in altitude. The vegetation is divided into several levels, the boundaries of which vary according to exposure. Within this tiering, the transitions in vegetation are distinguishable, and each level corresponds to a well-defined ecosystem. In addition, the isolation of the summits and the extreme prevailing conditions have encouraged the appearance of new species. Many species are endemic to these areas, making the NPSE one of the wealthiest regions in Portugal for certain groups of plants [88,89].

The climatic heterogeneity contributing to the region’s high biodiversity has attracted botanists’ interest [99]. Floristic expeditions from the 18th century to the present day have enabled a rigorous characterisation of the ecosystems’ flora, which is essential for their in-depth knowledge and conservation [93]. According to the three bioclimatic levels (Meso-Mediterranean, Supra-Mediterranean, Oromediterranean) defined in the Serra da Estrela region, three vegetation ranges (basal, intermediate, and upper) have been characterized. The three combined levels have identified approximately 900 vascular species and subspecies [88]. Endemic Iberian species belonging to the Mediterranean and Atlantic flora are particularly well represented and distributed in an area more or less delimited by the altitude of the massif and the climatic, edaphic, and sun exposure conditions [88,89,91,102]. Some relict plant populations from northern and central Europe have also invaded the area [88].

The botanical census of NPSE diversity enabled us to identify 97 families, 112 genera, and 888 vascular species (after eliminating synonyms) (Table S1). The number of native species on the Iberian Peninsula is 133, while there are only 9 endemic species in Portugal. By contrast, the number of introduced species is 36 [107,108,116].

The *Asteraceae* is the family with the most species/subspecies (sp) in the region (108 sp). It is followed by *Poaceae* (81 sp), *Fabaceae* (74 sp), *Caryophyllaceae* (60 sp), *Brassicaceae* (33 sp), *Apiaceae* (29 sp), *Lamiaceae* (29 sp), *Rosaceae* (28 sp), *Plantaginaceae* (27 sp), *Polygalaceae* (22 sp), *Ranunculaceae* (21 sp), *Cyperaceae* (19 sp), *Juncaceae* (18 sp), *Rubiaceae* (17 sp), *Cistaceae* (16 sp), *Amaryllidaceae* (15 sp), and *Crassulaceae* (14 sp). 

**Figure 3:**
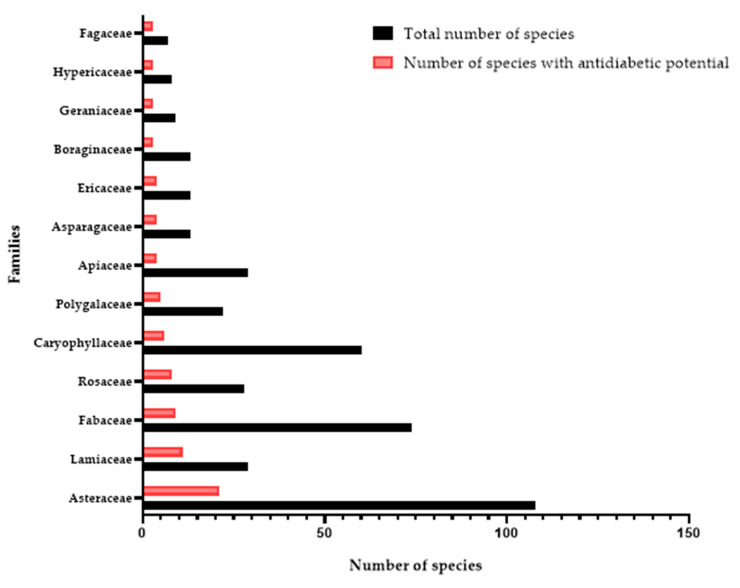
Families containing the highest number of species with antidiabetic potential (out of 888) in NPSE.

Several plants found in the NPSE have been used to treat diabetes. Despite the relatively large number of studies worldwide reporting their biological potential, NPSE species have been little investigated, and species with antidiabetic potential will be the subject of particular attention in the following section. Of the 888 species listed, only 138 plants (15.54%) from different geographical regions have been selected based on traditional uses and studies into their antidiabetic potential (Table S2). The families with the highest number of species are *Asteraceae* (21 sp), *Lamiaceae* (12 sp), *Fabaceae* (9 sp), *Rosaceae* (8 sp), *Caryophyllaceae* (6 sp), and *Polygalaceae* (5 sp) (Figure 3). The *Apiaceae*, *Asparagaceae*, and *Ericaceae* contain four species/subspecies, and *Boraginaceae*, *Geraniaceae*, *Hypericaceae*, and *Fagaceae* comprise three (Figure 3). However, the families *Brassicaceae*, *Cistaceae*, *Amaryllidaceae*, *Scrophulariaceae*, *Papaveraceae*, *Pteridaceae*, *Caprifoliaceae*, *Gentianaceae*, *Urticaceae*, *Malvaceae*, *Cupressaceae*, *Cytinaceae*, and *Pinaceae* present only two species with antidiabetic potential. As for the rest, 30 families have only one species, and 42 families have never been traditionally used or studied for their effects on diabetes (Table S2). 

The antidiabetic plants belong to fifty-five families (6.20% of the total families of NPSE) and have been reported in the literature for various traditional uses (Supplementary Table S3). The number of species for which evidence of traditional use against diabetes had been found was 83 (Supplementary Tables S3 and S4). The parts used and the preparation method vary from one plant to another. In most cases, the plant parts were used singularly and sometimes as a combination of two or more parts. However, there are 55 other species whose traditional use has not been revealed. They have, however, been studied for their antidiabetic efficacy (Supplementary Table S4).

#### 3.1.1. *Asteraceae*

The *Asteraceae* family has the most significant number of plants with antidiabetic potential. Twenty-one species were selected, or 19.44% of all *Asteraceae* species and 2.17% of all species identified in the NPSE (Tables S1 and S2). The *Asteraceae* family includes many flowering plants in nearly 1600 genera, comprising more than 23,000 species [117,118]. The *Asteraceae* are herbs, shrubs, trees, or lianas, with laticifers or resin ducts in some taxa [119,120]. Leaves are simple or compound, spiral or opposite [rarely whorled], and exstipulate. The most distinctive feature of the Asteraceae is their inflorescence structure: the highly compressed inflorescence branch system called a capitulum or flower head, in which all the flowers are attached to a receptacle surrounded by involucral bracts [119,120]. The *capitulum* forms a *pseudanthium*, a structure resembling a single large flower. The anthers, which include a tube, and the lower position of the ovary are other features that help to diagnose the family [121]. Modifying the outer floral whorl into pappus bristles, which help disperse the seeds, is also widespread in the family. The fruit is an achene (or “cypsela”, an achene derived from an inferior ovary), typically multiple fruits of achenes, with an elongated beak forming between the fruit and the pappus in some taxa. The seeds are exalbuminate [119,120]. 

Members of the *Asteraceae* family are distributed worldwide; some of these species are highly aromatic and have already been reported to have medicinal and therapeutic applications. For centuries, they have been used worldwide as traditional medicine against various human ailments, including T2DM, kidney, heart, lung, liver, and skin toothache inflammation, pain, constipation, toothache, throat pain, snake bite, headache, gastrointestinal disorders, diarrhoea, dysentery, tuberculosis, hepatitis, asthma, menopausal and menstrual disorders, stomach ulcers, sores, scabies, filariasis, elephantiasis, night-blindness, impotence, hair fall, jaundice, nose bleeding, allergies, viral infections, cough, bronchitis, different types of cancers, wounds and cuts, and malaria [122,123,124,125,126,127,128].

In the NPSE, only 19 of the 55 genera listed have been studied for their antidiabetic potential (Tables S1 and S2). Various biological activities have been reported for these *Asteraceae* species worldwide [118,123,125,126,127,128,129,130]. The species *Arctium minus* (Hill) Bernh, *Achillea millefolium*, *Anthemis canescens* (syn. *Matricaria aurea*), *Arnica montana* subsp. Atlantica, *Bellis perennis*, *Bidens frondosa*, *Calendula arvensis*, *Chamaemelum nobile* (syn. *Matricaria chamomilla* or *Matricaria recutita*), *Cichorium intybus*, *Dittrichia viscosa* subsp. Viscosa (Syn. *Inula viscosa*), *Galinsoga parviflora* (Syn. *Galinsoga quadriradiata*), *Helichrysum stoechas* subsp. *Stoechas*, and *Hypochaeris radicata* have been used extensively in traditional medicine to treat diabetes [131,132,133,134,135,136,137,138,139,140,141,142,143]. Different parts include flowers, leaves, seeds, stems, and roots. However, no evidence exists of using other species, *Anthemis canescens*, *Bellis perennis*, *Bidens frondose*, *Helichrysum stoechas* subsp. *Stoechas*, *Lactuca serriola*, *Onopordum acanthium* subsp. *Acanthium*, *Senecio vulgaris*, *Tanacetum parthenium*, and *Tanacetum vulgare* (Supplementary Tables S3 and S4).

#### 3.1.2. *Lamiaceae*

The *Lamiaceae* or *Labiatae* are a family of flowering plants with a cosmopolitan distribution, comprising around 236 genera and an estimated 6900 to 7200 species [144]. In continental Portugal, it is represented by 29 genera with 95 different species [108]. They are herbs or shrubs, often aromatic with ethereal oils, with generally four-sided stems, opposite (or verticillate) leaves, a verticillate or thyrse inflorescence (solitary and axillary flowers in some cases), and zygomorphic (rarely actinomorphic) flowers, usually bilabiate, with a superior ovary, often deeply four-lobed (by the formation of “false septa”) with a gynobasic style, the fruit being a schizocarp of usually four nuts or a berry or a drupe [119]. Since antiquity, the family has contained many culinary or flavouring herbs widely used as spices, teas, or traditional medicines. Several of its members are also used as sources of essential oils (EO) [145]. They have been reported as a rich source of antidiabetic plants [146]. 

Based on database analysis, twelve species were selected with antidiabetic potential, representing 37.93% of all *Lamiaceae* species and 1.13% of all species identified in the NPSE (Tables S1 and S2). Among these species are those traditionally used to treat diabetes, including *Clinopodium nepeta* subsp.* Spruneri* (Syn. *Calamintha officinalis* Moench), *Lavandula stoechas*, *Mentha aquatica*, *Mentha pulegium*, *Mentha suaveolens*, *Origanum vulgare*, *Prunella vulgaris*, and *Salvia verbenaca* [147,148,149,150,151,152,153,154,155,156,157,158,159]. To our knowledge, there is no record of *Lavandula pedunculata* subsp. *Pedunculata*, *Melissa officinalis*, *Origanum vulgare* subsp.* Virens*, and *Thymus mastichina* being used to treat diabetes in folk medicine (Supplementary Tables S3 and S4).

#### 3.1.3. *Fabaceae*

The *Fabaceae* (or *Leguminosae*) are one of the world’s twelve flowering plants after the *Orchidaceae* and *Asteraceae*, with no fewer than 19,400 species grouped into 740 genera [160]. Thanks to its ability to form root nodules with nitrogen-fixing bacteria [161], this family covers the entire globe in various habitats, with representatives in almost every biome, from deserts to tropical forests [160]. They grow as shrubs, trees, and even aquatic plants, have a diverse floral morphology, and are adapted to various ecological and climatic conditions. Most species in this family are of significant economic value [162]. Thanks to their nitrogen-fixing behaviour, these plants can produce large quantities of protein, a nutritional source for animal and human consumption [163,164]. They are also considered a good source of fibre, carbohydrates, minerals, and vitamins. The *Fabaceae* members are superior to other dietary supplements due to their low-fat content compared with most cereals [163]—the resistant starch and fibre act as prebiotics for probiotics or beneficial bacteria [164]. Micronutrients are also essential for reducing anaemia risk [164]. The consumption of most *Fabaceae* species helps moderate blood sugar levels after meals and improves insulin sensitivity. It also positively impacts sight reduction by inducing satiety [165].

In the Serra da Estrela region, nine species had antidiabetic benefits out of 79 species of *Fabaceae* (12.16%) listed in the area (Tables S1 and S2), representing 0.93% of all the species found in the NPSE. These species comprise *Acacia dealbata*, *Lupinus angustifolius*, *Lupinus luteus*, *Pisum sativum*, *Pterospartum tridentatum*, *Retama sphaerocarpa*, *Robinia pseudoacacia*, *Trifolium pratense* subsp. *Pratense*, and *Trifolium repens.* Only the four species (*Pisum sativum*, *Pterospartum tridentatum*, *Retama sphaerocarpa*, and *Trifolium pratense* subsp. *Pratense*) have been registered as being used in traditional medicine in ancient times (Supplementary Table S3). All parts of these plants (leaves, stems, roots, and flowers) are traditionally used to combat various ailments. Pollen, bark, gum, seeds, fruit, and even cladodes are also used. As far as diabetes is concerned, only the species *Pisum sativum*, *Pterospartum tridentatum*, *Retama sphaerocarpa*, and *Trifolium pratense* are recorded as being traditionally used to treat it (Supplementary Tables S3 and S4).

#### 3.1.4. *Rosaceae*

The *Rosaceae* family include species of herbs, shrubs, or trees. They are sometimes rhizomatous, climbing, or thorny and are cosmopolitan or sub-cosmopolitan [119,166]. They are very diverse, particularly in the northern hemisphere, and are very important from an economic point of view, as they are the source of many cultivated fruits. These species are economically and ecologically beneficial, providing habitat anchorage [167] and timber [168]. Herbaceous species of the *Rosaceae* grow in temperate forests as understorey plants, in salt or freshwater marshes, in arctic tundra, in old fields, and along roadsides [119,166]. Woody species are pioneer species that play an essential role in the early stages of forest succession. *Rosaceae* can also be a minor component of mature mixed deciduous forests [119,166]. Their leaves are spiral (rarely opposite), simple or compound, undivided or divided, generally stipulate (lost in some taxa), and the stipules often adnate at the base of the petiole. The inflorescence is variable. The flowers are bisexual (generally), actinomorphic, perigynous, or epi perigynous; the receptacle is sometimes enlarged or sunken [119,166]. The fruit is a drupe, pome, hip, follicetum, achenecetum, or capsule. The seeds typically have no endosperm [119,166]. Eight species of *Rosaceae* have been identified in the Serra da Estrela region (Tables S1 and S2), including *Agrimonia eupatoria*, *Crataegus monogyna*, *Geum urbanum*, *Potentilla erecta*, *Prunus avium*, *Prunus lusitanica* subsp. *lusitanica*, *Rosa canina*, and *Sorbus aucuparia*. These species have traditionally been used to treat diabetes, except for *Prunus lusitanica* subsp. *Lusitanica*.

#### 3.1.5. *Caryophyllaceae*

The *Caryophyllaceae* family, commonly known as the rose or carnation family, comprises 104 genera and over 2000 species. They are annual or perennial herbs or small erect or prostrate shrubs; some species are more prominent or small trees. The species are distributed over almost the entire globe, with the centres of biodiversity being in Europe and Asia’s moderate to warm regions [119,169,170]. They are also concentrated in the Mediterranean region, with various habitats and growth forms [169]. The *Caryophyllaceae* are distinguished by their often-swollen nodes; simple, opposite leaves; an inflorescence of solitary flowers or dichasial cymes; actinomorphic, biseriate flowers, usually pentamerous with distinct, clawed petals; an upper ovary with free or basal distal placentation; and a capsular fruit in which only anthocyanin pigments are present [119,169]. An unusual feature of this family is the stable, long-lasting foam that appears when plant parts are placed in water and shaken [170]. This behaviour is due to saponins, which can be as high as 20% (dry weight) in some species. The most significant quantity of saponins is generally found in the roots or seeds and can vary depending on the growing period, the part of the plant, and the season [170]. 

*Corrigiola litoralis*, *Corrigiola telephiifolia*, *Paronychia argentea*, *Saponaria officinalis*, and *Stellaria media* are all plants belonging to the *Caryophyllaceae* family found in the NPSE that have traditionally been used to treat diabetes (except *Spergularia rubra*). The leaves and roots are the most widely used parts of the plants identified (Supplementary Tables S3 and S4).

#### 3.1.6. *Polygalaceae*

The word *Polygalaceae*, or Milkwort family, comes from a Greek name meaning “much milk”, as certain species eaten by cows are thought to increase milk production [119]. This family is almost cosmopolitan (absent only from New Zealand, many islands in the South Pacific, Antarctica, and the Arctic), with many genera having a wide distribution [171]. The family has many habits, from rainforest trees to small achlorophyllous grasses, including annual and perennial herbs, lianas, and shrubs of various sizes [119,171]. The family comprises 22 genera and between 800 and 1000 species [171], characterized by simple, spiral-shaped leaves that are generally exstipulate (modified by a pair of glands or spines in some cases). Their inflorescence is a spike, raceme, or panicle. The flowers are bisexual, zygomorphic [rarely almost actinomorphic], hypogynous to perigynous, and subtended by a pair of bracteoles. The fruit is a loculicidal capsule, nut, samara, or drupe. The seeds are arillate (with a wattle) and endospermic (proteinaceous) [119].

The species in the *Polygalaceae* family with antidiabetic potential identified in the NPSE are *Polygonum aviculare*, *Polygonum hydropiper*, *Rumex acetosa* subsp. *acetosa, Rumex crispus*, and *Rumex obtusifolius* (Supplementary Table S3)*. Polygonum hydropiper* and *Rumex obtusifolius* have never traditionally been used to treat diabetes, but scientific evidence shows they are effective against the disease (Supplementary Table S4). 

#### 3.1.7. Other families

The families *Apiaceae* (*Daucus carota*, *Eryngium campestre*, *Foeniculum vulgare*, *Heracleum sphondylium*), *Asparagaceae* (*Muscari comosum*, *Polygonatum odoratum*, *Ruscus aculeatusi*, *Urginea maritima*), and *Ericaceae* (*Arbutus unedo*, *Erica scoparia* subsp. *Scoparia*, *Vaccinium myrtillus*, *Vaccinium uliginosum*) include four species whose antidiabetic potential has been studied (Supplementary Tables S3 and S4). However, only three species have been identified for the families of *Boraginaceae*, *Geraniaceae*, *Hypericaceae*, and *Fagaceae*. The species are *Anchusa undulata*, *Echium plantagineum*, *Lithodora prostrata*, *Geranium purpureum*, *Geranium pyrenaicum* subsp. *Lusitanicum, Geranium robertianum*, *Castanea sativa*, *Quercus pyrenaica*, and *Quercus suber* (Supplementary Table S3).

The families *Amaryllidaceae*, *Brassicaceae*, *Caprifoliaceae*, *Cistaceae*, *Cupressaceae*, *Cytinaceae, Gentianaceae, Malvaceae, Papaveraceae, Pinaceae, Scrophulariacea,* and *Urticaceae*, each of which is represented by just two species with antidiabetic potential, include *Allium victorialis, Narcissus pseudonarcissus, Capsella bursa-pastoris, Raphanus raphanistrum subsp. raphanistrum, Lonicera periclymenum, Sambucus nigra, Cistus ladanifer, Cistus salviifolius, Juniperus communis, Juniperus communis* subsp. *alpina, Cytinus hypocistis, Cytinus hypocistis* subsp. *hypocistis*, *Centaurium erythraea*, *Gentiana lutea* subsp. *lutea*, *Malva neglecta*, *Malva sylvestris*, *Chelidonium majus*, *Papaver dubium*, *Pinus pinaster*, *Pinus sylvestris*, *Verbascum sinuatum*, *Verbascum thapsus*, *Urtica dioica*, and *Urtica membranacea* (Supplementary Table S3).

A single species has been identified in the following families *Amaranthaceae* (*Chenopodium ambrosioides*), *Betulaceae* (*Corylus avellana*), *Buxaceae* (*Buxus sempervirens*), *Campanulaceae* (*Jasione montana* var. *gracilis*), *Cannabaceae* (*Humulus lupulus*), *Convolvulaceae* (*Convolvulus arvensis*), *Cucurbitaceae* (*Bryonia dioica*), *Dioscoreaceae* (*Tamus communis*), *Dryopteridaceae* (*Dryopteris dilatata*), *Juncaceae* (*Juncus acutus*), *Lauraceae* (*Laurus nobilis*), *Lycopodiaceae* (*Lycopodium clavatum), Lythraceae* (*Lythrum salicaria*), *Moraceae* (*Ficus carica*), *Myrtaceae* (*Eucalyptus globulus*), *Oleaceae* (*Olea europaea* var. *europaea*), *Oxalidaceae* (*Oxalis pes-caprae*), Phytolaccaceae (*Phytolacca americana*), *Poaceae* (*Avena sativa*), *Portulacaceae* (*Portulaca oleracea*), *Pteridaceae* (*Adiantum capillus-veneris* L.), *Primulaceae* (*Anagallis arvensis*), *Rubiaceae* (*Galium aparine*), *Simaroubaceae* (*Ailanthus altissima*), *Solanaceae* (*Solanum nigrum*), *Taxaceae* (*Taxus baccata*), *Thymelaeaceae* (*Daphne gnidium*), *Ulmaceae* (*Ulmus glabra*), *Verbenaceae* (*Verbena officinalis*), and *Vitaceae* (*Vitis vinifera* subsp. *sylvestris*) (Supplementary Tables S3 and S4). Finally, no species have been traditionally used or studied for its antidiabetic potential in the rest of the families listed (42) in the NPSE (Table S2).

### 3.2. Medicinal Plants with Antidiabetic Potential in NPSE

#### 3.2.1. *Asteraceae* Family

- *Arctium minus* (Hill) Bernh.

*Arctium* species are known for their pharmacological effects and chemical diversity [172]. These plants, also known as “burdock”, are biennial herbs found in waste ground, streams, and roadsides, more rarely in woods and forests, in temperate regions of Europe and Asia, and sporadically in subtropical and tropical regions [172]. Several Arctium plants have also been reported in folk medicines for T2DM. Among its most investigated members is the species *Arctium minus* (Hill) Bernh (Table 1). Its extracts exert antihyperglycaemic properties through various mechanisms. According to İlgün et al. [173], only the leaf extracts (excluding leaf ethyl acetate extract) showed *α*-amylase inhibition activity at a 1 mg/mL concentration (Table 2). In the *α*- glucosidase inhibition assay, the dichloromethane extract of the *A. minus* leaf had the highest enzyme inhibition activity, with 87.12% inhibition, compared with the other extracts and with acarbose at a concentration of 1 mg/mL [173]. The hypoglycaemic activity of the crude aqueous extract of the leaves and roots of *A. minus* was also tested in alloxan (ALO)-induced diabetic rats [174].

In this study, the aqueous extract of the leaves caused a 6.2% reduction in blood sugar levels in the rats. The same result was observed with the positive control Glibenclamide. These results are still better than those of the aqueous root extract (5.8%). In any case, these results prove the hypoglycaemic activity of this species [174]. Arctium roots contain inulin, the common name for all linear fructans (insulin-like fructans, ITF), a type of indigestible carbohydrate that is more or less polymerised [175]. It comprises fructose units (2 to 60 units) and a terminal glucose unit. Because of its complex structure, inulin resists breakdown by the digestive enzymes of the small intestine, which are specific to *α*-glycosidic bonds; the compound is therefore classified as a “non-digestible” oligosaccharide [175]. When inulin remains in the upper gastrointestinal tract, it is fermented by the microbial flora of the colon (or large intestine) to produce short-chain fatty acids (SCFAs), which serve as a source of energy for the resident bacteria while exerting numerous other effects on the health of the host [175]. Inulin promotes the growth (i.e., an increase in the number) of specific health-promoting intestinal micro-organisms, thereby positively modifying the intestinal ecosystem, in addition to inulin–host interaction or immunomodulatory effects [176,177]. In this way, dietary inulin-induced changes to the microbiota could improve type 2 diabetes mellitus [175,178,179,180]. The intestinal symbiosis supported by supplementation with inulin, among other dietary fibres, provides preventive and/or therapeutic options for many metabolic disorders, including obesity, type 2 diabetes mellitus, cardiometabolic diseases, kidney disease, and hyperuricaemia [175]. 

As a result, *A. minus* roots used by diabetic patients can slow the digestion of carbohydrates, reduce absorption, and control glucose intolerance [181]. However, controversial results have been obtained by Fereira et al. [182]. In their study, the plant did not control hyperglycaemia in a Goto-Kakizaki (GK) rat model. The plant extract was prepared from a plant sample from a Portuguese herbalist. However, analysis of the plant extract revealed the presence of heavy metals, nickel (Ni) and cadmium (Cd), which could inhibit insulin release and have toxic effects on rats [182]. According to the authors (Table 2), all medicinal plants may contain them, as they can bioaccumulate several heavy metals. These results could be attributed to the different animal models of diabetes, the conditions of experimentation, and the different chemotypes investigated [174,182]. Several studies have demonstrated the richness of this plant in bioactive compounds. *Arctium minus* is rich in polysaccharide compounds, flavonoids, phenolic acids, and the lignan Arctiin. These chemical compounds are associated with the diverse biological activities observed by the plant [183,184,185], which are helpful to diabetic patients in reducing oxidative stress and the common low-grade inflammation related to the disease [186,187].

**Table 1:** NPSE medicinal plants reported constituents to pharmacological use.

Pharmacological Uses	Chemical Constituents	References
*Asteraceae*		
*Arctium minus* (Hill) Bernh		
Antimicrobial, antioxidant, anti-inflammatory, antinociceptive, acetylcholinesterase inhibitory activities, anti-cancer.	Phenolic acids: Rosmarinic acid, quinic acid, caffeic acid, chlorogenic acid, cynarin, hydroxy cinnamoyl quinic acid. Flavonoids: Rutin, isoquercetin, luteolin kaempferol-3-*O*-rhamnoglucoside, quercimeritrin, astragalin, arabinose, rhamnose, mannose, cellulose, inulin. Polysaccharides: Pectic substance, rhamnogalacturonan, hemicellulose (arabinan, arabinogalactan, galactan, xylan, xyloglucan, galacturonic acid, glucose, galactose.	[118,173,183,184,185,188,189]
*Achillea millefolium*		
Anxiolytic, antimicrobial, antioxidant, vasoprotective, vasorelaxant, anti-appetite (orexigenic), anti-tumor, anti-ulcerogenic, hypotensive, analgesic, modulation of mitochondria respiration, anti-inflammatory, anti-neuroinflammatory, anti-proliferative, antiplatelet, skin-rejuvenating, antinociceptive, hepatoprotective, antiplasmodial, anthelmintic, antispasmodic, anti-cancer, antispermatogenic, for haemorrhoids and dysmenorrhea.	Phenolic acids: *Cis* and *trans*-3,5-*O*-dicaffeoylquinic acids, chlorogenic acid, *p*-coumaric acid, neochlorogenic acid, ferulic acid, stachydrine. Flavonoids: Resveratrol, morin, myricetin, naringin, naringenin, apigenin, quercetin, luteolin *O*-acetylhexoside, apigenin *O*-acetylhexoside, centaureidin, casticin, artemetin, luteolin 7-glucoside, luteolin 4′-*O*-glucosid, apigenin 7-glucoside, apigenin 4′-*O*-*α*-glucopyranoside, 5-Hydroxy-3,6,7,4′-tetramethoxyflavone, kaempferol, isorhamnetin glycosides, rutin, cynaroside, cosmosiin, vicenin-2. Sesquiterpenoids: paulitin, isopaulitin, psilostachyin C, desacetylmatricarin, sintenin, achillicin, 8a-(Angeloyloxy), artabsin 1,4-endoperoxide, 8a-(Tigloyloxy)artabsin 1,4-endoperoxide, 7b-Hydroxy-a-longipin-2-en-1-one, a-Longipin-2-en-1-one (longipinanes), Millefoliumins F and G, leucodin, 8*α*-angeloxy-leucodin, achillin, 8*α*-angeloxy-achillin, desacetylmatricarin. Organic acids and phenols: oxalic, quinic, citric acids, fatty acids (with linoleic and palmitic acids), tocopherols (*γ*-tocopherol), ascorbic acid, carboxylic acid, salicylic acid, thymol, carvacrol, pyrocatechol, adenine, mandelic acid, methyl esters of caprylic-linolenic-undecylenic acid.	[172,190,191,192,193,194,195,196,197,198,199,200,201,202,203,204,205,206,207,208,209,210,211,212,213,214,215,216]
*Anthemis canescens* (syn. *Matricaria aurea*)		
Antioxidant, anti-inflammatory, anti-ulcer, analgesic, antibacterial, anti-cancer.	Phenolic acids: *p*-coumaric acid, ferulic acid, shikimic acid, protocatechuic acid, *p*-aminobenzoic acid, digalloyl-shikimic acid, epicatechin, *p*-hydroxybenzoic acid, rosmarinic acid, 7,8-dihydroxycoumarin, chlorogenic acid, 1-*O*-*b*-d-glucopyranosyl sinapate, 5-methoxysalicylic acid. Flavonoids: Apigenin, apigenin-7-*O*-rhamnoglucoside (Rhoifolin), apigenin 8-C-glucoside, apigenin-7-*O*-glucoside, 4′-Methoxyapigenin (Acacetin), luteolin, luteolin-6-C-glucoside, quercetin, quercetin-3-D-xyloside, quercetin-7-*O*-rhamnoside, quercetin-3-arabinoside, quercitrin, kaempferol-3-glucuronide, kaempferol-3-*O*-alpha-L-rhamnoside, kaempferol-3-*O*-alpha-L-arabinoside, Kaempferide, eriodictyol-7-*O*-glucoside, baicalin, isovitexin 7-*O*-glucoside (saponarin), syringetin-3-*O*-galactoside, rhamnetin, isorhamnetin, isorhamnetin-3-*O*-rutinoside, isorhamnetin-3-*O*-glucoside, myricitrin, daidzein-8-C-glucoside, cyanidin-3-glucoside, myricetin, diosmetin 7-*O*-rutinoside, hesperetin-7-*O*-neohesperidoside, maritimetin-6-*O*-glucoside, acacetin-7-*O*-neohesperidoside, acacetin-7-*O*-rutinoside, naringenin, esculetin, formononetin, resveratrol, eriodictyol. Others: Anthocyanins (delphinidin-3-rutinoside), terpenes alkaloids (gibberellin A4), chalcones (Okanin-4′-*O*-glucoside), coumarins (Scopoletin, 4-methylumbelliferone).	[217,218,219,220,221,222]
*Arnica montana*		
Antiphlogistic, inotropic, antibiotic, anti-inflammatory, immunomodulatory, antiplatelet, uterotonic, anti-rheumatic, anti-osteoarthritic, antimicrobial, improve circulation, increase respiration, ureotonic, antioxidant, hepatoprotective, insecticidal, hypopigmentation, antihair loss, anticough, antihaemorrhagic and analgesic in febrile conditions.	Phenolic acids: Chlorogenic acid, 3,5-dicaffeoylquinic acid, 4,5-dicaffeoylquinic acid Flavonoids: Kaempferol 3-*O*-glucoside, 6-methoxy-kaempferol 3-*O*-glucoside, hispidulin, quercetin 3-*O*-glucoside, quercetin 3-*O*-glucuronic acid, patuletin 3-*O*-glucoside, luteolin, apigenin. Sesquiterpene lactones: Helenalin,11a,13-dihydohelenalin. Others: Carotenoids, diterpenes, arnidiol, 2-pyrrolidineacetic acid, pyrrolizidine alkaloids (tussilagine and isotussilagine), polyacetylenes, coumarins (umbelliferone and scopoletin), lignans, dicaffeoyl quinic derivatives (1,3-3,5 and 4,5 dicaffeoyl quinic acids), umbelliferone, scopoletin, oligosaccharides, sesquiterpene lactones (2,3-dihydroaromaticin, chamissonoid, mexicanin 1).	[223,224,225]
*Bellis perennis*		
Wound healing, anxiolytic, anti-tumor, antibacterial, anti-fungal, anti-hyperlipidemic, antioxidant, postpartum anti-hemorrhagic, pancreatic lipase inhibitor, and cytotoxic activities.	Phenolic acids: Chlorogenic acid, neochlorogenic acid, rosmarinic acid, caffeoylquinic acids. Flavonoids: Isorhamnetin 3-*O-β*-d-galactopyranoside, isorhamnetin 3-*O*-*β-*d-(6 ″-acetyl)-galactopyranoside and kaempferol 3-*O*-*β*-d-glucopyranoside. Triterpene saponins: Perennisosides I-VII, bellidioside A, asterbatanoside D, bernardioside A/F/B2, bellissaponin BS6/BA1/BA2, Anthocyanins: Cyanidin 3-*O*-(4″-*O*-(malonyl)-2″*O*-(*β* d-glucuronyl)-*β*-d-glucopyranoside), cyanidin 3-*O*-(2″-*O*-(*β*-d-glucuronyl)-*β*-d-glucopyranoside), cyanidin 3-*O*-(6″-*O*-(malonyl)-2″-O-(*β*-d-glucuronyl)-*β*-d-glucopyranoside).	[226,227,228,229,230,231,232,233,234,235,236,237]
*Bidens frondose*		
Antibacterial, antioxidant, antidiarrheal, anti-malarial, anti-inflammatory, allelopathic.	Phenolic acids and their ethers: Caffeic acid, 4,5-di-*O* caffeoylquinic acid 1-methyl ether, isoferuloyl ethyl ester, protocatechuic acid. Flavonoids: Okanin-4′-*O*-(6″-*O*-acetyl-2”-*O*-caffeoyl-6″-*O*-glucopyranoside), okanin-4′-*O*-(2”-*O*-caffeoyl-6″-*O*-*p*-coumaroyl-*β*-D-glucopyranoside), 4-*O*-methylokanin-4′-*O*-(6″-*O*-*p*-coumaroyl-β-D-glucopyranoside), 4-*O*-methylokanin-4′-*O*-(6″-*O*-acetyl-*β*-D-glucopyranoside), 4-*O*-methylokanin-4′-*O*-(6″-*O*-acetyl-2”-*O*-caffeoyl-*β*-D-glucopyranoside), okanin-4′-*O*-(6″-*O*-*p*-coumaroyl *β*-D-glucopyranoside), okanin-4′-*O*-(6″-*O*-acetyl-*β*-D-glucopyranoside), (Z)-6″-*O*-*p*-coumaroyl-maritimein, (Z)-6″-*O*-acetylmaritimein, apigenin, luteolin, luteolin-7-*O*- *β*-D-glucopyranoside, luteolin-7-*O*-(*β*-dglucopyranosyl)-2-glucopyranoside, kaempferol-3-*O*-*β*-D-glucopyranoside, quercetin-3-*O*-*β*-D-glucopyranoside, 8,3′,4′-trihydroxyflavone-7-*O*-(6′′-*O*-*p*-coumaroyl)-*β*-D-glucopyranoside, 6-hydroxyluteolin-7-*O*-glucoside, 3′′-(3-hydroxy-3-methylglutaroyl)-ester of 6-hydroxyluteolin-7-*O*-*β*-D-glucopyranoside, 8,3′,4′-trihydroxyflavone-7-*O*-*β*-D-glucopyranoside, 3′-hydroxyscutellarein-7-*O*-(6′′-*O*protocatechuoyl)-*β*-glucopyranoside, (−)-4′-methoxy-7-*Oβ*-dglucopyranosyl-8,3′-dihydroxyflavanone, (−)-4′-methoxy-7-*O*-(6′′-acetyl)-*β*dglucopyranosyl-8,3′-dihydroxyflavanone, hesperetin-7-*O*-*β*-D-glucopyranoside. Others: 2′-butoxyethylconiferin, butylconiferin, 2-methoxy-4-(2′-hydroxyethyl)-phenol-1-*O*-*β*-D-glucopyranoside, (1′R,2′R)-guaiacyl glycerol 3′-*O*-*β*-dglucopyranoside, threo-5-hydroxy3,7-dimethoxyphenylpropane-8,9-diol, 3-(4-hydroxy-3-methoxyphenyl)-3-methoxypropane1,2-diol, 3-(4-Hydroxy-3-methoxyphenyl)propane-1,2-diol, guaiacylglycerol, wilfordiol B, caffeoylcalleryanin, 1-*O*-(E)-caffeoyl-*β*-dgentiobiose, dihydrophaseic acid, 1,3,5-trimethoxybenzene, vanillin, galacturonic acid, galactose, glucose, arabinose, xylose, rhamnose.	[238,239,240,241,242]
*Calendula arvensis*		
Antibacterial, anti-fungal, antiparasitic, anti-inflammatory, antioxidant, wound healing, antimutagenic, immunomodulatory, and anti-cancer.	Phenolic acids: Isomeric form hydroxy ferulic acid hexoside, 5-*O*-caffeoylquinic acid, 4-*O*-caffeoylquinic acid, caffeic acid, sinapic acid, sinapic acid hexoside, hexoside derivative, caffeoylshikimic acid, 3,4-*O*-dicaffeoylquinic acid, 5-*O*-feruloyl quinic acid, protocatechuic acid pentoside, quinic acid with an aldonic residue. Flavonoids: Quercetin hydrate, quercetin dihexoside, quercetin-3-*O*-rutinoside, quercetin-3-*O*-neohesperidoside, quercetin-3-omalonylhexoside, quercetin acetyl hexoside, quercetin hexoside I, quercetin 3-*O*-*β*-D-glucopyranoside, quercetin 3-*O*-*β*-D-galactopyranoside, apigenin-8-C-pentose-6-chexose or apigenin-8-chexose-6-C-pentose, apigenin-*O*-hexosylpentosyl, isorhamnetin-3-*O*-hexoside. Saponins: 3-*O*-(*β*-D-galactopyranosyl-(1⟶3)-*β*-D-glucopyranosyl) oleanolic acid-28-*O*- *β*-D-glucopyranoside, 3*β*-*O*-(*β*-D-galactopyranosyl-(1⟶3)-*β*-D-glucopyranosyl) oleanolic acid, 3*β*-*O*-(*β*-D-galactopyranosyl-(1⟶3)-*β*-D-glucopyranosyluronic acid) oleanolic acid-28-*O*- *β*-D-glucopyranoside, 3*β*-*O*-(*β*-D-galactopyranosyl-(1⟶3)-*β*-D-glucopyranosyluronic acid) oleanolic acid, 4-*O*-(*β*-D-fucopyranosyl)-4-alloaromadendrole, arvensoside A, arvensoside B, derivatives of arvensoside B, calenduloside D, calenduloside C, 4-*O*-(*β*-D-fucopyranosyl)-4-alloaromadendrole, 4-*O*-(*β* -D-fucopyranosyl)-4-alloaromadendrol-2″-methylpropanoyl esters, 4-*O*-(*β* -D-fucopyranosyl)-4-alloaromadendrol -2″-methyl-2″-butenoyl esters, Sesquiterpeneglycosides: 3*α*,7*β*-dihydroxy-5*β*,6*β*-epoxyeudesm-4(15)-ene-11-(*O*-*β*-D-fucopyranoside-2′,4′ -diangelate-3′-acetate), 7β-Hydroxy-3*β*-acetoxy-5*β*,6*β*-epoxyeudesm-5(15)-ene-11-(*O*-*β*-D-ficopyranoside-2′,4′-diangelate-3′-acetate), 3α,7*β*-Dihydroxy-5*β*,6*β*-epoxyeudesm-4(15)-ene-11-(*O*-*β*-D-fucopyranoside-2′,4′-diangelate-3′-isobutyrate), 3*α*,7*β*-dihydroxy -5*β*, 6*β*-epoxyeudesm-4(15)-ene-11-(*O*-*β*-D-fucopyranoside-2′, 4′-diangelate-3′-methylbutyrate), and 3*α*,7*β*-dihydroxy-15-acetoxyeudesm-4(5)-ene-11-(*O*-*β*-D-fucopyranoside-2′,4′-diangelate-3′-acetate). Carboxylic acids/Fatty acids: Stearic acid, oleic acid, linoleic acid, linolenic acid, palmitic acid, palmitoleic acid, *α*-linolenic acid, quinic acid, citric acid, and tetracosanoic acid. Polysaccharides: L-threonic acid, D-(−)-tagatofuranose, D-(−)-fructofuranose, D-(−)-fructopyranose, D-(−)-psicopyranose, D-(+)-mannopyranose, D-(+)-galactopyranose, *β*-D-glucopyranose, D-gluconic acid, galactaric acid, sucrose, cellobiose. Others: Ethyl butyrate, 2-methyl-3-furanthiol, methional, 1-octen-3-one, ethyl hexanoate, 2-6-Dimethyl-3 ethyl pyrazine, (E)-2-nonenal, (E,E)-2,4-octadienal, 5-methyl-2-furanaldehyde, citronellol, phenethylacetate, *α*-terpineol, lactone-like, and *δ-*decalactone, Neophytadiene, phytol, *α*-bisabolol, 8,14-cedranoxide, stigmasterol, stigmast-5-ene, amyrin, lup-20(29)-en-28-al, 3-oxo-ursan-28-oic acid, myo-inositol, 1H-benzocyclohepten-9-ol, 1-hexacosanol, untriacontane, 4-aminobutanoic acid, isomer of platynecine derivative, ligstroside hexoside, calendasaponin A, calenduloside G isomer, *β*-sitosterol.	[243]
*Chamaemelum nobile* (syn. *Anthemis nobilis* L. or *Chamomilla nobilis*)		
Anti-inflammatory, antioxidant, antinociceptive, antimutagenic, sedative, anxiolytic, antispasmodic, anxiety, depression, sleep quality and insomnia, postoperative gastrointestinal dysfunction, diarrhoea, colic, nausea, vomiting, acute, diuretic, chronic pain, antibacterial, anti-fungal, insecticidal, hypotensive, antiplatelet aggregation, antioxidant, effect in asthma and polycystic ovary, nervous endocrine, cytotoxic, bronchodilator, antispasmodic, carminative, anti-emetic, antispasmodic, cytostatic, anti-oedema sedative properties	Phenolic acids: The glucose esters caffeic acid, ferulic acid, anthenobilic acid, trans-caffeic acid-glucose ester, trans- and cis- forms of the caffeic acid, 3-*O*-caffeoylquinic acid, 5-*O*-caffeoylquinic acid-hexoside, 3,4-*O*-dicaffeoylquinic acid, protocatechuic acid, caffeoyl-hexoside-methylglutarate, 5-*O*-caffeoylquinic acid, *p*-coumaroyl-hexoside-methylglutarate 1,3,5-*O*-Tricaffeoylquinic acid. Flavonoids: Apigenin, apigenin 6-C-glucose-8-C-glucose, apigenin *O*-glucuronide, apigenin *O*-glucuronylhexoside, luteolin, luteolin *O*-hexoside, luteolin *O*-rutinoside, luteolin *O*-acetylhexoside, luteolin-7-glucoside, luteolin *O*-pentosylhexoside, luteolin *O*-glucuronide, luteolin *O*-rhamnosylhexoside, quercetin, quercetin 3-*O*-glucuronide, quercetin 7-*O*-malonylhexoside, quercetin *O*-acetylhexoside, isorhamnetin *O*-acetylhexoside, myricetin 3-*O*-glucoside, rutin, anthemoside (apigenin2,3-dihydorycinnamoyl acid 7-*O*-*β*-D-glucose), cosmosioside (apigenin 7-*O*-*β*-D-glucose), apiin (apigenin 7-*O*-β-D-apiosylglucoside), chamaemeloside [apigenin 7-*O*-*β*-D-glucose-6″-(3′″-hydroxy-3′″-methyl-glutarate)], luteolin 7-*O*-*β*-D-glucose, quercetin 3-*O*-*α*-L-rhamnoside, kaempferol, kaempferol *O*-pentosylhexoside, catechins. Terpenoids and steroids: *α*-bisabolol, chamazulene, anthesterols, *β*-amyrin, taraxasterol, pseudotaraxasterol, *β*-sitosterol. Coumarins: Herniarin, umbelliferone, scopoletin-7-glucoside. Others: Angelic and tiglic acid esters, anthemic acid, choline, phenolic, phytosterols, inositol, oxalic acid, quinic acid, malic acid, citric acid, fumaric acids, octulosonic acid, betahydroperoxyisonobilin, hydroxyisonobiline, germacranolide-type sesquiterpene lactones (nobilin, 3-epinobilin, 1,10-epoxynobilin, 3-dehydronobilin), amyl and isobutyl alcohols, 1*β*-Hydroperoxyisonobilin, alkyl hydroperoxides, *Cis*- and *trans*-spiroether derivatives, *cis-* and *trans*-dehydromatricariaester and tiophenesetrs, polyacetylenes.	[244,245,246]
*Cichorium intybus*		
The hepatoprotective, anti-inflammatory, antioxidant, sedative, immunomodulatory effect, cardiovascular, hypolipidemic, gastro-protective, anti-tumor, anti-leukaemic, cytotoxic, antimicrobial, allergenic, antibiotic, anti-cancer, anti hyperuricemia, antiprotozoal, anthelmintic, anti-malarial, sedative.	Phenolic acids: Chlorogenic acid, chicoric acid, *p*-coumaric acids, protocatechuic acid, p-hydroxybenzoic, iso vanillic, gallic acid, 4-amino-benzoic, *p*-OH-benzoic, caffeine, ferulic acid, isoferulic acid, vanillic acid, benzoic acid, ellagic acid, alpha-cumaric, 3,4,5-methoxy-cinnamic, salycilic acid, cinnamic acid, 3-*O*-*p*-coumaroyl quinic acid. Flavonoids: Quercetin, quercetin glucuronide, luteolin glucuronide, catechin, catechol, epicatechin, cyanidin-3-*O*-(6″-malonyl-*β*-glucopyranoside), delphinidin 3,5-di-*O*-(6-*O*-malonyl-*β*-d-glucoside), delphinidin 3-*O*-(6-*O*-malonyl-*β*-d-glucoside)-5-*O*-*β-*d-glucoside, delphinidin 3-*O*-*β*-d-glucoside-5-*O*-(6-*O*-malonyl*-β*-d-glucoside), delphinidin 3,5-di-*O*-*β*-d-glucoside. Fatty acids and derivatives: Lauric acid methyl ester, myristic acid methyl ester, palmitoleic methyl ester, palmitic acid methyl ester, methyl dihydromalvalate, 9,12- linoleic methyl ester, stearic acid methyl ester, methyl linolelaidate, linolenic acid methyl ester, 11-eicosenoic acid methyl ester, eicosanoic acid methyl ester, *n-*hexadecanyl hexadecanoate, *n-*pentadecanyl octadec-9-enoate, *n*-hexadecanyl octadec-9-enoate, *n*-hexadecanyl octadecenoate, *n*-octadecanyl octadecenoate, *α*-linolenic acid, oleic acid, linoleic acid, palmitic acid. Sesquiterpene lactones: Lactucin, 8-deoxylactucin, 11(S),13-dihydro-8-deoxylactucin, lactucopicrin, 11(S),13- dihydrolactucopicrin, jacquinelin, crepidiaside B, lactuside A, 11(S), 13-dihydrolactucin, lactucin, 8-deoxylactucin, 11(S), 13-dihydro-8-deoxylactucin, 11(S),13-dihydrolactucopicrin, lactucopicrin Others: Inulin, coumarin, epigallocatechin gallate.	[247,248]
*Dittrichia viscosa* subsp. *Viscosa* (Syn. *Inula viscosa*)		
Antiphlogistic, antiviral, anti-fungal, antibacterial, antiseptic, anti-inflammatory, allelopathic potential, fungicidal, nematicidal, anti-ulcerogenic, antihelmintic, anti-cancer, neuroprotective effects	Phenolic acids and derivatives: Caffeic acid, di-o-caffeoylquinic acid, rosmarinic acid, protocatechuic acid hexoside, caffeoyl hexose, *p*-coumaroyl hexose, 1-O-caffeoylquinic acid, 3-O-caffeoylquinic acid, 4-O-caffeoylquinic acid, di-O-Caffeoylquinic acid, caffeic acid phenethyl ester, (*Epi*)-rosmanol methyl ether, rosmanol, epirosmanol, dicaffeoylshikimic acid, N-caffeoyl-tryptophan, dihydroxybenzoic acid. Flavonoids: Dihydroquercetin, 3-*O*-methylquercetin, quercetin-*O*-(caffeoyl)-hexoside, quercetin dihexoside, quercetin-3-*O*-(6″-acetyl) hexoside, quercetin rhamnoside, cirsiliol, 3-*O*-acetylpadmatin, padmatin, nepetin, spinacetin, diosmetin, rhamnetin, hesperetin, hispidulin, catechin, medioresinol, *γ*-mangostin, banaxanthone E, *epi*- granilin, naringenin, isorhamnetin, diosmetin, cirsimaritin derivative, genkwanin, rutin, kaempferol-*O*-deoxyhexoside, kaempferol-3-*O*-(6″-acetyl) hexoside, kaempferol-3-*O*-(caffeoyl)-hexoside, aromadendrin, naringenin-7-*O*-hexoside, isorhamnetin glycoside, isorhamnetin-*O*-pentosylhexoside, kaempferol-*O*-(*p*-coumaroyl)-hexoside, kaempferol-*O*-(feruloyl)-hexoside, 3,7-dihydroxycoumarin, nepetin, spinacetin, dihydroxycoumarin, padmatin isomer 1/2, cinchonain. Sesquiterpenes: *α*- and *γ*- costic acid isomers, ilicic acid, hydroxyalantolactone, tomentosine/inulviscolide, alantolactone, inulanolide, Others: Galloylquinic acid, (*Epi*)-gallocatechin-3-gallate, paxanthone, proanthocyanidin dimer, prodelphinidin B3, malic acid I and II, caffeoyl-malic acid, shikimoyl blechnic acid.	[249,250,251,252,253,254,255,256,257,258,259]
*Galinsoga parviflora*		
Antibacterial, antioxidant, anti-arthritic, antiplatelet, anti-inflammatory, anti-fungal.	Kaempferol, gallic acid, 2,4,5-tricaffeolylglucaric acid, 2,3,4,5-tetracaffeolylglucaric acid, 2,3,4-tricaffeolylaltraric acid, 3,4,5-tricaffeolylaltraric acid, beta-sitosterol-3-*O*-beta-glucoside, quercetine, beta-sitosterol, 3,5,7,3′,4′pentahydroxyflavanone, 4-hydroxybenzoic acid.	[260]
*Helichrysum stoechas*		
Antibacterial, anti-proliferative, neuroprotective, anti-inflammatory, antioxidant treatment for urolithiasis.	Neo-chlorogenic acid, chlorogenic acid and crypto-chlorogenic acid, isomeric dicaffeoyl quinic acids, isomeric naringenin glucosides, quercetin, isoquercitrin, kaempferol, apigenin glucosides, tetrahydroxychalcone-glucoside, Helipyrone A/B/C, Italipyrone, 20-prenylitalipyrone, Bitalin A (R)-form, 6-methyleuparin, helipyrone, 5,7-dihydroxy-3,6,8-trimethoxyflavone, quercetagetin-7-*O*-glucopyranoside, santinol B.	[261,262,263,264]
*Hypochaeris radicata*		
Treatment of jaundice, rheumatism, and antibacterial, anti-fungal properties with antioxidant and anti-inflammatory, antihemolytic.	Chicoric acid, hypochoeroside C, hypochoeroside D, 5-*O*-caffeoylshikimic acid, 4-(3,4-dihydroxybenzyl)-2-(3,4-dihydroxyphenyl)tetrahydrofuran-3-carboxy-*O*-*β*-D-glucopyranoside, 4-(3,4-dihydroxybenzyl)-2-(3,4-dihydroxyphenyl)tetrahydrofuran-3-carboxy-*O*-β-D-glucopyranosyl-2′-*O*-methacrylate, (7S,8R,8′R)-7-(3,4-dihydroxyphenyl)-3′,4′-dihydroxy-7,8,7′,8′-tetrahydronaphtho [8,8′-c]furan-1(3H)-one, (7S,8R,8′R)-7-(3,4-dihydroxyphenyl)-3′,4′-dihydroxy-8′-(hydroxymethyl)-7,8,7′,8′-tetrahydronaphthalen-8-carboxylic acid, confertin, scopoletin.	[265,266,267,268]
*Lactuca serriola*		
Hepatoprotective, antioxidant, antivenom, allelopathic, sedative, anticonvulsant, antiepileptic, anti-inflammatory, anti-carcinogenic activities	Chlorogenic acid, caffeic acid, quercetin, lactutin, 8-deoxylactucin, jacquilenin, 11-*β*-13-dihydrolactucin, deacetoxymatricarin (=leucodin, leucomisin), loliolide, guaiane ester, the melampolide glucoside, luteolin-7-*O*-*β*-D glucoside, protocatechuic acid, 4-hydroxybenzoic acid, lactuside A, kaempferol, lactucone, lactucic acids, lactucopicrin, sesquiterpene esters, vitamins, oxalic acid, *β*-carotene, iron, lupeol, lupeol acetate, oleanans, *α*-amyrin, *β*-amyrin.	[269,270,271,272,273,274,275,276]
*Onopordum acanthium*		
Antihypertensive, bactericide, cardiotonic, hemostatic agent, used against hypotonicity, anti-inflammatory, anti-malarial, anti-inflammatory, anti-tumor, cytotoxicity, antipyretic, analgesic, anti-tumor, regeneration, phytotoxic.	Phenolic acids and derivatives: Isochlorogenic acid, caffeic acid, Flavonoids: Apigenin, luteolin, scutellarein, nepetin, chrysoeriol, hispidulin, pectolinarigenin, scutellarein 4′-methyl ether, quercetin, apigenin-7-*O*-glucoside, apigenin-7-*O*-rutinoside, apigenin-7-*O*-*β*-D-glucuronide, luteolin-7-*O*-glucoside, quercetin-3-*O*-glucoside, isorhamnetin-3-*O*-glucoside, riodictyol; cyanin, aconiside. Others: Pinoresinol, syringaresinol, medioresinol, nitidanin diisovalerianate; arctiin, aesculin; aesculetin, 4*β*,15-dihydro-3-dehydrozaluzanin C, zaluzanin C, 4*β*,15,11*β*,13-tetrahydrozaluzanin C, onopordopicrin; arctiopicrin, Elemanolide 11(13)-dehydromelitensin *β*-hydroxyisobutyrate; acanthiolide, *α*-amyrin; *β*-amyrin, lupeol; taraxasterol, steroids, heptadecatetraen-(2,8,10,16)-diin-(4, 6)-al-(1), tridecadien-(1,11)-tetrain-(3,5,7,9), heptadecatetraen-(1,7,9,15)-diin-(11,13), heptadecatetraen-(2,8,10,16)-diin-(4,6)-ol-(1)), linoleic acid, oleic acid, palmitic acid, stearic acid, pentadecanoic acid, hentriacontanoic acid, nonacosanoic acid, arachidic acid, margaric acid, myristic acid, behenic acid, palmitoleic acid, gadoleic acid, erucic acid, vaccenic acids, *α*-tocopherol, *α*-tocotrienol, *β*-tocopherol, *γ-*tocopherol, 1-amino-2-propanol, stachydrine, choline, phytin.	[277,278,279,280,281,282,283,284]
*Senecio vulgaris*		
Antioxidant, cytotoxic, antibacterial, anti-fungal	Phenolic acids and derivatives: Caffeic acid, protocatechuic acid, 3-*O*-caffeoylquinic acid (chlorogenic acid), dicaffeoylquinic acid, *p*-hydroxy benzene-acetic acid, vanillic acid, syringic acid, *p*-hydroxy benzene-acetic acid derivative, *p*-hydroxycinnamic acid. Flavonoids: Quercitin-3-glucoside (Isoquercitrin), quercetin 3-*O*-rhamnoside (quercitrin), kaempferol-3-*O*-di-deoxyhexoside. Pyrrolizidine alkaloid: Retrorsine N-oxide, spartioidine N-oxide, seneciophylline N-oxide, integerrimine N-oxide, senecionine N-oxide, usaramine, neosenkirkine, riddelline, *neo*platyphylline, retrorsine, spartioidine, platyphylline, integerrimine, senecionine.	[285,286,287,288]
*Solidago virgaurea*		
Antioxidant, anti-inflammatory, analgesic, spasmolytic, antihypertensive, diuretic effects and benefits in other urinary tract conditions, antibacterial, anti-fungal, antiparasitic, cytotoxic and anti-tumor, antimutagenic, cardioprotective, antisenescence effects.	Phenolic acids and derivatives: Caffeic acid, chlorogenic acid, 5-*O*-caffeoylquinic (neo chlorogenic) acid, 3,5-di-*O*-caffeoylquinic acid, 3,4-di-*O*-caffeoylquinic acid, 4,5-di-*O*-caffeoylquinic acid, 3,4,5-tri-O-caffeoylquinic acid, methyl 3,5-di-*O*-caffeoylquinate, 3-hydroxyphenyl acetic acid, 3,4-dihydroxyphenylacetic acid, 5-p-coumaroylquinic acid, homovanilic acid, *p*-coumaric acid, ferulic acid, sinapic acid, rosmarinic acid benzoic acid, 3-hydroxybenzoic acid, 4-hydroxybenzoic acid, 3,4-dihydroxybenzoic (protocatechuic) acid, salicylic acid, gentisic acid, vanillic acid, gallic acid, leiocarposide, 2-methoxybenzyl-2,6-dimethoxy benzoate. Flavonoids: Quercetin, quercetin-3-*O*-glucoside (isoquercitrin), quercetin-3-*O*-galactoside (hyperoside), quercetin-3-*O*-rhamnoside (quercitrin), quercetin-3-*O*-rutinoside (rutin), quercetin-3-O-arabinopyranoside (avicularin), kaempferol-3-*O*-glucoside (astragalin), kaempferol-3-O-rhamnoside (afzelin), kaempferol-3-*O*-rutinoside (nicotiflorin), kaempferol-3-*O*-robinobioside (biorobin), myricetin 3-rhamnoside (myricitrin), Isorhamnetin-3-*O*-rutinoside (narcissin), cyanidin-3-gentiobioside mono-C-glycosylflavones, di-C-glycosyl flavones. Others: Virgaureasaponins 1–6, solidagosaponins X-XXIX, bellisaponin BA2, erythrodiol-3-acetate, *α*-tocopherol quinone, 2-phyten-1-ol.	[289]
*Sonchus asper*		
Antioxidant, anti-inflammatory, antibacterial, insecticidal, hepatorenal protective, used for treating bronchitis, gastrointestinal infection, cardiac dysfunction, kidney diseases, and cancer.	Phenolic acids and derivatives: Caffeic acid, 3-coumaric acid, chlorogenic acid, gallic acid, luteolin, luteolin-7-o, protocatechuic acid, rosemarinic acid, quinic acid, vanillic acid. Flavonoids: Apigenin, apigenin-7-o, luteolin, pyrocatechol, quercetin, rutin. Others: 11 beta,13-dihydrourospermal A, 15-*O*-beta-D-glucopyranosyl-11 beta,13-dihydrourospermal A, 15-*O*-beta-D-glucopyranosylurospermal A, 15-*O*-[6′-(p-hydroxyphenylacetyl)]-beta-D-glucopyranosylurospermal A and 14-*O*-methylacetal-15-*O*-[6′-(*p*-hydroxyphenylacetyl)]-beta-D-glucopyranosylurospermal A, asperal, emodin, methyl-(3,8-di-hydroxy-6-methyl-9-oxo-9H-xanthene)-1-carboxylate.	[118,290,291,292,293,294,295,296,297,298,299,300]
*Sonchus oleraceus*		
Antioxidant, anti-inflammatory, anti-tumor, antibacterial, anti-fungal, antidepressant, anxiolytic, antinociceptive effects, used for treating cancer, diarrhoea, and enteritis.	Phenolic acids and derivatives: Chicorin, caffeic acid glycoside, 4-cafffeoylquinic acid, 5-caffeoylquinic acid, *cis*-3′ caffeoylquinic acid, 5-coumaroylquinic acid, caftaric acid, chicoric acid, 3,4 dicaffeoylquinic acid, 3,5 dicaffeoylquinic acid, dicaffeoylquinic acid (isomer), *cis*-3,5 dicaffeoylquinic acid (isomer), tri-O-caffeyolyquinic acid, *cis*-3,4 dicaffeoylquinic acid, 4,5 dicaffeoylquinic acid. Flavonoids: Quercetin-glucoronide-glycosyl, quercetin-hexose-hexoside, quercetin glucoside glucoronide, luteolin-glycosyl-glucuronide, luteolin-diglucoside, isorhamnetin diglucoside, luteolin, luteolin glucuronide, luteolin glycoside, quercetin-rutinoside, isorhamnetin rutinoside, luteolin, quercetin acetylglycoside, apigenin glucuronide, apigenin rutinoside, kaempferol acetylglycoside sesquiterpenes, crepidiaside A. Others: 7S,10S-3,9-dioxo-di-nor-eudesma-4-en-11-oic acid, 6 R,7S,10S-3,9-dioxo-7-hydroxyldi-nor-eudesma-4-en-11-oic acid.	[301,302,303,304,305,306]
*Tanacetum parthenium*		
Antioxidant, anxiolytic, antidepressant, anti-migraine agent, anticoagulant, anti-inflammatory, neuroprotective, antiviral, anti-apoptotic, anti-cancer, antiparasitic, pain reliever.	Phenolic acids and derivatives: 4-o-caffeoyl-quinic acid, 3,4-dicaffeoyl-quinic acid, 3,5-dicaffeoyl-quinic acid, 4,5-dicaffeoyl-quinic acid, neochlorogenic acid, ellagic acid, chlorogenic acid. Flavonoids: Kaempferol-3-rutinoside, 6-hydroxykaempferol-3,6,4′-trimethylether (santin), 6-hydroxykaempferol-3,6-dimethylether, quercetagenin-3,6-dimethylether (axillarin), quercetagenin-3,6,3′-trimethylether (jaceidin), quercetagenin-3,6,4′-trimethylether (centaureidin), apigenin, luteolin, santin, chrysoeriol, luteolin-7-glucoronides, methylquercetin, trihydroxy-methoxyflavone, costunolide, dihydro-*β*-cyclopyrethrosin, sudachitin, aceronin, tanacetol A isomer, nevadensin, parthenolide, casticin, nevadensin, tanaphillin, 3-*β*-hydroxyanhydroverlotorin, seco-tanapartholide A/B, hispidulin.	[307,308,309,310,311,312,313,314,315,316,317]
*Tanacetum vulgare*		
Antioxidant, anti-inflammatory, anti-tumor, antibacterial, antiparasitic, anthelmintic, repellent, insecticidal, antiviral, anti-fungal.	Phenolic acids and derivatives: Caffeoylgluconic acid, 1-caffeoylquinic acid, protocatechuic acid, *p-*hydroxyphenylacetic acid 1-*O*-hexoside, protocatechuic acid-*O*-hexoside isomer, syringic acid 4-*O*-hexoside, neochlorogenic (3-caffeoylquinic) acid, *O*-caffeoyl hexose, vanillic acid 4-*O*-hexoside, vanillic acid, caffeoylgluconic acid isomer, *O*-caffeoyl hexose isómer, 4-hydroxybenzoic acid, 4-hydroxybenzoic acid-hexoside, 3-*p-*coumaroylquinic acid, caffeoylgluconic acid isomer, *O*-caffeoyl hexose isomer, quinic acid, chlorogenic (5-caffeoylquinic) acid, *p*-coumaric acid, 3-feruloylquinic acid, caffeic acid-*O*-hexoside, caffeic acid, gentisic acid, 5-*p*-coumaroylquinic acid, 3-caffeoyl-5-hydroxy-dihydrocaffeoylquinic acid, *p-*hydroxyphenylacetic acid, 5-feruloylquinic acid, 1-caffeoyl-3-hydroxy-dihydrocaffeoylquinic acid, vanillic acid-4-*O*-(6-*O*-caffeoyl)-hexoside, 3,4-dicaffeoylquinic acid, 3,5-dicaffeoylquinic acid, 3-dehydrocaffeoyl-5-caffeoylquinic acid, 4,5-dicaffeoylquinic acid, shikimic acid, 4-dehydrocaffeoyl-5-caffeoylquinic acid, salicylic acid, 3-feruloyl-4-caffeoylquinic acid, 3-*p*-coumaroyl-5-caffeoylquinic acid, caffeic acid-*O*-(salicyl)-hexoside, 3-caffeoyl-5-*p*-coumaroylquinic acid, 3-feruloyl-5-caffeoylquinic acid, 4-caffeoyl-5-*p*-coumaroylquinic acid, 4-caffeoyl-5-feruloylquinic acid, 3,4,5-tricaffeoylquinic acid. Flavonoids: Apigenin, apigenin-6,8-diC-hexoside, apigenin 7-*O*-glucoside, methoxyeriodictyol-*O*-hexuronide, apigenin-*O*-hexuronide, luteolin, luteolin-*O*-hexuronide, luteolin 7-*O*-glucoside, 6-hydroxyluteolin-*O*-hexoside, luteolin 7-*O*-gentiobioside dihexoside (gentiobioside) 6-glucopyranosyl-glucopyranoside, luteolin-7-*O*-neohesperidoside, luteolin-O-caffeoylhexoside, luteolin-*O*-acetylhexosidekaempferol 3-*O*-glucuronide, rutin, quercetin, quercetin 3-O-acetylhexoside, quercetin 7-*O*-hexuronide, kaempferol, kaempferol 3-*O*-glucoside, eriodictyol-*O*-hexuronide, patuletin-*O*-hexoside, nepetin-*O*-hexoside, isorhamnetin 3-*O*-glucoside, naringenin-*O*-hexuronide, hesperetin 7-*O*-rutinoside (hesperidin), nepetin-O-hexuronide, hispidulin-*O*-hexuronide, isorhamnetin-*O*-hexuronide, chrysoeriol-*O*-hexuronide, hesperetin-*O*-hexuronide, jaceosidin -*O*-hexuronide, patuletin (6-methoxyquercetin), nepetin (6-methoxyluteolin) 6-methoxykaempferol, naringenin, hispidulin (scutellarein-6-methyl ether), chrysoeriol, hesperetin, Isorhamnetin, jaceosidin (6-hydroxyluteolin-6,3′-dimethyl ether), quercetagetin-3,6,3′(4′)-trimethyl ether, cirsimaritin (6-hydroxyapigenin-6,7-dimethyl ether), eupatilin, casticin, acacetin. Sesquiterpene lactones and derivatives: *α*/*β* thujone, hydroxyarbusculin, ludovicin C, tanacetin/hydroxyraynosin/armefolin, parthenolide, camphor, caryophyllene oxide, dehydrosantamarin, caryophyllene/bisabolene, linoleamide, palmitamide, oleamide.	[315,318,319,320,321,322,323,324,325,326,327]
***Lamiaceae***		
*Calamintha nepeta* subsp. *nepeta* (Syn. *Clinopodium nepeta*)		
Stimulant, tonic, antiseptic, antispasmodic, antioxidant, antimicrobial, anti-inflammatory, anti-ulcer, phytotoxic.	Phenolic acids and derivatives: 3-*O*-Caffeoylquinic acid, 4-*O*-caffeoylquinic acid, 5-*O*-caffeoylquinic acid, rosmarinic acid, quercetin-3-*O*-rutinoside, gallic acid, protocatechuic acid, chlorogenic acid, *p*-hydroxybenzoic acid, vanillic acid, syringic acid, vanillin, *trans-*cinnamic acid, coumarin, quinic acid, 12-*O*-hexosyljasmonate, caffeic acid hexamer, caffeic acid pentamer, rosmarinic acid, 12-*O*-(6′-caffeoylhexosyl)jasmonate, acacetin 7-*O*-[hexosyl(1iv → 2″)]deoxyhexosyl(1′″ → 6″) hexoside. Flavonoids: Myricetin, quercetin, luteolin, hesperidin, kaempferol, kaempferol-di-*O*-hexoside, apigenin, luteolin-8-C-(3-hydroxy-3-methyl-glutaroyl) hexosyl hexoside, 6,8-C-dihexosylapigenin, caffeic acid dimer, quercetin-3-*O*-hexoside, quercetin-3-*O*-[6″-*O*-(3-hydroxy-3-methyl-glutaroyl)]hexoside, kaempferol-3-*O*-hexoside, salvianolic acid B, acacetin, acacetin 7-*O*-[6iv-*O*-acetyl-hexosyl(1iv → 2″)]deoxyhexosyl(1′″ → 6″)hexoside, acacetin 7-*O*-deoxyhexosyl(1′″ → 6″)hexoside.	[328,329,330,331,332,333,334,335,336]
*Lavandula pedunculata*		
Anti-inflammatory, antioxidant, antimicrobial.	Phenolic acids and derivatives: Salvianolic acid B, rosmarinic acid, caffeic acid, caffeic acid hexoside, *p*-coumaroyl hexoside, rosmarinic acid, rosmarinic acid hexoside, sangerinic acid, lithospermic acid A, chlorogenic acid, 3-hydroxy-4-methoxybenzaldehyde thiosemicarbazone, ferulic acid, syringic acid, vanilic acid, *p-*hydroxybenzoic acid, protocatechuic acid, gallic acid. Flavonoids: Luteolin-*O*-hexosyl-*O*-glucuronide, eriodictyol-*O*-glucuronide, luteolin-7-*O*-glucuronide, methylluteolin-*O*-glucuronide, eriodictyol-*O*-glucuronide, herniarin, myricetin.	[337,338,339,340]
*Lavandula stoechas*		
Anti-inflammatory, antioxidant, antispasmodic, sedative, antibacterial, anti-fungal, insecticidal, larvicidal, hepatoprotective, renoprotective, anti-leishmaniasis.	Phenolic acids and derivatives: Protocatechuic acid, chlorogenic acid, caffeic acid, rosmarinic acid, ferulic acid, 7-methoxy coumarin. Flavonoids: Flavone di-*O*-glycosides, flavone 7-*O*-monoglycosides, pinobanksin, quercetin, pinocembrin, luteolin, vitexin, acacetin, erythrodiol. Others: Ursolic acid, vergatic acid, oleanolic acid, *α*-amyrin, *α*-amyrin acetate, *β*-sitosterol, lupeol, two longipinane derivatives (longipin-2-ene-7*β*,9*α*-diol-1-one and longipin-2-ene-7*β*,9*α*-diol-1-one-9-monoacetate), lavanol.	[341,342,343,344,345,346,347]
*Melissa officinalis*		
Anti-proliferative, anti-tumor, antioxidant, antiangiogenic, cardioprotective, anxiolytic antidepressant, antinociceptive, neuroprotective, GABA-T inhibitor, anti-kinetoplastidae, analgesic, hypnotic, anti-Alzheimer, antispasmodic, antiviral, anti-fungal, antibacterial, for premenstrual syndromes.	Phenolic acids: Caffeic acid, caftaric acid, chlorogenic acid, ferulic acid, gentisic acid*, p-*Coumaric acid, rosmarinic acid. Flavonoids: Apigenin, cynaroside, daidzein, hyperoside, isoquercetin, kaempherol, luteolin, myricetin, quercetin, quercetrol, rutin. Triterpenes: Betulinic acid, oleanolic acid, ursolic acid, 23-sulfate ester of niga-ichigoside F1, 3*β*,16*β,*23-trihydroxy-13,28-epoxyurs-11-ene-3-*O*-*β*-D-glucopyranoside, 3,23-Disulfate ester of 2*α*,3*β*,19*α,*23-tetrahydroxyurs-12-en-28-oicacid, 3,23-Disulfate ester of 2*α*,3*β*,19*α*,23-tetrahydroxyurs-12-en-28-oicacid 28-*O*-*β*-D-glucopyranoside, 3,23-Disulfate ester of2*α*,3*β*,23,29-tetrahydroxyolean-12-en-28-oicacid, 3,23-disulfate ester of 3*β*-23,29-trihydroxyolean-12-en-28-oic acid, 3,23-disulfate ester of 2*α*,3*β*-23,29-tetrahydroxyolean-12-ene-28-oicacid, 23-sulfate ester of 2*α*,3*β,*19 *α*,23-tetrahydroxyurs-12-en-28-oic acid, melissioside A, melissioside B, melissioside C.	[348,349,350,351]
*Mentha aquatica*		
Antioxidant, anxiolytic, anti-inflammatory, hepatoprotective, antimicrobial, anti-cancer.	Phenolic acids: Rosmarinic acid, caffeic acid. Flavonoids: Luteolin-7-*O*-rutinoid, Eriodictyol-*O*-rutinoside, naringenin-7-*O*-rutinoside, hesperetin-7-*O*- rutinoside, luteolin glucoside, luteolin-7-O-*β*-D-diglucuronide, eriocitrin, apigenin-7-*O*-*β*-D-diglucuronide, luteolin-7-*O*-glucuronide, narirutin, apigenin-7-*O*-rutinoside, apigenin-7-*O*-glucuronide, hesperidin, catechin. Others: methyl ester palmitic acid, methyl ester linolenic acid, ethyl ester linolenic acid, neophytadiene, phytol, viridiflorol, rotundifolone, 2,3-*seco*-triterpene, 3-*O*-benzoyltormentic acid, tormentic acid, 1-*O*-benzoylhyptad, ienic acid, 3-epiursolic acid, hyptadienic acid, 3-*epi*-maslinic acid, 3-*epi*-tormentic acid, ursolic acid, *β*-sitosterol, oleanolic acid, pomolic acid, micromeric acid, 21*α*-hydroxyursolic acid, pomolic acid, hyptadienic acid, 1-*O*-linoleoyl-2-*O*-enadecanoyl-3-*O*-palmitoleoyl-*sn*-glycerol, 1-*O*-oleoyl-2-*O*-enadecanoyl-3-*O*-palmitoleoyl-*sn*-glycerol, 1, 3-*O*-dioleoyl-2-*O*-eicosanoyl-*sn*-glycerol, 1-*O*-linoleoyl-2-*O*-palmitoleoyl-*sn*-glycerol, corosolic acid, asiatic acid, choline, acetic acid, formic acid, lactic acid, quinic acid, salicylic acid, succinic acid, fructose, glucose, sucrose, alanine, aspartic acid, glycine, isoleucine, leucine, phenylalanine, threonine, valine.	[352,353,354,355,356,357,358,359,360,361,362,363,364,365,366,367,368,369,370,371]
*Mentha pulegium*		
Insecticidal, nematicidal, allelopathic, antioxidant, antimicrobial, antiviral, antileishmanial, anti-tumor, anti-cancer, anti-hemolytic, antihypertensive, anti-inflammatory, burn wound healing, cardioprotective, stomachic, astringent, emmenagogue, decongestant, antispasmodic, antiseptic, depurative, digestive, anti-rheumatic, anti-arthritic, hepatotoxicity.	Phenolic acids: Gallic acid, chlorogenic acid, caffeic acid, ellagic acid, fumaric acid, protocatechuic acid, *p*-hydroxybenzoic acid, syringic acid, cinnamic acid, vanillic acid, ferulic acids, *p*-coumaric acid, chlorogenic acid, rosmarinic acid. Flavonoids: Epicatechin, catechin, apigenin, salvigenin, salvigenin, luteolin, isorhamnetin, quercetagetin-3,6-dimethylether, kaempferol, kaempferol-3-*O*-rutinoside, hesperidin, thymonin, jaceosidin, pectolinaringenin, ladanein, sorbifolin, pedalitin, diosmin, luteolin, apigenin, naringenin, chrysin, chrysoeriol, vicenin-2, gallocatechin isomer 1. Others: Alterporriol, atropisomer, altersolanol, stemphypyrone, 6-*O*-methylalater-nin, macrosporin, salvianolic acid, Lithospermic acid, jaceidinisomer 1, Jaceosidin.	[371,372,373,374,375,376,377,378,379]
*Mentha suaveolens*		
Antioxidant, antimicrobial, antimutagenic, analgesic, anti-inflammatory, insecticidal, anti-cancer, antithermal skin-aging effect.	Phenolic acids: Cinnamic acid, chlorogenic acid, rosmarinic acid, caffeic acid, *p*-methyl coumarate, ferulic acid, *p*-coumaric acid, gallic acid, hydroxybenzoic acid, hydroxybenzoic acid, 3-dihydroxybenzoic acid, vanillic acid, salicylic acid, salicylic acid 2-*O*-*β-*glucoside, *trans-*cinnamic acid, *p*-methyl coumarate, *p*-anisic acid. Flavonoids: Hesperidin, rutin, quercetin, naringenin, luteolin, kaempferol, apigenin.	[352,376,380,381,382,383,384,385,386,387]
*Origanum vulgare* L.		
Antibacterial, anti-fungal, antiviral, antiparasitic, antioxidant, anti-inflammatory, anti-tumoral, beneficial activity on skin disorders, effects on melanin production and on human sperm mobility, anti-Alzheimer, energy producer, stomach booster, nervous system reliever, laxative, reducing the general weakness of the body, anti-cancer, relief of migraine pain, for external use by rubbing in place of fractures and numbness of body parts, toothache, disinfection, antidiarrhoea, anticonvulsant, expectorant, nourishing, menstrual regulator, anti-urinary tract infection, treatment of sexual dysfunction, colic, sinusitis, relaxing, cardiorespiratory booster, nervous system booster, treatment of blockages, hepatoprotective.	Phenolic acids: Rosmarinic acid, chlorogenic acid, cinnamic acid, caffeic acid, syringic acid, benzoic acid, vanillic acid, *galo*-coumaric acid, gallic acid, protocatechuic acid, 4-hydroxybenzoic acid, *p-*coumaric acid, ferulic acid, sinapic acid, *trans*-cinnamic acid, 2,4-dihydroxybenzoic acid, phenyllactic acid. Flavonoids: Quercetin, apigenin, kaempferol, naringenin, eriodictyol, salvianolic acid B, llithospermic acid B, amburosides A, luteolin, luteolin 7-O-glucuronide, apigenin 7-O-glucuronide, (−)-epigallocatechin, (+)-catechin, rutin. Others: Thymoquinone, thymol, carvacrol, demethylbenzolignanoid, chicoric acid, calleryanin 3,4-dihydroxybenzoate, calleryanin 3-hydroxy,4-methoxybenzoate, gastrodin 3,4-dihydroxybenzoate.	[388,389,390,391,392,393,394,395,396]
*Prunella vulgaris*		
Anti-tumor, anti-inflammation, immunoregulation, antiviral, antioxidant, anti-osteoporosis, antidepression, hypotensive, hypolipemic, cardioprotective, anti-dementia, anti-amnesia.	Phenolic acids: *p-*coumaric acid, caffeic acid, rosmarinic acid. Flavonoids: Kaempferol, luteolin, delphinidin, quercetin, quercetin-3-*O*-*β*-D-galactoside, homoorinet, cinaroside, quercetin-3-*O*-*β*-D-glucoside, kaempferol-3-*O*-*β*-D-glucoside. Steroids and derivatives: Beta-sitosterol, spinasterol, stigmasterol, vulgaxanthin-I, poriferasterol monoglucoside, morin, ducosterol, (22E20S24S)-stigmast-7,22-diene-3-e, Stigmast-7-en-3*β*-ol. Triterpenes: Oleanolic acid, ursolic acid, vulgarsaponin A/B, methyl oleanolate, methyl ursolate, methyl, maslinate, pravuloside A/B, palmitic acid, ethyl palmitate, tetracosanoic acid, stearic acid, 6,9-octodecadienoic acid, 3,6,7-eicosatrienoic acid, oleic acid, peanut oleic acid, moringoic acid, lauric acid, myristic acid, linolenic acid, palmitic acid, myristic acid, linoleic acid. Coumarins: Umbelliferon, scopoletin, esculetin.	[397]
*Salvia verbenaca*		
Antibacterial, antioxidant, anti-cancer, antiparasitic, insecticidal, antihemolytic.	Phenolic acids: *p*-Hydroxybenzoic acid, *p*-coumaric acid, rosmarinic acid, vanillic acid, caffeic acid, ferulic acid, 3-*O*- and 4-*O*-caffeoylquinic acids. Flavonoids: Naringenin, cirsiliol, luteolin, apigenin, naringin, hesperidin, genkwanin. Others: Palmitic acid, stearic acid, linolenic acid, arachidic acid, oleic acid, linoleic acid, palmitoleic acid, arachidic acid, verbenacines, salvinines, 6,7-dehydroroyleanone, cryptanol, sitosterol, campesterol, 6-hydroxysalvonolone, microstegiol, stigmasterol, carnosic acids, methyl carnosate contents, carnosol.	[387,398,399,400]
*Thymus mastichina*		
Antibacterial, anti-fungal, antioxidant, anti-cancer, antiviral, insecticidal, insect repellent, anti-Alzheimer, anti-inflammatory.	Phenolic acids: Rosmarinic acid, hydroxycinnamoylquinic acid, 3-methoxysalicylic acid, caffeic acid, chlorogenic acid, salvianolic acid B/E, salvianolic acid A/K isomer. Flavonoids: Quercetin glucoside, 6-hydroxyluteolin-7-*O*-glucopyranoside, luteolin glucoside, 6- hydroxyapigenin7-*O*glucopyranoside, apigenin-7-*O*glucoside, naringenin, luteolin, carnosol, apigenin, kaempferol, chrysoeriol-*O*-hexuronide, sakuranetin, sterubin. Others: Oleanolic acid, ursolic acid, xanthophyll lutein, *β*-sitosterol.	[401]

- *Achillea millefolium* L.

The *Achillea* genus is well known for its use in preventing diabetes (Table 2). Most of the research has been carried out on *Achillea millefolium* L. Yarrow, a perennial plant native to the temperate regions of Europe and Asia. Humans have used it for over 3000 years [213]. It is commonly known as yarrow (milefólio or erva-carpinteira) and is widespread in mountain meadows, pathways, crop fields, and home gardens [127]. In the study by Rezaei et al. [402], the effect of a hydroalcoholic extract of this plant at 25 mg/kg/day and 100 mg/kg/day was evaluated on streptozotocin (STZ)-induced diabetic rats. The results showed that this extract had a beneficial effect on serum glucose, lipids, and liver enzymes compared with the metformin-treated groups and controls. These effects were also more pronounced at 100 mg/kg/day than at 25 mg/kg/day [402]. According to Rezaei et al. [402], STZ caused a considerable increase in serum liver enzyme levels, while treatment with metformin or *A. millefolium* extract significantly attenuated these elevations. 

Similar results were reported in a study by Coskun et al. [403] on the protective effect of Achillea on abnormal lipid profiles. Mustafa et al. (2012) evaluated the hypoglycaemic and hypolipidemic effects of the extract of *A. millefolium* in ALO-induced diabetic rats [404]. They reported that the extracts at dose levels of 250 and 500 mg/kg body weight (BW) showed a significant decrease in blood glucose level, TG (Triglycerides), VLDL (Very Low-Density Lipoprotein), cholesterol, SGOT (Serum Glutamic-Oxaloacetic Transaminase Test), SGPT (Serum Glutamic Pyruvic Transaminase test), and ALP (Alkaline Phosphatase) in diabetic rats. Nematy et al. (2017) reported that the plant had dose-dependent positive effects on appetite in rats [212]. According to Rezaei et al. (2020), the flavonoids present in *A. millefolium* can block serotonin receptors and increase plasma ghrelin content [402], as well as appetite [212,405]. Therefore, this plant has antioxidant properties and can be used to improve the complications of oxidative stress conditions such as T2DM [211]. This extract could act as a hypoglycaemic factor and reduce intestinal glucose absorption thanks to its pharmacological properties (Table 1). According to Karimi et al. (2021), treatment with *A. millefolium* could protect renal tissue against the complications of diabetes by increasing Bax (Bcl-2-associated X protein) mRNA levels. This study indicates that hydroalcoholic extracts of the plant not only improve renal function through their antioxidant activity but modulate certain biochemical factors in diabetic rats [406]. 

Another study by Chávez-Silva et al. (2018) suggests that the hydroethanolic extract of *A. millefolium* probably induces its antidiabetic function via the PPAR*γ* (activating peroxisome proliferator-activated receptors)/GLUT4 pathway, improving sensitivity to insulin and promoting the expression of glucose metabolism genes, such as GLUT4, which allow glucose to be transported into the cell, resulting in its reduction in the blood [407]. On the other hand, Zolghadri et al. (2014) reported that the ethanolic extract of *A. millefolium* significantly decreased the expression of IL-1*β* and iNOS (inducible Nitric Oxyde Synthase) genes against the cytotoxic effect induced by STZ on pancreatic *β* cells, and those increasing insulinemia [408]. Furthermore, as a result, it was persistent throughout the experiments in the oral glucose tolerance test and the STZ diabetic model; this suggests another mode of function that participates as an extrapancreatic contribution, which could induce insulin sensitisation [407]. According to Chávez-Silva et al. (2018), these results could be related to PPAR*γ* activation, as there is evidence that this decreases inflammatory cytokines [IL-6, TNF-α (Tumour Necrosis Factor), IL-1*β*, IL-10, IL-12, and gelatinase B]. It decreases iNOs and scavenger receptor A gene expression [407].

**Table 2:** Medicinal plants in NPSE for diabetes management with scientific validation of the claimed antidiabetic effects (in vitro).

Target	Part Used/Extraction	Observations	References
*Asteraceae*			
*Arctium minus* (Hill) Bernh			
A-GLU/A-AMY	1 mg/mL of MeOH, CH_2_Cl_2_, EtOAc, and BuOH extracts of leaves (L), flowers (F) and roots (R).	AGLU-LMeOHext = 3.32 ± 9.80, AMY-LMeOHext = 12.65 ± 6.40. AGLU-LCH2Cl2-ext = 87.12 ± 8.06, AMY-LCH2Cl2-ext = 28.84 ± 5.57. AGLU-LEtOAc-ext = na, AMY-LEtOAc-ext = na. AGLU- LBuOH-ext = 24.49 ± 15.92, AMY-LBuOH-ext = 30.50 ± 8.35. AGLU-LAqua-ext = 15.51 ± 6.96, AMY-LAqua-ext = 5.74 ± 5.95. AGLU-FMeOHext = na, AMY-FMeOHext = na. AGLU-FCH2Cl2-ext = 21.68 ± 3.12, AMY-FCH2Cl2-ext = na. AGLU-FEtOAc-ext = 40.69 ± 6.90, AMY-FEtOAc-ext = na. AGLU-FBuOH-ext = 6.40 ± 4.45, AMY- FBuOH-ext = na. AGLU-FAqua-ext = 13.32 ± 2.22, AMY-FAqua-ext = na. AGLU-RMeOHext = na, AMY-RMeOHext = na. AGLU-RCH2Cl2-ext = 68.01 ± 7.02, AMY-RCH2Cl2-ext = na. AGLU-REtOAc-ext = 36.11 ± 10.68, AMY-REtOAc-ext = na. AGLU- FBuOH-ext = na, AMY- FBuOH-ext = na. AGLU-RAqua-ext = 30.40 ± 8.50, AMY-RAqua-ext = na.	[173]
*Achillea millefolium*			
A-GLU	Hydromethanolic extract of aerial parts.	AI 55% inhibition at 1.6 mg/mL.	[409]
A-GLU	Hydroethanolic extract of aerial parts.	The extract promoted the α-glucosidases inhibition by 55% at 1 mg/mL concerning control. It increased the PPARγ (five times) and GLUT4 (two-fold) relative expression than the control (*p* < 0.05). Finally, it significantly increased INS secretion and [Ca2+]i compared with the control.	[407]
INS secretion and calcium mobilization			
PPARγ and GLUT4 expression analysis.			
*Arnica montana*			
A-AMY	Methanolic extract fractions (dried cell biomass of seeds germinated).	All fractions inhibited α-amylase activity (almost 12%).	[410]
*Bellis perennis*			
Quantification of GLUT4 translocation.	- A mixture of flowers and leaves (EXT4404) ethanolic extracts. - Ethanolic extract of flowers alone (EXT4407). - Ethanolic extract was prepared from flowers collected from a local area.	Both extracts had a clear dose-response relationship, with EXT4404 being slightly more effective than EXT4407. However, EXT4407 had no effect at 0.25 mg/L, while EXT4404 at the same concentration only increased by about 4%. Overall, all the extracts are effective inducers of GLUT4 translocation without INS.	[411]
Glucose Transport Assay			
A-GLU/A-AMY	Methanol: water (80:20%, *v*/*v*) extract of flowers.	IC50A-AMY: 8.48 ± 0.07 mg/mL of dried flowers; IC50A-GLU: 49.62 ± 0.01 mg/mL of dried flowers.	[412]
*Bidens frondose*			
A-GLU/A-AMY	Ethanolic extracts (80%) of aerial parts.	IC50A-GLU = 0.41 mg/mL, the extracts inhibited α-glucosidase enzyme strongly (64.29–75.22% at 2 mg/mL); inactive on α-amylase activity.	[413]
*Cichorium intybus*			
A-AMY	Aqueous extracts of aerial parts.	IC50A-AMY = 136.13 ± 8.09 µg/mL,	
Insulinotropic investigations (IC1)	Caffeic, ferulic acids, and Chicoric acid (CAE, extracted from aqueous extract).	Caffeic acid mainly promotes a decrease in hepatic glycogenolysis. Ferulic acid elicits a clear increase in INS release and a reduction in hepatic glycogenolysis. CAE increases INS release and glucose uptake without affecting hepatic glycogenolysis. None of these compounds implicates hepatic glucose 6-phosphatase in contrast to chlorogenic acid, an inhibitor of glucose 6-phosphatase.	[414]
Insulin sensitizing investigations (IC2)			
Hepatocyte culture and glycogenolysis test (IC3)			
Evaluation of glucose 6-phosphatase activity (IC4)			
Glucose uptake assay.	Caffeic acid, chlorogenic acid (CGA), and chicoric acid (CAE).	CRA and CGA increased glucose uptake in L6 muscular cells, an effect only observed in the presence of stimulating concentrations of INS. Both CRA and CGA stimulated INS secretion from the INS-1E cells and rat islets of Langerhans. The effect of CRA is only observed in the presence of subnormal glucose levels.	[415]
β-cell culture and measurement of INS secretion.			
Rat pancreatic islet experiments.			
Study of G6Pase and PEPCK expression (IC5).	Three di-O-caffeoylquinic acids (CQA) were extracted from chicory roots methanolic extract.	CQA suppressed hepatic glucose production in H4IIE rat hepatoma cells by reducing the expression of G6Pase and PEPCK. Activation of PI3K and MAPK pathways as a method of controlling gene expression. Promoted increased mitochondrial respiration and cellular metabolism by inducing oxidative phosphorylation and proton leak.	[416]
Gene expression of PI3K and MAPK pathways			
Cellular bioenergetics (IC6).			
Differentiation induction of embryonal carcinoma stem cells into INS-producing cells (IC7)	Methanolic extracts (100%) of leaves.	The extract efficiently induced the differentiation of P19 EC cells into clusters similar to pancreatic islets with the molecular, cellular, and functional characteristics of mature β cells.	[417]
A-GLU/A-AMY	Aqueous methanolic extracts (80% methanol, 19% H2O, 1% HCl; *v*/*v*/*v*) of the plant.	IC50A-AMY: 18.3 ± 0.7 mg/mL; IC50A-GLU: 4.25 ± 0.08 mg/mL	[418]
Glucose uptake test.	- Natural chicoric acid extract (NCRAE): Hydroethanolic extract (70:30). - Synthetic Chicoric and Chlorogenic Acids Mixture (SCCAM) contains the two major compounds of NCRAE, in proportion to 70% of synthetic L-chicoric acid (CRA) and 30% of synthetic chlorogenic acid (CGA).	Adding NCRAE increased glucose uptake at 50 mg/mL, which agrees with our previous report. At the same concentration of 50 µg/mL, the SCCAM solution has also increased glucose uptake with a value close to the NCRAE values.	[419]
Glucose uptake test and lipid accumulation assays.	Methanolic extract (CME) and CME/DT (detannification).	CME and CME/DT exhibited significant glucose uptake in 3T3-L1 adipocytes with a dose-dependent response. The glucose uptake profile in the presence of PI3K and IRTK inhibitors (Wortmannin and Genistein) substantiates the mechanism used by both extracts. CME inhibited the differentiation of 3T3-L1 preadipocytes but failed to show glucose uptake in inhibitor-treated cells. The activity exhibited by CME/DT is exactly opposite to CME. PTP1B inhibition assay, mRNA, and protein expression analysis revealed the unique behaviour of CME and CME/DT.	[420]
PTP1B Inhibition study.			
Glucose uptake assay.	12, 8-guaianolide sesquiterpene lactones isolated from butan-1-ol and ethyl acetate fractions of roots extract	The compounds significantly facilitated glucose uptake in the hyperglycemic HepG2 cell model at 50 μM.	[259]
*Dittrichia viscosa* subsp. *Viscosa* (Syn. *Inula viscosa*)			
A-GLU/A-AMY	Methanol: water (80:20%, *v*/*v*) extract of leaves.	IC50A-AMY: 1.381 ± 0.085 mg/mL; IC50A-GLU: 0.118 ± 0.02 mg/mL.	[258]
A-GLU/A-AMY	Methanol (MeOH), ethyl acetate (EtOAc), and chloroform (CHL) extracts of leaves.	IC50A-GLU-EtOAc: 29.9 ± 1.04 µg/mL; PI-A-AMY: 22.152 ± 0.387% IC50A-GLU-MeOH: 22.3 ± 2.82 µg/mL; PI-A-AMY: 27.162 ± 1.623% IC50A-GLU-Chlo: 39.8 ± 0.76 µg/mL; PI-A-AMY: 17.157 ± 0.634%	[256]
A-GLU/A-AMY	Tomentosin is extracted and purified from dichloromethane and ethanolic extract.	IC50A-GLU-26.61 ± 0.236 μM; IC50A-AMY: 26.89 ± 1.54 μM	[421]
Glucose uptake assay (IC8).	7-O-Methylaromadendrin (MAD) extracted from methanolic extract of the aerial part of the plant.	MAD significantly stimulated INS-induced glucose uptake. MAD increased the P2a and PPARg2 gene expression. MAD stimulated the reactivation of INS-mediated phosphorylation of PI3K-(Akt/PKB) and AMPK in high glucose-induced, INS-resistant HepG2 cells.	[422]
Study of aP2 and PPARg2 gene expression.			
*Galinsoga parviflora*			
A-GLU/A-AMY	Aqueous extracts of leaves.	At 2.5 mg/mL IA% (A-GLU): 40%, A-AMY: no inhibition	[423]
A-GLU	Two compounds, Galinsosides A (1) and B (2), flavanone glucosides extracted from methanolic extract of whole plant.	IC50A-GLU (1): 286 ± 0.68 μM; IC50A-GLU (2): 46.7 ± 0.32 μM.	[424]
*Helichrysum stoechas*			
A-GLU/DPP-4	Methanol extracts of aerial parts.	IC50A-GLU: 481.01 μg/mL, IC50DPP-4: 81.71 μg/mL.	[261]
*Hypochaeris radicata*			
A-GLU/A-AMY	Aqueous extracts of leaves. HR1: Extract fresh plant materials; HR2: Extract plant materials after blanching; HR3: Blanching water extract.	IC50A-GLU-HR1: 79.4 ± 1.7 μg/mL; IC50A-GLU-HR2: 99.1 ± 1.9 μg/mL; IC50A-GLU-HR3: 83.4 ± 1.8 μg/mL IC50A-AMY-HR1: 41.9 ± 1.4 μg/mL; IC50A-AMY-HR2: 84.5 ± 1.8 μg/mL; IC50A-AMY-HR3: 51.9 ± 1.5 μg/mL	[267]
*Lactuca serriola*			
A-GLU	4-hydroxybenzoic acid (1), protocatechuic acid (2), kaempferol (3), quercetin (4), lactuside A (5), luteolin-7-O-β-D-glucoside (6) are extracted from methanolic extracts of the leaves.	IC50A-GLU-(1): 810.31 ± 1.03 µM; IC50A-GLU-(2): 126.65 ± 1.82 µM; IC50A-GLU-(3): 39.72 ± 0.43 µM; IC50A-GLU-(4): 39.82 ± 1.12 µM; IC50A-GLU-(5): 468.98 ± 0.45 µM; IC50A-GLU-(6): 161.29 ± 0.31 µM.	[270]
*Senecio vulgaris*			
A-AMY	Methanol extract (MeOH = 1 mg/mL), Dichloromethane extract (DCM1 = 100 and DCM2 = 50 μg/mL).	MeOH-IA%: 82.46 ± 0.0041%, DCM1-IA%: 90.95 ± 0.0001%, DCM2-IA%: 59.05 ± 0.0001%.	[286]
ALDO	Methanol extracts of aerial parts.	IA%: 42.00% at 1mg/mL.	[425]
*Solidago virgaurea*			
A-GLU/A-AMY	Conc-ASE (concentrated extract obtained after accelerated solvent extraction) Conc-LE (concentrated extract obtained after laser extraction).	Conc-ASE = IC50A-GLU: 9.3 ± 0.9 µg/mL, IC50A-AMY-33.9 ± 2.4 µg/mL. Conc-LE = IC50A-GLU: 8.7 ± 0.6 µg/mL, IC50A-AMY-32.1±1.9 µg/mL.	[426]
*Sonchus oleraceus*			
Glucose uptake assay (IC13) Analysis of p-AMPK/Akt/GSK3-β expression in cells.	Hydroethanolic extract (90%) of the leaves (SOL).	The glucose uptake in HepG2 cells treated with 200 μg/mL SOL was significantly increased to 145%, but the uptake was lower than that treated with insulin (320%). After treatment with SOL extracts for 24 h, the p-AMPK, Akt, and GSK3β expression levels significantly increased by approximately 1.7, 1.0, and 0.8 times, respectively, compared with the control.	[427]
*Tanacetum parthenium*			
ra-ALDO/AGEs	Methanolic extract (70%) (ME) Ferulic acid (FA), Apigenin (API), Luteolin-7-O-glucoside (LUG), Luteolin (LUT), Chrysosplenol (CHR), Kaempferol (KAE), Santin (SAN) were extracted and purified from the methanolic extract.	ME: ra-ALDO-IA% (61.1 ± 0.5%), IC50-ra-ALDO (8.04 ± 0.61 µg/mL), IC50-AGEs (163.71 ± 6.31 µg/mL). FA: IC50-ra-ALDO (3.20 ±0.12 µg/mL), IC50-AGEs (5.59 ± 0.26 µg/mL). API: IC50-ra-ALDO (1.97 ± 0.10 µg/mL), IC50-AGEs (NA). LUG: IC50-ra-ALDO (1.31 ± 0.09 µg/mL), IC50-AGEs (3.43 ± 0.12 µg/mL). LUT: IC50-ra-ALDO (1.76 ± 0.03 µg/mL), IC50-AGEs (6.73 ± 0.43 µg/mL). CHR: IC50-ra-ALDO (1.92 ± 0.08 µg/mL), IC50-AGEs (NA). KAE: IC50-ra-ALDO (1.11 ± 0.03 µg/mL), IC50-AGEs (NA). SAN: IC50-ra-ALDO (NA), IC50-AGEs (NA).	[428]
A-GLU/A-AMY	Ethanolic extract of aerial parts. Extraction by accelerated solvent extraction (ASE), microwave-assisted extraction (MAE), maceration (MAC), Soxhlet (SOX), and ultrasound−assisted extraction (UAE).	ASE: IC50A-GLU (1.63 ± 0.02 mmol acarbose equivalent (ACAE)/g extracts), IC50A-AMY (0.51 ± 0.02 ACAE/g extract). MAE: IC50A-GLU (1.64 ± 0.01 mmol ACAE/g extracts), IC50A-AMY (0.53 ± 0.05 mmol ACAE/g extract). MAC: IC50A-GLU (1.65 ± 0.01 mmol ACAE/g extracts), IC50A-AMY (0.52 ± 0.02 mmol ACAE/g extract). SOX: IC50A-GLU (1.67 ± 0.01 mmol ACAE/g extracts), IC50A-AMY (0.51 ± 0.03 mmol ACAE/g extract). UAE: IC50A-GLU (1.64 ± 0.01 mmol ACAE/g extracts), IC50A-AMY (0.56 ± 0.01 mmol ACAE/g extract).	[429]
*Tanacetum vulgare*			
A-GLU/A-AMY	Hexan, hydroethanolic, and infusion of flowers (HEXF, HETF, INFF), stems (HEXS, HETS, INFS), and aerial parts (HEXAP, HETAP, INFAP).	HEXF: IC50A-GLU (10.41 ± 0.06 mmol acarbose equivalent (ACAE)/g extracts), IC50A-AMY (0.53 ± 0.01 mmol ACAE/g extract). HETF: IC50A-GLU (10.77 ± 0.15 mmol ACAE/g extracts), IC50A-AMY (0.33 ± 0.01 mmol ACAE/g extract). INFF: IC50A-GLU (3.57 ± 0.13 mmol ACAE/g extracts), IC50A-AMY (0.07 ± 0.01 mmol ACAE/g extract). HEXS: IC50A-GLU (10.60 ± 0.06 mmol ACAE/g extracts), IC50A-AMY (0.50 ± 0.02 mmol ACAE/g extract). HETS: IC50A-GLU (7.54 ± 0.65 mmol ACAE/g extracts), IC50A-AMY (0.33 ± 0.02 mmol ACAE/g extract). INFS: IC50A-GLU (4.00 ± 0.09 mmol ACAE/g extracts), IC50A-AMY (0.10 ± 0.01 mmol ACAE/g extract). HEXAP: IC50A-GLU (10.56 ± 0.04 mmol ACAE/g extracts), IC50A-AMY (0.48 ± 0.03 mmol ACAE/g extract). HETAP: IC50A-GLU (8.67 ± 1.19 mmol ACAE/g extracts), IC50A-AMY (0.35 ± 0.03 mmol ACAE/g extract). INFAP: IC50A-GLU (4.26 ± 0.12 mmol ACAE/g extracts), IC50A-AMY (0.09 ± 0.01 mmol ACAE/g extract).	[322]
*Lamiaceae*			
*Calamintha nepeta* subsp. *Nepeta* (Syn. *Clinopodium nepeta*)			
A-GLU/A-AMY	Methanolic extract (80%) of leaves.	At 10 mg/mL IA% (A-GLU): 66.62 ± 1.61%, IA% (A-AMY): 16.45 ± 0.94%	[333]
A-AMY	Methyl alcohol: water (7:3) extract fractionated with ethyl acetate (AcOEt), dichloromethane (DCM), and n-butanol (BuOH).	IC50A-AMY of DCM, AcOEt, and BuOH >200 µg/mL	[332]
A-AMY	Methanolic extract (ME), essential oil (EO), and aqueous extract (AQ).	IC50A-AMY-ME: 24.46 mg/mL, IC50A-AMY-EO: 31.54 mg/mL, IC50A-AMY-AQ: 115.47 mg/ml	[335]
*Lavandula pedunculata*			
A-GLU/A-AMY	Aqueous extract of flowering tops.	IC50A-AMY: 0.44 ± 0.05 mg/mL, IC50A-GLU-131 ± 20 mg/mL.	[430]
Intestinal Glucose Absorption in vitro		The extract inhibited the intestinal glucose absorption (IC50 = 81.28 ± 4.01 µg/mL) in a concentration-dependent manner.	
*Lavandula stoechas*			
A-GLU/A-AMY	Aqueous extract of aerial parts.	IC50A-AMY: 0.485 ± 0.13 mg/mL, IC50A-GLU: 168 ± 40.10 μg/mL	[431]
Intestinal Glucose Absorption assay, in situ.		The extract lowered intestinal glucose absorption in situ at 250 mg/kg.	
A-GLU/A-AMY	EO of aerial parts.	IC50A-AMY: 3.00 ± 0.008 mg/mL, IC50A-GLU: 2.58 ± 0.04 mg/mL	[347]
Glucose production assay (IC9)	Ethyl acetate (EE) and n-butanol (BE) fractions are prepared from an aqueous extract of aerial parts.	EE and BE at low doses (25–50 µg/mL) significantly decreased hepatic gluconeogenesis in the H4IIE cell line by suppressing the expression of PEPCK and G6Pase. In C2C12 myotubes, both extracts increased the INS-stimulated glucose uptake more effectively than metformin. They also effectively increased the expression of lipoprotein lipase protein levels in INS-resistant myotubes at low doses. EE increased the protein level of PPARγ and stimulated the activation of AKT in INS-resistant H4IIE and C2C12 cell lines.	[432]
Glucose uptake assay (IC10)			
Effects on PEPCK and G6Pase gene expression.			
Effects on AKT activation and GLUT4 expression.			
Transcriptome analyses			
A-GLU	Ursolic acid extracted from Methanol (ME), ethanol (ET), methanol-dichloromethane (1: 1, *v*/*v*) (MDI), acetone (AC), ethyl acetate (EA), diethyl ether (DEE), and chloroform extracts (CHL).	IC50A-GLU-ME: 49.86 ± 0.36 mg/mL, IC50A-GLU-ET: 17.81 ± 0.55 mg/mL, IC50A-GLU-MDI: 29.57 ± 0.19 mg/mL, IC50A-GLU-AC: 24.63 ± 0.13 mg/mL, IC50A-GLU-EA: 40.31 ± 0.84 mg/mL, IC50A-GLU-DEE: 23.60 ± 1.04 mg/mL, IC50A-GLU-CHL: 26.21 ± 1.00 mg/mL.	[346]
A-GLU	EO of flowering leaves.	IC50A-AMY: 106.73 ± 3.27 µg/mL, IC50A-GLU: 98.54 ± 4.84 µg/mL.	[344]
*Melissa officinalis*			
Anti-glycation assay.	Aqueous extract of leaves (AQ). Rosmarinic acid (RA), melitric acid A (MA), salviaic acid A (SA), caffeic acid (CA).	IC50-AQ: 0.24 mg/mL, IC50-RA: 0.34 mM, IC50-MA: 0.38 mM, IC50-SA: 0.16 mM, IC50-CA: 0.48 mM.	[433]
A-GLU/A-AMY	Aqueous extract of leaves	IA%: 83.9%, A-AMY: No activity.	[434]
A-AMY	Lemon balm-based extract with 50% RA	IA%: 50%	[435]
Uptake inhibition of glucose (UIG) and fructose (UIF) (IC12)	Methanolic and aqueous extract of leaves.	UIG%: <25%, UIF: No activity for both extracts.	[436]
Glucose consumption (IC8)	EO (A, B, and C compagnies)	EO-A: 63.64 ± 11.46%, EO-B: 59.96 ± 3.65%, EO-C: 65.63 ± 9.76%. The Western blot data suggest that the key factors of the adenosine monophosphate-activated protein kinase/acetyl-CoA carboxylase pathway can be mediated by the EOs.	[437]
Gene expressions analysis of p-AMPK, AMPK, p-ACC, ACC, PPAR, CEBPα, and SREBP1 proteins.			
*Mentha aquatica*			
A-AMY	Hydroethanolic extract (70%) of the leaves.	IC50A-AMY: 229.50 ± 4.1 µg/mL.	[438]
A-AMY	Methanolic (ME) and aqueous extracts (AQ) of the leaves.	IA%-ME: 61.7 ± 5.5%, IA%-AQ: 14.0 ± 3.0%	[439]
Uptake inhibition of glucose (UIG) and fructose (UIF) (IC12)	Methanolic and aqueous extract of leaves.	UIG%: <25%, UIF: No activity for both extracts.	[436]
*Mentha pulegium*			
A-GLU/A-AMY	Methanolic and aqueous extract of leaves.	IC50A-GLU-ME: 20.38 µg/mL, IC50A-GLU-AQ: 21.65 µg/mL, IC50A-AMY-ME: 23.11µg/mL, IC50A-GLU-AQ: 36.47 µg/mL	[372]
A-GLU/A-AMY	Ethyl acetate fraction of aerial part.	IC50A-GLU: 61.85 ± 1.69 µg/mL, IC50A-AMY: 16.37 ± 0.11 µg/mL	[440]
*Mentha suaveolens*			
A-GLU/A-AMY	EO of the aerial part.	IC50A-GLU: 141.16 ± 0.2 µg/mL, IC50A-AMY: 94.30 ± 0.06 µg/mL	[384]
*Origanum vulgare*			
A-AMY	Clonal oregano shoots ethanolic extracts (50%) (O-1, O-9, O-11Y, O-11M, O-12, O-17, OK-17, O-23, O-24, O-26, GO-19-1).	The strongest anti-amylase activity was observed for extract O-24, which had an AI index value of 2.32 ± 0.28 and corresponded to 57% inhibition of enzyme activity. Eight of the eleven clonal oregano extracts tested had AI index values greater than or equal to 1.5. For these experiments, an AI index value of 1.5 corresponded to approximately 33% α-amylase enzyme inhibition.	[441]
ALDO	Caffeic acid (CA), rosmarinic acid (RO), lithospermic acid B (LTO), 12-hydroxy jasmonic acid 12-O-β-glucopyranoside (HDG), p-menth-3-ene-1,2-diol 1-O-β-glucopyranoside (MDG) isolated from the polar extracts of aerial parts.	ALDO-CA: 8 ± 4.6%, ALDO-RO: 95 ± 0.0%, ALDO-LTO: 96 ± 1.7, ALDO-HDG: 77 ± 1.4%, ALDO-MDG: 41 ± 0.6%. EB-CA: −7.68 kcal/mol, EB-RO: 15.71 kcal/mol, EB-LTO: −16.08 kcal/mol, EB-HDG: −14.58 kcal/mol, EB-MDG: −10.57 kcal/mol.	[442]
Docking studies of ALDO inhibitory activity (EB).			
A-GLU/A-AMY	Aqueous and ethanolic (12%) extract of plant clonal lines.	At 1000 µg/mL: A-GLU (93.7%), A-AMY (95%).	[434]
Analysis of PPARγ- and δ-mediated transactivation, a test of adipogenic potential, INS-stimulated glucose uptake, neutral red assay.	*Origanum vulgare* ssp. *vulgare* (1): hexane (Hex), dichloromethane (DCM), and ethyl acetate (EtOAc) extracts of the aerial part. *Origanum vulgare* ssp. *hirtum* (2): dichloromethane (DCM), methanol (MeOH) extracts of the aerial part.	(1): Hex ext = Activation of the γ, δ PPARs, adipocyte differentiation (NA), INS-stimulated GLU-uptake (+), Viability of endothelial cells (NA), Viability of macrophages (NA), DCM ext = Activation of the γ PPARs, adipocyte differentiation (NA), INS-stimulated GLU-uptake (+), Viability of endothelial cells (-), Viability of macrophages (66.1 ± 5.3%). EtOAc ext = Activation of the γ PPARs, adipocyte differentiation (NA), INS-stimulated GLU-uptake (+), Viability of endothelial cells (NA), Viability of macrophages (2.7 ± 1.4%). (2): DCM ext = Activation of the γ PPARs, adipocyte differentiation (NA), INS-stimulated GLU-uptake (+), Viability of endothelial cells (NA), Viability of macrophages (NA), MeOH ext = Activation of the γ, δ PPARs, adipocyte differentiation (NA), INS-stimulated GLU-uptake (+), Viability of endothelial cells (NA), Viability of macrophages (NA).	[443]
DPP-IV/PTP1B	Methanolic extracts of leaves: commercial oregano extract (E1) and greenhouse-grown oregano extract (E2). Chemical fractionation by flash chromatography system (fractions FA to FI).	DPP-IV-IC50: (E1) = 28.4 ± 6.3 μM GAE, (E2) = 86.2 ± 18.8 μM GAE, FA > 500 μM GAE, FB = 206.3 ± 47.2 μM GAE, FC > 500 μM GAE, FD = 317.4 ± 60.7 μM GAE, FE = 20.3 ± 3.9 μM GAE, FF = 23.3 ± 1.9 μM GAE, FG = NA, FH = NA, FI = NA. PTP1B-IA: (E1)/(E2) = NA, FA = 7.0 ± 3.5%, FB = 13.3 ± 4.2%, FC = 1.3 ± 1.0%, FD = NA, FE = 32.1 ± 3.3%, FF = 77.4 ± 18.4%, FG = NA, FH = NA, FI = NA.	[444]
A-GLU/A-AMY	(1): EO of *O. vulgare* subsp. *Hirtum.* (2): EO of *O. vulgare* subsp. *Vulgare.*	IC50A-AMY (1): 0.14 ± 0.008 mmol ACEs/g; IC50A-GLU (1): 0.88 ± 0.03 mmol ACEs/g. IC50A-AMY (2): 0.13 ± 0.004 mmol ACEs/g, IC50A-GLU (2): 6.04 ± 0.91 mmol ACEs/g.	[390]
A-GLU/A-AMY	Methanolic extract (80%) of leaves.	A-GLU-IA = 58.41 ± 1.97%, A-AMY-IA = 6.79 ± 0.57%.	[333]
A-GLU	Aqueous acetonitrile (50%) of powder leaves.	IC50A-GLU = 0.35 ± 0.03 μg/mL, AGEs-IC50 = 0.55 ± 0.07 mg/mL. Cells treated with extract leaf extract at a 100 μg/mL concentration showed significantly enhanced 2-NBDG uptake compared with INS-treated cells. The extract decreased the promoter activity and the mRNA and protein expression of PEPCK and SREBP-1c. I. The extract inhibited the expression of CPY2E1 and enhanced the expression of GLUT2.	[393]
AGEs assay			
Glucose uptake assay (IC13).			
The mRNA and protein expression of PEPCK, SREBP-1c, GLUT2, CYP2E1 (IC14).			
A-GLU/A-AMY/LIPA	Ethanolic extracts (80% *v*/*v*).	IC50A-AMY = 44.71 ± 0.86 µg/mL, IC50A-GLU = 7.11 ± 1.37 µg/mL, IC50-LIPA = 922.35 ± 30.99 µg/mL.	[395]
*Prunella vulgaris*			
Study of Glucose Production (IC15).	Methanolic extract of arial part (PVA). Rosmarinic and caffeic acids were extracted from solid residue PVA by organic solvent, representing about 26% and 0.3% *w*/*w* of total extract.	The PVA lowered glucose production from glycogen (glycogenolysis), dihydroxyacetone, and alanine (gluconeogenesis). None of the phenolic acids influenced PEPCK mRNA expression, which INS downregulated. G6Pase mRNA was decreased by INS, increased by PVA, and remained unaffected by other treatments. Both compounds significantly increased GK mRNA expression; PVA did not affect this gene expression.	[445]
The mRNA expression analysis of G6Pase, GLUK, CALM, PEPCK, and GK (IC16).			
ra-ALDO/hu-ALDO/AGEs assay	Aqueous extract (AQE) partitioned sequentially with n-hexane (HEX), methylene chloride (CH2Cl2), ethyl acetate (EtOAc), n-butanol (BuOH), and water (H2O). Compounds (C1 to C6) isolated from AQE fractionation.	ra-ALDO: AQE-IA = 36.18 ± 1.13%, HEX-IA = 33.94 ± 0.49%, CH_2_Cl_2_-IA= 32.49 ± 0.54%, EtOAc-IA = 87.33 ± 2.39% (IC50 = 2.99 ± 0.10 μg/mL), BuOH-IA = 59.56 ± 2.34%, H2O-IA = NA, C1 = NA, C2 = NA, C3-IC50 = 8.35 ± 0.51 μM, C4-IC50 = 2.77 ± 0.48 μM, C5-IC50 = 3.20 ± 0.55 μM, C6 = NA. hu-ALDO: C1 = NA, C2 = NA, C3 = NA, C4-IC50 = 18.62 ± 0.40 μM, C5-IC50 = 12.58 ± 0.32 μM, C6 = NA. AGEs assay: AQE-IA = 29.26 ± 0.94%, HEX-IA = 33.94 ± 0.41%, CH2Cl2-IA = 54.03 ± 1.00% (IC50 = 186.72 ± 2.05 µg/mL), EtOAc-IA = 68.31 ± 1.06% (IC50 = 141.34 ± 1.27 µg/mL), BuOH-IA = 40.47 ± 0.68%, H2O-IA = 30.24 ± 1.01%, C1-IA = 9.33 ± 0.27%, C2= NA, C3 = NA, C4 = 20.67 ± 0.37%, C5-IA = 74.81 ± 1.41% (IC50 = 33.16 ± 0.54 µg/mL), C6-IA = 88.69 ± 0.56% (IC50 = 304.36 ± 3.41 µg/mL).	[446]
Cas-3 activity and activation of the apoptotic signaling pathway (IC16) analysis (Bax/Bcl-2, Fas/FasL, phospho-JNK, phospho-ERK, phospho-p38, NF-κB binding activity, phosphorylated-IκB, TNF-α, IL-6).	Aqueous extract (AQE).	AQE administration significantly prevented IL-1β-increased INS-1 cell death and LDH activity and attenuated IL-1β-increased cas-3 activity.	[447]
A-GLU/A-AMY	Hydroethanolic extract of inflorescence (PV) contained RA (4.5%), CA (9.8%), and pCA (11.6%).	IC50A-GLU-PV: 90.9 μg/mL, IC50A-AMY-PV: 47.2 μg/mL IC50A-GLU-CA: 4.7 μg/mL, IC50A-AMY-CA: 5.1 μg/mL IC50A-GLU-RA: 11.6 μg/mL, IC50A-AMY-RA: 21.7 μg/ml	[448]
NPMDA	Active compounds tested (Kaempferol, luteolin, delphinidin, quercetin, beta-sitosterol, spinasterol, stigmasterol, vulgaxanthin-I, poriferasterol monoglucoside, stigmast-7-enol, morin)	The sterols and flavonoids play an active role by affecting the TNF signalling pathway, AGE-RAGE signalling pathway, MAPK pathway, and PI3K-Akt pathway-related targets such as IL-6 and INS.	[158]
A-GLU	Hydroethanolic extract (75%) (HE) partitioned sequentially with Petroleum ether (PE), Ethyl acetate (EtOAc), n-butanol (BuOH), and water (H2O) fractions. Compounds Caffeic acid (C1), Isoquercitrin (C2), and Rosmarinic acid (C3) isolated from AQE fractionation.	HE-IC50 = 130.46 ± 4.33 µg/mL, PE-IC50 = 194.61 ± 4.69 µg/mL, EtOAc-IC50 = 69.13 ± 2.86 µg/mL, BuOH-IC50 = 124.97 ± 2.56 µg/mL, H2O-IC50 = 191.88 ± 3.34 µg/mL, C1-IC50 = 3.91 ± 0.07 µg/mL, C2-IC50 = 85.52 ± 2.94 µg/mL, C3-IC50 = 0.65 ± 0.04 µg/mL.	[449]
*Salvia verbenaca*			
A-GLU/A-AMY	Methanolic extract (85%) (ME) and decoction (DE) of the aerial part.	IC50A-GLU-ME: 50.5 ± 1.40 µg/mL, IC50A-AMY-ME = 101.30 ± 0.08 µg/mL. IC50A-GLU-DE: 313.7 ± 1.36 µg/mL, IC50A-AMY-DE = NA.	[399]
*Thymus mastichina*			
A-GLU/A-AMY	Essential oil.	IC50A-GLU = 100 ± 0.0 µg/mL, IC50A-AMY = 4600 ± 0.0 µg/mL.	[450]

ACC: Acetyl-CoA carboxylase, ADIP: Adiponectin AGEs: Advanced glycation end products inhibition assay, AI: Amylase inhibition, Akt: Protein kinase B, ALDO: Aldose reductase, AMPK: Adenosine 5-monophosphate-activated protein kinase, aP2: Adipocyte-specific fatty acid binding protein; A-AMY: Alpha amylase, A-GLU: Alpha glucosidase, Bax: Bcl-2–associated X protein, Bcl-2: Marker linked to germinal center B cells, CALM: Calmodulin, cas-3: Caspase, CEBPα: Transcription factor CCAAT/enhancer binding protein alpha, DPP-IV: dipeptidyl peptidase IV, EB: Energy binding (kcal/mol), ERK: Extracellular Signal-Regulated Kinases, GAPDH: Glyceraldehyde-3-phosphate dehydrogenase, GLUT4: Glucose transporter type 4, GLUK: Glucokinase, G6Pase: Glucose-6-phosphatase, hIR: Human insulin receptor, Hu-ALDO: Human recombinant aldose reductase, IL-1β: Beta 1 Interleukin, INS-1 cell: Rat Insulinoma Cell Line, IκB: IkappaB kinase, JNK: c-Jun N-terminal cinase, LEP: Leptin, LPL: Lipoprotein lipase, NA: Not active, NF-κB: Nuclear factor-kappa B, NPMDA: Network pharmacology and Molecular Docking Analyses. The potential action targets were (IL-6 and INS), and the signalling pathways were (the AGE-RAGE signalling pathway, TNF signalling pathway, MAPK signalling pathway, PI3K-AKT signalling pathway), PEPCK: Phosphoenolpyruvate carboxykinase, PI3K: Phosphatidylinositol 3-kinase, PKB: Protein kinase B, PPAR: Activating peroxisome proliferator-activated receptors, PPARg2: Peroxisome proliferator-activated receptor g2, PTP1B: Protein tyrosine phosphatase 1B, P38: Mitogen-activated protein kinases, ra-ALDO: Rat lens aldose reductase, SREBP1: Sterol Regulatory Element-binding Protein, TNF-α: Tumor necrosis factor. In vitro Cellular models studied. IC1: Rat insulinoma-derived INS-1 β-cells, IC2: L6 myocyte cells, IC3: Hepatocytes were isolated from rats fed ad lib., IC4: Microsomal fractions of hepatic cells, IC5: H4IIE cells (Rat hepatoma cell line), IC6: Changes in mitochondrial respiration and glycolysis, IC7: The P19 cell line (embryonic carcinoma cell line), IC8: 3T3-L1 (Murine preadipocyte cell line), IC9: HepG2 (Human hepatocellular liver carcinoma) and differentiated 3T3-L1. IC10: Palmitate-induced INS resistance model in H4IIE cells, IC11: Palmitate-induced INS resistance model in C2C12 cells (immortalized mouse myoblast cell line), IC12: Human colorectal adenocarcinoma Caco2 (Cancer coli-2) cells, IC13: HepG2 cells, IC14: HepG2 and E47 cells (Cellosaurus Hep G2-E47), IC15: Rat hepatocytes, IC16: Fao Cells differentiated derivatives of the clonal cell line H4IIEC3, which was derived from the Reuber H35 rat hepatoma, IC17: INS-1 cells (INS-1 832/13 Rat Insulinoma Cell Line), IC18: The pRB-deficient mouse embryonic fibroblasts (ME3), adipocyte differentiation 3T3-L1 cells, Hepa 1-6 cells (CRL1830), mouse hepatoma from BW7756 tumours in C57L mice, Murine monocyte/macrophage cell line RAW264.7, the human hybrid cell line EAhy926.

- *Anthemis canescens* var. *aurea*

The native range of *Anthemis canescens* var.* aurea* (syn. *Matricaria aurea*), also known as corn chamomile, is from the Mediterranean to the northwest of India and the Arabian Peninsula. It is annual and grows primarily in the subtropical biome [116]. Several scientific studies have examined these effects, notably Ismail et al. [217]. A T2DM rat model was used, along with identification of chemical components by LC-MS/MS (liquid chromatography coupled to tandem mass spectrometry), and enzyme activity assays, gene expression analyses by q-RTPCR, network pharmacology analyses, and molecular docking simulations were also carried out in an attempt to elucidate the molecular mechanism(s) of this plant’s antidiabetic effects [217]. The results showed that only the polar hydroethanol extract of *M. aurea* exhibited remarkable antidiabetic activity. In addition, it improved dyslipidaemia, insulin resistance status, ALT (alanine transaminase), and AST (aspartate aminotransferase) levels [217]. 

LC-MS/MS analysis of the hydroethanol extract identified 62 compounds, including the popular flavonoids of chamomile, apigenin and luteolin, other flavonoids and their glycosides, coumarin derivatives, and phenolic acids (Table 1). According to the authors [217], the 46 compounds selected were linked to 364 candidate T2DM targets. Network analysis enabled them to identify 123 pivotal proteins, including insulin signalling and metabolic proteins: IRS1 (insulin receptor substrate 1), IRS2 (insulin receptor substrate 2), PIK3R1 (phosphoinositide-3-kinase regulatory subunit 1), AKT1 (AKT serine/threonine kinase 1), AKT2 (AKT serine/threonine kinase 2), MAPK1 (mitogen-activated protein kinase 1), MAPK3 (mitogen-activated protein kinase 3), and PCK1 (phosphoenolpyruvate carboxykinase 1), inflammatory proteins TNF and IL1B (interleukin-1Beta), antioxidant enzymes: CAT (catalase), SOD (superoxide dismutase), and others [217]. Subsequent filtering enabled them to identify 40 crucial principal targets (major hubs) of *M. aurea* in treating T2DM. Functional enrichment analyses of the candidate targets revealed that the plant targets were mainly involved in the inflammatory, energy-sensing/endocrine/metabolic, and oxidative stress modules. According to Ismail et al. (2022), the hydroethanol extract of *M. aurea* is capable of significantly increasing PIK3R1 and decreasing IL1B, PCK1 (phosphoenolpyruvate carboxykinase 1), and MIR29A (microRNA 29a human gene) according to q-RTPCR gene expression analysis [217]. Based on experimental and computational analyses, this study revealed that *M. aurea* exerted antidiabetic action via simultaneous modulation of multiple targets and pathways, including inflammatory, energy-sensing/endocrine/metabolic, and oxidative stress pathways [217].

- *Bellis perennis* L. and *Bidens frondose*

The *Bellis* genus comprises around twenty species of small annual or perennial herbs found mainly in the Mediterranean region. *Bellis perennis* L. (common daisy) is widely distributed in continental Portugal and used in folk medicine (Supplementary Table S3). Many of its pharmaceutical functions derive from the antioxidant characteristics of its contents and its quantity of phenolic compounds. All parts of this plant have been studied, and several chemical compounds have been characterized. In a study by Haselgrübler et al. (2018), the effect of its ethanolic extracts (50%) revealed significant efficacy in inducing GLUT4 translocation in the in vitro cell system applied using a screening assay based on fluorescence microscopy [411]. The extracts also reduced blood glucose levels in chicken embryos (in vivo), confirming the plant activity in a living organism. 

According to the results of high-performance liquid chromatography (HPLC), the numerous polyphenolic compounds identified and quantified, including apigenin glycosides, quercitrin, and chlorogenic acid (Table 1), potentially contribute to stimulating the transfer of GLUT4 from the cytosolic zone to the plasma membrane, leading to decreased blood glucose levels [411]. Moreover, it was shown in the study by Nowicka and Wojdyło (2019) that the methanolic extract (50%) has a high content of triterpenoids, carotenoids, and flavonols, with the ability to inhibit *α*-amylase and *α*-glucosidase (Table 1). The species *Bidens frondosa* also displayed promising results in diabetes [412]. The acute hypoglycaemic activity of its ethanolic extract (80%) was studied in normoglycaemic, glucose-loaded, and STZ-induced diabetic rats [413]. The subacute antidiabetic effect was studied in an 8-day experiment. The extract showed a promising and significant hypoglycaemic impact in all the in vivo models tested [413]. The acute antidiabetic effect was 42% at 500 mg/kg. The *α*-glucosidase and *α*-amylase inhibitory activity of the extract was also determined and showed strong inhibition of the *α*-glucosidase enzyme (75.22% at 2 mg/mL) [413] (Table 2).

- *Chamaemelum nobile*

Roman chamomile, as *Chamaemelum nobile* (syn. *Anthemis nobilis* L. or *Chamomilla nobilis*), is an ornamental plant known as a medicinal plant since the Middle Ages [245]. It is native to Southwest Europe (France, Spain, and Portugal) but is present all over Europe, North Africa, and Southwest Asia [245]. The plant is cultivated mainly in England, Belgium, France, Germany, Hungary, Poland, Bulgaria, Egypt, and Argentina. Pharmacological studies have revealed a wide range of biological properties and a broad phytochemical diversity (Table 1). 

Among these studies, Eddouks et al. (2005) carried out a single study on the plant against diabetes. A single dose and daily oral administration (20 mg/kg BW) for 15 days tested the aqueous extract of the aerial part of Roman chamomille on blood glucose concentrations and basal insulin levels in normal and STZ-induced diabetic rats [451]. Single oral administration of the aqueous extract reduced blood glucose levels from 6.0 ± 0.3 mmol/L to 4.9 ± 0.09 mmol/L (*p* < 0.05) 6 h after administration in normal rats and from 21.1 ± 1.3 mmol/L to 14.5 ± 0.9 mmol/L (*p* < 0.001) in streptozotocin-induced diabetic rats [451]. In addition, blood glucose levels decreased from 6.1 ± 0.06 mmol/L to 4.6 ± 0.17 mmol/L and from 21.1 ± 1.31 mmol/L to 13.7 ± 0.90 mmol/L in normal and STZ diabetic rats, respectively, after 15 days of treatment [451]. Basal plasma insulin concentrations remained unchanged after treatment in normal and STZ diabetic rats. 

According to the authors, the mechanism of this pharmacological activity may be independent of insulin secretion [451]. It can exert hypoglycaemic activity in the gastrointestinal tract by slowing digestion and reducing the carbohydrate absorption rate [451]. Another study conducted on the compound chamaemeloside, an apigenin glycoside containing a hydroxymethylglutaric acid (HMG) fraction extracted from this plant [452], showed that it did not affect glucose uptake in cultured L6 muscle cells but reduced plasma glucose levels in SwissWebster mice by 19.2% and 31.9% at doses of 125 and 250 mg/kg, respectively. Chamaemeloside only exerted its effect after 4 h of i.p. (intraperitoneal) administration [452]. The effect on interpretandial blood glucose levels in normal rats and oral glucose tolerance was also studied. The results showed that interprandial blood glucose levels were unaffected, but that chamaemeloside significantly improved glucose tolerance 4 h after administration [452]. 

According to Witherup et al. (1995), chamaemeloside may influence glucose homeostasis via multiple mechanisms, including the release of HMG acid from chamaemeloside and its modulation of insulin secretion [452,453]. A clinical study was also conducted on the hypoglycemic effect of Roman chamomile [454]. Twenty-six pre-diabetic volunteers (twenty-one men and five women; mean age: 50.5 ± 8.5 years) were selected for an 8-week study of supplementation with mixed plant extracts combining a hot water extract of *Anthemis nobilis* (synonym of Roman chamomile), *Crataegus oxyacantha* (hawthorn berry), *Houttuynia cordata* (dokudami), and *Vitis vinifera* at a dose of 1200 mg [454]. The results showed that the mixture reduced abnormal glucose levels and the risk of developing diabetes. The underlying mechanism of action can be attributed to stimulating peripheral energy utilisation, particularly in muscle and adipose tissue [454].

- *Cichorium intybus* L.

Plants in the *Cichorium* genus are particularly valuable for their exceptional therapeutic and medicinal properties (Table 1). It includes plants from the dandelion tribe in the sunflower family, with ten to twelve species, four to six of which are wild species. They are herbaceous perennials, typically Mediterranean, native to Europe, western Asia, and North America. Among its species is *Cichorium intybus* L. (*Asteraceae*), commonly known as chicory, widely cultivated in many countries worldwide for its many traditional uses and as an edible food. Indeed, it has been included in Chinese Pharmacopoeia for its many beneficial effects on various ailments, from wounds to diabetes (Supplementary Table S3).

In a study by Pushparaj et al. (2007), the hypoglycaemic and hypolipidaemic properties of an ethanolic extract of *C. intybus* were tested on Sprague–Dawley rats treated with STZ (Table 3). The oral glucose tolerance test (OGTT) (a dose of 125 mg plant extract/kg BW) showed a potent hypoglycaemic effect. In addition, the daily administration of the extract (125 mg/kg) for 14 days to diabetic rats reduced serum glucose by 20%, triglycerides by 91% and total cholesterol by 16% [455]. However, there was no change in serum insulin levels, ruling out the possibility that the extract induces insulin secretion by pancreatic *β*-cells. 

This effect on pancreatic *β*-cells was also demonstrated in a study by Ghamarian et al. (2012). Aqueous chicory seed extract prevented BW loss and reduced fasting blood glucose levels in a four-week trial in rats (Table 3). Chicory appears to have both short-term (around 2 h) and long-term (28 days) effects on T2DM and is a natural food supplement for slowing the progression of diabetes [456]. The ethanolic extract of chicory seeds containing 9.6% caffeoylquinic acids improved blood sugar levels, reduced the atherogenic index, and increased blood antioxidant status during a 28-day treatment on Wistar rats (WR) [457]. 

In another study by Petrović et al. (2024), a polyherbal mixture composed of *Centaurium erythraea* aerial parts, *Cichorium intybus* roots, and *Potentilla erecta* rhizomes was tested to determine its potential toxicity in vivo and its effect on diabetic complications [458]. The results showed that treatment with the decoction had no toxic effect. Its antidiabetic activity was high in vitro and in vivo studies (Table 2 and Table 3). Fourteen days of treatment with the decoction (15 g/kg) completely normalized the blood glucose levels of diabetic animals, whereas treatment with insulin and glimepiride only slightly lowered glycaemic values [458]. In addition, the lipid status of the treated animals and the levels of AST, ALT, ALP, creatinine, urea, and MDA (malondialdehyde) were completely normalized [458]. In addition, the polyherbal mixture completely restored histopathological changes in the liver, the kidneys, and the four *Cornu ammonis* regions of the hippocampus. According to the authors, the ameliorative effect of the treatment was essentially due to bioactive compounds [458]. They are known for their hepatoprotective activities and ability to lower serum transaminases, ALP, and MDA [459,460], with even greater success than glimepiride [461,462]. 

On the other hand, treatment with the polyherbal mixture increased hepatic glycogen deposition via *β*-cell regeneration by the various compounds present in the preparation [458]. Indeed, hydroxybenzoic acid, one of the bioactive compounds in the tested polyherbal mixture, can regenerate *β*-cells and normal serum insulin and hepatic glycogen levels [463]. In addition, the hepatoprotective activity observed can also be explained by the high antioxidant activity of the mixture [458], which boosts antioxidant defense enzymes via the expression of Nrf2/cytochrome P450 2E1 (CYP2E1), reduces inflammation via the inactivation of MAPK/NF-κB (Nuclear factor-kappa B) signalling pathways, and reduces apoptosis via the regulation of B-cell lymphoma 2 (Bcl-2)/protein kinase B (AKT)/CAT expression [464]. 

Furthermore, caffeic acid in the herbal mixture decreases the level of MDA in the kidney and visibly reduces renal epithelial damage in the diabetic animal model [458]. At the same time, rutin administration regulates renal function and reduces the degree of renal tissue damage in induced nephropathy by downregulating TGF*β*-1 (transforming growth factor beta 1) and fibronectin expression [460,465]. Epicatechin also reduces lipid peroxidation in kidneys due to benzo rings and aromatic compounds that can bind hydroxyl radicals in tissues [466]. Hyperoside, in treating diabetic nephropathy, regulates blood urea and creatinine levels. It reduces renal tissue damage by suppressing the expression of fibronectin, collagen IV, and tissue inhibitors of metalloproteinase 1 (TIMP-1) while promoting the expression of matrix metalloproteinases 9 and 2 (MMP-9 and MMP-2) [467]. It also prevents further podocyte apoptosis in the glomerulus following diabetes, allowing the regeneration of damaged tissue via the miR-499-5p/APC (anaphase-promoting complex) axis [468]. In addition, isoquercetin possesses nephroprotective capacity through its hypoglycaemic, hypolipidaemic [469,470], and hepatoprotective activities, similar to sulphonylureas [470]. In addition, caffeic acid regulates lipid status and blood glucose. It attenuates oxidative damage in blood cells, liver cells, and kidney tissue [460] by upregulating nuclear erythroid-related factor 2 (NRF2) and downregulating NF-κB [471]. Caffeoylquinic or chlorogenic acids (CQAs), abundant intermediates of lignin biosynthesis in chicory, have also been reported to improve human glucose metabolism (Table 3). 

According to the study by Palatini Jackson et al. (2017), the three di-O-caffeoylquinic acids extracted from chicory suppressed hepatic glucose production in H4IIE rat hepatoma cells by reducing the expression of glucose-6-phosphatase (G6Pase) and phosphoenolpyruvate carboxykinase (PEPCK), two key enzymes that regulate hepatic gluconeogenesis [416]. Direct comparisons between CQAs and their metabolites (3-caffeoylquinic, caffeic, and quinic acids) revealed that the caffeic acid component alone was responsible for the effects observed [416]. Further analysis suggested that the activation of the PI3K (phosphoinositide 3-kinases) and MAPK pathways to control gene expression was common in caffeoylquinic and caffeic acids (Table 1). These compounds promoted increased mitochondrial respiration and cellular metabolism, inducing oxidative phosphorylation and proton leakage [416].

In a study by Azay-Milhau et al. (2013), the antihyperglycaemic effect of three hydroxycinnamic acids present in the roots of *C. intybus* was also tested (Table 1). In vitro experiments were carried out to compare the results of two hydroxycinnamic acids, caffeic and ferulic acids, with those obtained with chicoric acid extracted (CAE) (50 and 100 µg/mL) on the three main tissues involved in blood sugar regulation (pancreas, muscle, and liver) [414]. 

In vivo experiments were performed on WR given a daily intraperitoneal injection of CAE (3, 15, or 30 mg/kg) for four days (Table 3). An intraperitoneal glucose tolerance test (1 g/kg) was performed on the fourth day. The results showed that the three compounds used could induce an original response. Caffeic acid mainly reduces hepatic glycogenolysis [414]. Ferulic acid caused a marked increase in insulin release and a reduction in hepatic glycogenolysis. However, this compound also inhibited muscle glucose uptake. CAE increased insulin release and glucose uptake without affecting hepatic glycogenolysis (Table 3). The study showed that none of these compounds involved hepatic G6Pase, unlike chlorogenic acid, which is known to inhibit the enzyme and can reduce glucose production by hepatocytes [414]. These results underline that CAE can lower blood glucose levels without affecting the liver. The in vivo experiments provide evidence that daily i.p. administration of CAE improves i.p. glucose tolerance in a dose-dependent manner and mainly via an insulin sensitization effect [414].

**Table 3:** Medicinal plants in NPSE for diabetes management with scientific validation of the claimed antidiabetic effects (in vivo).

Part Used/Extract Tested	Model/Parameters Studies	Intervention and Duration	Observations	References
*Asteraceae*				
*Arctium minus* (Hill) Bernh				
R/DEC	Male diabetic GK (Goto-kakizak) rats.	125 g/L DR ad libitum/4 weeks.	The DEC led to a GK rats’ occasional glycaemia decrease. It did not significantly affect glycemic control; long-lasting treatments induced toxic effects. The DEC decreased several parameters of GK liver mitochondria respiratory activity.	[182]
R/L/AQ	ALLO-induced diabetic rats.	RAQ (500 mg/kg) and LAQ (200 mg/kg) OG/21 days.	RAQ was reduced by 34.6 ± 5.8%, and LAQ was reduced by 6.2 ± 22.9%.	[174]
	Measurement of biochemical parameters (B1)			
*Achillea millefolium*				
NI/HET	STZ-induced diabetic rats.	25 mg/kg/day and 100 mg/kg/day OG/28 days.	Compared with Metformin, the HET reduces lipid abnormality, HYG and hepatic enzymes with a dose-dependent effect in diabetic rats.	[402]
	Measurement of biochemical parameters (B2)			
NI/AQ/MET	OGTT	250 and 500 mg·kg^−1^ BW DR/18h.	The AQ/MET at dose levels of 250 and 500 mg/kg BW showed a significant decrease in BG level, TGL, VLDL, cholesterol, SGOT, SGPT, and ALP in diabetic rats.	[404]
	ALLO-induced diabetic rats.	250 and 500 mg/kg BW DR/14 days.		
	Measurement of biochemical parameters (B3)			
AP/HET (70%)	OGTT	100 mg/kg 0, 0.5, 1, 1.5, 2, and 3 h	The HET showed significant glucose diminution on oral glucose tolerance tests and in acute experimental T2DM assay. It reduced the BG levels in a dose-dependent manner.	[407]
	STZ-induced diabetic mice.	33, 100 and 330 mg/kg 1, 3, 5, and 7 h		
AP/HET	STZ-induced diabetic rats.	100 mg/kg/day i.p./14 days	The HET significantly reduced the expression of both IL-1β and iNOS genes. The serum INS levels in the HET group animals increased while the BG levels decreased significantly. The HET enhanced overexpression of IL-1β and iNOS genes, which may have a protective effect on β-cells.	[408]
	Measurement of biochemical parameters (B4) and IL-1β/iNOS gene expression.			
NI/HET	STZ-induced diabetic rats.	250 mg/kg NI/21 days	The results indicated that the HET improves renal function due to antioxidant activity and modulates some biochemical factors in diabetic rats.	[406]
	Measurement of biochemical parameters (B5) Analysis of oxidative stress-related factors (O1).			
*Anthemis canescens* (syn. *Matricaria aurea*)				
ET/EA/DCM/HEX	STZ-induced diabetic rats.	ETH1 = 100 mg/kg, ETH2 = 200 mg/kg EA1 = 100 mg/kg, EA2 = 200 mg/kg DCM1 = 100 mg/kg, DCM2 = 200 mg/kg HEX1 = 100 mg/kg, HEX2 = 200 mg/kg OG/4 weeks.	Treatment with either ETH1/2 extracts or pioglitazone successfully ameliorated INS resistance, hyperlipidemia, and fatty liver without significantly affecting fasting INS levels or pancreatic secretory capacity. It increased liver protection from injury associated with T2DM, as evidenced by a significant decrease in ALT and AST.	[217]
	Measurement of biochemical parameters (B6).			
	Oxidative stress and antioxidant markers in the liver (O2)			
*Bellis perennis*				
L/F/ EXT4404/EXT4407/HMET	Avian embryos in the first two-thirds of embryonic development (lasting 21 days) Hens egg test-chorioallantoic membrane (HET-CAM) assay.	EXT4404 (300 mg/L), EXT4407 (300 mg/L), HMET (300 mg/L).	All three extracts resulted in a comparable decrease in BG levels (~20% after 1 h and 30% after 2 h) and were statistically significant after 2 h incubation. The three extracts significantly reduced BG levels at both time points with comparable efficacy (~12% after 1 and 2 h).	[411]
*Bidens frondosa*				
AP/ET (80%)	OGTT	250 and 500 mg/kg BW OG/7 days	ET exhibited weak to moderate hypoglycaemic effects on normoglycemic rats at the tested doses. In OGTT, higher doses of the extract indicated significant inhibitory activities. The ET lowered BG levels in varying ratios. The body weight of the animals was not changed significantly during this experiment.	[413]
	Healthy and STZ-induced diabetic rats.			
	Measurement of biochemical parameters (B1)			
*Chamaemelum nobile* (syn. *Anthemis nobilis* L. or *Chamomilla nobilis*)				
AP/AQ	STZ-induced diabetic rats.	20 mg/kg BW OG/15 days.	Single oral administration of AQ reduced BG levels in normal and STZ diabetic rats. BG levels were decreased in normal and STZ diabetic rats, respectively, after 15 days of treatment.	[451]
	Measurement of biochemical parameters (B7)			
*Cichorium intybus*				
R/AQ	ALLO-induced diabetic rat	2.5, 5, 10, and 15 g DPM/kg OG/2 weeks	Treatment with 10 g/kg of the herbal mixture significantly lowered glycaemic values compared with the diabetic controls. The treatment with the highest tested concentration (15 g/kg) completely restored BG level to normoglycemic values in experimental groups. The lipid status of treated animals and serum AST, ALT, ALP, CRE, URE, and MDA were completely normalised. The polyherbal mixture completely restored the histopathological changes in the liver, kidneys, and hippocampus.	[458]
	Measurement of physiological and biochemical parameters (P1)			
WP/ET (80%)	STZ-induced diabetic rats (male Sprague–Dawley rats)	125 mg of plant extract/kg BW OG/14 days	Daily administration of ET (125 mg/kg) for 14 days to diabetic rats attenuated BG by 20%, TG by 91%, and tTC by 16%.	[455]
	OGTT			
	Measurement of biochemical parameters (B8)			
R/AQ	STZ- niacinamide (NIA/STZ) induced diabetic rats	125 mg/kg BW i.p. injections/28 days.	The extract prevented body-weight loss and decreased BG level. ALT activities and TG, TC, and HbA1c levels decreased, and NO concentration increased in the chicory-treated groups. Unlike late-stage diabetes, fasting serum INS concentrations were higher, and the OGTT pattern was approximated to normal in chicory-treated early-stage diabetic rats.	[456]
	Measurement of biochemical parameters (B9)			
	OGTT			
AP/CAE	Healthy rats	3, 15 or 30 mg/kg i.p. injections/4 days.	The CAE can decrease BG without any effect hepatic effect. Daily i.p. administrations of CAE improve i.p. glucose tolerance in a dose-dependent manner, mainly via an INS sensitising effect.	[414]
	OGTT			
	Measurement of biochemical parameters (B10)			
S/CQA-ET	Healthy rats	Diet with CQA-ET FEE/28 days	The CQA-ET was found to decrease the atherogenic index to the level observed in the control rats’ group and to increase blood antioxidant status. Both dietary supplements reduced the content of thiobarbituric acid-reactive substances in kidney and heart tissue compared with the experimental group.	[457]
	High-fructose diets			
	Measurement of physiological and biochemical parameters (P2)			
	Antioxidant status of rats (O3)			
NCRAE, SCCAM	STZ-induced diabetic rats	15 mg/kg i.p. injections/7 days.	Both NCRAE and SCCAM can improve glucose tolerance in STZ diabetic rats after a subchronic administration of seven days. Alone, NCRAE significantly decreases the basal HYG after six days of treatment.	[419]
	OGTT			
	Measurement of biochemical parameters (B11).			
*Dittrichia viscosa* subsp. *iscosa* (Syn. *Inula viscosa*)				
L/AuNPs	High-fat diet (HFD)/STZ-induced diabetes in rats	2.5 mg/kg i.p. injections/21 days.	Treatment with AuNP significantly lowered the BG level, the gene expression, and the activity of hepatic PEPCK in comparison with the untreated diabetic group. The AuNPs synthesised can alleviate HYG in HFD/STZ-induced diabetes in rats by reducing hepatic gluconeogenesis by inhibiting the expression and activity of the hepatic PEPCK gene.	[472]
	Measurement of biochemical parameters (B12)			
AP/AQ	Normal and STZ-induced diabetes rats	20 mg/kg OG/for 2 weeks.	A significant reduction in BG levels was observed in normal rats 2 h after a single oral administration. Repeated daily oral administration significantly reduced BG levels after 4 days of treatment. In diabetic rats, a significant reduction in BG levels was observed 1 h after a single oral administration. Repeated oral administration reduced BG levels on the 4th day. No change in TC and TG levels was observed after a single and repeated oral administration in both normal and diabetic rats. Plasma INS levels and body weight remained unchanged after 15 days of repeated oral administration in normal and diabetic rats.	[473]
	Measurement of biochemical parameters (B13)			
*Galinsoga parviflora*				
WP/HET 80%	STZ-induced diabetic rats.	400 mg/kg BW NI	The extract reduced the BG level equivalent to GLIB (5 mg/kg BW) in diabetic rats.	[474]
	Measurement of biochemical parameters (B13)			
*Lactuca serriola*				
L/AQ	STZ-induced diabetic rats.	200 and 500 mg/kg BW OG/NI	Both doses of extracts restored β -cell function and INS secretion.	[475]
	OGTT			
	Measurement of biochemical parameters (B14)			
*Onopordum acanthium*				
L/MET	STZ-induced diabetic rats	200 and 400 mg/kg OG/8 days	Administration of extracts significantly increased INS content in β-cells with a marked enhancement of pancreatic islet structure, significantly reducing BG level and BW loss. Extract treatment suppressed the increase in inflammatory cell score in myocardial tissue with an M2–like macrophage elevation.	[476]
	Measurement of biochemical parameters (B15)			
*Solidago virgaurea*				
AP/HE	ALLO-induced diabetic rat.	250 mg/kg BW OG 15 days	Extract significantly reduced BG level, serum AMY activity, TNF-α level, and pancreatic MDA level, as well as increasing the serum INS, liver GLY level, pancreatic SOD, and CAT activities in comparison with their corresponding diabetic rats.	[477]
	Measurement of physiological and biochemical parameters (P3)			
*Sonchus asper*				
NI/ME	STZ-induced diabetic rats.	200 mg/kg 21 days	The ME improve the activity of the antioxidant enzymes, TBARS contents, and cholesterol profile of the diabetic rats. DTR’s BG and INS levels were significantly lower in treatment than the diabetic rats on day 21.	[478]
	Measurement of physiological and biochemical parameters (P4)			
*Sonchus oleraceus*				
WP/ME	STZ-induced diabetic rats.	75, 150, 300 mg/kg 14 days	The Me (150 mg/kg) treatment exhibited 39.40% glycaemia reduction. The measurement of stress markers in plasma, liver, and kidney after ME administration showed a significant reduction in MDA and hydrogen peroxide levels, coupled with a substantial increase in CAT activity.	[479]
	OGTT			
	Antioxidant status of rats (O4)			
L/HET (90%)	STZ-induced diabetes in rats	100, 200, 400 mg/kg/day BW 6 weeks	HET significantly increased both SOD activity and GSH levels while causing a reduction of MDA levels in the liver. Moreover, HET ameliorates STZ-induced liver function and pathological damage. DTR fed with HET daily for 6 weeks showed significantly decreased levels of TNF-α and IL-1β in the liver. HET decreased MyD88, TGF-β, and TLR4 expression levels in the liver of DTR.	[480]
	Measurement of physiological and biochemical parameters (P5)			
L/HET (90%)	HFD/STZ-induced diabetes in rats	100, 200, 400 mg/kg/day BW 6 weeks	In DTR treated by HET (400 mg/kg/day for 6 weeks), TG, TC, and LDL-c were reduced by 43%, 22%, and 16%, respectively. Meanwhile, it was also found that daily feeding of DTR decreased plasma glucose levels by approximately 23%. DTR with HET at 400 mg/kg/day for 6 weeks show portal tract and mild fibrous expansion without sep inflammation formed of lymphocytes. The administration of HET exhibited a protective effect against the hepatic damage induced by STZ, which was also corroborated by the apparent condition and colour observed in HET-administered rats.	[427]
	OGTT			
	Measurement of physiological and biochemical parameters (P6)			
L/ET (80%)	ALLO-induced diabetic rat	100, 200, and 300 mg/kg BW 56 days	The treatment of SOE 200 and 300 mg/kg in diabetic rats for two months dramatically decreased BG, total lipid, TC, TG, and LDLc, while HDLc levels improved liver and kidney functions. The histological assay revealed that the treatment of SOE 300 mg/kg significantly improved the pancreas tissues.	[481]
	Measurement of physiological and biochemical parameters (P7)			
***Lamiaceae***				
*Lavandula pedunculata*				
FTO/AQ	Healthy rats	1 g/kg BW NI	Acute and chronic oral administration of extract reduced the peak of the BG (30 min) and the area under the curve. The effect was at the same amplitude as the positive control Metformin.	[430]
	Acute OGTT and chronic			
	OGTT for plant mixtures			
	Measurement of biochemical parameters (B13)			
*Lavandula stoechas*				
AP/EO	ALLO-induced diabetic rat	50 mg/kg BW i.p. injections/15 days.	Subacute EO administration prevented BW gain decline and protected against alloxan-induced increase in hepatic and renal relative weights. EO treatment corrected the BG level significantly, protected against lipoperoxidation and decreased (−SH) group levels, and reversed antioxidant enzyme depletion. Induced by alloxan treatment. Treatment with EO significantly protected against hepatic and renal dysfunctions and the disturbance of lipid metabolic parameters induced by alloxan treatment.	[482]
	Measurement of biochemical parameters (B16)			
AP/EO	ALLO-induced diabetic rat	50 mg and 160 mg/kg BW i.p. injections/15 days	EO treatment protects against decreased BW gain, relative reproductive organ weights, testosterone level, and sperm quality decline. EO treatment protects against oxidative damage to DTR’s male reproductive organ systems.	[483]
	Measurement of physiological and biochemical parameters (P8)			
R/ET (70%)	ALLO-induced diabetic rat	50, 100, and 150 mg/kg BW i.p. injections/NI	The extract significantly reduced BG levels of DTR in a dose-dependent manner.	[484]
	Measurement of biochemical parameters (B13)			
NI/EO	STZ-induced diabetic rats	0.05 mL DDR/21 days	The percentage of healing was highest in the EO group on Days 7, 14, and 21. Microscopic examination of the biopsies supported accelerated wound healing on Days 7 and 14. Reduced mononuclear cell density and increased hair follicle and adipose tissue development were observed in the T2DM-EO group on Day 7. On Day 14, the T2DM-EO group increased collagen levels and hair follicles.	[485]
	Wound healing test			
	Measurement of physiological and biochemical parameters (P9)			
AP/AQ	ALLO-induced diabetic rat	150 mg/kg OG/NI	Oral extract administration reduced HYG induced by sucrose and starch in normal and diabetic rats.	[431]
	OGTT			
	Measurement of biochemical parameters (B13)			
*Melissa officinalis*				
EO	db/db mice	0.0125 mg EO/d FEE/6 weeks	Mice administered EO for 6 weeks showed significantly reduced BG (65%; *p* < 0.05) and TG concentrations, improved glucose tolerance, as assessed by an OGTT, and significantly higher serum INS levels than the CGr. All the genes were significantly upregulated, whereas G6Pase and PEPCK expression was downregulated in the livers of the EO group.	[486]
	Measurement of biochemical parameters (B17)			
	OGTT			
L/ET	HFD C57BL/6 mice	200 mg/kg/day FEE/6 weeks	The DTR revealed significantly reduced fasting BG concentrations (14% decrease versus vehicle). The extract showed no significant effects on FPIL. It significantly decreased the HFD-induced INS resistance by 35%. It reduced the HFD-provoked rise in fasting plasma concentrations of nonesterified FAs by 59% and plasma TAG gain by 66%. The extract-fed mice showed reduced plasma levels of LDL/VLDL-c (32% decrease) and a slight decrease in TC (8% decrease). The extract treatment led to an increase in the HDL/LDL ratio of 56%.	[487]
	Measurement of biochemical parameters (B18)			
NI/EO	STZ-induced diabetic rats	0.01, 0.02 and 0.04 mg/day FEE/4 weeks	EO at both high doses restored glycemia and reduced the BW of DTR compared with untreated diabetic animals.	[488]
	Measurement of biochemical parameters (B1)			
L/HAE	ALLO-induced diabetic rat	20, 100 or 500 mg/kg BW OG/4 weeks	There was a significant decrease in blood sugar levels, TC, TG, and LDL in DTR with HAE. An increase in HDL levels was observed in HAE-DTR.	[489]
	Measurement of biochemical parameters (B19)			
L/HE-EA (ALS-L1023)	HFD C57BL/6 mice	HFD supplemented with 0.4% (*w*/*w*) ALS-L1023 (HFD-ALS) FEE/12 weeks	Administration of ALS-L1023 to high-fat-diet-induced OMI caused reductions in BW gain, VFM, and VAS without changes in FC profiles. ALS-L1023 improved HYG, HYIN, BG, and INIT and normalised INS-positive β-cell area in OMI. ALS-L1023 decreased hepatic LIA and concomitantly increased the expression of PPARα target genes responsible for fatty acid β-oxidation in livers.	[490]
	Measurement of physiological and biochemical parameters (P10)			
	OGTT and IPITT			
L/HE/EA	Otsuka Long-Evans Tokushima fatty (OLETF) rats	HFD with 0.4% or 0.8% (*w*/*w*) of extract FEE/4 weeks	The EAE administration resulted in a BW reduction without changes in FI. It also markedly inhibited HYG and HYIN, restoring the β-cell mass severely impaired in OLETF rats. There was a decrease in LIA in the liver and skeletal muscle of the ORAT after treatment with EAE. After EAE treatment, the liver and skeletal muscle increases the expression levels of FAs-oxidizing enzymes (AMPKα2, ACOX, MCAD, and VLCAD). The EAE attenuated the pancreatic inflammation, including the infiltration of CD68-positive macrophages and mast cells, and attenuated the expression of inflammatory factors (IL-6 and CD68).	[491]
	Measurement of physiological and biochemical parameters (P111)			
*Mentha aquatica*				
L/AQ	STZ-induced diabetic rats	50 mg, 100 mg and 150 mg OG/90 days.	FBG and HbA1c levels decreased in DTR. The BW and INS levels of DTR were significantly increased. The levels of TC TG were reduced, and the levels of HDL were significantly increased. The ALB of DTR were significantly increased. However, the levels of UR and CREA were decreased in DTR. TBARS/MDA level formation significantly decreased in DTR. The activities of CAT, SOD, GPx, and GST were increased in DTR. DTR at a dose of 100 mg/kg bw/day showed normal glomeruli, normal intertubular vessels, and tubular epithelial cells, indicating degenerative changes in the kidney.	[492]
	Measurement of physiological and biochemical parameters (P12)			
*Mentha pulegium*				
AP/AQ	STZ-induced diabetic rats	20 mg/kg BW OG/15 days	The AQE caused a significant reduction in BG levels in DTR. The morphometric analysis and histological sections realised in the pancreas and liver have shown the beneficial effect of AQE in the cellular population. According to OGTT, the AQE has improved glucose tolerance in normal rats.	[493]
	OGTT			
	Measurement of physiological and biochemical parameters (P13)			
AP/AQ	STZ-induced diabetic rats	20 mg/kg BW OG/15 days	The AQE alleviated hyperlipidemia in diabetic rats by lowering significantly the TC levels without affecting the TG levels significantly. It exerted some increasing activity on plasma HDL-c level.	[494]
	Measurement of biochemical parameters (B20)			
*Mentha suaveolens*				
AP/AQ	STZ-induced diabetic rats.	20 mg/kg BW OG/15 days	The AQE decrease the BG, TC, and TG levels in both normal and diabetic rats. The AQE treatment was demonstrated to act positively on the liver and pancreas histopathological tissues.	[495]
	OGTT			
	Measurement of physiological and biochemical parameters (P14)			
*Origanum vulgare* L.				
L/AQ	STZ-induced diabetic rats	20 mg/kg OG/2 weeks	The AQE produced a significant decrease in BG levels in DTR. The BG levels were normalised from the fourth day after daily repeated oral administration of AQE. No changes in basal plasma INS concentrations were observed after treatment in either normal or DTR.	[496]
	Measurement of biochemical parameters (B4)			
L/AQ	STZ-induced diabetic rats	20 mg/kg OG/6 weeks	Administration of AQE significantly decreased BG levels, HbA1c, and AMY in DTR.	[497]
	Measurement of biochemical parameters (B21).			
L/ME/AQ	STZ-induced diabetic rats	5 mg/kg per day i.p. injections/10 days	ME reduced diabetes incidence and preserved normal insulin secretion. ME scavenged reactive oxygen and nitrogen species and alleviated the need to upregulate antioxidant enzymes. ME treatment attenuated the pro-inflammatory response mediated by T helper 17 cells. It enhanced anti-inflammatory T helper 2 and T regulatory cells by impacting specific signalling pathways and transcription factors.	[394]
	Measurement of physiological and biochemical parameters (P15)			
L/EtOAc	STZ-induced diabetic rats	2 mg/mouse OG/10 days	EtOAc treatment significantly preserved pancreatic islets and reduced diabetes incidence in DTR. Besides the down-modulatory effect on macrophages, EtOAc reduced the number of total CD4+ and activated CD4+ CD25+ T cells. EtOAc affected the number of T helper 1 (Th1) and T helper 17 (Th17) cells by downregulating their key transcription factors T-bet and RORγT.	[498]
	Measurement of physiological and biochemical parameters (P16)			
L/AQ	ALLO-induced diabetic rat	150 mg/kg, 300 mg/kg BW 300 mg/kg Equal mixture (150 mg chamomile + 150 mg oregano) OG/6 weeks	Treatment with higher or lower doses or a mixture of extracts had significant weight gain, hypoglycemic effect, decreased AMY activity, and increased INS levels. Restoration of the renal profile and lipid profile with an increase in HDL-C and the reversal of Bax and Bcl-2 were well observed, with a 300 mg/kg mixture showing synergistic activity of the extracts compared with individual low doses of 150 mg/kg.	[499]
	Measurement of biochemical parameters (B22)			
L/HE	Glucose-induced-diabetic zebrafish	10 μg/L FEE/24H	The BG level, TC, and TG were significantly reduced in diabetic zebrafish treated.	[500]
	Measurement of biochemical parameters (B23)			
L/INF	ALLO-induced diabetic rat	55 mL OG/40 days	The INF reduced BG levels after the first day of treatment, compared with the diabetic CGr. The INF appears to stimulate INS secretion.	[501]
	Measurement of biochemical parameters (B10)			
*Prunella vulgaris*				
WP/AQ	db/db mice HCF/HFD	100 mg, 200 mg/kg/day DR/8 weeks	AQE treatment markedly lowered BG and SBP. The CRE clearance was restored by treatment with AQE. The AQE markedly decreased TC, TG, LDL-c, MDA, and TGFβ1 and increased HDL-c and NO levels. AQE ameliorated vascular relaxation of aortic rings by acetylcholine or SNP-inducement in a dose-dependent manner. AQE treatment significantly reduced the aortic expressions of ICAM-1, VCAM-1, ET-1, and nitrotyrosine. The expression of eNOS in aortic was increased by AQE treatment.	[502]
	Measurement of physiological and biochemical parameters (P17)			
Fr/HE/TAP	STZ-induced diabetic rats	50 mg/kg, 100 mg/kg, 200 mg/kg of TAP OG/6 weeks	The BW and the levels of BG, FMN, MDA, NO, and the activity of NOS in serum decreased significantly compared with the STZ group in a dose-dependent manner. The activity of SOD in serum and BW increased significantly compared with the STZ group in a dose-dependent manner. The DTR showed a significant increase in SOD mRNA expression in pancreatic β cells. Histopathological examination also showed the protective effect of TAP on pancreatic β cells.	[503]
	Measurement of physiological and biochemical parameters (P18)			
HFOR/HE/AQ	Male CD-1 (ICR) mice/FFF	8.02 g/kg OG/10 weeks	HEE could improve glucose intolerance and normalise the lipid profile. HEE provokes an increase in peripheral and hepatic INS sensitivity, a decrease in FAs level, enhanced GLUK activity and GLY content, and improved serum antioxidant activity. Hepatic histopathological examination showed that HEE administration markedly decreased fatty deposits in the liver of mice.	[504]
	OGTT and IPITT			
	Measurement of physiological and biochemical parameters (P19)			
IF/PV(HE)/CARF/CA/RA	ALLO-induced diabetic rat model	50, 100, 150 mg/kg i.p. injections/8 weeks	CARF reduced BG levels and improved in vivo oxidative stress. CARF reduced HbA1c levels more significantly than PV and equivalent amounts of CA or RA. CARF had significantly increased serum-INS, and ameliorated thermal hyperalgesia and tactile allodynia more significantly than the effects of PV and equivalent amounts of CA or RA. The tested compounds showed potential restoration of the lipid peroxide levels.	[448]
	Measurement of physiological and biochemical parameters (P20)			
WP/AQ	Male Sprague-Dawley (SD) STZ-induced diabetic rats	100 mg, 300 mg/kg/day DR/8 weeks	In DTR, AQE significantly decreased BG and BUN and ameliorated plasma CRE. AQE reduced the PAS positivity staining intensity and basement membrane thickening in the glomeruli of DTR.	[505]
	Measurement of physiological and biochemical parameters (P21)			

ACOX: Acyl-CoA oxidase, ALB: Albumin, ALLO: Alloxan, ALP: Alkaline Phosphatase, ALT: Alanine aminotransferase, AMPKα2: AMP-activated protein kinase α2, AMY: Amylase, AP: Arial parts, AQ: Aqueous extract, AST: Aspartate aminotransferase, AuNPs: Gold particles of dried leaf aqueous extract, BG: Blood glucose, BUN: Urea nitrogen, BW: Body weight, CA: Caffeic acid, CAE: Chicoric acid extracted and purified from water extract, CARF: Caffeic acid-rich fraction, CAT: Catalase, CGr: Control group, CQA-ET: Caffeoylquinic acids-rich ethanol extract (75%) from chicory seeds (9.6% of CQA), CRA: L-chicoric acid, CRE: Creatinine, CGA: Chlorogenic acid, DCM: Dichloromethane extract, DDR: Daily dressings, DPM: Dry plant material, DR: Drinking, DTR: Diabetic treated rats, EA: Ethyl Acetate extract, EO: Essential oil, ET: Ethanol extract, EXT4404: Mixture of flowers and leaves ethanolic extracts, EXT4407: Ethanolic extract of flowers alone, FBG: Fasting blood glucose, FC: Food consumption, FEE: Feeding, FFF: Fed with a fructose/fat-rich combination diet, FI: Food intake, FPIL: Fasting plasma insulin levels, FTO: Flowering tops of plants, GK1: Hepatic glucokinase, GLIB: Glibenclamide, GLUT4: Glucose transporter type 4, GLUK: Glucokinase, GLY: glycogen, GPx: Glutathione peroxidase, G6Pase: Glucose-6-phosphatase, HAE: Hydroalcoholic extract, HbA1c: Glycosylated haemoglobin, HFD: High cholesterol food, HDL-c: High-density lipoprotein cholesterol, HE: Hexane extract, HET: Hydroethanolic extract, HFD: High fat diet, HFOR: Ethanolic extract of herbal formulation composed of *R. dioscorea*, *L. barbarum*, *P. vulgaris*, and hawthorn in a ratio of 6:4:2:3, HMET: Homemade ethanolic extract prepared from flowers collected locally, HYG: hyperglycemia, HYIN: Hyperinsulinemia, IL-6: Interleukin 6, INS: Insulin, INIT: Insulin tolerance, IPITT: Intraperitoneal insulin tolerance tests, LDH: Lactate dehydrogenase, LDL-c: Low-density lipoprotein cholesterol, LIA: Lipid accumulation, ME: Methanolic extract, MCAD: Medium-chain acyl-CoA dehydrogenase, MDA: Malondialdehyde, NCRAE: Natural chicoric acid extract (from hydroethanolic extract 70%), NI: Not indicated, NO: Nitric oxide, OG: Oral gavage, OGTT: Oral glucose tolerance tests, OMI: Obese mice, ORAT: Obese rats, PAL: Phosphatase alkaline, PASS: Periodic acid schiff, PEPCK: Phosphoenolpyruvate carboxykinase, PV: *Prunella vulgaris*, RA: Rosmarinic acid, SBP: Systolic blood pressure, SCCAM: Synthetic chicoric and chlorogenic acids mixture containing the two major compounds of NCRAE, in proportion of 70% of synthetic L-chicoric acid and 30% of synthetic chlorogenic acid, SGOT: Glutamic-oxaloacetic transaminase, SGPT: Serum glutamate pyruvate transaminase, SOD: Superoxide dismutase, STZ: Streptozine, TAG: Triacylglycerol, TAP: triterpenic acid from *Prunella vulgaris* hydroethanolic extract (75%), TC: Serum total cholesterol, TG: Triglycerides, TGF-β: Transforming growth factor beta, TLR4: Toll-like receptor, T2DM: Type 2 diabetes, UA: Uric acid, URE: Plasma urea, VAS: Visceral adipocyte size, VFM: Visceral fat mass, VLCAD: Very long-chain acyl-CoA dehydrogenase, VLDL: Very-low-density lipoprotein levels, WP: Whole plant. Biochemical parameters studied. B1: Assessment of the body weight (BW) and blood glucose (BG) levels; B2: Assessment of the BW, BG, TG, and total-, LDL-c, and HDL-c levels, B3: Assessment of the BW, BG, TC, TG, VLDL levels, SGOT, SGPT, and ALP activities, B4: Assessment of the BW, BG, and insulin levels (INS), B5: Assessment of BG, TC, HDL-c, TG, and LDL-c levels, B6: Assessment of fasting BG and INS. Systemic insulin resistance was estimated using the homeostasis model assessment index for insulin resistance (HOMA-IR). Study the triglyceride and glucose (TyG) index, insulin sensitivity, and the secretory capacity of the pancreas (HOMA-B index). Assessment of lipid profile and liver injury markers TC, TG, HDL-c, LDL-c, ALT, and AST, B7: Assessment of BG levels and basal plasma INS concentrations, B8: Assessment of the BW, serum glucose, TG, TC, and INS levels, B9: Assessment of the BW, fasting BG, intraperitoneal glucose tolerance test (IPGTT), HbA1c, ALT, AST, NO, TAG, TC, total protein (TPRO), and INS levels, B10: Assessment of BG and INS levels, B11: Assessment of the BW, food intake (FI), and plasma/urinary glucose levels, B12: Assessment of BG level. Hepatic gene expression and activity of phosphoenolpyruvate carboxykinase (PEPCK), B13: Assessment of BW, BG, TG, and TC levels, B14: Assessment of BG, HbA1c, INS, TC, TG, and LDL-c levels. IR-HOMA and HOMA-B (β-cell function) were evaluated using the homeostatic model assessment (HOMA) index, B15: Assessment of BG, BW, morphometric analysis, histopathological study, and immunohistochemical analysis, B16: Assessment of BG, biochemical determination of protein, -SH groups, MDA, and antioxidant enzyme activities (SOD, CAT) in the liver and the kidney homogenates. Assessment of liver function (AST, ALT, PAL, LDH). Assessment of renal function (URE, CRE, UA, and ALB analyses). Determination of metabolic parameters (TC, LDL-c, HDL-c, and TG concentrations), B17: Assessment of BG, INS levels, TC, TG, and HDL-c levels. GK1, GLUT4, adipocyte GLUT4, PPAR-γ, PPAR-α, SREBP-1c, G6Pase, PEPCK expression analysis, B18: Assessment of BG, TAG, FAs, and LDL/VLDL-c levels, B19: Assessment of BG level, TC, TG, LDL, and HDL contents, B20: Assessment of TC, TG, and HDL-c levels, B21: Assessment of BG, INS, liver and muscle glycogen, URE, UA, CRE contents, and pancreatic AMY, B22: Assessment of BG, INS, HbA1c, TC, TG, HDL-C, VLDL-c, LDL-c, URE, CRE, UA, TPRO, and AMY, B23: Assessment of BG level, TC, and TG levels.

*Antioxidant status parameters studied*. O1: Analysis of oxidative stress-related factors in renal tissue: MDA level, SOD, and glutathione peroxidase (GPx) enzyme activity, measurement of Bcl-2-associated X protein (BAX) expression, O2: Assessment of the level of MDA, reduced glutathione (GSH) levels, and catalase and SOD activities. O3: Analysis of oxidative stress-related factors: GPx, SOD, serum antioxidant capacity [hydrophilic substances, lipophilic substances, TBARS (Thiobarbituric Acid Reactive Substances) in heart, kidney, and liver tissues, O4: Titration of markers of oxidative stress in treated rats (MDA and hydrogen peroxide levels, CAT activity).

*Physiological and biochemical parameters studied*. P1: Assessment of the BW, BG level, TC, HDL, TG, AST, ALT, ALP, CRE, and URE. Sub-chronic toxicity study and histopathological analysis, P2: Determination of diet intake, BW, the mass of selected organs of rats; indices of gut functioning of rats; basic biochemical indices of rat serum (BG, ALT, ALP, TC, HDL-c, TG, Atherogenic index), P3: Assessment of BG, INS, serum lipid profile, tumor necrosis factor-α (TNF-α), liver glycogen levels (GLY), and amylase activity (AMY). Histopathological study of pancreatic tissue, SOD, CAT, and MDA levels in pancreatic tissue was also assessed, P4: Assessment of BG, INS, lipase, measurement of the protein concentration of the supernatant from pancreatic tissue. Determination of lipid peroxidation enzymes, glutathione-S-transferase (GST), glutathione reductase (GR), GPx, lipid peroxidation (TBARS), CAT, and SOD activities, P5: Assessment of BG, SOD, GSH activities, MDA, GLY levels, histological analyses of the liver tissue sample, determination of the levels of IL-1β and TNF-α and gene expression of NF-κB, TNF-α, IL-1β, p-TLR4, MyD88, and TGF-β analysis in the liver tissue sample, P6: Assessment of BG, BW, TG, TC, HDL, LDL, GLY levels, immunohistochemistry of the liver tissues and analysis of p-AMPK/Akt/GSK3-β expression in liver cells, P7: Assessment of BG, TG, TC, HDL, LDL levels, activities of liver enzymes, including (ALT) and (AST), as well as the serum total bilirubin (TB), total protein, and serum ALB, URE, UA, and CRE. Determination of MDA levels, GSH, SOD, CAT, and GST activities and histological analyses of the pancreatic tissue samples, P8: Evaluation of serum testosterone and sperm characteristics (count, motility, viability, morphology, production). Assessment of plasma glucose, relative body and reproductive organ weights, plasma acetylcholinesterase and butyrylcholinesterase activities. Biochemical determination of protein, -SH groups, MDA, and antioxidant enzyme activities (SOD, CAT), GPx in homogenates of testis, epididymis, and sperm, P9: Assessment of BG levels, BW. Macroscopic and microscopic data analysis, P10: Assessment of BW, FI, BG, HbA1c, TG, and FAs. QUICKI (quantitative insulin sensitivity check index) and HOMA-IR values were determined. Histological analysis (quantification of the visceral adipocyte sizes, INS-secreting β-cells detection), P11: Assessment of BG, INS levels, TAG, FAs, and body weight. Histology (quantification of the epididymal adipocytes size, examination of the lipid accumulation in the liver and gastrocnemius muscle tissues), immunohistochemistry (detection of INS and CD68), and real-time polymerase chain reaction, P12: Assessment of BG, BW, HbA1c, TC, TG, URE, CRE, ALB, INS, HDL-c, ALB, URE, CRE levels. Determination of antioxidant enzyme activities (SOD, CAT, GPx, GST) and the kidney lipid oxidative degradation (TBARS, MDA contents), P13: Assessment of BG, the histological sections and morphometric analysis, P14: Assessment of BG, TC, TG, LDL, HDL contents and histopathological examination of pancreas and liver, P15: Assessment of BG, INS, metabolic parameters (Glutathione S-transferase), and leucocyte and erythrocyte blood counts. Isolation of pancreatic-infiltrating mononuclear cells and immunofluorescence analysis. Determination of cytokine and nitrite levels. Histology and immunohistochemistry of the pancreas. Determination of antioxidant enzyme activity (Catalase activity, Glutathione peroxidase (GSHPx), Glutathione reductase), P16: Assessment of BG, determination of cellular composition and cytokine production and histopathological examination of pancreas, P17: Assessment of BG level, BW, urine volume, urine osmolality, and electrolytes (sodium, potassium, and chloride), systolic blood pressure (SBP), INS levels, transforming growth factor-beta1 (TGF-β1) and total NO levels, TC, HDL, cholesterol, LDL-c, TG, blood urea nitrogen (BUN), total bilirubin, TPRO, albumin (ALB), globulin, glutamic oxaloacetic transaminase (GOT), glutamic pyruvic transaminase (GPT), lactate dehydrogenase (LDH), AMY, and MDA levels in plasma. Histological and immuno-histological examinations, P18: Measurement of BG, BW, fructosamine (FMN), nitric oxide (NO), nitric oxide synthase (NOS), MDA, and SOD. Histopathological studies of rat pancreatic islet cells and expression of SOD mRNA in pancreatic β-cells, P19: Assessment of BG, HOMA-IR index, hepatic glucokinase activity, hepatic glycogen content, serum lipid profile (TC, TG, LDL-c, HDL-c contents), FAs level, serum antioxidant status (total antioxidant capacity [T-AOC], SOD, MDA), and histological examination of liver, P20: Assessment of BG level, BW, INS level, HbA1c, diabetic neuropathy management (hot plate latency test, tail flick latency test, Von Frey filaments test), measurement of lipid peroxidation and serum catalase (CAT) levels, P21: Assessment of BG level, BUN, and creatinine (CRE). Immunohistochemistry and Western blot analysis of the kidneys.

#### 3.2.2. *Lamiaceae* Family

- *Lavandula stoechas*

*Lavandula stoechas* L. is widely recognised for its pharmacological properties [341]. It is one of the best-known and most economically valued plants and was probably the first species to be used for its EO. The genus comprises around 39 species, numerous hybrids, and almost 400 registered cultivars [506]. Phytochemical studies of its many co-products have revealed the presence of a variety of bioactive compounds (Table 1). This plant is frequently used in traditional medicine to treat various conditions, including inflammation, neurological disorders, microbial infections, etc. [159,341,507,508]. It is the subject of much attention from diabetes scientists, and numerous in vitro, animal, and clinical studies have been carried out on its use (Table 3).

Elrherabi et al. (2023) evaluated the antihyperglycaemic impact of the aqueous extract of *L. stoechas* and attempted to explore its mechanisms. The researchers used an OGTT on normal and diabetic WR, administering 150 mg/kg of extract. Hyperglycaemia induced by sucrose and starch was reduced under the effect of the plant in normal and diabetic rats. The extract also caused a decrease in intestinal glucose absorption in situ at 250 mg/kg. As a result, the aqueous extract would have clear antihyperglycaemic effects attributed to inhibiting intestinal glucose absorption and key enzymes in monosaccharide digestion, such as *α*-amylase and *α*-glucosidase [431]. Indeed, the IC50 equals 0.485 ± 0.13 mg/mL and 168 ± 40.10 μg/mL, respectively, for amylase and glucosidase [431]. In addition, the antidiabetic potential of lavender essential oil and its main compounds was also investigated by measuring their inhibitory activity towards the two digestive enzymes [344]. Camphor (76.92 ± 2.43 μg/mL) and fenchone (69.03 ± 2.31 μg/mL) showed the best inhibitory activities for the *α*-amylase and *α*-glucosidase tests, respectively. The essential oil had an IC50 equal to 98.54 µg/mL. Interestingly, all the elements in this study had higher activities than acarbose, whatever the test adopted [344]. 

A study by Kulabas et al. (2018) aimed to elucidate the potential ameliorative effects of aqueous extracts of *L. stoechas* on insulin resistance and inflammation patterns using multi-targeted in vitro approaches and also to elucidate the mechanism of action by analysing transcriptional and metabolic responses [432]. The anti-insulin-resistant effects of ethyl acetate (EE) and butanol (BE) extracts prepared from the aqueous extract were assessed on the palmitate-induced insulin resistance model of H4IIE (rat hepatoma cell line), C2C12 (immortalized mouse myoblast cell line), and 3T3L1 (murine adipocytes) cells using several metabolic parameters. Specifically, whole genome transcriptome analysis was performed using microarrays on over 55,000 genes in control-, insulin-, and EE (25 µg/mL)-treated H4IIE cells. Both extracts at low doses (25–50 µg/mL) significantly decreased hepatic gluconeogenesis in the H4IIE cell line by suppressing PEPCK and G6Pase expression [432]. In C2C12 myotubes, both extracts increased insulin-stimulated glucose uptake more effectively than metformin. Both extracts decreased isoproterenol-induced lipolysis in the 3T3L1 cell line. In addition, they also effectively increased lipoprotein lipase protein expression in insulin-resistant myotubes at low doses. EE increased PPAR*γ* protein levels and stimulated AKT activation in insulin-resistant H4IIE and C2C12 cell lines [432]. 

The results obtained from biochemical analyses, mRNA/protein studies, and whole genome transcriptome analyses were complementary. They supported the hypothesis that EE may be biologically active against insulin resistance and act via the inhibition of hepatic gluconeogenesis and the activation of AKT. According to the study data, *L. stoechas* EE contains phytochemicals that may be effective in the treatment/prevention of insulin resistance and inflammation [432].

In the study by Sebai et al. (2013), EO of *L. stoechas* (LSEO), collected in the Ain-Draham region (northwest Tunisia), were tested for their protective effects against ALO-induced diabetes and oxidative stress in rats [482]. Antidiabetic and antioxidant activities were assessed after a subacute intraperitoneal injection of LSEO (50 mg/kg BW, i.p.) to rats for 15 days. They found that the EO significantly protected against increased blood glucose and decreased antioxidant enzyme activities induced by ALO treatment. Subacute treatment with EO induced a decrease in lipoperoxidation and an increase in antioxidant enzyme activities. These results suggest that LSEO protect against diabetes and oxidative stress induced by ALO treatment. These effects are partly due to its powerful antioxidant properties [482].

On the other hand, two other studies blended lavender with other plants to study their joint efficacy against diabetes. The first was by Sebai et al. [483], who sought to assess the protective effect of *Rosmarinus officinalis* EO (ROEO) and *L. stoechas* EO (LSEO) against ALO-induced reproductive damage and oxidative stress in diabetic male rats. The results showed that treatment with ROEO and LSEO protected against ALO-induced decreases in BW gain, relative reproductive organ weights, testosterone levels, and sperm quality. They also showed that the administration of ALO was accompanied by a state of oxidative stress assessed by an increase in levels of MDA and hydrogen peroxide (H_2_O_2_), as well as by a decrease in the sulphydril group (-SH) content and antioxidant enzyme activities such as SOD, CAT, and glutathione peroxidase (GPx) in the testes, epididymis, and spermatozoa [483]. More importantly, treatment with ROEO and LSEO significantly protected against oxidative damage to male reproductive organs in ALO-induced diabetic rats. The study’s results suggest that ROEO and LSEO potentially protect against ALO-induced damage to reproductive function and oxidative stress in male rats. According to the authors, the beneficial effect of ROEO and LSEO could be linked, in part, to their antioxidant properties [483].

In the other study by Mustafa et al. [484], hydroalcoholic extracts of *L. stoechas, Curcuma longa*, *Aegle marmelos*, and *Glycyrrhiza glabra* and their polyherbal preparation (PHP) were tested for their antihyperglycaemic potential in ALO-induced diabetic mice. The plant extracts tested significantly reduced the blood glucose concentration of the diabetes-induced mice dose-dependently. According to the authors, all the medicinal plants studied, with mixed PHP, had antihyperglycaemic activity, possibly due to bioactive phytoconstituents in the plants. However, more extensive studies are needed to identify, isolate and characterise the bioactive phytoconstituents responsible for the antihyperglycaemic activity of the medicinal plants studied [484].

- *Melissa officinalis*

*Melissa officinalis,* or lemon balm, is a medicinal plant native to southern Europe and the southern Mediterranean, northern Africa, and western Asia [509]. It is a bushy, herbaceous perennial commonly cultivated in herb and border gardens for its distinctive lemon-fragrant leaves. Numerous studies have described its therapeutic and pharmacological benefits thanks to its wealth of bioactive compounds (Table 1). It is particularly known for managing hyperlipidaemia, diabetes, and other metabolic syndromes [509,510].

Numerous studies have described the antidiabetic potential of this plant (Table 2 and Table 3). In cell models, *M. officinalis* extracts stimulated the expression of PPAR*α* target genes involved in fatty acid β-oxidation and lipolysis [437]. Lemon balm EO significantly activated the AMPK/ACC (acetyl-CoA carboxylase) pathway. The activation of AMPK allows cells to take up more glucose from the environment and downregulate ACC, thereby inhibiting lipid accumulation in adipocytes. The effect of EO on the expression of key transcription factors, such as C/EBPR (CCAAT-enhancer-binding protein R), SREBP1 (sterol regulatory element-binding transcription factor 1), and PPAR*γ*, was assessed using Western blotting analysis [437]. Compared with untreated adipocytes, the presence of lemon balm EO during adipogenic differentiation caused an increase in the expression levels of SREBP1 and C/EBPR but not PPAR*γ*. Although increased expression levels of SREBP1 and C/EBPR should increase lipid accumulation in cells, activated ACC proteins can inhibit lipid synthesis [437]. 

Finally, the Western blot data confirm and explain why lemon balm EO caused adipocytes to use more glucose but inhibited lipid accumulation [437]. These results are supported by those obtained in vivo. Mice given EO (0.0125 mg/d) for six weeks showed a significant reduction in glycaemia and TG concentrations, improved glucose tolerance, and significantly higher serum insulin levels than the control group. In addition, of all the glucose metabolism-related genes studied, hepatic glucokinase and GLUT4 and adipocyte GLUT4, PPAR*γ*, PPAR*α*, and SREBP-1c expression were significantly upregulated. In contrast, G6Pase and PEPCK were downregulated in the livers of the EO-treated group [437]. 

Another study was conducted on the hydroalcoholic extract of lemon balm (ALS-L1023) to examine its effect on the regulation of hepatic lipid accumulation, obesity, and insulin resistance and determine whether its mechanism of action involves PPAR*α* [490]. Indeed, excessive lipid accumulation in non-adipose tissue is thought to be responsible for developing insulin resistance. Activating the PPAR*α* receptor would reduce weight gain and improve insulin sensitivity in obese mice [511,512]. In this study, the administration of ALS-L1023 (food supplemented with 0.4% (*w*/*w*)) to obese mice (induced by a high-fat diet) resulted in a reduction in weight gain, visceral fat mass, and visceral adipocyte size without any change in food consumption profiles [490]. 

ALS-L1023 improved hyperglycaemia, hyperinsulinaemia, glucose and insulin tolerance, and normalised insulin-positive *β*-cell surface area in obese mice [490]. ALS-L1023 decreased hepatic lipid accumulation and concomitantly increased the expression of PPAR*α* target genes responsible for fatty acid *β*-oxidation in the liver [490]. Higher phosphorylated protein kinase B (*p*Akt)/Akt ratios and a lower expression of gluconeogenesis genes were also observed in the livers of ALS-L1023-treated mice. According to the authors, ALS-L1023 may inhibit obesity and improve insulin sensitivity in part by inhibiting hepatic lipid accumulation via hepatic PPAR*α* activation [490].

In a similar study by Shin et al. (2021), the administration of ALS-L1023 (HFD with 0.4% or 0.8% (*w*/*w*)) for four weeks to Otsuka Long-Evans Tokushima fatty (OLETF) rats resulted in a reduction in BW with no change in food intake [491]. The extract also markedly inhibited hyperglycaemia and hypoinsulinaemia and restored *β*-cell mass, severely impaired in rats. A reduction in lipid accumulation in the liver and skeletal muscle of obese rats was observed after treatment with ALS-L1023 [491]. In parallel, the expression levels of fatty acid oxidising enzymes [AMPK*α*2, ACOX (Acyl-CoA Oxidase), MCAD (Medium-chain acyl-coenzyme A dehydrogenase), and VLCAD (Very Long Chain Acyl-CoA Dehydrogenase Deficiency)] increased in liver and skeletal muscle after ALS-L1023 treatment. In addition, ALS-L1023 attenuated pancreatic inflammation, including the infiltration of CD68-positive macrophages and mast cells, and attenuated the expression of inflammatory factors (IL-6 and CD68) [491].

- *Mentha* sp.

The mint family includes 25 species in Europe, Africa, America, and Australia [376,513]. These are the herbs most widely used in traditional medicine for nutritional and medical purposes since ancient times (Supplementary Table S3). Because of their ability to grow rapidly and invade and tolerate a wide range of agro-climatic conditions, they are now cultivated and distributed worldwide. Their therapeutic benefits derive mainly from their chemical composition, which has many properties (Table 1). In addition to these medicinal effects, mint and its various species are also known for their antidiabetic value (Table 2 and Table 3). 

The effect of oral administration of the aqueous extract of the three plants *Mentha aquatica, M. pulegium*, and *M. suaveolens* was evaluated on the glycemic and lipid profiles of normal and streptozotocin-induced diabetic rats. A dose of 20 mg of the aqueous extract of *M. pulegium* and *M. suaveolens* was effective against diabetes. They produced significant hypoglycaemic effects in normal and diabetic rats [493,494,495]. In addition, a significant influence on glucose tolerance was also observed by the aqueous extract of *M. suaveolens* [495]. Both extracts showed an improvement in TC (total cholesterol) and TG levels, while no significant effect of the *M. suaveolens* extract was observed on serum lipoproteins (HDL and LDL (low-density lipoprotein)] [494,495]. In addition, both plants acted positively on histopathological tissues of the liver and pancreas. Furthermore, in a study by Yellanur et al. (2020) a dose of 100 mg/kg bw/day of *Mentha aquatica* aqueous extract for 90 days significantly reduced levels of fasting glycaemia, HbA1c (glycated hemoglobin test), TC, TG, plasma urea, creatinine, urinary albumin, and renal lipid peroxidation, and increased BW, insulin, HDL cholesterol, plasma albumin, urinary urea, urinary creatinine, and antioxidant enzyme activities (CAT, SOD, GPx, and GST) [492]. The authors demonstrated that the aqueous extract of *M. aquatica* leaves has antidiabetic activity by stimulating insulin secretion and potential nephroprotective activity by reducing lipid peroxidation and enhancing the scavenging capacity of the antioxidant defence system in the body [492].

- *Origanum vulgare* L.

*Origanum vulgare* (oregano) is another of the best-known plants in the *Lamiaceae* family, traditionally used to control and treat diabetes (Supplementary Table S3). It is an annual, perennial, and shrubby plant native to the Mediterranean, Euro-Siberian, and Iranian-Siberian regions [514]. The main constituents of oregano are quercetin, apigenin, kaempferol, naringenin, eriodictyol, salvianolic acid B, lithospermic acid B, amburosides A, luteolin, luteolin 7-*O*-glucuronide, apigenin 7-*O*-glucuronide, epigallocatechin, catechin, rutin, etc. (Table 1). These compounds have been reported to have several pharmacological activities, including antibacterial, anti-fungal, antiviral, antiparasitic, antioxidant, anti-inflammatory, anti-tumor, and antidiabetic (Table 1). They hold great promise for the design of new plant-based medicines, and derivatives of these compounds are being produced to assess their pharmacological effects for future use, particularly in the treatment of diabetes [515]. Antidiabetic characteristics have been previously identified in oregano, and it is currently one of the most sought-after plants for treating hyperglycaemia due to its wide accessibility, potency and lack of adverse effects [84].

Oregano can potentially inhibit the enzymatic activity of pancreatic amylase and glucosidase. This property may be part of the mechanism by which oregano may be used in the treatment/prevention of T2DM. According to the results of a study by McCue et al. (2004), extracts of clonal lines of oregano have strong inhibitory activity against porcine pancreatic amylase in vitro [441]. The inhibition rate depended on the clonal line and varied from 9% to 57%. This difference was because the clonal lines tested were characterized by a difference in the phenolic distribution of the main components (rosmarinic acid, quercetin, protocatechuic acid, and *p*-coumaric acid) [441]. However, the clonal line with the highest rosmarinic acid content (O-11Y with 114.0 μg phenolic/200 μg total phenolic content per extract) showed weak inhibition of *α*-amylase. 

According to McCue et al. (2004), the antidiabetic potential of the different clonal lines involves a synergistic role between rosmarinic acid and other identified (quercetin and *p*-coumaric acid) and unidentified phenolics [441]. Gonçalves et al. (2017) also reported the efficacy of methanolic extracts of oregano obtained in Portugal on the enzymatic inhibition activity of *α*-glucosidase and *α*-amylase [333]. The results showed that inhibition was more specific and higher for α-glucosidase than for *α*-amylase. This observation was particularly linked to the content of phenolic compounds in oregano identified by HPLC. High contents of rosmarinic acid (23.53 mg/g dry extract), (-)-epicatechin (4.65 mg/g dry extract), 3,4-dihydroxybenzoic acid (1.97 mg/g dry extract), gallic acid (1.19 mg/g dry extract), and (+)-catechin (1.03 mg/g dry extract) were reported [333]. These results are also consistent with Kwon et al.’s (2006) study on aqueous and ethanolic extracts (12%) [434]. The aqueous extract showed a higher dose-dependent inhibitory activity of *α*-glucosidase than the ethanolic extracts. It was also related to the phenolic content of each extract. Indeed, the aqueous oregano extracts showed a higher rosmarinic acid content than the ethanolic extract (16.5 vs. 3.78 mg/g dry weight, respectively). Rosmarinic acid was then tested and showed an inhibitory activity of 85.1% [434]. 

Dipeptidyl peptidase IV (DPP-IV) inhibition is another hypoglycaemic target of oregano that may be involved [516]. In a study by Bower et al., 2014, it was shown that the methanolic extract of commercial Greek oregano (28.4 ± 6.3 μM) was a better inhibitor of DPP-IV than greenhouse-grown Greek oregano extract (86.2 ± 18.8 μM) [444]. The chemical fractions designed according to flash chromatography retention times OE and OF from Greek oregano were the most potent inhibitors of DPP-IV (IC50 from 20.3 to 23.3 µM) [444]. As previously indicated, the oregano species’ phenolic content and distribution are related to the inhibitory activity of αglucosidase and *α*-amylase; this is also the case for DPP-IV. Of all the greenhouse-grown herbs tested in the study, Greek oregano extracts contained the highest concentration of polyphenols (430.1 ± 17.1 μg of GAE/mg DW). Considering the extract’s yield, the total polyphenol concentration for greenhouse-dried Greek oregano herbs was 6452 mg GAE/100 g DW. 

According to these studies (Table 1), oregano has numerous active compounds that can act through multiple actions and give rise to synergistic or antagonistic interactions (Table 1). Thus, the utilisation of plant-derived antioxidants with antidiabetic qualities, such as the action of DPP-IV inhibitors, is considered the most effective strategy for maintaining normal *β*-cell physiology and treating diabetes [517]. One of the advantages of therapies based on DPP-IV inhibition is their perceived impact on improving HbA1c, fasting glycaemia, and 2 h postprandial glycaemia [518]. In addition, most studies have reported its beneficial effects on regulating triglycerides (TG), HDL-c and LDL-c [519,520]. The DPP-IV inhibitor has fewer side effects, such as hypoglycaemia, increased BW, and gastrointestinal disorders. Glucose tolerance tests in animals have shown improved glucose tolerance and increased insulin secretion thanks to the genetic deletion of DPP-IV. In clinical observations, some DPP4 inhibitors influence enzymatic activity, reducing the baseline value by more than 50% [521]. 

Another important target in treating diabetes is the peroxisome proliferator-activated receptor (PPAR) *γ*, a key regulator of adipogenesis and glucose homeostasis. In Christensen et al.’s (2009) study, extracts of common medicinal/food plants, including oregano, were tested in a screening platform comprising a series of bioassays, including PPAR *γ*, *α*, and *δ* transactivation, adipocyte differentiation, and insulin-stimulated glucose uptake, to identify plants containing potentially interesting PPAR agonists [443]. Hexane (Hex), dichloromethane (DCM), and ethyl acetate (EtOAc) extracts of *Origanum vulgare* ssp. *vulgare*, dichloromethane, and methanol (MeOH) extracts of the aerial parts of *Origanum vulgare* ssp. *hirtum* were tested. According to the study results, all the extracts activated PPAR *γ* and increased insulin-stimulated glucose uptake. In addition, hexane and methanolic extracts activated PPAR *γ* and *δ*. However, they did not affect endothelial cells or adipocyte differentiation [443].

At the cellular level, Yu et al. (2021) evaluated the hypoglycemic effect of leaf extract on HepG2 and HepG2- GFP-CYP2E1 (E47) cells [393]. This work examined the plant’s potential for promoting glucose uptake, inhibiting glycosylation, and alleviating oxidative stress. The promoter activity, the mRNA and protein expression of PEPCK and SREBP-1c, and the expression of CPY2E1 and GLUT2 in the antidiabetic capacity mediated by oregano were analysed in HepG2 and E47 cells. The inhibition of PEPCK activity by the extract will effectively reduce sugar isogenesis and lower blood glucose levels, as it is a key enzyme in gluconeogenesis [393]. Studying the transcriptional and translational levels of SREBP-1c in HepG2 cells is an important step in analyzing carbohydrate and lipid metabolism. Indeed, these proteins are highly expressed in most tissues of diabetic mice and humans, including liver, adipose tissue, and skeletal muscle. Therefore, the inhibition of SREBP-1c promoter activity and mRNA and protein expression in HepG2 cells would indicate that oregano extract could inhibit hepatic lipogenesis in vitro. The overexpression of cytochrome P450 2E1 (CYP2E1) indicates the presence of oxidative stress caused by excess glucose in the blood [393]. The use of oregano would, therefore, reduce the oxidative damage associated with insulin resistance and changes in glucose metabolism, particularly the activation of the glucose transporter 2 (GLUT2) function [393]. 

In vitro tests showed that the extract promoted glucose uptake, inhibited glycosylation, and relieved oxidative stress, suggesting that oregano leaf extract has a strong hypoglycaemic capacity [393]. In addition, mechanical analysis also showed that the extract decreased promoter activity and mRNA and the protein expression of PEPCK and SREBP-1c. The extract, therefore, inhibited CPY2E1 expression and increased GLUT2 expression [393]. 

Several studies have demonstrated the efficacy of oregano in vivo models (Table 1). In a rat model treated with ALO, the oral administration of an infusion of oregano leaves reduced blood glucose levels after the first day of treatment, compared with the diabetic control group [501]. The results showed that the infusion exhibited hypoglycaemic activity, possibly due to the combined effect of rosmarinic acid and other minor compounds [501]. According to Luis et al. (2022), oregano infusion has an antidiabetic effect by stimulating insulin secretion. In the streptozotocin-treated rat model (Table 3), aqueous leaf extract (20 mg/kg) slightly decreased blood glucose levels in normal rats 6 h after single oral administration and 15 days after repeated daily oral administration [496]. The treatment also caused a significant reduction in blood glucose levels in STZ diabetic rats. Blood glucose levels normalised from the fourth day after treatment. However, this effect was less pronounced two weeks later, and no change was observed in basal plasma insulin concentrations after treatment in normal or STZ diabetic rats, indicating that aqueous oregano extract exhibited a blood glucose lowering activity independently of insulin secretion by pancreatic *β* cells of Langerhans islets [496]. 

Different results were reported in another study by Mohamed and Nassier (2013), who reported that oregano extracts improved glycaemia by enhancing insulin sensitivity. The antihyperglycaemic effect of the same aqueous preparation of oregano leaves at the same dose (20 mg/kg) was tested in STZ-induced diabetic rats. Its oral administration significantly reduced blood glucose levels, glycosylated haemoglobin, and pancreatic amylase in STZ diabetic rats compared with the standard drug, Glibenclamide (0.9 mg/kg BW) [497]. Liver weight/BW ratios were also reduced, while kidney weight/BW ratios, urea, uric acid, and creatinine levels were partially improved. Oral treatment with the extract reduced serum insulin, liver and muscle glycogen levels, and body weight in STZ diabetic rats. This evidence suggests that the modulation of insulin secretion and/or action mechanisms may be involved in oregano’s antidiabetic effect (Mohamed and Nassier, 2013). 

Furthermore, according to Mohamed and Nassier (2013), oregano’s hypoglycaemic effect may be mediated by interference with the absorption of dietary carbohydrates in the small intestine or by stimulation of glucose utilisation by peripheral tissues [497]. The results showed that oregano leaves contain phenolic glucosides that help control blood sugar levels and stimulate the *β*-cells of the pancreas to produce insulin. The reduced HbA1c levels in treated diabetic rats could be due to an improvement in insulin secretion by the remaining pancreatic *β* cells in diabetic rats, resulting in improved glycaemic control [497]. A decrease in liver and muscle glycogen has also been observed in diabetic rats, possibly attributable to insulin insufficiency and the inactivation of the glycogen synthase system in the diabetic. However, after oregano treatment, there was a significant increase in liver and muscle glycogen levels in diabetic rats [497]. According to the authors, the higher liver glycogen levels in treated diabetic rats could be caused by increased insulin levels, which increased glycogenesis and decreased glycogenolysis and gluconeogenesis. Thus, the antihyperglycaemic effect of aqueous oregano extract may protect surviving pancreatic β cells and increase insulin secretion and glycogen storage [497].

In the study by Vujicic et al. (2015), methanolic (ME) and aqueous (AQ) extracts were administered to C57BL/6 mice treated with multiple low doses of STZ for the induction of diabetes (MLDS) (Table 3). According to the study results, prophylactic treatment with the AQ extract had no impact on diabetes induction [394]. On the other hand, ME extract reduced the incidence of diabetes and preserved normal insulin secretion. In addition, it specifically attenuated the pro-inflammatory response mediated by T-helper 17 cells (Th17) [394]. It enhanced anti-inflammatory T helper 2 (Th2) and regulatory T cells by activating specific signalling pathways and transcription factors. Finally, it also preserved *β* cells from apoptosis in vitro by blocking caspase 3. Rosmarinic acid, a predominant compound in the ME extract, was also tested and showed only partial protection against the induction of diabetes [394]. According to the authors, through its antioxidant, immunomodulatory, and anti-apoptotic properties, oregano protected the mice against the development of diabetes. 

In addition, this efficacy could be mediated by the influence of additional chemical compounds [394]. In a comparable efficacy study, the immunomodulatory effect of oregano ethyl acetate extract (EtOAc) was assessed by measuring immune cell proliferation or cytokine secretion [498]. In addition, it was administered intraperitoneally for ten days in male MLDS-treated C57BL/6 mice. EtOAc extract suppressed macrophage and lymphocyte function in vitro. The in vivo oregano treatment significantly preserved pancreatic islets and reduced the incidence of diabetes in mice with MLDS [498]. In addition to the modulatory effect on macrophages, EtOAc reduced the number of total CD4+ T cells and activated CD4+CD25+ T cells. It also affected the number of T helper 1 (Th1) and Th17 cells by downregulating their key transcription factors T-bet and ROR*γ*T (RAR-related orphan receptor gamma). Treatment with EtOAc thus protected the mice against the development of hyperglycaemia by reducing the pro-inflammatory macrophage/Th1/Th17 response and shifting the macrophage population to the protective M2 form [498]. The EtOAc extract is reported to exert a wide range of immunomodulatory effects that appear to be useful for attenuating islet-directed inflammation [498], unlike the methanolic extract of oregano tested by Vujicic et al. (2015), which showed very specific effects on IL-17-producing T lymphocytes [394]. According to Vujicic et al. (2016), the possible interaction of EtOAc with TLR4 (Toll-like receptor 4) function would make this extract a possible candidate for the treatment of immunoinflammatory and autoimmune diseases such as systemic lupus erythematosus, uveitis, inflammatory bowel disease, arthritis, and diabetic nephropathy, which seem to benefit from TLR4 blockade in the preclinical setting [498].

- *Prunella vulgaris*

Around 20 species of *Prunella* are in the world, found in the temperate regions and tropical mountains of Europe and Asia, northwest Africa, and North America [522,523]. They are herbaceous perennials that grow mainly in forests, bare mountains, ridges, and roadsides. In common with other members of the *Prunella* genus, which is currently attracting great interest due to its important new therapeutic application [523,524]. *Prunella vulgaris* L. (PV) has been used worldwide for centuries as an alternative medicine for various illnesses [172]. It is a native plant that grows mainly in the temperate biome, extending from the temperate and subtropical northern hemisphere to Central America [116]. Its dried fruit spikes have been used in folk medicine, particularly in China, for thousands of years to relieve sore throats as an antipyretic and accelerate wound healing (Supplementary Table S3). As a result, it is listed in the Chinese Pharmacopoeia as a commonly used Chinese medicinal material [524,525]. It has also been classified as one of the new Chinese herbal medicines in the list of medicinal and food homologies by the National Health Commission of the People’s Republic of China [524,526].

Several pharmacological and/or clinical studies have demonstrated the efficacy of PV, including its antiviral, antibacterial, anti-inflammatory, immunomodulatory, anti-cancer, antioxidant, hypolipidaemic, anti-tumor, hypotensive, and sedative actions (Table 3). It has great value in terms of application and research into treating hyperglycaemia. Numerous researchers have been attracted to exploring and studying its effects on diabetes (Table 2 and Table 3). 

Fructosamine (FMN) is a non-enzymatic glycation product that can monitor glucose control [527]. This parameter is correlated with blood glucose levels. It reflects the state of glycaemic control over the last 2–3 weeks, and its increase favors the development of diabetes [503]. These observations were observed in STZ-induced diabetic rats in a study by Zhou et al. (2013). However, after six weeks of treatment with 100 mg/kg and 200 mg/kg of PV triterpene acid, blood glucose and FMN levels were significantly reduced in STZ-induced diabetic rats and improved body weight was observed compared with the control group [503]. These data indicate that PV has a dose-dependent antihyperglycaemic effect. Other parameters were also investigated in this study. An increase in nitric oxide (NO) concentration and nitric oxide synthase (NOS) activity was observed in STZ-induced diabetic rats [503]. The increase in oxygen free radicals is linked to the development of hyperglycemia. In the study, the oral administration of Prunella to diabetic rats significantly decreased NO levels and NOS activity. In addition, PV has the effect of increasing the concentration of MDA, serum SOD activity, and total SOD mRNA expression in pancreatic islet *β*-cells, demonstrating that the plant may have an antioxidant capacity to enhance the oxidative stress response to eliminate the increase in free radicals and prevent radical damage [503].

In a study by Raafat et al. (2016), PV’s caffeic acid-rich fraction (CARF) reduced blood glucose levels and improved oxidative stress in vivo [448]. It inhibited alpha-amylase and alpha-glucosidase enzymes and reduced HbA1c levels more significantly than hydroethanolic extract and equivalent amounts of caffeic acid (CA) or rosmarinic acid (RA) [448], indicating that CARF provides continuing glycemic restoration in diabetic animals. For longer durations, it significantly increased serum insulin and improved thermal hyperalgesia and tactile allodynia more significantly than the effects of hydroethanolic extract and equivalent amounts of caffeic acid or rosmarinic acid. In addition, the compounds tested showed potential for restoring lipid peroxide levels. As a result, CARF and the hydroethanolic extract observed an increase in serum insulin, the attenuation of alpha-amylase and alpha-glucosidase, and their antioxidant potential could be responsible for their antidiabetogenic and antinociceptive properties. RP-HPLC and ^1^H and ^13^C NMR experiments showed that CARF isolated from PV contained CA (93.4%) and RA (6.6%). According to the authors, the acute and subchronic hypoglycemic activity of CARF was 1.27 and 2.72 folds more effective than Glibenclamide’s, respectively. Subchronically, it has significantly improved body weight, indicating its long-term efficacy in T1D (Type 1 Diabetes) amelioration [448].

According to existing research, diabetic patients are at greater risk of developing atherosclerosis [528,529]. Diabetes mellitus, in both its forms, is an independent risk factor for the accelerated development of atherosclerosis [530]. Diabetes and atherosclerosis appear to be linked by multiple pathogenic mechanisms [528]. Dyslipidemia with high levels of atherogenic LDL, hyperglycaemia, oxidative stress, and increased inflammation are possible explanations for this acceleration [530]. *Prunella vulgaris* can improve impaired insulin secretion, vascular dysfunction, and metabolic abnormalities and markedly attenuate hyperglycaemia and vascular inflammatory processes in db/db T2DM mice. The study by Hwang et al. (2012) was conducted to determine whether *Prunella vulgaris* could inhibit diabetic atherosclerosis in db/db mice fed a high-fat, high-cholesterol (HFHC) diet [502]. This eight-week regimen increased body weight, blood glucose, and insulin levels to study the relationship between hyperglycaemia, which may also contribute to atherogenesis in db/db mice and the efficacy of PV in treating this dysfunction [502]. According to the authors, treatment with an aqueous extract (100 and 200 mg/kg/day) reduced glycaemia and systolic blood pressure. A clear improvement in renal function markers such as creatinine clearance and blood urea nitrogen was also observed. These results suggest the possible beneficial effects of using PV in treating nephropathy in T2DM [502]. The groups treated with the plant also had a reduction in glucose and lipid levels and body weight, even though food intake did not change. According to the authors, the improvement in diabetic complications by PV is independent of diet. It involves energy consumption and additional factors outside the scope of insulin resistance and hyperlipidaemia and is necessary to mediate the effects of vascular complications on HFHC-db/db mice. 

In Hwang et al. (2012) study, PV’s antioxidant effect also improved diabetic atherosclerosis [502]. Chronic plant treatment of db/db mice significantly reduced malondialdehyde levels, a naturally occurring reactive species marker of oxidative stress. By improving lipid levels and attenuating oxidative stress, PV could be one of the potential therapeutic strategies for the early management and prevention of coronary heart disease [502]. The NO levels were increased by PV treatment, suggesting an association with vascular dysfunction. The vascular relaxation of the aortic rings induced by acetylcholine or SNP (sodium nitroprusside) was improved by PV in a dose-dependent manner. In addition, PV ameliorated the dysfunction associated with vascular intimal injury and media hypertrophy observed in db/db mice. The aortic expression of ICAM-1 (the intercellular adhesion molecule-1), VCAM-1 (vascular cell adhesion protein 1), ET-1 (endothelin 1), and nitrotyrosine was significantly reduced by PV. In addition, eNOS expression in the aorta was remarkably increased by PV treatment [502].

PV also has a significant protective effect against diabetic renal dysfunction, including inflammation and fibrosis, by disrupting TGF-*β* (transforming growth factor-beta)/Smad signalling [505]. In human mesangial cells (HMCs), PV pre-treatment attenuated the suppression of TGF-*β* and Smad-2/4 expression induced by 25 mM high glucose and increased the level of Smad-7 expression. PV significantly reduced connective tissue growth factor (CTGF) and collagen IV, fibrosis biomarkers [505]. In addition, it suppressed inflammatory factors such as intracellular cell adhesion molecule-1 (ICAM-1) and monocyte chemoattractant protein-1 (MCP-1). PV inhibited the activation and translocation of NF-κB in high glucose-stimulated HMCs. In addition, PV significantly reduced high glucose-induced ROS dose-dependently [505].

The aim of a study by Jiao et al. (2021) was to investigate the active ingredients, potential targets, and signalling pathways of PV and to explore the ‘multi-target, multi-pathway’ molecular mechanism of *Prunella vulgaris* L. on diabetes mellitus complicated by hypertension (DH) [158]. This work was based on network pharmacology and molecular docking analyses. According to the analysis results, 11 active compounds, 41 key targets, and 16 significant signalling pathways were identified from *P. vulgaris*. The main active components of PV against DH that were discovered were beta-sitosterol, kaempferol, spinasterol, stigmasterol, delphinidin, luteolin, vulgaxanthin-I, poriferasterol monoglucoside_qt, stigmast-7-enol, morin, and quercetin [158]. The potential action targets identified are diverse, including IL-6 and INS (Insulin), MAPK3, ALB (Albumin), AKT1, TNF, etc. The most active signalling pathways are AGE-RAGE (advanced glycation endproducts/receptor for AGE), the TNF signalling pathway, the MAPK signalling pathway, the PI3K-AKT signalling pathway, etc. PV is involved in biological processes such as cell proliferation, apoptosis, and inflammatory response [158]. According to the authors, the main molecular mechanism of *P. vulgaris* against DH is via sterols and flavonoids, which play an active role in affecting the TNF signalling pathway, the AGE-RAGE signalling pathway, the MAPK pathway, the PI3K-Akt pathway, and targets linked to the IL-6 and INS pathways [158].

## 4. Conclusions and Perspectives

In this review, we have described the botanical diversity of the NPSE. We have tried to investigate the traditional, therapeutic, and antidiabetic uses, and the chemical composition, of the various plants found there. It was noted that there are few or no existing studies on medicinal plants in this region. Therefore, we have tried to collect all the relevant information concerning worldwide traditional and modern medicine in medicinal plants of the NPSE to enhance their value and explore their therapeutic and chemical potential for possible application in preventing and treating diabetes. It has been noted that several plants used in traditional medicine have not yet been tested for their antidiabetic effects. Therefore, further research is needed on these medicinal plants’ in vitro and in vivo antidiabetic effects. Some of them have been tested against diabetes without having had any subsequent traditional use. We found that the two families, *Asteraceae* and *Lamiaceae*, are the two plant groups most present in the Park with the highest number of citations of traditional use against diabetes. Studies of the hypoglycemic potential of members of these two families have revealed numerous remarkable properties against diabetes. Several mechanisms of action have been implicated. However, further research, particularly of a clinical nature, is required. Phytochemical characterisation has shown that these medicinal plants contain numerous bioactive compounds from different chemical groups. Pharmacological studies of these remedies have shown that they have interesting therapeutic effects. Given this information, we need to study the possibilities for successfully integrating the medicinal plants of the NPSE into a public health framework. Studies should be conducted to develop new antidiabetic drugs based specifically on medicinal plants from the Park and their respective bioactive compounds.

## Supplementary Materials

The following supporting information can be downloaded at: https://www.mdpi.com/article/10.3390/pharmaceutics16040454/s1, Table S1: Description of botanical data and search methodology for Serra da Estrela Natural Park plants in the Flora-on database; Table S2: Analysis of the plant number with anti-diabetic potential in Serra da Estrela Natural Park; Table S3. Traditional uses of NPSE medicinal plants with antidiabetic potential; Table S4: Species of plants in Serra da Estrela Natural Park reported to have anti-diabetic potential.
