# Supplementary material for: Overview of Ethnobotanical–Pharmacological Studies Carried Out on Medicinal Plants from the Serra da Estrela Natural Park: Focus on Their Antidiabetic Potential
Source: Pharmaceutics. 2024 Mar 25;16(4):454. doi: 10.3390/pharmaceutics16040454 (PMC11054966; doi:10.3390/pharmaceutics16040454)
Supplement: Supplementary file 1 [file pharmaceutics-16-00454-s001.zip › Suplementery Table S3.pdf]

Supplementary Table S3. Traditional uses of Serra da Estrela Natural Park medicinal plants with antidiabetic potential.

Plant	**Common Name (EN, POR)**	**Flowering period**	**Part used**	**Preparation**	**Traditional uses**	**References**
					Asteraceae	
Arctium minus (Hill) Bernh	*Lesser burdock. *Teijeira.	Jun - Aug	NI	NI	Antidiabetic potential.	[1–3]
			L/R	NI	Treat rheumatic pains, fever, sunstroke, wounds, general infections, skin and body inflammations, alopecia, and bladder diseases.	[4–6]
			L	VIM	For sunstroke or colds.	[7]
			R/L	INF	Snake and scorpion bites to purify the blood, stimulate the appetite and liver functions	[8]
Achillea millefolium	*Common yarrow. *Milefólio.	May - Aug	NI	NI	Antidiabetic potential.	[9,10]
					Treatment of inflammatory, spasmodic gastrointestinal disorders, hepatobiliary complaints, and overactive cardiovascular and respiratory ailments.	[11,12]
					Astringent, antiseptic agent, menstrual irregularity, relieving menstrual cramps and pain, anti-spasmodic agent.	[13]
Anthemis canescens var *aurea* (syn. *Matricaria aurea*)	*Golden Mayweed, corn chamomile. *Macela-galega.	Mar – Jul	-	-	ANI	-
			F	NI	Used to treat digestive system problems (colics, haemorrhoids, constipation, anti-spasmodic), cosmetics, skin cracks, urinary tract infections, and cough.	[14]
	*Wolf's bane.	Apr-Jun	NI	NI	Diabetic neuropathy.	[15]

Arnica montana subsp. *atlantica*	*Panaceia-das-quedas.		R/F	MOA/OIT/GE/CRE	Applied externally on the bruises, rheumatic pains, sprains, and phlebitis to reduce the inflammations and as an immune system activator.	[16–19]
			F	DFE	Modified form of tinctures, ointments, creams, and gels as an immediate treatment of sprains, bruises, contusions, and trauma pain in homoeopathy medicine.	[20]
Bellis perennis	*English daisy. *Bonina.	Jan - Sep	-	-	ANI.	-
			F	DEC	Carminative, diarrhoea, diuretic, purgative.	[21]
			NI	NI	Treatment of bruises, broken bones, and wounds. Used in treating sore throat, headache, common cold, stomachache, eye diseases, eczema, skin boils, gastritis, enteritis, diarrhoea, bleeding, rheumatism, inflammation, and upper respiratory tract infections in traditional medicine. Its astringent, expectorant, diuretic, booster, purgative and diaphoretic properties have been recorded.	[22–25]
Bidens frondosa	*Devil's beggarticks. *Erva-rapa.	Jul - Sep	-	-	ANI	-
			SE	NI	Asthma, bronchitis, colds, influenza.	[26]
Calendula arvensis	*Field marigold. *Erva-vaqueira.	Dec - May	F/L/S	INF/DEC	Antidiabetic potential.	[27–29]
			FH	DEC	Wounds, contusions, burns	[30]
			FH/L	TE	Antiseptic, astringent.	
			L	EXU	On sores and wounds.	
			IF	NI	Antiecchymotic.	[31]
	*Roman chamomile	Apr-Sep	NI	NI	Antidiabetic potential.	[32]

Chamaemelum nobile (syn. *Anthemis nobilis* L. or *Chamomilla nobilis* (L.) Godr.)	*Camomila-de-Paris		F	NI	Tonic, analgesic, anti-spasmodic, diuretic, treatment of indigestion, gout, headache, external swelling, inflammatory pain or congested neuralgia.	[33]
			FH	INF	Anti-inflammatory, Sedative, eczema.	[34]
			NI	NI	Analgesic, menstruation balance and pain, appetite, weight loss, cardiovascular diseases, respiratory diseases, immune system diseases, anti-spasmodic, and headache.	[35]
Cichorium intybus	*Common chicory.<br>*Almeirão.	Jun-Sep	F/L/R/AP	DEC/INF/SFL	Antidiabetic potential.	[36]
			F/L/R	DEC/INF	Cleaning the liver, regulating bladder function and purifying the human body.	[36]
			WP	NI	It's used for dyspepsia, loss of appetite, and adverse chronotropic and inotropic effects.	[37,38]
			R/WP	NI	Used to clear away heat, eliminate dampness, and promote digestion.	[39]
			L/R	DEC/TE/OIT	Wound healing, kidney stones, cancer, haemorrhoids and eczema.	[7]
Dittrichia viscosa subsp. *Viscosa* (Syn. *Inula viscosa*)	*False yellowhead<br>*No	Aug - Oct	L	DEC	Antidiabetic potential.	[40]
			AP/R	DEC/FUM	Treatment of skin irritations of allergic origin. Used for bleeding and psoriasis.	[30]
			R	NI	Used against cough, catarrh, antiseptic, expectorant agent and supports mucus membranes.	[41]
			NI	CATA	Antihelmintic, diuretic, expectorant and anti-anemic. Used for rheumatic pain, tuberculosis, and bronchitis treatment.	[42]
			L/R	DEC	Treat hypertension.	[43]

			WP/FH/L	DEC/CATA	Treat cancer, antihelmintic, muscle relaxant, expectorant, diuretic, bronchitis, tuberculosis, anaemia, and rheumatic pain.	[44–47]
			L	NI	Muscle relaxation and to treat infertility.	[48]
*Galinsoga parviflora (*Syn. *Galinsoga quadriradiata)*	*Gallant soldier. *Pacunga Pequena.	May - Oct	L/R	TE/POUL/GAR	Antidiabetic potential.	[49]
			WP	NI	Treatment of wounds, cold, flu, cold sores, halt bleeding, as fodder for cattle, body injuries, yellow fever, hepatic pain, painful joints, fever and internal inflammation, potherb, earache, skin diseases, and scorpion bite.	[50–54]
			L	NI	Treatment of diarrhoea, fever, snake bite, haemorrhages, eaten as a vegetable, malaria, anaemia-with jaundice, to detach fetal placenta, colorectal cancer, analgesic, liver problem, toothache.	[50,55–58]
			AP	NI	Mitigating lichens, acne, wounds, eye problems, eczema, and rosacea protects against UV irradiation-induced damage, fever, toothache, inflammation, liver problems, and insect bites.	[59–63]
			F	NI	Treatment of toothache and enhanced memory.	[50,64]
			R	NI	Treatment of beetle bites.	[64]
			S/L	NI	Treatment of colds and sores.	[59]
Helichrysum stoechas subsp. *stoechas*	*Mediterranean strawflower, Everlasting flower *Marcenilha	Apr-Sep	-	-	ANI.	-
			AP	NI	Treat disorders of the cardiovascular, digestive (bloating, hepatic and pancreatic problems) and respiratory systems (influenza and cold) and have anti-urolithiasis and diuretic effects.	[65–68]
	*Hairy cat's ear.	Apr - Nov	NI	NI	Antidiabetic potential.	[69]

Hypochaeris radicata	*Erva-das-tetas.		NI	NI	Treatment of inflammation, jaundice, rheumatism, dyspepsia, constipation and kidney-related problems.	[65,70,71]
Lactuca serriola	*Prickly lettuce.<br>*Alface-brava.	Jun - Aug	-	-	ANI.	-
			WP	NI	Sedative–hypnotic, anxiolytic, expectorant, cough suppressant, purgative, demulcent, diuretic, antiseptic, vasorelaxant, and anti-spasmodic. Thus, it is used to manage bronchitis, phthisis, asthma, pertussis, gastrointestinal, sedative, diaphoretic, anti-spasmodic, against hypercholesterolemia, for memory Enhancing and ophthalmic purposes.	[72–78]
			SA	NI	It contains '*lactucarium*', used for anti-spasmodic and narcotic purposes. It is also used to treat insomnia, anxiety, neuroses, and hyperactivity in children.	[79]
Onopordum acanthium subsp. *Acanthium*	*Scotch thistle.<br>*Acanto-Bastardo.	Jun-Sep	-	-	ANI.	-
			NI	NI	Treatment of different cancer types stimulates the central nervous system and has cardiotonic and haemostatic properties.	[80,81]
			AP	Jui/DEC/Pow	Diuretic and treat nervousness.	[80,81]
			AP	DEC/BTH/Pow/Jui/INF	Expectorant, anti-asthmatic, and diuretic. Used to treat nervousness, tetanus, and carcinomas. For gonorrhoea, dermatosis ulcers, and tuberculosis cutis. The plant is also effective in rehabilitation (after cancer surgery) in malignant tumours, skin cancer, scrofula, and purulent skin diseases. Useful in diseases of the gallbladder, respiratory infections, and hemostasis. It is	[82]

					helpful to the diseases of the kidneys and the bladder.	
			L	FRE/Jui	Skin cancer, freckles, ulcerations, lupus, suppurative wounds.	[83]
			F	DEC	Haemorrhoids, hepatic diseases.	[84]
			NI	NI	Appetiser, diuretic, stomachic, carminative, resolutive.	[85]
			R	CATA	Antirheumatic menstruation.	[86]
			SE	NI	Antibacterial, anti-inflammatory, antimalarial and hypertensive agent.	[87,88]
Senecio vulgaris	*Groundsel, old-man-in-the-spring. *Tasneirinha.	May-Oct	-	-	ANI.	-
			R	Jui	Eczema.	[89]
			WP	INF	Astringent and diuretic.	[90]
			NI	NI	Emmenagogue, used in menstruation problems, inflammatory conditions such as gout, arthritis, thrombophlebitis, skin wounds (bruises, fungal infections, ulcers, fractures, strains), varicose veins, oral lesions (gargle), bronchitis, allergies, gastritis, gastroduodenal ulcers, diarrhoea bronchitis, asthma, and cough; urinary and pulmonary complaints, blood purification, for rheumatism and skin irritations.	[91,92]
Solidago virgaurea	*European goldenrod, Woundwort. *NI	Jun-Sep	AP	INF/DEC	Antidiabetic potential.	[93,94]
					Kidney disorders (being often found in teas intended to help pass kidney calculi), urinary tract infections, overactive bladder syndrome, and prostatic diseases. Healing and antiseptic properties, allergies, and gastrointestinal disorders. Antibacterial and anti-inflammatory	[93–99]

					effects, including inflammation of the oral cavity and throat, when used as a mouth rinse.	
Sonchus asper	*Prickly Sowthistle. *Serralha-Áspera.	Jul-Oct	L/R	DEC	Antidiabetic potential.	[100,101]
			AP	DEC	The plant decoction is cathartic; it is used to treat ascites and hydrothorax.	[101]
			WP	DEC	Hyperthyroidism.	[102]
Sonchus oleraceus	*Common sowthistle. *Serralha.	Jan-Jun	L	DEC	Antidiabetic potential.	[103]
			R	DEC	Treat ear, nose and throat (ENT) diseases.	[104]
			SAP	NI	Treat tonsils.	[105]
			L	FRE	Digestive, and treat gastritis.	[106]
			AP	DEC	Diuretic, laxative.	
Tanacetum parthenium	*Feverfew. *NI.	Jun - Aug	-	-	ANI.	-
			L/F	INF	Vaginal discharge, stomachache.	[107]
				DEC	Bronchitis, common colds.	[108]
			AP	PAS	Feverish horses.	[109]
			WP/F	DEC	Used to alleviate menstrual pain and treat skin ulcers and contusions.	[110]
			L		Gastric ailments, sedatives, fever, and nerve system relaxants.	[111]
			L/St	NI	Analgesic.	[112]
			AP/L	INF	Abortifacient.	[113]
			FAP		Antiseptic in cows after calving.	[114]
			F		Stress-related disorders, sedative.	[115]
			NI	NI	Ulcerative colitis.	[116]
Tanacetum vulgare	*Tansy. *Atanásia.	Jul - Aug	F/L	INF/DEC	Antidiabetic potential.	[117–120]
				INF	Antihelminthic, carminative, anti-spasmodic, abdominal organ stimulant, tonic, menstruation	[120]

					stimulant, diuretic, and anti-hypertensive properties.	
			Poaceae			
Avena sativa	*Oat, Groats. *Aveia-comum.	May - Jul	-	-	ANI.	-
			WP	NI	Treat nervous exhaustion, insomnia, and weakness of the nerves. antispasmodic, antitumor, cyanogenetic, demulcent, diuretic, neurotonic, stimulant, tonic, and vulnerary. Useful in rheumatic conditions and to treat water retention.	[121]
				WB	Treat insomnia, anxiety, and various skin conditions, including burns and eczema.	
			GTO	TINC	Nerve stimulant and help in withdrawal from tobacco and opium addiction.	
			S	NI	Remedies for tumours.	
			STR	NI	Useful in rheumatism.	
			Fabaceae			
Acacia dealbata	*Silver wattle *Acácia-dealbada	Jan - Mar	-	-	ANI	-
			POL	Pow	Beauty.	[122–124]
			BAR/R	BOI	Treat stomachaches, chest pains, and eye problems.	[125]
			GU	NI	Bronchial troubles.	[126]
Lupinus angustifolius	*Blue lupin. *Tremoção-bravo.	Mar - May	-	-	ANI	-
			WP	DEC	Skin Related Problems, treat digestive system disorders	[127,128]
		Mar - Jul	-	-	ANI	-

Lupinus luteus	*Annual yellow lupin. *Tremoceiro-amarelo.		SE	NI	Anthelmintic, Emmenagogue, Carminative, liver disorders.	[129,130]
Pisum sativum	*Common garden pea. *Ervilheira-brava.	May - Jul	PD	NI	Antidiabetic potential.	[131]
			EAP/SE	CATA	Skin (impetigo), cataplasm.	[132]
			VAL	TIS	Intestinal anti-inflammatory and laxative.	[114]
			SE	FOO	Laxative, poisoning, paralysis, muscle weakness.	[133]
Pterospartum tridentatum (Syn. *Chamaespartium tridentatum* or *Genista tridentata*)	*NI. *Carqueija.	Mar - Jun	F	NI	Antidiabetic potential.	[134]
			FAP/Sho/BD	INF/DEC/OIT	Used for upper respiratory infection acute, strep throat asthma, gastroenteritis and colitis of unspecified origin, cutaneous abscess, furuncle and carbuncle, abdominal and pelvic pain, functional dyspepsia, urinary retention, cystitis, malaria.	[135]
			F	DEC	Cold, depurative, Influenza, Coug, malaise, stomach troubles.	[136]
					Tonic; diuretic; digestion; used for influenza and cold; nervousness, anti-asthenia; anti-inflammatory; anti-influenza; anxiolytic; carminative; diuretic; stomachic and for gases.	[137]
			AP	NI	Condiment, fuel, and animal feed.	[138]
Retama sphaerocarpa	*Broom bushes. *Piorneira.	Apr - Jul	R	DEC	Antidiabetic potential.	[139]
			CL	NI	Emetic, purgative, vermifuge, joint aches, fever, luxation.	[140–142]
			CL/F	NI	Rheumatism, warts, healing.	[141]
			R	NI	Diphtheria.	[143]
			NI	NI	To cure rabies.	[144]
			Fr	NI	Diarrhoea, discolouration, pain.	[141,142]

			F	NI	Liver disease, healing wounds skin.	[141,142]
Robinia pseudoacacia	*Black locust. *Acácia-bastarda.	Apr - Jul	-	-	ANI	-
			F	INF	Sedative, expectoration, cold, pulmonary diseases, against joint pains and rheumatisms.	[145–147]
			BA/L	CHE	Toothache boils.	[148]
Trifolium pratense subsp. *pretence*	*Red clover. *Pé-de-lebre.	May-Oct	FOF	BOI	Antidiabetic potential.	[149]
			WP	NI	Treats menopausal symptoms and is an anti-ageing agent. Treat high cholesterol, premenstrual syndrome, breast pain, cancer, heart problems, inflammatory diseases, epilepsy, high cholesterol, sexually transmitted diseases, dysmenorrhea, mastosis, and mastalgia.	[150–155]
Trifolium repens	*White clover. *Trevo branco.	Mar - Jun	-	-	ANI	-
			AP	DEC/POUL	Used as expectorant and diuretic.	[156]
			F	NI	For blood purification and mouth inflammations.	[157]
			BA	TE	Tea for cervical wounds and increased vaginal secretion.	[158]
				Caryophyllaceae		
Corrigiola litoralis	*Strapwort. *Correjola.	Jul - Oct	R	Pow	Antidiabetic potential.	[118,159]
			WP	DEC	Diuretic.	[160,161]
			R	DEC	Acute ache; digestion problems.	[162]
Corrigiola telephiifolia	*Strapwort. *NI.	Mar - Oct	R	BRE	Antidiabetic potential.	[163]
				DEC/INF	Diuretic and used to treat anaemia, lung disorders and for hair care.	[164–166]
			WP/L	DEC	Rheumatic diseases, cancer.	[29]
Paronychia argentea	*Silver nailroot. *Erva-dos-linheiros.	Jun – Sep	L/S	INF	Antidiabetic potential.	[167]
			L/F	INF	Stones in kidney, diuretic, nephropathy, anti-obesity.	[168,169]

Saponaria officinalis	*Common soapwort. *Erva-saboeira.	Jun - Sep	R	DEC	Antidiabetic potential.	[170]
			R	DEC	Used as emmenagogue detergent. Exudative diathesis is caused by metabolic disorder, metabolism improvement and diuretics.	[171,172] [170]
Spergularia rubra	*Red Sand Spurrey. *Arenária.	Mar - Sep	-	-	ANI.	-
			WP	INF	Abdominal and pelvic pain.	[135]
			L/S	DEC	Renal lithiasis.	[173]
			WP	DEC	Used to treat genitourinary diseases.	[132]
			AP	NI	Antiseptic for treating diseases related to the renal system.	[174,175]
Stellaria media	*Common chickweed. *Erva-canária.	Jan - Oct	WP	TE	Antidiabetic potential.	[176,177]
				NI	Is used as astringent, carminative, anti-asthmatic, demulcent, depurative, diuretic, emmenagogue, expectorant, galactogogue, anti-obesity, useful in kidney complications, rheumatic joints, wounds, and ulcers. It is also used in various kinds of skin diseases, for bronchitis, rheumatic pains and dysmenorrhoea. It can be applied to cuts, burns, and bruises.	[178]
				CATA	Rheumatism, sedative, Eczema, Fractures.	[133]
				PLS	Used for dislocated bones and swelling, cooling, astringent, vulnerary.	[179]
				NI	Plant is very effective for menstrual distress.	[180]
				EXT	Anti-pyretic, cures chest congestion, cough and cold. It is also used as antacid and anti-spasmodic.	[181]
			SE	NI	Wounds healing.	[182]
				Brassicaceae		
	*Shepherd's purse.	Jan - Jul	AP	INF	Antidiabetic potential.	[170]

Capsella bursa-pastoris	*Bolsa-de-pastor.				Haemostatic.	[183]
					Diuretic, Nosebleed.	[184]
			F/SE	INF	Used as diuretic, against urinary diseases, and kidney stones.	[185]
Raphanus raphanistrum subsp. *raphanistrum*	*Cabrestos. *Wild radish.	Apr - Nov	Rhi/S	NI	Antidiabetic potential.	[186]
			AP/S	Raw/BOI	Liver protector, antirheumatic, cholagogue, choleretic and depurative activities, gastric stimulant, purgative or antidiarrhoeic remedies.	[187–189]
			R/Rhi	NI	Health tonic and gastrostimulant.	[190,191]
			Fr	NI	Used for the treatment of cancer.	[192]
			L	BOI	Carminative.	[193]
			Rhi/S	NI	Intestinal disorders, Epigastric pain.	[186]
					Apiaceae	
Daucus carota	*Wild carrot. *Cenoura-brava.	Apr - Aug	WP	NI	Antidiabetic potential.	[194]
			R	NI	For digestion, carminative, dysentery, antidiarrhoeic, coughs, strong urinary remedy, diuretic, preventing stone, ridding gravel, against cystitis. For related conditions of gout, rheumatic and arthritic complaints, and easing oedema. Aphrodisiac, a contraceptive, provokes menses and tonic for uterine function.	[195–199]
			L/R	NI	For cough, stomachache, eye medicine, wrinkles, wounds, chilblain, burns, dermatitis, aphthous stomatitis, ulcerations, cracks, breast fissure, furuncle, abscess, impetigo, tinea, inflammations, wrinkles, senile keratosis.	[200]
Eryngium campestre	*Field eryngo. *Cardo-corredor.	Jun - Sep	-	-	ANI	-
			R	FOO	Bitter tonic, aperitive, cholagogue, diuretic.	[199]

			L/YB	FOO	Laxative, aperitif.	[201,202]
			R/S	BOI	Aphrodisiac.	[193]
			R	NI	Diuretic, febrifuge, digestive, flatulence, hepatitis, muscle pain, palliative care of general and cancer patients.	[203–207]
Foeniculum vulgare	*Common fennel. *Erva-doce.	Jul - Aug	S/AP	DEC/INF	Antidiabetic potential.	[119,208]
			WP	DEC//INF/DRI/CATA/INH/Pas/Pow/PIL/TAG/EYD/RW	Dermatological diseases (urticaria/hives, Hair problems, eczema), respiratory-nose, ear, oral/dental, throat problems, internal medical diseases (hypertension, fever, cancer, abdominal pains, colic, nausea and vomiting, tachycardia, anaemia, obesity, dizziness, hiccup, anorexia, headache, dysphagia.), urogenital, obstetric, and gynaecological (leucorrhea, kidney diseases, diuretic, amenorrhea and oligomenorrhoea, galactagogue, problem of repeated abortions, kidney stones, dysmenorrhea, urinary calculi), eye diseases (irritation and conjunctivitis), musculoskeletal system and connective tissue disorders (arthritis, rheumatism, neck pain, backache, low back pain), infectious and parasitic disease (Dysentery, Rabies), epilepsy, motion sickness, insomnia, refreshing, hypnotic, appetiser, mosquitocidal, sedative for children, fat reduction, anxiety, and poisoning.	[209]
Heracleum sphondylium	*Hogweed. *Branca-ursina.	Jun - Aug	AP	NI	Antidiabetic potential.	[210]
			AP/L/R	INF	Digestive, odontalgic and aperitif.	[211,212]
			L/R	CATA	Rheumatoid arthritis.	[213]
			AP	TINC	Anti-depressives.	[214]

					Lamiaceae	
Acinos alpinus (Syn. *Clinopodium alpinum)*	*Alpine rock thyme. *NI.	May - Jul	L	DEC	Antidiabetic potential.	[215,216]
			FAP	DRI	Digestive, genito-urinary, analgesic, appetiser.	[217]
Clinopodium nepeta subsp. *Spruneri (*Syn. *Calamintha officinalis* Moench)	*Calamint. *Erva-das-azeitonas ou nêveda.	Apr - Dec.	SE/L/S	DEC	Antidiabetic potential.	[40,215,218]
			L	NI	Against stomachache.	[219]
			L	DEC/INF	Flu, cold, against different aches, antipyretic.	[220,221]
			F	NI	To cure insect and snake bites, cicatrising.	[222]
			NI	NI	To cure jaundice, dropsy, splenomegaly.	[222]
Lavandula pedunculata subsp. *pedunculata*	*French Lavender. *Rasmonino.	Mar - Sep	-	-	ANI.	-
			L	DEC	Treat respiratory diseases.	[132]
			FB	INF	Used against influenza.	[223]
Lavandula stoechas	*Spanish Lavender. *Rosmaninho.	Fev - Jul	L	INF/EO	Antidiabetic potential.	[224]
			WP	NI	For epilepsy.	[225]
			L	DEC	Antiseptic, antispasmodic, diuretic, digestive system stimulant.	[226,227]
			L	CATA	Against dislocations, wounds, sores, and ulcers.	[226]
			AP	INF	Analgesic Teeth, Rheumatism, system digestive, cystitis and nephritis. For heartburn, seasickness, blood circulation and sedatives.	[227]
			L	INF/EO	Menstrual pains, kidney stones, carbuncles, otitis and hypertension. anticonvulsant, sedative, antispasmodic.	
				FAP	As herbal tea and for making liqueur.	

				FB	Expectorant, menstrual regulari, antispasmodic and carminative.	
Melissa officinalis	*Lemon balm or honey balm.<br>*Citronela-pequena.	Jun - Aug	-	-	ANI.	-
			NI	NI	Possess sedative/tranquilising, anti-gas, fever-reducing, antibacterial, spasmolytic, hypotensive, memory-enhancing, menstrual-inducing, and thyroid-related effects; antiviral and antioxidant activities; antifungal, antiparasitic, and antispasmolytic activities; flatulence; asthma; Bronchitis; amenorrhea; cardiac failure; arrhythmias; ulcers; and wounds. It effectively treats headaches, indigestion, colic, nausea, nervousness, anaemia, vertigo, syncope, malaise, insomnia, epilepsy, depression, psychosis, and hysteria.	[228]
			F	INF	With *Hypericum perforatum*: Clean the kidney, insomnia, relaxant, and sadness.	[229]
			AP	INF	Alone or with *Hypericum perforatum* to treat bronchitis.	
			AP	INF	It is used against influenza and colds, has a calming effect, stomach disorders, painful and irregular menstruation, sleep improvement, and has a positive effect on the nervous system.	[230,231]
			WP	DEC	Antispasmodic, choleretic, stimulating and tonic.	[232,233]
			L	Jui	Human and domestic animal nutrition and veterinary medicine use.	[231]
			L	FRE	They are used for bee swarming.	
			L/S	MAC	Attracts bees.	[234]

Mentha aquatica	*Water mint. *Hortelã-da-água.	Jul - Oct	NI	NI	Antidiabetic potential.	[235]
			WP	DEC	It is used to treat digestive problems and cough. The herb is carminative and is used for flatulence.	[236]
			L/S/SE	INF/TE	Hypertension.	[237,238]
			AP/L	OI	Treatment of oligomenorrhea and amenorrhea.	[239]
			AP	RW	Anxiety disorders and hypochondria.	[240]
Mentha pulegium	*Pennyroyal. *Poêjo.	Jun - Aug	L/S	DEC/INF	Antidiabetic potential.	[241–245]
			AP	DEC/INF	Respiratory diseases, gastrointestinal disorders, hypertension, cardiac diseases, rheumatism, migraine, infertility, pains, fever, obesity, nervous disorders.	[241–244,246]
			L	EO	Gastrointestinal disorders (carminative), musculoskeletal Illnesses (anti-spasmodic).	[247]
			L	DEC	Respiratory diseases, gastrointestinal disorders, gynaecological and endocrinological injuries.	[248]
			L/F/WP	DEC/INF/MAC	Respiratory disorders, gastrointestinal disorders, genito-urinary diseases, infectious and parasitic diseases, haemorrhoids, and undefined symptoms (headache, flatulence).	[244,249,250]
			L/S	INF/EO	Respiratory disorders, skin pathologies.	[251]
			AP/L	CATA/DEC	Hematomas, wounds, swelling, digestive, refreshing, liver disorders.	[252]
			AP	INF/SYR	Antihypercholesterolaemic, gastric analgesic, seasickness, for headaches, for colds, intestinal analgesic, hepatic protector, anti-tussive for colds.	[253]
			AP	DEC	Heart stimulant, antiemetic, anti-convulsive, calmative, dizziness, migraine.	[254]

			WP	INF	Digestif, antidiarrhoeic, diuretic, slimming, anti-catarrhal agent.	[255]
			L	INF/CATA	Asthma, wheezing, cough, cold and flu.	[256]
Mentha suaveolens	*Apple Mint. *Hortelã-brava, montastre, Mentastro.	Jul - Oct	L	INF	Antidiabetic potential.	[257]
				DEC/INH/EAP	Respiratory diseases, gastrointestinal disorders, musculoskeletal illnesses, otolaryngological and pediatric illnesses.	[248]
			L/WP	DEC/INH/EAP	Digestive disorders, allergies.	[250]
			WP	NI	Common cold, cough, anti-convulsive, influenza, respiratory ailments, rheumatisms, skin diseases and irritations.	[258]
			L	DEC	Antirheumatic, antispasmodic.	[259]
				INF/MAC	Respiratory inflammations, local inflammations.	[256]
				LC	Mosquito and bee repellent.	[234]
Origanum vulgare	Wild marjoram. Manjerona-brava, Oregão.	Jun-Seb	AP/L/S	INF/DEC	Antidiabetic potential.	[260,261]
			AP	DEC/INF	Expectorant, antiseptic, digestive aid and anti-spasmodic properties	
				TE	Pulmonary and stomach diseases, colds, kidney and urinary infections, improving appetite, sedative, tonic.	
				INF	Immune system strengthening, digestive, hypolipemic, anti-tussive, stimulates gastric secretion, choleretic, cholagogue, aperitive, digestive. mild antiseptic, spasmolytic, expectorant, in case of inflamed lungs. Anti-catarrhal; to reduce pain after childbirth. Bloating, calmative, Common cold, appetiser, stimulant.	

				DEC	Cold, flu, abdominal pain, kidney stones and stomach diseases.	
			FAP	TE	Stomachache, digestive, vaginitis, panacea	
			F	INF	Stomachic, diuretic, digestive, mouth and teeth disease, dermal respiratory, neurological, vulnerary, hypertension, epilepsy, headache, asthma, cold.	
Prunella vulgaris	*Common self-heal. *Brunéla-	Mar - Aug	NI	NI	Antidiabetic potential.	[262–264]
					Used as a remedy for relieving sore throat, reducing fever and accelerating wound healing. Rheumatism, colds, diuretics and heart disease. It treats sore throat, bleeding, pneumonia, headaches, dysentery, haemorrhoids, diabetes and eye inflammation.	[265–268]
				INF	Hypertension.	[262]
				NI	Reduce fever. It was also employed in folk medicine as an antipyretic. Treatment of Endometriosis.	[269]
			FCF	NI	Improve eyesight and promote blood circulation.	[270]
			WP	NI	Clear liver, purge fire, resolve knots, dissolve swelling, pacify liver and improve eyesight.	
			S	RW	Purgative, antipyretic, tonic, eyesight weakness asthma, heart diseases, inflammation, diuretics.	[271]
			F	Pas	Migraine, cold and fever, foot fever, stomach gas.	[272]
			AP/FPI	INF	Stomach diseases, eyewash.	[231]
			L	FRE/PE	Wounds healing, teeth cleaning, respiratory problems, joint pains, easing gastric spasms, anti-inflammatory.	[181,273,274]
	*Wild Sage.		AP	DEC/INF	Antidiabetic potential.	[275]

Salvia verbenaca	*Erva-crista.	All the year		INF/DEC	Used for thyroid disorders management.	[276]
				NI	Digestive problems.	[277]
				DEC/INF	Cancer management (Breast, blood, colorectal, liver, lung, intestine).	[278]
			L/S	DEC/INF/MOL/ FPG/BEV/COM/ PLAS	Herpes, eye infection, stomachache, wounds.	[279]
			NU	FRE	Extracting a foreign body from the eye.	[280]
			NI	NI	Used for influenza.	[281]
			L	CRW	Toothache; deodorising the oral cavity.	[282]
				DEC	Stomach ulcers; hypotensive; facilitating menstruation.	
				INF	Digestive.	
				NI	Cough, cold, bronchitis.	
			NI	NI	Abscesses, blisters, cysts, inadequate heart function, insect bites, intestinal worms, kidney stones, liver diseases, lumps, microbial infections, pimples, stomach diseases, and wounds.	
			L	DEC/Pow	Abdominal colics, cold, fever, healing.	[283]
				Pow	Wound treatment.	[284]
				DEC/INF	Genitourinary, skin, digestive, and respiratory problems.	[285]
			L/WP	Pow	Healing of burns, wounds, and abscesses.	[284]
Thymus mastichina	*Spanish Marjoram.	Mar - Aug	-	-	ANI.	-
	*Bela-luz.		NI	INF	Digestif, anticatarrhal agent, analgesic.	[255]

				NI	Used for treating respiratory, digestive, and rheumatic disorders. Used as a condiment/spice flavouring, in seasoning traditional dishes and salads, to preserve olives, to aromatise olive oil, and as a substitute for salt.	[286,287]
			WP	INF	Colds, cough, throat irritations, and abdominal pain. For indigestion and stomach pain.	
			AP	INF	Pain, rheumatism, common cold, cough, diseases of the digestive system, gastric ulcer, functional dyspepsia, abdominal and pelvic pain, nausea, vomiting, malaise, fatigue and localised oedema.	[135]
				FRO	Burns and corrosions	
				NI	Cold; digestive disorder; gastralgia; Bronchitis; urinary infection.	[288]
				DEC	Injuries.	[289]
Plantaginaceae						
No plant.	-	-	-	-	-	-
Rosaceae						
Agrimonia eupatoria	*Common agrimony. *Agrimónia.	May - Jul	NI	NI	Antidiabetic potential.	[290,291]
			AP	INF/DEC	Treatment of tract diseases, chronic wounds, lung inflammation, diarrhoea, liver diseases, cholecystitis, cholestasis, intestinal or bladder atony, pyelonephritis, bleeding disorders, skin defects, and inflammatory conditions of the oral mucosa.	[292]
			L/F/S	PIG	Increase the storage stability of beer.	
			L	INF	Antidiarrheal effects (astringent action), as a gargle in oral cavity inflammation and pharyngitis (sore throat).	

			NI	NI	Antimicrobial actions and improve digestion. Treat pyelonephritis, cholecystitis and cholestasis.	
			GU	INF	Eczema, purulent wound, psoriasis and haemostatic effects.	
Crataegus monogyna	*Hawthorn. *Abronceiro.	Apr-May	Fr	DEC	Antidiabetic potential.	[293]
			F/L	NI	Cardiac diseases, hypertension, hyperlipidemia. anti-atherosclerotic agent, anti-spasmodic, cardiotonic, astringent, and diuretic agent. Improuve blood circulation system, blood stasis elimination, treatment of gastrointestinal diseases, stimulation of digestion, promotion of stomach functions, indigestion, epigastric distension, abdominal pain, diarrhea.	[294]
Geum urbanum	*Wood avens. *Cariofilada.	May - Jul	AP	DEC	Antidiabetic potential.	[295,296]
			F	INF	Sedative, hemostatic, astringent, anti-inflammatory, loss of appetite and diarrhea,	[297]
			R	WB, CATA	Frost bite, hemorrhoids, inflammations of the bladder and urinary tract and skin diseases.	
			R	CATA	Gum and mucous membrane inflammations.	
Potentilla erecta	*Tormentil. *Consolda-vermelha.	May-Aug	R	DEC	Antidiabetic potential.	[295,296]
				INF	Anti-inflammatory in gastric and digestive diseases, antidiarrhea, and antiseptic for the mouth and throat.	[298,299]
			Rhi	DEC	Used to bathe purulent facial eczema and to rinse oral cavity ulceration.	
Prunus avium	*Cherry, wild.		Fr	NI	Antidiabetic potential.	[300]

	*Cerdeira.	Mar - May	L/Fr/Pt	RW/DEC	Diuretic, nephralgia, kidney stones, cardiotonic, vasodilator, diet, debilitating.	[301]
			Fr/Pt	INF	Urinary—bladder, diuretic, kidneys, urinary infection, urinary tract.	[302]
			Pt	DEC	Expectorant, laxative	[303]
			Pt	INF	Intestinal anti-inflammatory, antidiarrhoeic, diuretic, for bladder ailments, analgesic.	[304]
			L/Fr/Pt	INF/DEC/LWP/MS	Atherosclerosis, blood purifier, cardiotonic and vasodilator, tachycardia, cold and flu, bronchitis, carminative, costiveness and intestinal spasm, dyspepsia, diet and debilitating, edema, diuretic, kidney gravels.	[305]
			L	INF	Cough.	[306]
			S	DEC	To pass kidney stones.	[307]
			Fr	NI	Acute nasopharyngitis (common cold), acute pharyngitis, dyspepsia, allergic urticaria, disorder of prostate.	[308]
			Fr	ML	Dysmenorrhoea.	[309]
			LMW	CATA	Wounds/burns.	[310]
			F/Fr/S	DEC	Diuretic, influenza.	[311]
Prunus lusitanica subsp. *lusitanica*	*Portugal laurel. *Azereiro.	May - Jul	NI	NI	ANI.	-
			BAT	NI	Antimalarial agent and to combat snake bites in cattle.	[312]
Rosa canina	*Dog rose. *Rosa-brava.	Apr - Jul	PFr	NI	Antidiabetic potential.	[313]
			PET	INF	Nephritis.	[314]
			Fr	DEC	Cold, flu, cough, bronchitis, immunotonic, asthma.	
			L	DEC	Cold, flu, cough, itching, eczema.	

	L/Fr	DEC	Gallbladder diseases, nephritis.	
	RPFr	NI	Prevention and treatment of common colds; influenza-like infections; infectious diseases; for vitamin C deficiencies; fever; general exhaustion; gastric spasms; prevention of gastritis and gastric ulcers; diarrhea; as a laxative; for gallstones and gallbladder discomforts; urinary tract diseases and discomforts; as a diuretic; for gout, arthritis, sciatica, inadequate peripheral circulation, as an astringent, for lung ailments, as an eye rinse.	[313]
	RFr	NI	For diseases and ailments of the kidneys and lower urinary tract; as a diuretic; for arthritis, rheumatism, gout, sciatica; for Colds; as a laxative; for fever; for vitamin C deficiency and for 'blood purification.	
	Fr	SYR	For preventing scurvy.	[315]
	Fr	TE	To strengthening the body's immune system against infections, common colds, influenza, scurvy, diarrhea, gastritis, cough, vaginitis and urinary tract infections	
	NI	NI	Treating headaches, neural, gastrointestinal diseases, a tonic for heart and brain, and for treating some hepatic disorders.	
	RH	Pow	A remedy to treat rheumatoid arthritis, wounds, and inflammations.	[316]
	Fr	NI	Diuretic, laxative, effective for digestive problems.	
	F/L	DEC	Applied in eye infections as an eye wash.	[317]

			L	NI	the treatment of carbuncles, furuncles and burns.	
				MAC	Diuretic, ingredients in common cold remedies.	
Sorbus aucuparia	*European mountain ash. *Tramazeira.	Jun - Jul	Fr	Jui/INF/DEC	Antidiabetic potential.	[318]
				INF	Headache.	[319]
				DEC	Antirheumatic, astringent, diuretic.	[214,318]
				DEC/Jui	Dietetic, soothing for throat and skin.	[320]
				FRE	Stomachache, warts, headache, Hemorrhoids.	[319,321]
				TINC	It is applied to relieve heart problems, headaches, and hypertension and to disinfect wounds.	[318,319]
				BWS	Blood pressure.	[319]
			BA	TE	Anticancer.	[322]
			L	DEC	Prostatitis, anticancer, for diarrhea, relieve cramps.	[318,321]
			Fr	Jui	Strengthening the walls of blood vessels, but significantly increases blood clotting.	[318]
			NI	NI	Thyrotoxicosis, hypertension, atherosclerosis, decreased immunity, gastritis with low acidity, hypertension and anemia. Antitumor, antisclerotic, anti-inflammatory, hypotensive, pressure regulating properties, regulates digestion, promotes the removal of heavy metals and radioactive materials from the body, and affects the body's hematopoietic activity.	
			L	BIT	Contribute to formation and removal of bile.	
			Fr	INF	sudorific and antipyretic.	

			Fr/L	Jui/INF	Applied in gastro enteric diseases, especially in dysentery and as laxative.	
			Fr	Jui/INF/DEC	Diuretic, against haemorrhoids, anastaltic properties.	
			Fr/F/L	NI	Antidiarrheal, anti-inflammatory, a remedy for abdominal distention.	
			Fr	NI	Can help atherosclerosis.	
			F	NI	Are used to heal urolithiasis.	
			Polygalaceae			
Polygonum aviculare	*Common knotgrass. *Centonódia.	Jul - Oct	AP	NI	Antidiabetic potential.	[323,324]
					Treatment of Colic, Antidiarrhea.	
			WP	TE	Treatment of gastrointestinal diseases, kidney colic as a painkiller, and an anti-inflammatory agent. It's used to treat dislocations, fractures, and furuncles, burns, bronchitis, cough, fever, tumors, and tuberculosis.	[325,326]
Polygonum hydropiper	*Marshpepper knotweed. *Persea.	Jul - Oct	-	-	ANI.	-
			WP	NI	Gastrointestinal disorders and helminthiasis,	[327–329]
				CRU	To treat insomnia, to arrest haemorrhage.	
				NI	To treat inflammatory dyspepsia, diarrhoea, menorrhagia, haemorrhoids, helminthiasis, for convulsion, insomnia and anxiety. Used to treat digestive problems of livestock, as insect repellent for protecting cloths and crops, and as piscicidal medicine.	
			L	NI	Tranquilisers and sedation.	
				INF	It's used to relieve colic pain.	

				Jui	Used to treat anxiety, insomnia, and stomach pain, used as a remedy for headache, pain, toothache, gastric ulcer, dysentery, anorexia, and dysmenorrhoea.	
			R	Pow	For prevention of pregnancy. Continuous use leads to permanent sterility.	
			S	NI	Used to treat snake- bite.	
Rumex acetosa	*Garden sorrel. *Azeda.	May - Sep	L/F/R	DEC	Antidiabetic potential.	[330]
			WP	NI	Gastrointestinal disorders, dermatological infections.	[331–333]
			L	NI	the management of bruises, jaundice, sore throat, scurvy, wounds and warts in Britain and Ireland.	
			F/L	NI	Used to manage lumps and tumors.	
			L/S	NI	Treatment for constipation, cramping, and diarrhea.	
			Fr/L/R	DEC	Treatment for skin disorders, sore throats, and warts.	
			R/S	Pow/Fre	Tenesmus, dysentery, gonorrhoea, fever, ulcer, scabies, skin itch kidney diseases.	
			L	Jui	Fever, diarrhea, lack of appetite, worm, "blood cleanser.	
			R	NI	Tenesmus, dysentery, gonorrhoea, fever, ulcer, scabies, skin itch, kidney diseases.	
			WP	DEC	Digestive problems.	[334]
Rumex crispus	*Curly dock. *Cata-cuz.	Jun - Aug	L/R	DEC/COO	Antidiabetic potential.	[335,336]
			L	BOI	Emollient, laxative.	[337]
			R	NI	Laxative, "blood cleanser", skin diseases, icterus, gastrointestinal tract ailments, bruises, burns,	[331,338]

					swellings, venereal diseases, sores, rashes, gonorrhoea, swellings.	
				DEC	Laxative.	
				NI	Antipyretic, laxative, diarrhoea treatment.	
			NI	NI	Anthrax, purgative, astringent, dysentery, skin diseases, rheumatism, cough, constipation, tonic.	
			Fr	NI	Dysentery, urinary system.	
				BOI	Reduction in blood fat.	
				DEC	Diuretic.	
			L	CRU	Rheumatism, Headache.	
				DEC	Cough, Asthma, Haemorrhoid, Gynecologically Diseases, inflamed wounds, rheumatism.	
				COO	Anticancer.	
				NI	for scabies, blood pressure lowering, antipyretic, for worm, for the biliary system, diuretic, heart diseases, antiphlogistic, infections.	
				NI	Eye infections, antipyretic, expectorant, anti-tussive, vermicide, constipation, dizziness.	
			Rhi	NI	Antipyretic, laxative, Diarrhea treatment,	
Rumex obtusifolius	*Bitter dock.<br>*Azeda-de-folha-larga.	Jun – Jul	-	-	ANI	[331]
			R/SE/AP	NI	Skin diseases, constipation, cough.	
			NI	NI	Astringent, laxative, tonic, antidote to nettle, sores, blisters, burns, cancer, tumour.	
			R	INF	Skin eruption, blood purifier, jaundice, contraceptive.	
			SE	NI	Cough, colds, bronchitis	
			L/R	NI	Burns and boils, blisters, nettle stings/ wrapping up butter, sores, tumours, hepatic, eye,	

					dermatitis, furuncles, bruises, jaundice and fever.	
			Ranunculaceae			
No plant.	-	-	-	-	-	-
			Cyperaceae			
No plant.	-	-	-	-	-	-
			Juncaceae			
Juncus acutus	*Spiny rush. *Unco-agudo.	Mar - Jul	-	-	ANI.	-
			Fr	DEC	Diuretic, Anti-inflammatory, colic, genito-urinary disease, sedative.	[339]
			IF	POP	Stomachic.	[340]
			LAX	CATA	Skin burns.	[341]
			S	RW	Rheumatism.	[342]
			L	INF	Kidney stones.	
			AP/Fr	DEC/Jui	Antihemorragic, nasal bleeding.	[343]
			Fr	DEC	Kidney stones.	[165]
			WP	INF	Diuretic	[344]
			Rubiaceae			
Galium aparine	*Cleavers, bedstraw. *Amor-de-hortelão.	Mar - Jun	F/L	INF	Antidiabetic potential.	[345]
			SE/STK	JIW	Can be applied after bites from spiders and snakes.	[346,347]
			NI	NI	Treatment of lymph swellings, wounds, fever, hypertension, epilepsy disease and cancer.	
			AP	NI	Treatment of lymph swellings, tonsillitis, jaundice, wounds, cancer, fever, scurvy, hypertension and leukemia.	[348,349]

			NI	NI	Can clear away damp-heat, eliminate stasis, dissipate detumescence and detoxify, hematuria, traumatic injury, acute appendicitis, furuncle, otitis media.	[350]
			WP	NI	Used as cooling diuretic in fevers and for urinary tract infections, in skin diseases such as eczema or psoriasis, ulcers, chronic sores, as a blood purifier i.e., to increase lymphatic flow, to reduce swellings, infection and inflammation, or to stop bleeding from wounds.	[351]
			Cistaceae			
Cistus ladanifer	*Gum rockrose.<br>*Esteva.	May - Jun	AP	DEC	Antidiabetic potential.	[352,353]
					Used as an antidiarrhoeal, antiacid, antispasmodic.	
			L	Pow	Gastric acidity.	[354]
Cistus salviifolius	*Sage-leaved rockrose, salvia cistus or Gallipoli rose.<br>*Estevinha.	May - Jun	L/R	DEC/Pow	Antidiabetic potential.	[40,118,355]
			AP	DEC	Skin inflammations, antifungal.	[356,357]
				DEC/WAC	Kidney problems, internal ulcers, skin infections.	[358]
			L/SE/S	DEC/INF/Pow	Respiratory, digestive problems.	[359]
			L/F	INF	Gynaecological disorders.	[360]
			Amaryllidaceae			
Allium victorialis	*Victory onion.<br>*Alho-oblongo-de-raízes-enredadas.	May - Jul	-	-	ANI.	-
			WP	KS	Cough.	[361]
			BU/L/WP	NI	Antitumor, anticytotoxic.	[362,363]
			WP	NI	Abdominal problems, swellings, asthma, respiratory problems and dysentery.	[364]

				EXT	Asthma, respiratory problems.	[365]
Narcissus pseudonarcissus	*Wild daffodil or Lent lily. *Narciso.	Mar - Jun	-	-	ANI	-
			L/BU/F	TE	Anti-spasmodic, tranquiliser, treatment of haemorrhoids, skin diseases.	[366]
			LMH	CATA	Mastitis.	[367]
			R	CATA	Burns, rheumatism.	
				Asparagaceae		
Muscari comosum (*Leopoldia comosa* (L.) Parl. Basion. *Hyacinthus comosus* L)	*Tassel Hyacinth, Tassel grape hyacinth. *Cebolinha-de-flor-azul.	Mar - Jun	-	-	ANI.	-
			BU	INF	Emetic, diuretic.	[133]
				MF	Anti-fever.	[368,369]
				BOI	Hypertension.	[370]
				INF/CATA	Toothache, bad breath, aphrodisiac.	[371]
			NI	NI	Aphrodisiac	[372]
Polygonatum odoratum	*Fragrant Solomon's Sea. *Selo-de-Salomão.	Mar - Jul	Rhi	NI	Antidiabetic potential.	[373]
				NI	Lung diseases, cough, diabetes, indigestion, cardiac disease.	[374,375]
				TE	Will strengthen the body and is especially good for recovering from weakness.	[376]
				SOK	To eliminate fatigue, moisten skin, and make one beautiful.	
Ruscus aculeatus	*Butcher's broom, knee holly or piaranthus. *Erva-dos-vasculhos.	Mar - Jul	-	-	ANI.	-
			Rhi	MAC/Pow	Depurative, aphrodisiac, intestinal pains, hypertension, eczema, otitis, rheumatism.	[371]
			Sho	COO	Liver depurative.	[377]
Urginea maritima	*Squill. *Cebola-albarrã.	Aug - Oct	L	DEC	Antidiabetic potential.	[378]
			NI	NI	Used as an antibacterial, anthelmintic, abortifacient, and diuretic. Also used as an	[379]

(syn. *Drimia marítima*)					expectorant in bronchitis, chronic catarrh and pneumonia. Fresh bulbs are applied to wounds to promote healing. Useful for rheumatism, edema, gout, slowing down the pulse, and in the treatment of cancer.	
			BU	VIN	Used in treating combat hydrops, one of the striking symptoms of cardiac failure.	[380]
				PAD	Aphrodisiac.	[381]
			Boraginaceae			
Anchusa undulata	*Common bugloss or alkanet. *Buglossa-ondeada.	Feb - Aug	L/S	NI	Antidiabetic potential.	[382]
			L/S	NI	Stomach aches.	
			R	DEC/Pow	Anaemia, liver diseases, body pain.	[383]
			S/L	Raw	Kidney stones.	[384]
			L	DEC	Diarrhoea.	[385]
				Raw	Haemorrhages.	[385]
Echium plantagineum	*Paterson's curse. *Chupa-mel.	Mar - Jul	-	-	ANI.	-
			AP	DEC	Diaphoretic and diuretic.	[386]
				INF	Expectorant.	[387]
			NI	NI	Muscular-Skeletal and respiratory problems.	[388]
					Bronchitis, lung cancer, cosmetic applications and skin care.	[389,390]
			F	DEC	Stomach-aches.	[391]
Lithodora prostrata (syn. *Glandora prostrata)*	*Shrubby gromwell, creeping gromwell or purple gromwell.	Jan - Oct	-	-	ANI.	-
			AP	DEC	Blood troubles (reported as "blood purifier or to strengthen blood") abortive and hypercholesterolemia.	[136]

	*Erva-das-sete-sangrias.			INF	For bladder ailments, blood circulation, for chilblain, for colds, anti-tussive, for seasickness, gastric analgesic, antipyretic, hepatic protector, digestive, for influenza, antipneumonic, dismenorrheic, Intestinal anti-inflammatory, blood depurative, for intoxication, antidermatosic, sedative, antirheumatic, renal anti-spasmodic.	[304]
			NI	NI	Anti-hypertensive and used for cataracts.	[392]
					Ericaceae	
Arbutus unedo	*Strawberry tree. *Ervedeiro.	Oct - Feb	L/R	DEC	Antidiabetic potential.	[40,159,393–396]
			L/R/COR /Fr	DEC/INF/RW	Hypertension, respiratory problems, digestive problems, rheumatism, kidney diseases.	[371]
			NI	NI	Anticholesterolemic, anti-hypertensive, antihypotensive, cardiotonic, diuretic, for iron-deficiency, hematocathartic, vasotonic.	[392]
			R	DEC	Cardiotonic, for abdominal pain, renal anti-spasmodic, bladder ailments.	[304]
					Abortive, antihypercholesterolaemic.	
				SYR	Blood depurative.	
Erica scoparia	*Besom Heath. *Moita-alvarinha.	Dec - Aug	-	-	ANI.	-
			L	INF	Antiinflammatory.	[397]
			F	INF	Urinary antiseptic, antiemetic, antispasmodic.	[398,399]
		Apr - Jul	Fr/L	FRE/LIQ/TE	Antidiabetic potential.	[400,401]

Vaccinium myrtillus	*Bilberry, blaeberry, wimberry, and whortleberry. *Arando.		L/F	NI	Acute diarrhoea, blood purifier, inflammation of the mouth and throat.	[400,402]
			Rr	TE	Asthma.	
			NI	NI	Endocrine and gastrointestinal system disorder. Antihaemorrhoidal, anti-hypertensive., diuretic.	[403]
			L	NI	An astringent, diuretic, vasoprotective, for anaemia, varicose and thread veins, poor circulation improving blood supply to veins and capillaries.	[402]
			Fr	DRE/INF	Laxative.	
				FRE/LIQ/TE	Gastrointestinal tract.	[404]
				FRE	Astringent and remedy for circulation and sight disorders.	[405]
Vaccinium uliginosum	*Bog bilberry, bog blueberry, northern bilberry or western blueberry. *NI	Jul - Aug	-	-	ANI.	-
			L	SYR	Used for inducing menstruation, treatment of bladders stones, liver disorders, coughs and lung ailments, intestinal conditions, typhoid fever, infections of the mouth, skin, and urinary tract infections, rheumatism.	[406]
			Fr	TE	Used as an astringent for diarrhea and dysentery, as a diuretic, cooling nutritive tonic, to prevent scurvy (vitamin C deficiency), and to stop bleeding. It is also used as an astringent and disinfectant mouthwash for mouth inflammations.	
				FRE	Snack.	[407]
			Onagraceae			

No plant.	-	-	-	-	-	-
			Orchidaceae			
No plant.	-	-	-	-	-	-
			Campanulaceae			
Jasione montana var. *gracilis* (syn. *Jasione montana* subsp. *Montana*)	*Sheep's-bit. *Baton-azul.	Apr - Sep	-	-	ANI.	-
			NI	NI	Sleep disorders in children.	[408]
			Scrophulariaceae			
Verbascum sinuatum	*Scallop-leaved mullein. *Cachapeiro.	Jun - Sep	-	-	ANI	-
			L/F	NI	Ichotoxic (poison) plant to harvest fish in rivers by the ancient Mediterranean or as an insecticidal agent to kill pests in food products.	[409]
			NI	TOP/BOI	Anti-hemorrhoidal, leg treatment, anti-hypertensive, arthrosis, rheumatism, eczema, exanthema.	[410]
				BOI	Teeth pain, gumboil, chilblain, helminthiasis.	
				TOP	Liver inflammation, contusion, broken bones, cysts and zits.	
				INF	Gallstone, anti-diarrhoea, cold.	
				INF/TOP/BOI	Ucer/inflammation.	
				INF/BOI/MAC	Cough, asthma, bronchitis, hemoptysis.	
				INF/TOP	Hoarse, tonsillitis.	
				MAC/TOP	Wounds, ulcers, burns.	
				MAC	Conjunctivitis, otitis.	

Verbascum thapsus	*Common mullein. *Barbasco.	Jun - Aug	R	NI	Antidiabetic potential.	[411–413]
			L	DEC	Wound healing.	[414]
			L/F/R	NI	Used to reduce mucous formation and stimulate the coughing up of phlegm.	[415]
				TOP	It is used as good emollient, wound healer, astringent.	
			AP	INF	Pulmonary affections (bronchitis)	[416]
			L/R	CATA	Asthma, treatment for respiratory disorders.	[417]
				INF/MAC	Arthritis and gout.	
			R	SYR	For coughs.	
			L	CATA/INF	Analgesic, anti-inflammatory, a blood purification, treatment for headaches.	
				DEO	Stomachache.	[418]
				WA	To relieve pain and also used for softening boils.	[419]
			L/F	MIL	Asthma.	
				Geraniaceae		
Geranium purpureum	*Little robin. *Bico-de-grou.	Mar - Jul	-	-	ANI.	-
			AP	INF	Antiulcerose, vulnerary, anti-cancerous, intestinal anti-spasmodic, digestive, gastric analgesic, hepatic protector, for seasickness, for gallbladder ailments, for gastritis, for influenza, intestinal anti-inflammatory, renal anti-spasmodic.	[304]
Geranium pyrenaicum subsp. *lusitanicum*	*Hedgerow cranesbill. *NI	May - Aug	-	-	ANI.	-
			-	-	-	-

Geranium robertianum	*Herb-Robert. *Erva-de-São-Roberto.	Mar - Jul	AP/L	DEC/INF	Antidiabetic potential.	[420]
			F	INF	Gripes, headaches, stomach problems, liver problems.	
				MAC	Tonsillitis.	
			F/L	INF	Diarrhea.	
				DEC	Rheumatic.	
			L	INF/DEC	Prevention of cancer, astringent.	
				MG	Oropharyngeal inflammation.	
				INF/DEC	Poorly healing wounds, mosquito bites.	
			AP	INF/DEC	Diarrhea, gastritis, gout, gallbladder inflammation, kidney inflammation, liver problems, bladder inflammation, calculosis, sinuses diseases, nose bleeding, sciatica, tonic, hypertension, diuretic, cancer, anti-spasmodic.	
			Orobanchaceae			
No plant.	-	-	-	-	-	-
			Papaveraceae			
Chelidonium majus	*Greater celandine. *Aruda.	Mar - Sep	S/ROD	DEC/POW	Antidiabetic potential.	[421]
			NI	HJH	Used to treat visual impairment and eye disease.	[422]
				SWA	Useful in treating jaundice and dermatologic disorders such as herpes.	
			L	INF	Cholagogue and to regulate action of the digestive trac	
			L/F/S/AP	LAX/Jui/CATA	Used to remove warts, to treat wound.	
			R/L	FRE	Treat corns.	
			AP/SE	DEC/INF/BTH	To expel roundworms.	

			NI	NI	Diuretic, for gout, used as tonic and stimulant of cardiac functions, also increasing blood pressure. Antidote for snake venom, depurative.	
			AP	TE	Employed in the treatment of cancer.	
				SLA	Remedy for pustules.	
				INF	Digestion, hemorrhoids, jaundice, liver, eye disease, skin diseases.	[423]
				RAW	Carminative, costiveness, warts, corns.	
			L	DEC	Rheumatism, sciatica.	
			SA	DAP	Vulnerary, for warts.	[304]
Papaver dubium	*Long-headed poppy. *Papoila.	Jan - Jul	-	-	ANI.	-
			F	NI	Used for diseases and discomforts of the respiratory tract, treat jaundice, for disturbed sleep and as a sedative, and for the relief of pain.	[424]
			PET	PCW	Immunotonic, galactagogue, and for epistaxis.	
			NI	NI	Anti-tussive and expectorant action, especially for the elderly and children. Pain reliever and sedative. Used to treat nervousness, insomnia, digestive and respiratory disorders, baldness, eye infection, as well as for measles treatment.	
			PET	SYR	Bronchial calmative, for a cough, and a sore throat.	
			AP	INF	To treat rheumatism.	
			L	FRE	Tonic.	
					Hypericaceae	
Hypericum androsaemum	*Tutsan or sweet-amber. *Erva-da-pedra.	Jun - Sep	-	-	ANI.	-
			L	INF	Used for liver, kidney and bladder ailments.	[425]
				PLO	Dressing cuts and wounds.	

			NI	NI	Cholagogue, hepatoprotector, nephroprotective and diuretic	[426]
			AP	INF	Sedative, hepatic protector, antihypertensive, for good disposition.	[304]
Hypericum humifusum	*Trailing St John's Wort *Erva-das-mil-folhinhas.	Mar - Sep	-	-	ANI.	-
			AP	INF	Diuretic, gastric analgesic, digestive, hepatic protector, for gallbladder and bladder ailments, urinary system.	[304]
			WP	NI	Hematemesis, hematochezia, irregular menstruation.	[427]
			NI	NI	Skin and digestive diseases.	[428]
Hypericum perforatum	*St John's-wort. *Erva-de-São-João.	May - Oct	NI	NI	Antidiabetic potential.	[429]
					Reproductive, muscular-skeletical, skin and digestive diseases.	[428]
					The relief of temporary mental exhaustion, for the symptomatic treatment of minor inflammations of the skin (such as sunburns), and as an aid in the healing of minor wounds, as well as for the symptomatic relief of mild gastrointestinal discomfort.	[430]
			AP	INF	Used for migraine, renal anti-spasmodic, hepatic protector, bladder and gallbladder ailments, seasickness, Intestinal anti-inflammatory, and its sedative.	[304]
			WP	CATA/DEC	Hemoptysis, hematemesis, metrorrhagia, traumatic hemorrhage, irregular menstruation, acute mastitis, jaundice, sore throat, swelling and pain of eye, urinary tract infection, sore furuncle, burn, wounds and bruises, rheumatic arthritis.	[427]

			Liliaceae			
No plant.	-	-	-	-	-	-
			Fagaceae			
Castanea sativa	*Spanish chestnut or sweet chestnut or European chestnut. *Castanheiro.	May - Jun	-	-	ANI.	-
			NI	NI	Blood cleaning, anthelmintic, against rachitis, In rheumatism and arthritis, as astringent and insecticide.	[431]
					Treat cough in children, hypertension, infertility and diarrhea.	[432]
			Fr/STB	INF	Antipyretic, diarrhea, hemorrhoids.	[184]
			L	INF/CATA	Bechic, against sores.	[433]
			L/F/BA	MAC	Anti-dysenteric, anti-spasmodic, infertility treatment, diarrhoea., cough (children), mucolitic.	[137]
			Fr	MAC	Odontalgy, haemorrhoids, erysipelas.	[68]
				FRE	Nervousness.	
Quercus pyrenaica	*Pyrenean oak. *Carvalho-da-Beira.	Apr - May	-	-	ANI.	-
			-	-	-	-
Quercus suber	*Cork oak. *Chaparreiro.	Apr - Jul	-	-	ANI.	-
			BA	MAC	Odontalgy, muscle pain.	[434]
				PSP	Hemostatic and healing in wound care.	[284]
			L	CRU	Hair strengthening	[435]
			Dryopteridaceae			
Dryopteris dilatata	*Broad wood fern or broad buckler fern. *NI	May - Nov	-	-	ANI.	-
			-	-	-	-
			Salicaceae			
No plant.	-	-	-	-	-	-

			Aspleniaceae			
No plant.	-	-	-	-	-	-
			Pteridaceae			
Adiantum capillus-veneris L.	*Maidenhair fern. *Aivenca.	All the year.	NI	NI	Antidiabetic potential.	[436]
			FROD	DEC	Used for female pains. It is used as a sedative in cases of dysmenorrhea, toothache, rheumatism and intestinal spasms in the diarrheic colitis. Is used in case of haemorrhaging in childbirth, pain in childbirth. For respiratory ailments, cough, asthma, depurative after childbirth, abortive, to increase milk production. It's diuretic and used against inflammation of the genitourinary tract. Used as a male-aphrodisiac.	[437]
				POUB	Are employed in cases of eye inflammation.	
				POUR	Poultices of roasted fronds induce cicatrisation.	
				CATA	Eye inflammation.	
				INF	Is used as an emenagogue and to treat bronchitis and coughs. As expectorant; aperitive, gastritis, carminative to treat liver disorders.	
				Pas	Used for scorpion and snake bite.	
				NI	Are used as febrifuge.	
				IDM	Is used as an anti-tussive.	
				DPR	Used as regulator of menses, anti-cough, abortive.	
			WP	NI	Is used as an aromatic and an emollient in cough. Used for mouth blisters, eye diseases, fever, bronchitis, menstrual complaint. Used for the	

					treatment of cough, asthma, fever, jaundice, cold, measles and chest pain.	
			Rhi	CATA	Used as a cicatrizant.	
			AP	DEC	Is used as an external application for hair loss, renal disorders. It is used as a bath in cases of dermatitis, cystitis, as astringent, diuretic, tonic, antibiotic, emetic, expectorant, febrifuge, jaundice and hepatitis.	
				Caprifoliaceae		
Lonicera periclymenum	*European honeysuckle. *Madressilva.	May - Jul	-	-	ANI.	-
			L/F/BA	DEC	Anti-asthmatic, antibiotic, antipyretic, childbirth induction, diaphoretic, diuretic, hepatic protector, oral antiseptic, urinary tract diseases.	[137]
			FPA	INF	Abdominal and pelvic pain, cramps general.	[135]
			L		Cardiotonic.	[399]
Sambucus nigra	*Black elder. *Sabugueiro.	Apr - Aug	-	-	ANI.	-
			IF	FUM	For mastitis	[438]
			F	IAP/ESS	Antidiarrhoeal.	
				INF/ESS	Intestinal antiseptic.	
			Fr	SYR		
			BA	DEC	Gout.	[68]
			F	INF/MAC	Cold, asthma.	
			L	CATA/INF	Insect bites, erysipelas.	
			Br	NI	Haemorrhoids.	
			F/L/BA	TE	Anti-allergic, anti-catarrhal; anti-inflammatory; anti-influenza; diaphoretic; diuretic.	[137]
			L	NI	Local abscesses, fever, headache, and urinary tract infections.	[439]

			F	DEC	Colds, pains, and skin diseases.	
			Fr	NI	Fever, influenza, nasal congestion, and coughs.	
					Gentianaceae	
Centaurium erythraea	*Common centaury and European centaury. *Fel-da-terra.	Apr - Sep	AP/FAAP	INF/DEC	Antidiabetic potential.	[40,159,304,395,396]
			AP	INF	For bladder ailments, kidney stone, antipyretic, anti-infectious, Intestinal anti-inflammatory, for migraine, sedative.	[304]
			AP	MAW	Appetising.	
			FTOP	DEC	Tonic, appetiser, febrifuge, increase bile production, anthelmintics, digestive, sedative.	[133]
Gentiana lutea subsp. *lutea*	*The great yellow gentian. *Genciana-amarela.	Jun - Aug	NI	EXT	Antidiabetic potential.	[440]
			SE/F/L	INF	Digestive disorders, sexual tonic, appetite, rheumatism, dermatological problems.	[68]
			NI	EXT/WMAC	Improving the immune system, for stomach, aphrodisiac, gastric and duodenal ulcer, blood purification, for appetite, cancer diseases.	[440]
			R	LIN	Abdominal pains, digestive, strengthening the immune system.	[213]
					Euphorbiaceae	
No plant.	-	-	-	-	-	-
					Plumbaginaceae	
No plant.	-	-	-	-	-	-
					Resedaceae	
No plant.	-	-	-	-	-	-
					Saxifragaceae	
No plant.	-	-	-	-	-	-

			Violaceae			
No plant.	-	-	-	-	-	-
			Betulaceae			
Corylus avellana	*European filbert, European hazelnut or cobnut. *Avelaneira.	Dec - Feb	-		ANI.	-
			Fr	JAM/Raw/COS	Antialopecic.	[441]
			L	TE	For diarrhoea and heavy menstruation.	[402]
			Fr/BA	INF	Cardiac diseases, prostatic hypertrophy.	[214]
			Fr/BA/L	DEC	Antiseptic, intestinal astringent, for haemorrhoids (piles), antiseptic for minor wounds and sores.	
			Raw	SEC	Cough, high cholesterol, anemia.	[401]
			Iridaceae			
No plant.	-	-	-	-	-	-
			Primulaceae			
Anagallis arvensis	*Scarlet pimpernel. *Erva-do-garrotilho.	Feb - Oct	WP	TE	Antidiabetic potential.	[442]
			WP	CATA	Expectorant, diuretic, hepatitis, itching, insect bites, chronic infectious wounds.	[133]
			AP	DEC	Diuretic, anti-tussive, inflamed wound.	[320]
			FTOP	INF/WB	Diuretic, astringent, anti-tussive, to stimulate flow of bile, expectorant, stimulates glandular secretion, soothes swollen legs and feet.	
			L/F	SANJ	Skin inflammations and infections, burns, corns, wounds.	[443]
			Iridaceae			
No plant.	-	-	-	-	-	-
			Woodsiaceae			
No plant.	-	-	-	-	-	-
			Urticaceae			

Urtica dioica	*Common stinging nettle. *Ortigão.	May - Jun	L/S	DEC/INF	Antidiabetic potential.	[40,133,355,395]
			L/R	DEC/INF	Anemia, antiinflammatory, digestive.	[133]
			L	TOP	Infected wounds, skin inflammations, corns, hard skin, warts.	[443]
			L/S	TLS/BOI/SOU/Raw/FRIC	Circulatory conditions, thin the blood, rheumatism and arthritis pain.	
				TLS/BOI/SOU	Gall stones, stimulate immune system.	
Urtica membranacea	*Large-leaved Nettle, Membranous Nettle. *Ortiga-alta.	All the year.	-	-	ANI.	-
			AP	DEC	Tussis, Tonsillitis, gum inflammations.	[444]
			WP/R	NI	Gastrointestinal purifier and anti-diarrhoeic.	[445]
			WP	INW	Hair loss.	[282]
			L	TOP	Sciatica	
			L	DEC	Diuretic; depurative; abdominal colics.	
				Portulacaceae		
Portulaca oleracea	*Purslane or moss rose. *Baldroega.	May - Sep	AP	DEC	Antidiabetic potential.	[163,167]
			L/SE	FOO	Stomach problems, Kidney stone, Stop bleeding, Breast inflammations in breastfeeding mothers, Thirst.	[133]
			L	FRE/COO	Stomach and intestinal bleeding, diuretic, anemia, food.	[446]
			AP	EPP	Aphrodisiac.	[438]

			AP/SE	NI	Hemoptysis, gastritis, liver inflammation, intestinal ulcers, kidneys and bladder ulcers, hemorrhagic vomiting, fevers, insomnia, cough, tonsillitis, asphyxia, nocturnal emissions. As an anti-thirst, food digestive, anti-parasite, depurative, anti-hemorrhoids and diuretic medicine.	[447]
				EPP	Severe inflammations, erysipelas, pulsatile headaches caused by hot temperament, eye pain, teeth sensitivity, blepharitis, mouth ulcers, testicular swelling.	
				Malvaceae		
Malva neglecta	*Buttonweed, Common Mallow. *Malva-das-folhas-redondas.	May - Sep	AP	INF/COO	Antidiabetic potential.	[448]
			L	INF	Antiseptic, ocular antiseptic, antihaemorrhoidal, genital antiseptic.	[304]
			Fr/F/L	FOO	Colds, kidney and bladder infections, anti-tussive, antipyretic.	[133]
			L/F	INF/DEC	Infectious wound, sinusitis, teeth pain, acne removing, abdominal pains, foot infection, uterus infection, laxative, cough.	[449]
			AP	DEC	Diuretic, for urinary inflammations, abscesses, tranquilliser.	[21]
Malva sylvestris	*Common mallow. *Malva-comum.	Apr - Sep	-	-	ANI.	-
			L/Sho	INF	For obstipation, anti-hypertensive.	[304]
			L	VAP	Antihaemorrhoidal.	
				INF	Antiseptic, intestinal anti-inflammatory, anti-inflammatory, anti-infectious, vaginal antiseptic, antidermatosic, for urinary system.	
			AP	GAR	Buccopharyngic, anti-inflammatory.	

				MOU	Antiodontalgic.	
			L/F	FOO	Laxative, diuretic, cough, mouth and throats inflammations, anti-inflammatory.	[133]
					Oleaceae	
Olea europaea var. europaea	*European olive. *Oliveira-brava.	May - Jul	L/F/Fr	INF/DEC/MAC/ Pow	Antidiabetic potential.	[167,243,394–396,450]
			L/S	INF	Anti-hypertensive, hepatic protector, antihypercholesterolaemic, sedative, antiulcerose, anti-anaemic, cardiotonic.	[304]
			Fr	OLJ	To treat gallstones.	[451]
			SE	OI	Laxative.	
			L/Fr	DEC	Diarrhea, respiratory, and urinary tract infection.	
			Fr	EPP	To prevent hair loss.	
			L	BOI	To treat asthma.	
				INF	Antipyretic, anti-inflammatory, tonic, eye infections treatment.	
					Santalaceae	
No plant.	-	-	-	-	-	-
					Solanaceae	
Solanum nigrum	*European black nightshade. *Erva-moira.	Apr - Nov	WP	Pow	Antidiabetic potential	[100]
			NI	Pow	Malignant sores, bad breath.	[452]
				DEC	Invigorate qi and nourish blood, remove blood stasis and detoxify, cancer, phlegm scrofula, breast mass, wheezing, vomiting.	
			Fr	FOO	Diuretic diaphoretic, antispasmodic, emollient and emetic. For treatment of diarrhea, fever and eye problems.	[453]

			L	INF	Has sedative, cholagogue and narcotic properties.	
				Amaranthaceae		
Chenopodium ambrosioides (Syn. *Dysphania ambrosioides*)	*Mexican tea *Ambrósia-do-México.	Aug - Nov	AP/L/BA	DEC/INF/MAC	Antidiabetic potential.	[454–456]
			R	SYR	Is used for influenza treatment.	[457]
			L	OI	Treat parasitic diseases.	
				MIM	Used for the treatment of flu or intestinal worms.	
				TE	Employed to treat digestive disorders.	
				NI	Fertility control.	
				INF	The leaf infusion is orally taken to alleviate stomach pains.	
			AP	DEC	Used to relieve muscle pain and treat bone injuries.	
			L/R	Pas	Applied to the area affected by inflammation for treatment.	
				Apocynaceae		
No plant.	-	-	-	-	-	-
				Araliaceae		
No plant.	-	-	-	-	-	-
				Cupressaceae		
Juniperus communis	*Common juniper. *Zimbro-anão.	Jun - Aug	BA	NI	Antidiabetic potential.	[458]
			Fr	NI	Inflammatory diseases of the lower urinary tract, dyspeptic complaints.	[400]
				Jui/INF	Diuretic, common cold, cough, asthma, gonorrhoea.	[459]
			L	INF	It is given in leucorrhoea and dysmenorrhoea.	[460]
				Pas	Applied on skin ailments.	

				Lycopodiaceae		
Lycopodium clavatum	*Common club moss. *Licopódio-da-estrela.	Jul - Sep	NI	NI	Antidiabetic potential.	[461]
			WP/AP/ L/S/SPO	NI	Used for the treatment of rheumatic disease, pulmonary disease, dermatological diseases including rashes, rush in babies and protection of tender skin, muscle pain and fatigue, stomach pain, headache, Burns, wounds, and inducing pregnancy, Alzheimer's disease, central nervous system conditions, as nerve tonics and for motor disorders, and used as expectorants, laxatives and diuretics.	[462]
			Sho	Pow	Relieve from muscle pain and fatigue.	[463]
			SPO	OIS	Against dermatitis.	[464]
			L/SPO	POIL	Cancers, prostate enlargement.	[465]
				Lythraceae		
Lythrum salicaria	*Purple loosestrife. * Erva-carapau.		-	-	ANI.	-
			F	DEC	Astringent, antihemorrhagic, tonic, cleansing. Dysentery, diarrhoea, intestinal inflammation, hematuria, leucorrhea, epistaxis, dysmenorrhea, lupus, eczema, impetigo, female urogenital inflammation.	[466]
			WP	INF	Lowering blood pressure.	
			R	NI	Diarrhea, gastrointestinal tract ailments, blood circulation disorders, skin ailments	
			AP	DEC	Digestive, carminative. Indisposition of intestines, colic, diarrhoea.	
				CATA	Menorrhagia, wound healing, ulcers and eczema.	[467]
				Paeoniaceae		

No plant.	-	-	-	-	-	-
				Cytinaceae		
Cytinus hypocistis	*Rockrose parasite. * Pútegas-de-escamas-largas.	Mar - Jul	-	-	ANI.	-
			F	NI	Heart problems.	[468]
			NI	DEC	Used for dysentery and its astringent qualities; other reported uses include the treatment of tumours of the throat and eye inflammations and as an emmenagogue.	[469]
			WP	COM	Astringent, tonic, and haemostatic substance.	[470]
Cytinus hypocistis subsp. *hypocistis*	*Yellow Cytinus. *Coalhadas.	Mar - Jul	-	-	-	-
				Pinaceae		
Pinus pinaster	*Maritime pine or cluster pine. *Pinheiro-bravo.	Mar - Mar	Sho	INF	Antidiabetic potential.	[304]
					For colds	
				MOU	Buccal antiseptic.	
			RES	DAP	For wounds on the hands and feet, for burns.	
				MAL	Muscular analgesic.	
			CO	INH	Antiasmatic.	
			Sho, CO	SYR	Antitussive.	
Pinus sylvestris	*Scots pine. *Pinheiro-da-Flandres.	Mar	-	-	ANI.	-
			CO/Sho/ RES	SYR/DEC	Cough.	[471]
			NED	TE	Anticancer.	[472]
			Sho	BTH	Rheumatic pain.	[473]
				Thymelaeaceae		
		Jul - Oct	L/BA	INF	Antidiabetic potential.	[243,352]

Daphne gnidium	*The flax-leaved daphne.<br>*Erva-de-João-Pires.		L	NI	Haircare, hair dyeing, abortive, dermatological problems.	[474]
				DEC/L	Remove toxins.	
			L/BA	INF	Cure toxicity, abortive, haircare, purgative, and against skin diseases.	
			AP	DEC	Odontalgy.	
			R/BR	NI	Insecticides.	
Typhaceae						
No plant.	-	-	-	-	-	-
Ulmaceae						
Ulmus glabra	*Scotch elm.<br>*NI	Feb - Apr	-	-	ANI.	-
			BA	POMIL	To soften blood vessels in painful vascular obstruction in extremities.	[7]
			BA/TWI	CATA	Diuretic, diarrhea, rheumatism, warts, fractures.	[133]
			BA	OIT/DEC	Applied to wounds as an haemostatic. Applied to haematoma and haemorrhoids.	[303]
			L/BA	DID	Antidiarrheal.	[475]
			BA	LOT	Antiinflammatory.	
			BR	DID	Weakening, galactacrasic.	
			L	LOT/DID	Antiecchymotic, laxative.	
Valerianaceae						
No plant.	-	-	-	-	-	-
Xanthorrhoeaceae						
No plant.	-	-	-	-	-	-
Lauraceae						
Laurus nobilis	*Bay laurel.	Feb - Apr	L	INF/DEC	Antidiabetic potential.	[391,476,477]

	*Loireiro-vulgar.		NI	NI	For indigestion, skin swellings, hysteria, analgesic, sedative, anti-spasmodic, tonic, stimulant, astringent.	[478]
			L	INF	Colopathy, stress, urinary, cutaneous infections, constipation, obesity, bronchitis, rhinitis, muscular, inflammation, gynecological problems, sleep disturbances, allergies.	[479]
				OI	For acne, hair loss, pain.	[391]
				DEC	Stomachache, cold, chokes. Scorpion bite, bee sting, snake bit, hemorrhoid, rheumatism, pain, digestive, antipyretic, antiseptic, stomach disorders, prostatitis, wound, cold, kidney diseases, appetising, diaphoretic, stomachache, diuretic, weakness, sedative, headache, cardiovascular disease, ulcer, tonic, antihelminthic, antistress, respiratory disease, flatulence, intestinal diseases, diarrhea, skin diseases.	
				Cannabaceae		
Humulus lupulus	*Common hop. *Engatadeira.	Jun - Aug	L	NI	Antidiabetic potential.	[480]
			NI	NI	Useful to treat sleeplessness and nervousness, treat excitability and restlessness associated to tension headache; to improve appetite, digestion; to relieve toothache, earache and neuralgia. Exert diuretic, antispasmodic and anaphrodisiac effects. Sedative, antirheumatic, analgesic and as a urinary aid for "gravel" and inflammation, to treat restlessness associated with nervous tension, headache and indigestion.	[481]

			HP	POU	Treatment of pneumonia.	
			NI	DEC	Intestinal pain and fevers.	
			NI	NI	Toothache, earache, neuralgia, leprosy, tuberculosis, asbestosis, silicosis, anthelmintic, antiparasitic, cough, spasms, fever, anxiety, clearing blood, flatulence, Delirium tremens, irritable bladder, aches, diuresis, liver disorders (porphyria), gastric problems, indigestion, appetite, bleary eyes, tenderness of limbs, sedative, sleep disturbances, antibiotic, anti inflammatory.	[482]
					Colchicaceae	
No plant.	-	-	-	-	-	-
					Commelinaceae	
No plant.	-	-	-	-	-	-
					Convolvulaceae	
Convolvulus arvensis	*Field bindweed. *Corriola.	Apr - Sep	L/WP	DEC	Antidiabetic potential.	[483]
			WP	TOP	It used as vegetable relieving constipation and as purgative, and for skin problems.	[484]
			WP	NI	It is used in excessive menstrual bleeding and leucorrhea.	[485]
				DEC/TOP	Abdominais pains, diarrhea, jaundice, gynecological problem, abdominal pains, diarrhea, jaundice, gynecological problema.	[486]
					Cucurbitaceae	
Bryonia dioica (Syn. *Bryonia cretica* subsp. *Dioica*)	*Red bryony. *Briónia.	Apr - Sep	R	NI	Antidiabetic potential.	[487]
			NI	NI	Breast cancer, hypercholesterolemia, fertility disorder, rheumatism.	
			R	MHO	Used for the Treatment of Cancer.	[488]

			L/Fr	INF	Anti-hypertensive.	[381]
			R	MCO/PRW	Diuretic, to reinforce bones, anti-sciatica.	[433]
			R/L	DEC/POU	Purgative, revulsive.	[489]
			R	DECO	Antirheumatic and antineuralgic.	[490]
			Fr	MAL	Injuries.	[280]
			NI	NI	Is used as a potent cathartic, cytotoxic, diaphoretic, expectorant, hydrogogue, irritant, pectoral, purgative and also as a vermifuge. Various inflammatory conditions, bronchial complaints, asthma, intestinal ulcers, hypertension and arthritis.	[491]
			WP	FOO/CATA	Laxative, diuretic, diaphoretic, rheumatism, bruises, mucosa inflammation, warts, bronchitis, asthma.	[133]
					Dennstaedtiaceae	
No plant.	-	-	-	-	-	-
					Dioscoreaceae	
Tamus communis (Syn. *Dioscorea communis*)	*black bryony. *Arrebenta-boi.	Mar - Jun	-	-	ANI.	-
			Rhi	TE/RHP	For rheumatism, sciatica.	[402]
			Fr/R	GRA/FRE	Used against furunculosis, rheumatic pains and against muscle pains.	[492]
			Fr	RUB	Aching bones.	[493]
					Droseraceae	
No plant.	-	-	-	-	-	-
					Elatinaceae	
No plant.	-	-	-	-	-	-
					Haloragaceae	
No plant.	-	-	-	-	-	-

			Melanthiaceae			
No plant.	-	-	-	-	-	-
			Menyanthaceae			
No plant.	-	-	-	-	-	-
			Moraceae			
Ficus carica	*Common fig. *Bebereira.	Aug - Sep	L/Fr	DEC	Antidiabetic potential.	[159,163,355,476]
			Fr	SYR	Is used as a remedy for mild constipation.	[494,495]
				JH	It can be used for haemorrhage.	[495]
				NI	Antidiarrheal, metabolic, cardiovascular, respiratory, anti-spasmodic, anti-inflammatory, antiplatelet, inflammatory, and gut motility, antioxidant, laxative, anticancer, expectorant, and diuretic.	[495,496]
			L	NI	Cough., prevention of nutritional anaemia, anthelmintic, irritant potential, tuberculosis.	
			Fr/L/R	NI	Indigestion, colic treatment, loss of appetite.	
			Myrtaceae			
Eucalyptus globulus	*Tasmanian bluegum. *Eucalipto-comum.	Nov - Mar	L/Fr	DEC	Antidiabetic potential.	[159,167,395]
			L/S	INF	Analgesic, antiseptic, antirheumatic.	[304]
			L/S	INF	Intestinal anti-inflammatory, renal anti-spasmodic, for bladder ailments, anti-hypertensive.	
			L/S	VAP/INH	Nasal descongestive, antiasmatic, bronchodilator.	
			L	INF	Cough, grippe.	[113]
				INF/VAP	Anti-catarrhal. expectorant. for respiratory disorders.	[497]
			Oxalidaceae			

Oxalis pes-caprae	*Bermuda buttercup. * Amendoim-bravo.	Jan - Apr	-	-	ANI.	-
			BU/R	DEC	Digestive and respiratory problems.	[174,203]
			NI	NI	Vermifuge.	[498]
			AP	INF/	Mouth problems (aphthous), appetiser, stomachache.	[499]
			S	FRE	Digestive.	[302]
					Phytolaccaceae	
Phytolacca americana	*Pokeweed. *Baga-moira.	May - Aug	-	-	ANI.	-
			L/STK	EA	Clean blood.	[500]
			L	INS	Insecticide.	[501]
			AP	NI	Digestive, treatment of obesity.	[502]
			L	BOI	Management of skin disorders.	[503]
				INF	Depurative.	[504]
			R	CC	Anaemia.	[505]
			L/Fr/R	NI	Diarrhoea, rheumatism.	[506]
					Potamogetonaceae	
No plant.	-	-	-	-	-	-
					Proteaceae	
No plant.	-	-	-	-	-	-
					Rhamnaceae	
No plant.	-	-	-	-	-	-
					Sapindaceae	
No plant.	-	-	-	-	-	-
					Simaroubaceae	
Ailanthus altissima	*Tree of heaven. *Ailanto.	Apr - Jun	BA	NI	Antidiabetic potential.	[507]
			SE	OI	To treat urticaria.	[508]
			BR	INF	Diarrhoea.	[509]

			L/BA	DEC	Anthelmintic, gastroenteritis.	[385]
			BA		Treatment of uterine bleeding, postpartum hemorrhage, acute and chronic dysentery, metritis, and enteritis.	[510]
				DEC1	Treatment of large intestine damp-heat ulcerative colitis.	
				DEC2	Treatment of ulcerative colitis.	
				DEC3	Treatment of late threatening abortion.	
				DEC4	Treatment of dysfunctional uterine bleeding induced by hepatic heat and spleen asthenia.	
				NI	Used for asthma, epilepsy, spermatorrhea, bleeding, and ophthalmic diseases. It's an intestinal astringent, tourniquet, antidiarrheal, insecticide, maintain hemostasis and used for reducing stool output. Used to remove phlegm from the lungs and stomach and relieve spleen dampness.	
				PIL1	Treatment of irregular menstruation, morbid vaginal discharge, edema, and chronic inflammation.	
				PIL2	Treatment of epilepsy, asthma, yellowish leukorrhea, trichomonas vaginitis, amoeba vaginitis, senile vaginitis, and cervix inflammation.	
				PIL3	Treatment of chronic cervicitis, endometritis, and vaginal mucositis.	
					Taxaceae	
Taxus baccata	*English yew.	Mar - Apr	R	DEC	Antidiabetic potential.	[163]

	*Teixo.		BA/L	DEC/TE	Headache, bone fracture, cancer, asthma, bronchitis, epilepsy, arthritis, scorpion stings.	[511]
			L	MAL	Antirheumatic and antineuralgic.	[490]
				INF	Epilepsy, headache.	[512]
			BA	NI	Used to cure bone fracture.	[513]
			BR	DEC	Emmenagogue, abortifacient, cardiotonic.	[492]
			COP	OIT/BTH	Antipyrotic, external antiseptic, cosmetic (hair), resolvent.	[31]
					Verbenaceae	
Verbena officinalis	*Common vervain. *Algebrado.	Jun - Dec	L	DEC	Antidiabetic potential.	[393]
			AP	DEC/CATA	Depression, mild tonic, sedative, nervous system tonic.	[133]
			L/R	Pow/Pas	Burns, wounds, nuts, abscesses, pimples, healing wounds.	[284]
			AP	PLS	For haemostatic and cicatrising purposes.	[493]
				PLSP	Rheumatic pains in knees and elbows.	
				CHOP	For thyroid problems.	
				OIT/POUL	Antiecchymotic, antineoplastic, resolvent.	[31]
				ROI	Bruises, muscular pains, rheuma.	[514]
				TE	A diuretic, calms the nerves, increases perspiration for insomnia, depression, migraines and nervousness, for fever, lowers high temperatures, reduces inflammation and relieves pain.	[402]
					Vitaceae	
Vitis vinifera	*European wine grape.	May - Jun	L	DEC	Antidiabetic potential.	[163,515,516]

	*Parreira.		L/Fr/BR/ SE/LAX	DRO/Raw/FCW/ DFC/Jui/CIN/FR ANK/DRE/MK Ms/CATA	Watering of eyes, lung cancer, burn, boil and wound care, anemia, bronchitis, cold and flu, carminative, costiveness and intestinal spasm, dyspepsia, allergy.	[305]
			UNF/RIF	FOO/REW/VIN	Anti-diarrhoea, galactagogue, against sore throat, laxative, anti-dandruff.	[517]
			F/L/Fr	Jui/RIF	Is used as laxative, purgative, diuretic, aphrodisiac, in fever, asthma, jaundice, vomiting, stomach problems, laves used in piles, joint aches, flower is expectorant, liver tonic, bronchitis, constipation.	[518]
			Fr	Mas/SYR	For strengthening of the organism and strengthening of the thyroid gland, and for improving of blood picture and strengthening of the organism.	[519]
					Buxaceae	
Buxus sempervirens	*Common Box. *Buxo-arbóreo.	Jan - Fev	L	DEC	Antidiabetic potential.	[450]
				OIT/BAL/TINC	For purulent ulcers and wounds, tincture against rheumatism.	[519]
				BTH/LOT	Antiverrucose.	[31]
				INF	Purgative.	[520]
				DEC	Febrifuge	[521]
			AP	TE/Raw	Anticholagogue, antihelminthic, antinauseous, bechic, carminative, curative, reconstituent.	[475]

ANI: Antidiabetic potential not identified, AP: Arial parts, BA: Bark, BAL: Balm, BAT: The bark of the trunk, BEV: Beverage, BD; Bud, BIT: Bitter, BOI: Boiled, BR: Branches, BRE: Fresh roots are pulverised and mixed with flour to make bread, BTH: Bath, BU: Bulb, BWS: Boiled with sugar, CATA: Cataplasm, CHE: Chewing, CHOP: Chopped together with *Thymus longicaulis subsp. longicaulis* and *Parietaria diffusa*. Then add bean flour and beaten egg white and apply with a cloth to the neck for 4–5 h or, preferably, overnight. Use for 3 days, suspend for three days, repeat cycle three times, CIN: Cinder, CL: Cladodes, CO: Cone, COM: Compresses, COP: Cortical parenchyma, COO: Cooked, COR: Cortex, COS: Cooked (boiled with salty water and then toasted), CRE: Creams, CRU: Crushed plant, CRW: By chewing and rubbing, DAP: Direct application, DEC: Decoction, DEC1: The plant was mixed with *Paeoniae radix* Alba, *Coptidis Rhizoma*, *Astragali Radix*, *Codonopsis Radix*, Poria, *Angelicae sinensis* Radix, *Arecae Semen*, Aucklandiae Radix, Cinnamomi Cortex, *Glycyrrhizae radix* and Rhizoma, DEC2: The plant was mixed with *Gentianae macrophyllae* Radix, Atractylodis Rhizoma, *Phellodendri chinensis* Cortex, DEC3: The plant was mixed with *Pulsatillae Radix*, *Lonicerae japonicae* Caulis, *Taxilli Herba*, *Asini corii* Colla, Pseudostellariae Radix, Astragali Radix, Scutellariae Radix, Mori Folium, DEC4: The plant was mixed with *Atractylodis macrocephalae* Rhizoma, Platycladi Cacumen, Trachycarpi Petiolus, DECO: Decoction mixed with olive oil, DEO: Leaves are mixed with bulbs of onion (*Allium cepa* L.) and decocted. Preparation is given orally, DFC: Dried fruit eaten by chewing; DFE: Dried flower extracts, DPR: Decoction of about a quarter of a litre of water with some dried figs, an apple, some *Prunus cerasus* fruits, some pieces of *Prunus dulcis epicarpus* and *A. capillus-veneris* fronds. The decoction is filtered and honey or sugar is added, DRE: Dried, DID: Direct digestion, DRI: Drink, EA: Usually eaten with other wild greens; fried or "killed" by pouring hot grease on top of leaves, DRO: Drops, EAP: Extract- ash of pea, EMU: Emulsion, EPP: External application, ESS: Essence, EXT: Extract, EX EYD: Eye drops, F: Flowers, FAAP: Flowering and arial parts, FAP: Flowered aerial parts, FB: Flowering branches, FCF: Fruit cluster flower, FCW: Fruits are eaten by chewing, FH: Flower heads, FOF: Flowering offshoot, FOO: Food, FPG: Fresh plant ground, FPI: Flowering apical parts, Fr: Fruits, FRANK: Frankincense; FRE: Fresh, FRO: Fry in oil, FROD: Fronds, FTOP: Flowering tops, FUM: Fumigation, GAR: Gargle, GCB: Gargles, compresses and baths, GE: Gel, GTO: Green tops, GU: Gum, HJH: Use herb juice, boiled with honey in a brass vessel, HP: Heated hops, IAP: Tisane (with *Achillea ptarmica*), IDM: Infusion or decoction in milk, INF: Infusion, IF: Inflorescences, INH: Inhalation, INS: The plant, dried in the sun and ground to a powder, was placed in stalls as an insecticide, INW: By washing the head with infusion, JAM: As a thickening agent for jams, JH: The juice of fig fruit mixed with honey, JIW: Juice mixed in wine, Jui: Juice, KS: Korean salad, seasoned cooked vegetables, mixed vegetables with spices, L: Leaves, LAX: Latex, LC: Crushed leaves scrubbed on the hands, LIN: Lincura-'gentian brandy': about 10 g root, dried and diced is put into a dark glass bottle, covered with brandy and left to sit for 3 weeks: slighter people drink 1 small glass, while larger people can have 2 small glasses a day, before meals, LIW: Leaves immersed in wine, mixed with tomato pulp, LIQ: Liquor, LMH: Fresh leaves macerated with honey, LOT: Lotion, Lpw: Leaf powder; MAC: Aqueous maceration, MAL: Alcohol maceration, MAW: Maceration in wine, MF: Cut, macerated in water, then fried, MG: Mouthwashes/gargles (infusion/decoction), MHO: Mixed with honey, MIL: About 40 g of dried leaves and flowers are boiled in 1000 g of Cow's milk for 10 min, strained and the filtered milk is given to the patient thrice daily in cases of asthma, MIM: Mixed with milk, MK: Mask, MOA: Macerated in alcohol or in oil, MOL: Macerated in olive oil, MOU: To wash one's mouth, Ms: Mash, NED: Needles, NU: Nuts, OB: Oral bath, OI: Oil, OIS: Ointment made by mixing and heating olive oil with the spores, OIT: Ointment, OLJ: Olive oil + lemon juice, PAD: Pads form, Pas: Paste, PD: Pods, PE: Plant extract, PET: Petals, PCW: Petals (excludiIg the black parts) waiting in sun with lemon and water, PIG: Pigment, PIL: Pills, PIL1: Pills contained also Codonopsis Radix, *Angelicae sinensis* Radix, Aucklandiae Radix, Foeniculi Fructus, Eucommiae Cortex, Psoraleae Fructus, Indigo

Naturalis, Ostreae Concha, *Atractylodis macrocephalae* Rhizoma, Paeoniae radix Alba, Cyperi Rhizoma, Amomi Fructus, Corydalis Rhizoma, Dipsaci Radix, *Celosiae cristatae* Flos, PIL2: Pills contained also *Phellodendri chinensis* Cortex, *Paeoniae* radix Alba, Angelicae sinensis Radix, Cyperi Rhizoma, PIL3: Pills contained also *Phellodendri chinensis* Cortex, Poria, Dioscoreae Rhizoma, *Schisandrae chinensis* Fructus, *Testudinis carapax* et Plastrum, Asini corii Colla, PLAS: Plasters and poultices, PLO: The pounded leaves were mixed with lard to produce an ointment, PLS: Plaster, PLSP: Plasters, sometimes with petrol, PO: Pollen, POIL: Powder or oil are mixed with Beeswax and honey, and taken daily in the form of capsule during one month, POMIL: Peeled barks are cut into small pieces and boiled in milk, then the poultice is applied to affected extremities, POUB: Poultices of boiled fronds, POUL: Poultice, POUR: Poultices of roasted fronds, POP: Powder consumed with honey, Pow: Powder, PRW: Poultice prepared from prolonged boiling of cubes of the root in a small amount of water, PSP: The internal bark powder is sprinkled directly on burns, Pt: Pedicel, R: Roots, RES: Resin, REW: Red wine, RFr: *Rosae fructus*, Rhi: Rhizome, RPFr: *Rosae pseudofructus*, RH: Rose hips, RHP: Rub sore areas with root; afterwards sore areas become red and warm; apply oil or milk afterwards to prevent inflammation; or, apply a compress prepared from planed rhizome with or without plum brandy, RIF: Ripe fruit, ROD: Rods, ROI: Roasted with oil and egg whites in poultice and placed between cloths, RUB: For pains in the joints, rubbed onto the skin, RW: Raw, S: Stems, SA: SAP, SANJ: Pomada sanjuanera: Ointment prepared frying together many plants such as: *Achillea millefolium*, *Allium ampeloprasum*, *Anagallis arvensis*, *Calendula officinalis*, *Chamaemelum nobile*, *Cruciata glabra*, *Eleocharis quinqueflora*, *Glycyrrhiza glabra*, *Hedera helix*, *Hypericum hirsutum*, *Hypericum perforatum*, *Inula montana*, *Jasonia tuberosa*, *Lavandula latifolia*, *Malva sylvestris*, *Mentha×piperita*, *Pinus sylvestris*, *Plantago lanceolata*, *Plantago major*, *Potentilla erecta*, *Potentilla reptans*, *Primula veris*, *Pulmonaria longifolia*, *Ranunculus ficaria*, *Rumex sp.*, *Sambucus nigra*, *Santolina chamaecyparissus*, *Symphytum tuberosum*, *Thymus vulgaris*, *Tussilago farfara*, *Verbascum thapsus* and *Verbena officinalis*. SE: Seeds, SEC: The seeds are crushed, SFL: Squashed fresh leaves, Sho: Shoots, SLA: A salve from celandine, olive oil, fir resin and beeswax was a remedy for abscesses, SOK: Soaked with liquor and sugar for six months, SOU: Soup, SPO: Spore, STB: Stem bark, STK: Stalks, STR: Straws, SWA: Soaked in wine together with anise fruits, SYR: Syrup, TAG: Tablets and granules, TB: Topic/Boiled; TE: Tea, TINC: Tincture, TIS: Tisane (with *Parietaria officinalis* ssp. *judaica* and *Beta vulgaris* ssp. *vulgaris* var. *vulgaris*), TLS: Tender leaves and stems, TOP: Topic, TWI: Twigs, UNF: Unripe fruits, VAL: Valve, VAP: Vapor, VIN: Vinegar, VIM: The naked body of the patient is covered with fresh leaves which are wetted with vinegar or raw sheep milk and kept warm to induce perspiration, WA: The leaves are warmed, tightly tied on the joints to relieve pain and also used for softening boils, WAS: Washing, WB: In water bath, WMAC: Water maceration, WP: Whole plant, YB: Young buds.

References

1. Lorenzi, H.; Matos, F.J.A. *Plantas Medicinais No Brasil: Nativas e Exoticas*; Instituto Plantarum, Ed.; 2002;
2. Corrêa, A.D.; Batista, R.S.; Quintas, L.E.M. Plantas Medicinais: Do Cultivo à Terapêutica: Contém Formulação e Modo de Preparo de Cosméticos. **2003**, 247–247.
3. De Liz Oliveira Cavalli, V.L.; Sordi, C.; Tonini, K.; Grando, A.; Muneron, T.; Guigi, A.; Júnior, W.A.R. Avaliação in Vivo Do Efeito Hipoglicemiante de Extratos Obtidos Da Raiz e Folha de Bardana Arctium Minus (Hill.) Bernh. *Revista Brasileira de Farmacognosia* **2007**, *17*, 64–70, doi:10.1590/S0102-695X2007000100014.
4. Coelho De Souza, G.; Haas, A.P.S.; Von Poser, G.L.; Schapoval, E.E.S.; Elisabetsky, E. Ethnopharmacological Studies of Antimicrobial Remedies in the South of Brazil. *J Ethnopharmacol* **2004**, *90*, 135–143, doi:10.1016/J.JEP.2003.09.039.
5. Erdemoglu, N.; Turan, N.N.; Akkol, E.K.; Sener, B.; Abacioglu, N. Estimation of Anti-Inflammatory, Antinociceptive and Antioxidant Activities on Arctium Minus (Hill) Bernh. Ssp. Minus. *J Ethnopharmacol* **2009**, *121*, 318–323, doi:10.1016/J.JEP.2008.11.009.
6. Neves, J.M.; Matos, C.; Moutinho, C.; Queiroz, G.; Gomes, L.R. Ethnopharmacological Notes about Ancient Uses of Medicinal Plants in Trás-Os-Montes (Northern of Portugal). *J Ethnopharmacol* **2009**, *124*, 270–283, doi:10.1016/J.JEP.2009.04.041.
7. Sezik, E.; Yeşilada, E.; Honda, G.; Takaishi, Y.; Takeda, Y.; Tanaka, T. Traditional Medicine in Turkey X. Folk Medicine in Central Anatolia. *J Ethnopharmacol* **2001**, *75*, 95–115, doi:10.1016/S0378-8741(00)00399-8.
8. Mosaddegh, M.; Naghibi, F.; Moazzeni, H.; Pirani, A.; Esmaeili, S. Ethnobotanical Survey of Herbal Remedies Traditionally Used in Kohghiluyeh va Boyer Ahmad Province of Iran. *J Ethnopharmacol* **2012**, *141*, 80–95, doi:10.1016/J.JEP.2012.02.004.
9. Ramírez, G.; Zavala, M.; Pérez, J.; Zamilpa, A. In Vitro Screening of Medicinal Plants Used in Mexico as Antidiabetics with Glucosidase and Lipase Inhibitory Activities. *Evidence-based Complementary and Alternative Medicine* **2012**, *2012*, doi:10.1155/2012/701261.
10. Akram, M. Minireview on Achillea Millefolium Linn. *Journal of Membrane Biology* **2013**, *246*, 661–663, doi:10.1007/S00232-013-9588-X.
11. Li, Y.; Zhang, M.-L.; Cong, B.; Wang, S.-M.; Dong, M.; Sauriol, F.; Huo, C.-H.; Shi, Q.-W.; Gu, Y.-C.; Kiyota, H. Achillinin A, a Cytotoxic Guaianolide from the Flower of Yarrow, Achillea Millefolium. *jstage.jst.go.jpY Li, ML Zhang, B Cong, SM Wang, M Dong, F Sauriol, CH Huo, QW Shi, YC Gu, H KiyotaBioscience, biotechnology, and biochemistry, 2011•jstage.jst.go.jp* **2011**, *75*, 1554–1556, doi:10.1271/bbb.110234.

12. Dall'Acqua, S.; Bolego, C.; Cignarella, A.; Gaion, R.M.; Innocenti, G. Vasoprotective Activity of Standardized Achillea Millefolium Extract. *Phytomedicine* **2011**, *18*, 1031–1036, doi:10.1016/J.PHYMED.2011.05.005.
13. medicines, M.B.-T. guide to herbal; 1999, undefined The Complete German Commission E Monographs. *cir.nii.ac.jp*.
14. Abdelhalim, A.; Aburjai, T.; Hanrahan, J.; Abdel-Halim, H. Medicinal Plants Used by Traditional Healers in Jordan, the Tafila Region. *Pharmacogn Mag* **2017**, *13*, S95, doi:10.4103/0973-1296.203975.
15. Paradise, L. *Homeopathic Pharmaceutical Compositions*; 1998;
16. Pieroni, A.; Giusti, M.E. Alpine Ethnobotany in Italy: Traditional Knowledge of Gastronomic and Medicinal Plants among the Occitans of the Upper Varaita Valley, Piedmont. *J Ethnobiol Ethnomed* **2009**, *5*, 1–13, doi:10.1186/1746-4269-5-32/TABLES/3.
17. Vidic, D.; Ćavar Zeljković, S.; Dizdar, M.; Maksimović, M. Essential Oil Composition and Antioxidant Activity of Four Asteraceae Species from Bosnia. *http://dx.doi.org/10.1080/10412905.2016.1150216* **2016**, *28*, 445–457, doi:10.1080/10412905.2016.1150216.
18. Pljevljakušić, D.; Janković, T.; Jelačić, S.; Novaković, M.; Menković, N.; Beatović, D.; Dajić-Stevanović, Z. Morphological and Chemical Characterization of Arnica Montana L. under Different Cultivation Models. *Ind Crops Prod* **2014**, *52*, 233–244, doi:10.1016/J.INDCROP.2013.10.035.
19. Devkota, H.P.; Aftab, T. Medicinal Plants of the Asteraceae Family: Traditional Uses, Phytochemistry and Pharmacological Activities. *Medicinal Plants of the Asteraceae Family: Traditional Uses, Phytochemistry and Pharmacological Activities* **2022**, 1–230, doi:10.1007/978-981-19-6080-2/COVER.
20. Šutovská, M.; Capek, P.; Kočmalová, M.; Pawlaczyk, I.; Zaczyńska, E.; Czarny, A.; Uhliariková, I.; Gancarz, R.; Fraňová, S. Characterization and Pharmacodynamic Properties of Arnica Montana. *Int J Biol Macromol* **2014**, *69*, 214–221, doi:10.1016/J.IJBIOMAC.2014.05.051.
21. Cakilcioglu, U.; Turkoglu, I. An Ethnobotanical Survey of Medicinal Plants in Sivrice (Elazığ-Turkey). *J Ethnopharmacol* **2010**, *132*, 165–175, doi:10.1016/J.JEP.2010.08.017.
22. Karakaş, F.P.; Karakaş, A.; Boran, Ç.; Türker, A.U.; Yalçin, F.N.; Bilensoy, E. The Evaluation of Topical Administration of Bellis Perennis Fraction on Circular Excision Wound Healing in Wistar Albino Rats. *Pharm Biol* **2012**, *50*, 1031–1037, doi:10.3109/13880209.2012.656200.
23. Morikawa, T.; Li, X.; Nishida, E.; Nakamura, S.; Ninomiya, K.; Matsuda, H.; Hamao, M.; Muraoka, O.; Hayakawa, T.; Yoshikawa, M. Medicinal Flowers. XXXII.1) Structures of Oleanane-Type Triterpene Saponins, Perennisosides VIII, IX, X, XI, and XII, from the Flowers of Bellis Perennis. *Chem Pharm Bull (Tokyo)* **2011**, *59*, 889–895, doi:10.1248/CPB.59.889.

24. Cakilcioglu, U.; Turkoglu, I. An Ethnobotanical Survey of Medicinal Plants in Sivrice (Elazi{dotless}ĝ-Turkey). *J Ethnopharmacol* **2010**, *132*, 165–175, doi:10.1016/J.JEP.2010.08.017.
25. Uzun, E.; Sariyar, G.; Adsersen, A.; Karakoc, B.; Ötük, G.; Oktayoglu, E.; Pirildar, S. Traditional Medicine in Sakarya Province (Turkey) and Antimicrobial Activities of Selected Species. *J Ethnopharmacol* **2004**, *95*, 287–296, doi:10.1016/J.JEP.2004.07.013.
26. Laurie Pippen *All Natural Antiviral Agents - Natural Ways to Prevent or Treat*; Eiram Publishing.; 2015;
27. Naceiri Mrabti, H.; Bouyahya, A.; Naceiri Mrabti, N.; Jaradat, N.; Doudach, L.; Faouzi, M.E.A. Ethnobotanical Survey of Medicinal Plants Used by Traditional Healers to Treat Diabetes in the Taza Region of Morocco. *Evidence-based Complementary and Alternative Medicine* **2021**, *2021*, doi:10.1155/2021/5515634.
28. Chaachouay, N.; Benkhnigue, O.; Fadli, M.; Ibaoui, H. El; Zidane, L. Ethnobotanical and Ethnopharmacological Studies of Medicinal and Aromatic Plants Used in the Treatment of Metabolic Diseases in the Moroccan Rif. *cell.comN Chaachouay, O Benkhnigue, M Fadli, H El Ibaoui, L ZidaneHeliyon, 2019•cell.com* **2017**, *5*, e02191, doi:10.1016/j.heliyon.2019.e02191.
29. El Hachlafi, N.; Chebat, A.; Soulaymani Bencheikh, R.; Fikri-Benbrahim Research, K. Ethnopharmacological Study of Medicinal Plants Used for Chronic Diseases Treatment in Rabat-Sale-Kenitra Region (Morocco). *Ethnobotany Research and Applications*, **2020**, *20*, doi:10.32859/era.20.2.1-23.
30. Passalacqua, N.G.; Guarrera, P.M.; De Fine, G. Contribution to the Knowledge of the Folk Plant Medicine in Calabria Region (Southern Italy). *Fitoterapia* **2007**, *78*, 52–68, doi:10.1016/J.FITOTE.2006.07.005.
31. Rigat, M.; Vallès, J.; Dambrosio, U.; Gras, A.; Iglésias, J.; Garnatje, T. Plants with Topical Uses in the Ripollès District (Pyrenees, Catalonia, Iberian Peninsula): Ethnobotanical Survey and Pharmacological Validation in the Literature. *J Ethnopharmacol* **2015**, *164*, 162–179, doi:10.1016/J.JEP.2015.01.055.
32. Wazaify, M.; Afifi, F.; El-Khateeb, M.; Ajlouni, K. Complementary and Alternative Medicine Use among Jordanian Patients with Diabetes. *Complement Ther Clin Pract* **2011**, *17*, 71–75.
33. Ravindran, P.N.; Divakaran, M.; Pillai, G.S. Other Herbs and Spices: Achiote to Szechuan Pepper. *Handbook of Herbs and Spices: Second Edition* **2012**, *2*, 534–556, doi:10.1533/9780857095688.534.
34. Boudjelal, A.; Henchiri, C.; Sari, M.; Sarri, D.; Hendel, N.; Benkhaled, A.; Ruberto, G. Herbalists and Wild Medicinal Plants in M'Sila (North Algeria): An Ethnopharmacology Survey. *J Ethnopharmacol* **2013**, *148*, 395–402, doi:10.1016/j.jep.2013.03.082.
35. Ouelbani, R.; Bensari, S.; Mouas, T.N.; Khelifi, D. Ethnobotanical Investigations on Plants Used in Folk Medicine in the Regions of Constantine and Mila (North-East of Algeria). *J Ethnopharmacol* **2016**, *194*, 196–218, doi:10.1016/j.jep.2016.08.016.

36. Dei Cas, L.; Pugni, F.; Fico, G. Tradition of Use on Medicinal Species in Valfurva (Sondrio, Italy). *J Ethnopharmacol* **2015**, *163*, 113–134, doi:10.1016/J.JEP.2014.12.054.
37. Fleming, T. *PDR for Herbal Medicines (3rd Ed.)*; Medical Economics Company, Ed.; Montvale, 2000;
38. Blumenthal, M. The Complete German Commission E Monographs. *Therapeutic Guide to Herbal Medicines* **1999**.
39. National Institute for the Control of Pharmaceutical and Biological Products *Chinese Traditional Medicine*; Beijing, 1990;
40. Idm'hand, E.; Msanda, F.; Cherifi, K. Ethnopharmacological Review of Medicinal Plants Used to Manage Diabetes in Morocco. *Clinical Phytoscience 2020 6:1* **2020**, *6*, 1–32, doi:10.1186/S40816-020-00166-Z.
41. Saad, B.; Said, O. Greco-Arab and Islamic Herbal Medicine: Traditional System, Ethics, Safety, Efficacy, and Regulatory Issues. **2011**.
42. Hmamouchi, M. *Les Plantes Médicinales et Aromatiques Marocaines. 2éme*; 2001;
43. Tahraoui, A.; El-Hilaly, J.; Israili, Z.H.; Lyoussi, B. Ethnopharmacological Survey of Plants Used in the Traditional Treatment of Hypertension and Diabetes in South-Eastern Morocco (Errachidia Province). *J Ethnopharmacol* **2007**, *110*, 105–117, doi:10.1016/J.JEP.2006.09.011.
44. Afifi-Yazar, F.U.; Kasabri, V.; Abu-Dahab, R. Medicinal Plants from Jordan in the Treatment of Cancer: Traditional Uses vs in Vitro and in Vivo Evaluations Part 1. *Planta Med* **2011**, *77*, 1203–1209, doi:10.1055/S-0030-1270832.
45. Talib, W.H.; Mahasneh, A.M. Antimicrobial, Cytotoxicity and Phytochemical Screening of Jordanian Plants Used in Traditional Medicine. *Molecules* **2010**, *15*, 1811–1824, doi:10.3390/MOLECULES15031811.
46. Hudaib, M.; Mohammad, M.; Bustanji, Y.; Tayyem, R.; Yousef, M.; Abuirjeie, M.; Aburjai, T. Ethnopharmacological Survey of Medicinal Plants in Jordan, Mujib Nature Reserve and Surrounding Area. *J Ethnopharmacol* **2008**, *120*, 63–71, doi:10.1016/J.JEP.2008.07.031.
47. Talib, W.; Molecules, A.M.-; 2010, undefined Antimicrobial, Cytotoxicity and Phytochemical Screening of Jordanian Plants Used in Traditional Medicine. *mdpi.comWH Talib, AM MahasnehMolecules, 2010•mdpi.com* **2010**, *15*, 1811–1824, doi:10.3390/molecules15031811.
48. Kaileh, M.; Berghe, W. Vanden; Boone, E.; Essawi, T.; Haegeman, G. Screening of Indigenous Palestinian Medicinal Plants for Potential Anti-Inflammatory and Cytotoxic Activity. *J Ethnopharmacol* **2007**, *113*, 510–516, doi:10.1016/J.JEP.2007.07.008.
49. Trojan-Rodrigues, M.; Alves, T.L.S.; Soares, G.L.G.; Ritter, M.R. Plants Used as Antidiabetics in Popular Medicine in Rio Grande Do Sul, Southern Brazil. *J Ethnopharmacol* **2012**, *139*, 155–163, doi:10.1016/J.JEP.2011.10.034.
50. Govindarajan, M.; Vaseeharan, B.; Alharbi, N.S.; Kadaikunnan, S.; Khaled, J.M.; Al-anbr, M.N.; Alyahya, S.A.; Maggi, F.; Benelli, G. High Efficacy of (Z)-γ-Bisabolene from the Essential Oil of Galinsoga Parviflora (Asteraceae) as Larvicide and

Oviposition Deterrent against Six Mosquito Vectors. *Environmental Science and Pollution Research* **2018**, *25*, 10555–10566, doi:10.1007/S11356-018-1203-3.

51. Santosa, E.; Zaman, S.; … D.G.-I.J. of; 2020, undefined Agroecology and Uses of Galinsoga Parviflora as Indigenous Vegetable in Highland of Kuningan, Banjarnegara and Wonosobo, Indonesia. *journal.ipb.ac.idE Santosa, S Zaman, D Guntoro, AD SusilaIndonesian Journal of Agronomy, 2020•journal.ipb.ac.id* **2020**, *48*, 339–347, doi:10.24831/jai.v48i3.32800.

52. Zhang, Z.; Chen, Y.; Jiang, X.; Zhu, P.; Zeng, Y.; Tang, T.; Li, L. Characterization of the Complete Chloroplast Genome Sequence of Galinsoga Parviflora and Its Phylogenetic Implications. *Mitochondrial DNA B Resour* **2019**, *4*, 2106–2108, doi:10.1080/23802359.2019.1623106.

53. Ali, S.; Zameer, S.; Research, M.Y.-T.J. of P.; 2017, undefined Ethnobotanical, Phytochemical and Pharmacological Properties of Galinsoga Parviflora (Asteraceae): A Review. *tjpr.orgS Ali, S Zameer, M YaqoobTropical Journal of Pharmaceutical Research, 2017•tjpr.org* **2017**, *16*, 3023–3033, doi:10.4314/tjpr.v16i12.29.

54. Parihaar, R.; Bargali, K.; Agricultural, S.B.-I.J. of; 2014, undefined Diversity and Uses of Ethno-Medicinal Plants Associated with Traditional Agroforestry Systems in Kumaun Himalaya. *academia.eduRS Parihaar, K Bargali, SS BargaliIndian Journal of Agricultural Sciences, 2014•academia.edu*.

55. Katiyar, S.; Saxena, A.; … M.S.-… of P. and; 2020, undefined In-Vitro Anti-Arthritic and Anti-Platelet Activity of Galinsoga Parviflora Linn. *phytojournal.comS Katiyar, A Saxena, M Singh, D DarundeJournal of Pharmacognosy and Phytochemistry, 2020•phytojournal.com* **2020**, *9*.

56. Matu, E.N.; Van Staden, J. Antibacterial and Anti-Inflammatory Activities of Some Plants Used for Medicinal Purposes in Kenya. *J Ethnopharmacol* **2003**, *87*, 35–41, doi:10.1016/S0378-8741(03)00107-7.

57. North, M.A.E.-G.-A. and B.J. of; 2016, undefined Traditional Medicinal Plants of Nigeria: An Overview. *scholar.cu.edu.egMM Abd El-GhaniAgriculture and Biology Journal of North America, 2016•scholar.cu.edu.eg* **2016**, doi:10.5251/abjna.2016.7.5.220.247.

58. Sen, S.; Chakraborty, R.; De, B.; Research, N.D.-J. of F.; 2011, undefined An Ethnobotanical Survey of Medicinal Plants Used by Ethnic People in West and South District of Tripura, India. *SpringerS Sen, R Chakraborty, B De, N DevannaJournal of Forestry Research, 2011•Springer* **2011**, *22*, 417–426, doi:10.1007/s11676-011-0184-6.

59. Matu, E.N.; Van Staden, J. Antibacterial and Anti-Inflammatory Activities of Some Plants Used for Medicinal Purposes in Kenya. *J Ethnopharmacol* **2003**, *87*, 35–41, doi:10.1016/S0378-8741(03)00107-7.

60. Katiyar, S.; Saxena, A.; … M.S.-… of P. and; 2020, undefined In-Vitro Anti-Arthritic and Anti-Platelet Activity of Galinsoga Parviflora Linn. *phytojournal.comS Katiyar, A Saxena, M Singh, D DarundeJournal of Pharmacognosy and Phytochemistry, 2020•phytojournal.com* **2020**, *9*.
61. North, M.A.E.-G.-A. and B.J. of; 2016, undefined Traditional Medicinal Plants of Nigeria: An Overview. *scholar.cu.edu.egMM Abd El-GhaniAgriculture and Biology Journal of North America, 2016•scholar.cu.edu.eg* **2016**, doi:10.5251/abjna.2016.7.5.220.247.
62. Sen, S.; Chakraborty, R.; De, B.; Devanna, N. An Ethnobotanical Survey of Medicinal Plants Used by Ethnic People in West and South District of Tripura, India. *J For Res (Harbin)* **2011**, *22*, 417–426, doi:10.1007/S11676-011-0184-6/METRICS.
63. STP, C. Fungi-Toxic Properties of Leaf Extracts of Some Herbaceous Wild Plants. *Int. J. Sci. Res* **2014**.
64. Ali, S.; Zameer, S.; Yaqoob, M. Ethnobotanical, Phytochemical and Pharmacological Properties of Galinsoga Parviflora (Asteraceae): A Review. *Tropical Journal of Pharmaceutical Research* **2017**, *16*, 3023–3033, doi:10.4314/tjpr.v16i12.29.
65. Onaran, M.; Orhan, N.; Farahvash, A.; Ekin, H.N.I.; Kocabiyik, M.; Gönül, I.I.; Şen, I.; Aslan, M. Successful Treatment of Sodium Oxalate Induced Urolithiasis with Helichrysum Flowers. *J Ethnopharmacol* **2016**, *186*, 322–328, doi:10.1016/J.JEP.2016.04.003.
66. Orhan, N.; Onaran, M.; Şen, I.; Işik Gönül, I.; Aslan, M. Preventive Treatment of Calcium Oxalate Crystal Deposition with Immortal Flowers. *J Ethnopharmacol* **2015**, *163*, 60–67, doi:10.1016/J.JEP.2015.01.009.
67. Carmona, M.D.; Llorach, R.; Obon, C.; Rivera, D. "Zahraa", a Unani Multicomponent Herbal Tea Widely Consumed in Syria: Components of Drug Mixtures and Alleged Medicinal Properties. *J Ethnopharmacol* **2005**, *102*, 344–350, doi:10.1016/J.JEP.2005.06.030.
68. Benítez, G.; González-Tejero, M.R.; Molero-Mesa, J. Pharmaceutical Ethnobotany in the Western Part of Granada Province (Southern Spain): Ethnopharmacological Synthesis. *J Ethnopharmacol* **2010**, *129*, 87–105, doi:10.1016/J.JEP.2010.02.016.
69. Pullaiah, T. *Encyclopedia of World Medicinal Plants*; Regency Publication.; 2006;
70. Rajan, S.; Sethuraman, M. *Folk Medicine of the Nilgiri Hills in Southern India, Survey of Medicinal Plants and Collection Unit,* ; New Delhi, India, 2008;
71. Abraham, Z. *Ethnobotany of the Todas, the Kotas and the Irulas of the Nilgiris.* ; S. K. Jain, Ed.; Oxford and IBH Publications: New Delhi, India, 1981;
72. Technol, T.A.-J.-Int.J.D.Del.; 2020, undefined Lactuca Serriola: Short Review of Its Phytochemical and Pharmacological Profiles. *researchgate.netTZ Abdul-JalilInt. J. Drug Del. Technol, 2020•researchgate.net* **2020**, *10*, 505–508, doi:10.25258/ijddt.10.3.34.

73. Janbaz, K.H.; Latif, M.F.; Saqib, F.; Imran, I.; Zia-Ul-Haq, M.; De Feo, V. Pharmacological Effects of Lactuca Serriola L. in Experimental Model of Gastrointestinal, Respiratory, and Vascular Ailments. *Evidence-based Complementary and Alternative Medicine* **2013**, *2013*, doi:10.1155/2013/304394.
74. Yadava, R.N.; Jharbade, J. New Antibacterial Triterpenoid Saponin from Lactuca Scariola. *Fitoterapia* **2008**, *79*, 245–249, doi:10.1016/J.FITOTE.2007.11.028.
75. Khan, D.; Hina Iqbal, P.; Sher, Z.; Khan, Z.U. Medicinal Plants from Salt Range Pind Dadan Khan, District Jhelum, Punjab, Pakistan. *academicjournals.orgH Iqbal, Z SherJournal of Medicinal Plants Research, 2011•academicjournals.org* **2011**, *5*, 2157–2168.
76. Aziz, M.A.; Adnan, M.; Khan, A.H.; Rehman, A.U.; Jan, R.; Khan, J. Ethno-Medicinal Survey of Important Plants Practiced by Indigenous Community at Ladha Subdivision, South Waziristan Agency, Pakistan. *J Ethnobiol Ethnomed* **2016**, *12*, 53, doi:10.1186/S13002-016-0126-7.
77. Kayani, S.; Ahmad, M.; Zafar, M.; Sultana, S.; Khan, M.P.Z.; Ashraf, M.A.; Hussain, J.; Yaseen, G. Ethnobotanical Uses of Medicinal Plants for Respiratory Disorders among the Inhabitants of Gallies - Abbottabad, Northern Pakistan. *J Ethnopharmacol* **2014**, *156*, 47–60, doi:10.1016/J.JEP.2014.08.005.
78. Ullah, M.; Khan, M.U.; Mahmood, A.; Malik, R.N.; Hussain, M.; Wazir, S.M.; Daud, M.; Shinwari, Z.K. An Ethnobotanical Survey of Indigenous Medicinal Plants in Wana District South Waziristan Agency, Pakistan. *J Ethnopharmacol* **2013**, *150*, 918–924, doi:10.1016/J.JEP.2013.09.032.
79. Leila, J.; Ghassem, H.; Hamide, S. Introduction the Medicinal Species of Asteraceae Family in Ilkhji and Sharafaldin Regions of Esat Azarbaijan in Iran. *Journal of American Science* **2011**, *7*, 455–458.
80. Mobli, M.; Qaraaty, M.; Amin, G.; Haririan, I.; Hajimahmoodi, M.; Rahimi, R. Scientific Evaluation of Medicinal Plants Used for the Treatment of Abnormal Uterine Bleeding by Avicenna. *Arch Gynecol Obstet* **2015**, *292*, 21–35, doi:10.1007/S00404-015-3629-X.
81. Garsiya, E.R.; Konovalov, D.A.; Shamilov, A.A.; Glushko, M.P.; Orynbasarova, K.K. Traditional Medicine Plant, Onopordum Acanthium L. (Asteraceae): Chemical Composition and Pharmacological Research. *Plants 2019, Vol. 8, Page 40* **2019**, *8*, 40, doi:10.3390/PLANTS8020040.
82. Mamedov, N.; Mehdiyeva, N.P.; Lyle, E.Craker. Medicinal Plants Used in Traditional Medicine of the Caucasus and North America. *Journal of Medicinally Active Plants* **2015**, *4*, 42-66., doi:10.7275/R51834DS.
83. Gilca, M.; Tiplica, G.S.; Salavastru, C.M. Traditional and Ethnobotanical Dermatology Practices in Romania and Other Eastern European Countries. *Clin Dermatol* **2018**, *36*, 338–352, doi:10.1016/J.CLINDERMATOL.2018.03.008.

84. Mükemre, M.; Behçet, L.; Çakilcioılu, U. Ethnobotanical Study on Medicinal Plants in Villages of Çatak (Van-Turkey). *J Ethnopharmacol* **2015**, *166*, 361–374, doi:10.1016/J.JEP.2015.03.040.
85. Gras, A.; Garnatje, T.; Ibáñez, N.; López-Pujol, J.; Nualart, N.; Vallès, J. Medicinal Plant Uses and Names from the Herbarium of Francesc Bolòs (1773–1844). *J Ethnopharmacol* **2017**, *204*, 142–168, doi:10.1016/J.JEP.2017.04.002.
86. Mechaala, S.; Bouatrous, Y.; Adouane, S. Traditional Knowledge and Diversity of Wild Medicinal Plants in El Kantara's Area (Algerian Sahara Gate): An Ethnobotany Survey. *Acta Ecologica Sinica* **2022**, *42*, 33–45, doi:10.1016/J.CHNAES.2021.01.007.
87. Sharifi, N.; Souri, E.; Ziai, S.A.; Amin, G.; Amini, M.; Amanlou, M. Isolation, Identification and Molecular Docking Studies of a New Isolated Compound, from Onopordon Acanthium: A Novel Angiotensin Converting Enzyme (ACE) Inhibitor. *J Ethnopharmacol* **2013**, *148*, 934–939, doi:10.1016/J.JEP.2013.05.046.
88. Ghods, R.; Gharouni, M.; Amanlou, M.; Sharifi, N.; Ghobadi, A.; Amin, G. Effect of Onopordon Acanthium L. as Add on Antihypertensive Therapy in Patients with Primary Hypertension Taking Losartan: A Pilot Study. *Adv Pharm Bull* **2018**, *8*, 69–75, doi:10.15171/APB.2018.009.
89. Uzun, E.; Sariyar, G.; Adsersen, A.; Karakoc, B.; Ötük, G.; Oktayoglu, E.; Pirildar, S. Traditional Medicine in Sakarya Province (Turkey) and Antimicrobial Activities of Selected Species. *J Ethnopharmacol* **2004**, *95*, 287–296, doi:10.1016/J.JEP.2004.07.013.
90. Cocco, E.; Maccioni, D.; Sanjust, E.; Falconieri, D.; Farris, E.; Maxia, A. Ethnopharmacobotany and Diversity of Mediterranean Endemic Plants in Marmilla Subregion, Sardinia, Italy. *Plants* **2022**, *11*, doi:10.3390/PLANTS11223165.
91. Leonti, M.; Staub, P.O.; Cabras, S.; Castellanos, M.E.; Casu, L. From Cumulative Cultural Transmission to Evidence-Based Medicine: Evolution of Medicinal Plant Knowledge in Southern Italy. *Front Pharmacol* **2015**, *6*, 207, doi:10.3389/FPHAR.2015.00207.
92. Quan, N. Van; Xuan, T.D.; Teschke, R. Potential Hepatotoxins Found in Herbal Medicinal Products: A Systematic Review. *Int J Mol Sci* **2020**, *21*, 1–18, doi:10.3390/IJMS21145011.
93. Móricz, Á.M.; Jamshidi-Aidji, M.; Krüzselyi, D.; Darcsi, A.; Böszörményi, A.; Csontos, P.; Béni, S.; Ott, P.G.; Morlock, G.E. Distinction and Valorization of 30 Root Extracts of Five Goldenrod (Solidago) Species. *J Chromatogr A* **2020**, *1611*, doi:10.1016/J.CHROMA.2019.460602.
94. Móricz, Á.M.; Ott, P.G.; Häbe, T.T.; Darcsi, A.; Böszörményi, A.; Alberti, Á.; Krüzselyi, D.; Csontos, P.; Béni, S.; Morlock, G.E. Effect-Directed Discovery of Bioactive Compounds Followed by Highly Targeted Characterization, Isolation and Identification, Exemplarily Shown for Solidago Virgaurea. *Anal Chem* **2016**, *88*, 8202–8209, doi:10.1021/ACS.ANALCHEM.6B02007.

95. Borchert, V.E.; Czyborra, P.; Fetscher, C.; Goepel, M.; Michel, M.C. Extracts from Rhois Aromatica and Solidaginis Virgaurea Inhibit Rat and Human Bladder Contraction. *Naunyn Schmiedebergs Arch Pharmacol* **2004**, *369*, 281–286, doi:10.1007/S00210-004-0869-X.
96. Blum, H. Goldenrod Has Increasing Importance as a Herbal Drug. *Gemuse* **1999**, *35*, 431–432.
97. Tyszkiewicz, E. Assessment Report on Solidago Virgaurea L., Herba. *Med. Hum. Use* **2008**, 1–31.
98. Gross, S.C.; Goodarzi, G.; Watabe, M.; Bandyopadhyay, S.; Pai, S.K.; Watabe, K. Antineoplastic Activity of Solidago Virgaurea on Prostatic Tumor Cells in an SCID Mouse Model. *Nutr Cancer* **2002**, *43*, 76–81, doi:10.1207/S15327914NC431_9.
99. Melzig, M.F. Echtes Goldrutenkraut - Ein Klassiker in Der Urologischem Phytotherapie. *Wiener Medizinische Wochenschrift* **2004**, *154*, 523–527, doi:10.1007/S10354-004-0118-4.
100. Tariq, A.; Sadia, S.; Fan, Y.; Ali, S.; Amber, R.; Mussarat, S.; Ahmad, M.; Murad, W.; Zafar, M.; Adnan, M. Herbal Medicines Used to Treat Diabetes in Southern Regions of Pakistan and Their Pharmacological Evidence. *J Herb Med* **2020**, *21*, 100323, doi:10.1016/J.HERMED.2019.100323.
101. Kamau, L.N.; Mbaabu, M.P.; Mbaria, J.M.; Karuri, G.P.; Kiama, S.G. Knowledge and Demand for Medicinal Plants Used in the Treatment and Management of Diabetes in Nyeri County, Kenya. *J Ethnopharmacol* **2016**, *189*, 218–229, doi:10.1016/J.JEP.2016.05.021.
102. Chaachouay, N.; Benkhnigue, O.; Fadli, M.; El Ibaoui, H.; Zidane, L. Ethnobotanical and Ethnopharmacological Studies of Medicinal and Aromatic Plants Used in the Treatment of Metabolic Diseases in the Moroccan Rif. *Heliyon* **2019**, *5*, e02191, doi:10.1016/J.HELIYON.2019.E02191.
103. Cornara, L.; La Rocca, A.; Marsili, S.; Mariotti, M.G. Traditional Uses of Plants in the Eastern Riviera (Liguria, Italy). *J Ethnopharmacol* **2009**, *125*, 16–30, doi:10.1016/J.JEP.2009.06.021.
104. Njoroge, G.N.; Bussmann, R.W. Traditional Management of Ear, Nose and Throat (ENT) Diseases in Central Kenya. *J Ethnobiol Ethnomed* **2006**, *2*, doi:10.1186/1746-4269-2-54.
105. Atzei, A.D. *Domenico. Le Piante Nella Tradizione Popolare Della Sardegna: Documentazione Sugli Usi Alimentari, Aromatizzanti, Profumieri, Artigianali, Cosmetici, Medicinali, Veterinari, Magici, Ornamentali, Rituali, Religiosi, Tintori, Antiparassitari e Vari, Delle Piante.*; 2003;
106. Lentini, F.; Venza, F. Wild Food Plants of Popular Use in Sicily. *J Ethnobiol Ethnomed* **2007**, *3*, doi:10.1186/1746-4269-3-15.
107. Kujawska, M.; Schmeda-Hirschmann, G. The Use of Medicinal Plants by Paraguayan Migrants in the Atlantic Forest of Misiones, Argentina, Is Based on Guaraní Tradition, Colonial and Current Plant Knowledge. *J Ethnopharmacol* **2022**, *283*, doi:10.1016/J.JEP.2021.114702.

108. Karaköse, M. An Ethnobotanical Study of Medicinal Plants in Güce District, North-Eastern Turkey. *Plant Divers* **2022**, *44*, 577–597, doi:10.1016/J.PLD.2022.03.005.
109. Lans, C.; Turner, N.; Brauer, G.; Lourenco, G.; Georges, K. Ethnoveterinary Medicines Used for Horses in Trinidad and in British Columbia, Canada. *J Ethnobiol Ethnomed* **2006**, *2*, doi:10.1186/1746-4269-2-31.
110. Guarino, C.; De Simone, L.; Santoro, S. Ethnobotanical Study of the Sannio Area, Campania, Southern Italy. *Ethnobotany Research and Applications* **2008**, *6*, 255–317, doi:10.17348/ERA.6.0.255-317.
111. Rajaei, P.; research, N.M.-I. journal of pharmaceutical; 2012, undefined Ethnobotanical Study of Medicinal Plants of Hezar Mountain Allocated in South East of Iran. *ncbi.nlm.nih.govP Rajaei, N MohamadiIranian journal of pharmaceutical research: IJPR, 2012•ncbi.nlm.nih.gov*.
112. Basati, G.; Abbaszadeh, S.; Zebardast, A.; Teimouri, H. Analgesic Medicinal Plants in Shahrekord, Southwest of Iran: An Ethnobotanical Study. *Galen Medical Journal* **2019**, *8*, 1593, doi:10.31661/GMJ.V8I0.1593.
113. Andrade-Cetto, A. Ethnobotanical Study of the Medicinal Plants from Tlanchinol, Hidalgo, México. *J Ethnopharmacol* **2009**, *122*, 163–171, doi:10.1016/J.JEP.2008.12.008.
114. Bonet, M.À.; Vallès, J. Ethnobotany of Montseny Biosphere Reserve (Catalonia, Iberian Peninsula): Plants Used in Veterinary Medicine. *J Ethnopharmacol* **2007**, *110*, 130–147, doi:10.1016/J.JEP.2006.09.016.
115. ERUÇAR, F.; Tan, N.; Products, M.M.-R. of N.; 2023, undefined Ethnobotanical Records of Medicinal Plants of Turkey Effective on Stress Management Complied with the Literature Survey in Their Chemical Content and Activities. *avesis.istanbul.edu.trF ERUÇAR, N Tan, M MİSKİRecords of Natural Products, 2023•avesis.istanbul.edu.tr* **2022**, *17*, 1307–6167, doi:10.25135/rnp.324.2203.2372.
116. Karimi, M.; Mardani, M.; Mahmoodnia, L. Colic Phytotherapy in Iranian Ethnobotany: An Overview of the Ffectiveness of the Most Important Native Medicinal Plants of Iran on Olic Disease. *International Journal of Pharmaceutical and Clinical Research* **2017**, *9*, doi:10.25258/IJPCR.V9I1.8265.
117. Chaachouay, N.; Benkhnigue, O.; Fadli, M.; Ibaoui, H. El; Zidane, L. Ethnobotanical and Ethnopharmacological Studies of Medicinal and Aromatic Plants Used in the Treatment of Metabolic Diseases in the Moroccan Rif. *cell.comN Chaachouay, O Benkhnigue, M Fadli, H El Ibaoui, L ZidaneHeliyon, 2019•cell.com* **2017**, doi:10.1016/j.heliyon.2019.e02191.
118. Benkhnigue, O.; Akka, B.; Salhi, S.; Fadli, M.; Douira, A.; Zidane, L. Catalogue Des Plantes Médicinales Utilisées Dans Le Traitement Du Diabète Dans La Région d'Al Haouz-Rhamna (Maroc). *m.elewa.orgO Benkhnigue, FB Akka, S Salhi, M Fadli, A Douira, L ZidaneJ Anim Plant Sci, 2014•m.elewa.org* **2014**, *23*, 3539–3568.

119. Bouyahya, A.; El Omari, N.; Elmenyiy, N.; Guaouguaou, F.E.; Balahbib, A.; Belmehdi, O.; Salhi, N.; Imtara, H.; Mrabti, H.N.; El-Shazly, M.; et al. Moroccan Antidiabetic Medicinal Plants: Ethnobotanical Studies, Phytochemical Bioactive Compounds, Preclinical Investigations, Toxicological Validations and Clinical Evidences; Challenges, Guidance and Perspectives for Future Management of Diabetes Worldwide. *Trends Food Sci Technol* **2021**, *115*, 147–254, doi:10.1016/J.TIFS.2021.03.032.

120. Azimova, S.S.; Glushenkova, A.I. Tanacetum Vulgare L. *Lipids, Lipophilic Components and Essential Oils from Plant Sources* **2012**, 138–139, doi:10.1007/978-0-85729-323-7_453.

121. Singh, R.; De, S.; Belkheir, A. Avena Sativa (Oat), A Potential Neutraceutical and Therapeutic Agent: An Overview. *Crit Rev Food Sci Nutr* **2013**, *53*, 126–144, doi:10.1080/10408398.2010.526725.

122. Wybenga, W. Wildflowers of Lesotho's Tséhlanyane National Park. *Veld & Flora* **2006**, *92*, 81–85.

123. Moffett, R. Sesotho Plant and Animal Names and Plants Used by the Basotho. **2010**.

124. Moteetee, A.; Moffett, R.O.; Seleteng-Kose, L. A Review of the Ethnobotany of the Basotho of Lesotho and the Free State Province of South Africa (South Sotho). *South African Journal of Botany* **2019**, *122*, 21–56, doi:10.1016/J.SAJB.2017.12.012.

125. Ngorima, A.; Shackleton, C.M. Livelihood Benefits and Costs from an Invasive Alien Tree (Acacia Dealbata) to Rural Communities in the Eastern Cape, South Africa. *J Environ Manage* **2019**, *229*, 158–165, doi:10.1016/J.JENVMAN.2018.05.077.

126. Narayan Das Prajapati; U. Kumar *Agros Dictionary of Medicinal Plants*; (India) Jodhpur, 2005;

127. Tsioutsiou, E.E.; Amountzias, V.; Vontzalidou, A.; Dina, E.; Stevanović, Z.D.; Cheilari, A.; Aligiannis, N. Medicinal Plants Used Traditionally for Skin Related Problems in the South Balkan and East Mediterranean Region—A Review. *Front Pharmacol* **2022**, *13*, 1, doi:10.3389/FPHAR.2022.936047.

128. Chaachouay, N.; Douira, A.; Zidane, L. Herbal Medicine Used in the Treatment of Human Diseases in the Rif, Northern Morocco. *Arab J Sci Eng* **2022**, *47*, 131, doi:10.1007/S13369-021-05501-1.

129. Amiri, M.S.; Joharchi, M.R. Ethnobotanical Investigation of Traditional Medicinal Plants Commercialized in the Markets of Mashhad, Iran. *Avicenna J Phytomed* **2013**, *3*, 254.

130. Moradi, M.T.; Asadi-Samani, M.; Bahmani, M.; Shahrani, M. Medicinal Plants Used for Liver Disorders Based on the Ethnobotanical Documents of Iran: A Review Study on Antimicrobial Property of Essential Oils from Medicinal Plants View Project Evidence Base of the Concepts of Basic Terminology of Persian and Chinese Medicine View Project.

131. Andrade, C.; Gomes, N.G.M.; Duangsrisai, S.; Andrade, P.B.; Pereira, D.M.; Valentão, P. Medicinal Plants Utilized in Thai Traditional Medicine for Diabetes Treatment: Ethnobotanical Surveys, Scientific Evidence and Phytochemicals. *J Ethnopharmacol* **2020**, *263*, 113177, doi:10.1016/J.JEP.2020.113177.

132. Chaachouay, N.; Douira, A.; Zidane, L. Herbal Medicine Used in the Treatment of Human Diseases in the Rif, Northern Morocco. *Arab J Sci Eng* **2022**, *47*, 131, doi:10.1007/S13369-021-05501-1.
133. Mehrnia, M.; Akaberi, M.; Amiri, M.S.; Nadaf, M.; Emami, S.A. Ethnopharmacological Studies of Medicinal Plants in Central Zagros, Lorestan Province, Iran. *J Ethnopharmacol* **2021**, *280*, 114080, doi:10.1016/J.JEP.2021.114080.
134. Fernanda M. Ferreira; Francisco P. Peixoto; Raquel Seiçad; Maria S. Santos Diabetes and Medicinal Plants in Portugal. In *Natural Products: Research Reviews Vol. 1* ; V. K. Gupta, Ed.; 2012; pp. 1–41.
135. Rivera, D.; Verde, A.; Fajardo, J.; Obón, C.; Consuegra, V.; García-Botía, J.; Ríos, S.; Alcaraz, F.; Valdés, A.; Moral, A. del; et al. Ethnopharmacology in the Upper Guadiana River Area (Castile-La Mancha, Spain). *J Ethnopharmacol* **2019**, *241*, 111968, doi:10.1016/J.JEP.2019.111968.
136. Camejo-Rodrigues, J.; Ascensão, L.; Bonet, M.À.; Vallès, J. An Ethnobotanical Study of Medicinal and Aromatic Plants in the Natural Park of "Serra de São Mamede" (Portugal). *J Ethnopharmacol* **2003**, *89*, 199–209, doi:10.1016/S0378-8741(03)00270-8.
137. Neves, J.M.; Matos, C.; Moutinho, C.; Queiroz, G.; Gomes, L.R. Ethnopharmacological Notes about Ancient Uses of Medicinal Plants in Trás-Os-Montes (Northern of Portugal). *J Ethnopharmacol* **2009**, *124*, 270–283, doi:10.1016/J.JEP.2009.04.041.
138. Frazao-Moreira Ana Maria Ca rvalho, A.; OlTtcnologia, ciJ; Antropologia, U.E. Etnobotânica Do Nordeste Português: Saberes, Plantas e Usos. *Etnobotânica do Nordeste Português: saberes, plantas e usos* **2009**.
139. Tahraoui, A.; El-Hilaly, J.; Israili, Z.; of, B.L.-J.; 2007, undefined Ethnopharmacological Survey of Plants Used in the Traditional Treatment of Hypertension and Diabetes in South-Eastern Morocco (Errachidia Province). *Elsevier*.
140. Bellakhdar, J. Contribution à l'étude de La Pharmacopée Traditionnelle Au Maroc : La Situation Actuelle, Les Produits, Les Sources Du Savoir (Enquête Ethnopharmacologique de Terrain Réalisée de 1969 à 1992). **1997**.
141. Benítez Cruz, G. *El Uso de Las Plantas a Través de La Cultura Tradicional Lojeña: Una Perspectiva Etnobotánica*; FUNDACION IBN AL-JATIB DE ESTUDIOS Y, Ed.; Fundación Ibn al-Jatib, 2007; ISBN 9788493426439.
142. Benítez, G.; González-Tejero, M.R.; Molero-Mesa, J. Pharmaceutical Ethnobotany in the Western Part of Granada Province (Southern Spain): Ethnopharmacological Synthesis. *J Ethnopharmacol* **2010**, *129*, 87–105, doi:10.1016/J.JEP.2010.02.016.
143. Mouhajir, F. Medicinal Plants Used by Berber and Arab Peoples of Morocco: Ethnopharmacology and Phytochemistry, University of British Columbia: Vancouver, 2002.
144. Louaar, S.; Akkal, S.; … A.B.-C. of natural; 2005, undefined Phytochemical Study of Retama Sphaerocarpa. *researchgate.netS Louaar, S Akkal, A Bousetla, K Medjroubi, L Djarri, E SeguinChemistry of natural compounds, 2005•researchgate.net* **2005**, *41*, 107–108, doi:10.1007/s10600-005-0091-1.

145. Guarrera, P.M. Food Medicine and Minor Nourishment in the Folk Traditions of Central Italy (Marche, Abruzzo and Latium). *Fitoterapia* **2003**, *74*, 515–544, doi:10.1016/S0367-326X(03)00122-9.
146. Janaćković, P.; Gavrilović, M.; Miletić, M.; Radulović, M.; Kolašinac, S.; Stevanović, Z.D. Small Regions as Key Sources of Traditional Knowledge: A Quantitative Ethnobotanical Survey in the Central Balkans. *J Ethnobiol Ethnomed* **2022**, *18*, 70, doi:10.1186/S13002-022-00566-0.
147. Tsioutsiou, E.E.; Giordani, P.; Hanlidou, E.; Biagi, M.; De Feo, V.; Cornara, L. Ethnobotanical Study of Medicinal Plants Used in Central Macedonia, Greece. *Evid Based Complement Alternat Med* **2019**, *2019*, 22, doi:10.1155/2019/4513792.
148. Shaheen, H.; Qaseem, M.F.; Amjad, M.S.; Bruschi, P. Exploration of Ethno-Medicinal Knowledge among Rural Communities of Pearl Valley; Rawalakot, District Poonch Azad Jammu and Kashmir. *PLoS One* **2017**, *12*, doi:10.1371/JOURNAL.PONE.0183956.
149. Bahmani, M.; Zargaran, A.; Rafieian-Kopaei, M.; Saki, K. Ethnobotanical Study of Medicinal Plants Used in the Management of Diabetes Mellitus in the Urmia, Northwest Iran. *Asian Pac J Trop Med* **2014**, *7*, S348–S354, doi:10.1016/S1995-7645(14)60257-1.
150. Booth, N.L.; Overk, C.R.; Yao, P.; Burdette, J.E.; Nikolic, D.; Chen, S.N.; Bolton, J.L.; Van Breemen, R.B.; Pauli, G.F.; Farnsworth, N.R. The Chemical and Biologic Profile of a Red Clover (Trifolium Pratense L.) Phase II Clinical Extract. *Journal of Alternative and Complementary Medicine* **2006**, *12*, 133–139, doi:10.1089/ACM.2006.12.133.
151. Duke, J. Handbook of Medicinal Herbs. **2002**.
152. Cosentino, M.; Marino, F.; Rasini, E.; Legnaro, M.; Bombelli, R.; Luini, A.; Pacchetti, B. Improved Solubility and Increased Biological Activity of NeoSol™RCL40, a Novel Red Clover Isoflavone Aglycones Extract Preparation. *Biomedicine & Pharmacotherapy* **2019**, *111*, 91–98, doi:10.1016/J.BIOPHA.2018.12.065.
153. Malca-Garcia, G.R.; Zagal, D.; Graham, J.; Nikolić, D.; Friesen, J.B.; Lankin, D.C.; Chen, S.N.; Pauli, G.F. Dynamics of the Isoflavone Metabolome of Traditional Preparations of Trifolium Pratense L. *J Ethnopharmacol* **2019**, *238*, 111865, doi:10.1016/J.JEP.2019.111865.
154. Malca-Garcia, G.R.; Zagal, D.; Graham, J.; Nikolić, D.; Friesen, J.B.; Lankin, D.C.; Chen, S.N.; Pauli, G.F. Dynamics of the Isoflavone Metabolome of Traditional Preparations of Trifolium Pratense L. *J Ethnopharmacol* **2019**, *238*, doi:10.1016/J.JEP.2019.111865.
155. Cosentino, M.; Marino, F.; Rasini, E.; Legnaro, M.; Bombelli, R.; Luini, A.; Pacchetti, B. Improved Solubility and Increased Biological Activity of NeoSol ™ RCL40, a Novel Red Clover Isoflavone Aglycones Extract Preparation. *Biomedicine and Pharmacotherapy* **2019**, *111*, 91–98, doi:10.1016/J.BIOPHA.2018.12.065.

156. Vitalini, S.; Puricelli, C.; Mikerezi, I.; Iriti, M. Plants, People and Traditions: Ethnobotanical Survey in the Lombard Stelvio National Park and Neighbouring Areas (Central Alps, Italy). *J Ethnopharmacol* **2015**, *173*, 435–458, doi:10.1016/J.JEP.2015.05.036.
157. Šarić-Kundalić, B.; Dobeš, C.; Klatte-Asselmeyer, V.; Saukel, J. Ethnobotanical Survey of Traditionally Used Plants in Human Therapy of East, North and North-East Bosnia and Herzegovina. *J Ethnopharmacol* **2011**, *133*, 1051–1076, doi:10.1016/J.JEP.2010.11.033.
158. Šarić-Kundalić, B.; Dobeš, C.; Klatte-Asselmeyer, V.; Saukel, J. Ethnobotanical Study on Medicinal Use of Wild and Cultivated Plants in Middle, South and West Bosnia and Herzegovina. *J Ethnopharmacol* **2010**, *131*, 33–55, doi:10.1016/J.JEP.2010.05.061.
159. Hachi, M.; Ouafae, B.; Hachi, T.; Mohamed, E.; Lazaroa, B.I.-; 2016, undefined Contribution to the Ethnobotanical Study of Antidiabetic Medicinal Plants of the Central Middle Atlas Region (Morocco). *researchgate.netM Hachi, B Ouafae, T Hachi, EB Mohamed, B Imane, R Atmane, L ZidaneLazaroa, 2016•researchgate.net* **2016**, *37*, 135–144, doi:10.5209/LAZAROA.51854.
160. Bencheikh, N.; Elbouzidi, A.; Kharchoufa, L.; Ouassou, H.; Merrouni, I.A.; Mechchate, H.; Es-Safi, I.; Hano, C.; Addi, M.; Bouhrim, M.; et al. Inventory of Medicinal Plants Used Traditionally to Manage Kidney Diseases in North-Eastern Morocco: Ethnobotanical Fieldwork and Pharmacological Evidence. *Plants* **2021**, *10*, doi:10.3390/PLANTS10091966.
161. Cocco, E.; Maccioni, D.; Sanjust, E.; Falconieri, D.; Farris, E.; Maxia, A. Ethnopharmacobotany and Diversity of Mediterranean Endemic Plants in Marmilla Subregion, Sardinia, Italy. *Plants* **2022**, *11*, doi:10.3390/PLANTS11223165.
162. Es-Safi, I.; Mechchate, H.; Amaghnouje, A.; Jawhari, F.Z.; Bari, A.; Cerruti, P.; Avella, M.; Grafov, A.; Bousta, D. Medicinal Plants Used to Treat Acute Digestive System Problems in the Region of Fez-Meknes in Morocco: An Ethnopharmacological Survey. *Ethnobotany Research and Applications* **2020**, *20*, 1–14.
163. Benkhnigue, O.; Akka, B.; Salhi, S.; Fadli, M.; Douira, A.; Zidane, L. Catalogue Des Plantes Médicinales Utilisées Dans Le Traitement Du Diabète Dans La Région d'Al Haouz-Rhamna (Maroc). *Journal of Animal &Plant Sciences* **2014**, *23*, 3539–3568.
164. Benkhnigue, O.; Chaachouay, N.; Khamar, H.; Azzouzi, F. El; Douira, A.; Zidane, L. Ethnobotanical and Ethnopharmacological Study of Medicinal Plants Used in the Treatment of Anemia in the Region of Haouz-Rehamna (Morocco). *J Pharm Pharmacogn Res* **2022**, *10*, 279–302.
165. Noureddine, B.; Mostafa, E.; Mandal, S.C. Ethnobotanical, Pharmacological, Phytochemical, and Clinical Investigations on Moroccan Medicinal Plants Traditionally Used for the Management of Renal Dysfunctions. *J Ethnopharmacol* **2022**, *292*, 115178, doi:10.1016/J.JEP.2022.115178.

166. El-Hilaly, J.; Hmammouchi, M.; Lyoussi, B. Ethnobotanical Studies and Economic Evaluation of Medicinal Plants in Taounate Province (Northern Morocco). *J Ethnopharmacol* **2003**, *86*, 149–158, doi:10.1016/S0378-8741(03)00012-6.
167. Boubaker, H.; Katiri, A.; Barkaoui, M.; Msanda, F. Ethnobotanical Survey of Medicinal Plants Used for the Treatment of Diabetes in the Tizi n'Test Region (Taroudant Province, Morocco). *researchgate.netA Katiri, M Barkaoui, F Msanda, H BoubakerJ pharmacogn nat prod, 2017•researchgate.net* **2017**, *3*, 1, doi:10.4172/2472-0992.1000130.
168. Khatib, C.; Nattouf, A.; Hasan Agha, M.I. Traditional Medicines and Their Common Uses in Central Region of Syria: Hama and Homs – an Ethnomedicinal Survey. *Pharm Biol* **2021**, *59*, 776, doi:10.1080/13880209.2021.1936078.
169. Saad, B.; Kmail, A.; Haq, S.Z.H. Anti-Diabesity Middle Eastern Medicinal Plants and Their Action Mechanisms. *Evid Based Complement Alternat Med* **2022**, *2022*, doi:10.1155/2022/2276094.
170. Shikov, A.N.; Narkevich, I.A.; Akamova, A. V.; Nemyatykh, O.D.; Flisyuk, E. V.; Luzhanin, V.G.; Povydysh, M.N.; Mikhailova, I. V.; Pozharitskaya, O.N. Medical Species Used in Russia for the Management of Diabetes and Related Disorders. *Front Pharmacol* **2021**, *12*, doi:10.3389/FPHAR.2021.697411.
171. Marie, L.; Totelin, V. Hippocratic Recipes: Oral and Written Transmission of Pharmacological Knowledge in Fifth-and Fourth-Century Greece. **2009**.
172. Aliotta, G.; Piomelli, D.; Pollio, A.; Touwaide, A. Le Piante Medicinali Del "Corpus Hippocraticum." **2003**.
173. Bouasla, A.; Bouasla, I. Ethnobotanical Survey of Medicinal Plants in Northeastern of Algeria. *Phytomedicine* **2017**, *36*, 68–81, doi:10.1016/j.phymed.2017.09.007.
174. González-Tejero, M.R.; Casares-Porcel, M.; Sánchez-Rojas, C.P.; Ramiro-Gutiérrez, J.M.; Molero-Mesa, J.; Pieroni, A.; Giusti, M.E.; Censorii, E.; de Pasquale, C.; Della, A.; et al. Medicinal Plants in the Mediterranean Area: Synthesis of the Results of the Project Rubia. *J Ethnopharmacol* **2008**, *116*, 341–357, doi:10.1016/J.JEP.2007.11.045.
175. Prajapati, R.; Kalariya, M.; Umbarkar, R.; Parmar, S.; Sheth, N. Colocasia Esculenta: A Potent Indigenous Plant. *Int J Nutr Pharmacol Neurol Dis* **2011**, *1*, 90, doi:10.4103/2231-0738.84188.
176. Ali, H.; Sannai, J.; Sher, H.; Rashid, A. Ethnobotanical Profile of Some Plant Resources in Malam Jabba Valley of Swat, Pakistan. *Journal of Medicinal Plants Research* **2011**, *5*, 4676–4687.
177. Munir, M.; Qureshi, R. Antidiabetic Plants of Pakistan. *Plant and Human Health, Volume 1* **2018**, 463–545, doi:10.1007/978-3-319-93997-1_12.
178. Chidrawar, V.; Bothra, S.; Koli, A.; Patel, K.; Shiromwar, S.; Kalyankar, G. Anti-Obesity Effect of Stellaria Media Methanolic Extract in the Murine Model of Cafeteria Diet Induced Obesity. *Int J Nutr Pharmacol Neurol Dis* **2012**, *2*, 121, doi:10.4103/2231-0738.95963.

179. Shinwari, M.I.; Khan, M.A. Folk Use of Medicinal Herbs of Margalla Hills National Park, Islamabad. *J Ethnopharmacol* **2000**, *69*, 45–56, doi:10.1016/S0378-8741(99)00135-X.
180. Kumar, K.; Sharma, Y.P.; Manhas, R.K.; Bhatia, H. Ethnomedicinal Plants of Shankaracharya Hill, Srinagar, J&K, India. *J Ethnopharmacol* **2015**, *170*, 255–274, doi:10.1016/J.JEP.2015.05.021.
181. Rahman, I.U.; Ijaz, F.; Iqbal, Z.; Afzal, A.; Ali, N.; Afzal, M.; Khan, M.A.; Muhammad, S.; Qadir, G.; Asif, M. A Novel Survey of the Ethno Medicinal Knowledge of Dental Problems in Manoor Valley (Northern Himalaya), Pakistan. *J Ethnopharmacol* **2016**, *194*, 877–894, doi:10.1016/J.JEP.2016.10.068.
182. Maqsood, T.; Munawar, T.; Bibi, Y.; El Askary, A.; Gharib, A.F.; Elmissbah, T.E.; Elesawy, B.H.; Qayyum, A. Study of Plant Resources with Ethnomedicinal Relevance from District Bagh, Azad Jammu and Kashmir, Pakistan. *Open Chem* **2022**, *20*, 146–162, doi:10.1515/CHEM-2022-0129/ASSET/GRAPHIC/J_CHEM-2022-0129_FIG_006.JPG.
183. Shikov, A.N.; Tsitsilin, A.N.; Pozharitskaya, O.N.; Makarov, V.G.; Heinrich, M. Traditional and Current Food Use of Wild Plants Listed in the Russian Pharmacopoeia. *Front Pharmacol* **2017**, *8*, 841, doi:10.3389/FPHAR.2017.00841.
184. Güler, B.; Manav, E.; Uıurlu, E. Medicinal Plants Used by Traditional Healers in Bozüyük (Bilecik–Turkey). *J Ethnopharmacol* **2015**, *173*, 39–47, doi:10.1016/J.JEP.2015.07.007.
185. Danna, C.; Poggio, L.; Smeriglio, A.; Mariotti, M.; Cornara, L. Ethnomedicinal and Ethnobotanical Survey in the Aosta Valley Side of the Gran Paradiso National Park (Western Alps, Italy). *Plants* **2022**, *11*, 170, doi:10.3390/PLANTS11020170/S1.
186. Kharchoufa, L.; Merrouni, I.A.; Yamani, A.; Elachouri, M. Profile on Medicinal Plants Used by the People of North Eastern Morocco: Toxicity Concerns. *Toxicon* **2018**, *154*, 90–113, doi:10.1016/J.TOXICON.2018.09.003.
187. Leporatti, M.L.; Ghedira, K. Comparative Analysis of Medicinal Plants Used in Traditional Medicine in Italy and Tunisia. *J Ethnobiol Ethnomed* **2009**, *5*, 31, doi:10.1186/1746-4269-5-31.
188. La Rosa, A.; Cornara, L.; Saitta, A.; Salam, A.M.; Grammatico, S.; Caputo, M.; La Mantia, T.; Quave, C.L. Ethnobotany of the Aegadian Islands: Safeguarding Biocultural Refugia in the Mediterranean. *J Ethnobiol Ethnomed* **2021**, *17*, doi:10.1186/S13002-021-00470-Z.
189. Guarrera, P.M.; Savo, V. Perceived Health Properties of Wild and Cultivated Food Plants in Local and Popular Traditions of Italy: A Review. *J Ethnopharmacol* **2013**, *146*, 659–680, doi:10.1016/J.JEP.2013.01.036.
190. Guarrera, P.M. Food Medicine and Minor Nourishment in the Folk Traditions of Central Italy (Marche, Abruzzo and Latium). *Fitoterapia* **2003**, *74*, 515–544, doi:10.1016/S0367-326X(03)00122-9.

191. Batool, Z.; Singh, K.; Gairola, S. Medicinal Plants Traditionally Used in the Health Care Practices by the Indigenous Communities of the Trans-Himalayan Region of Ladakh, India. *J Ethnopharmacol* **2023**, *317*, 116837, doi:10.1016/J.JEP.2023.116837.
192. Amrati, F.E.Z.; Bourhia, M.; Slighoua, M.; Mohammad Salamatullah, A.; Alzahrani, A.; Ullah, R.; Bari, A.; Bousta, D. Traditional Medicinal Knowledge of Plants Used for Cancer Treatment by Communities of Mountainous Areas of Fez-Meknes-Morocco. *Saudi Pharmaceutical Journal* **2021**, *29*, 1185–1204, doi:10.1016/J.JSPS.2021.09.005.
193. Polat, R.; Satil, F. An Ethnobotanical Survey of Medicinal Plants in Edremit Gulf (Balıkesir – Turkey). *J Ethnopharmacol* **2012**, *139*, 626–641, doi:10.1016/J.JEP.2011.12.004.
194. Marles, R.J.; Farnsworth, N.R. Antidiabetic Plants and Their Active Constituents. *Phytomedicine* **1995**, *2*, 137–189, doi:10.1016/S0944-7113(11)80059-0.
195. Ahn, D.K. *Illustrated Book of Korean Medicinal Herbs*; Kyo-hak Publishing Co, Ed.; Seoul, 1998; Vol. 107;.
196. Hernawati, D.; Putra, R.R.; Meylani, V. Indigenous Vegetables Consumed as Lalapan by a Sundanese Ethnic Group in West Java, Indonesia: Potential, Traditions, Local Knowledge, and It's Future. *South African Journal of Botany* **2022**, *151*, 133–145, doi:10.1016/J.SAJB.2022.09.007.
197. Dei Cas, L.; Pugni, F.; Fico, G. Tradition of Use on Medicinal Species in Valfurva (Sondrio, Italy). *J Ethnopharmacol* **2015**, *163*, 113–134, doi:10.1016/J.JEP.2014.12.054.
198. Tobyn, G.; Denham, A.; Whitelegg, M. Daucus Carota, Wild Carrot. *Medical Herbs* **2011**, 145–154, doi:10.1016/B978-0-443-10344-5.00019-7.
199. Guarrera, P.M. Food Medicine and Minor Nourishment in the Folk Traditions of Central Italy (Marche, Abruzzo and Latium). *Fitoterapia* **2003**, *74*, 515–544, doi:10.1016/S0367-326X(03)00122-9.
200. Gilca, M.; Tiplica, G.S.; Salavastru, C.M. Traditional and Ethnobotanical Dermatology Practices in Romania and Other Eastern European Countries. *Clin Dermatol* **2018**, *36*, 338–352, doi:10.1016/J.CLINDERMATOL.2018.03.008.
201. Lentini, F.; Venza, F. Wild Food Plants of Popular Use in Sicily. *J Ethnobiol Ethnomed* **2007**, *3*, doi:10.1186/1746-4269-3-15.
202. Guarrera, P.M.; Savo, V. Perceived Health Properties of Wild and Cultivated Food Plants in Local and Popular Traditions of Italy: A Review. *J Ethnopharmacol* **2013**, *146*, 659–680, doi:10.1016/J.JEP.2013.01.036.
203. Tuttolomondo, T.; Licata, M.; Leto, C.; Savo, V.; Bonsangue, G.; Letizia Gargano, M.; Venturella, G.; La Bella, S. Ethnobotanical Investigation on Wild Medicinal Plants in the Monti Sicani Regional Park (Sicily, Italy). *J Ethnopharmacol* **2014**, *153*, 568–586, doi:10.1016/J.JEP.2014.02.032.

204. Guarrera, P.M. Traditional Phytotherapy in Central Italy (Marche, Abruzzo, and Latium). *Fitoterapia* **2005**, *76*, 1–25, doi:10.1016/J.FITOTE.2004.09.006.

205. González-Tejero, M.R.; Casares-Porcel, M.; Sánchez-Rojas, C.P.; Ramiro-Gutiérrez, J.M.; Molero-Mesa, J.; Pieroni, A.; Giusti, M.E.; Censorii, E.; de Pasquale, C.; Della, A.; et al. Medicinal Plants in the Mediterranean Area: Synthesis of the Results of the Project Rubia. *J Ethnopharmacol* **2008**, *116*, 341–357, doi:10.1016/J.JEP.2007.11.045.

206. Pourali Kahriz, M.; Pourali Kahriz, P.; Ahmed, F.; Khawar, K.M. An Introduction to Herbal Medicines and Palliative Care of Cancer Patients and Related Diseases through Times in Turkey. *Herbal Medicines: A Boon for Healthy Human Life* **2022**, 579–590, doi:10.1016/B978-0-323-90572-5.00004-4.

207. Akgul, A.; Akgul, A.; Senol, S.G.; Yildirim, H.; Secmen, O.; Dogan, Y. An Ethnobotanical Study in Midyat (Turkey), a City on the Silk Road Where Cultures Meet. *J Ethnobiol Ethnomed* **2018**, *14*, doi:10.1186/S13002-017-0201-8.

208. Zovko Končić, M.; Bljajić, K. Traditional Herbal Products Used for the Management of Diabetes in Croatia: Linking Traditional Use With α-Glucosidase-Inhibitory Activity. *Bioactive Food as Dietary Interventions for Diabetes* **2019**, 647–664, doi:10.1016/B978-0-12-813822-9.00042-4.

209. Jadid, N.; Widodo, A.F.; Ermavitalini, D.; Sa'adah, N.N.; Gunawan, S.; Nisa, C. The Medicinal Umbelliferae Plant Fennel (Foeniculum Vulgare Mill.): Cultivation, Traditional Uses, Phytopharmacological Properties, and Application in Animal Husbandry. *Arabian Journal of Chemistry* **2023**, *16*, 104541, doi:10.1016/J.ARABJC.2023.104541.

210. Eddouks, M.; Maghrani, M.; Lemhadri, A.; Ouahidi, M.L.; Jouad, H. Ethnopharmacological Survey of Medicinal Plants Used for the Treatment of Diabetes Mellitus, Hypertension and Cardiac Diseases in the South-East Region of Morocco (Tafilalet). *J Ethnopharmacol* **2002**, *82*, 97–103, doi:10.1016/S0378-8741(02)00164-2.

211. Vitalini, S.; Puricelli, C.; Mikerezi, I.; Iriti, M. Plants, People and Traditions: Ethnobotanical Survey in the Lombard Stelvio National Park and Neighbouring Areas (Central Alps, Italy). *J Ethnopharmacol* **2015**, *173*, 435–458, doi:10.1016/J.JEP.2015.05.036.

212. Dei Cas, L.; Pugni, F.; Fico, G. Tradition of Use on Medicinal Species in Valfurva (Sondrio, Italy). *J Ethnopharmacol* **2015**, *163*, 113–134, doi:10.1016/J.JEP.2014.12.054.

213. Jarić, S.; MačUkanović-Jocić, M.; Djurdjević, L.; Mitrović, M.; Kostić, O.; Karadžić, B.; Pavlović, P. An Ethnobotanical Survey of Traditionally Used Plants on Suva Planina Mountain (South-Eastern Serbia). *J Ethnopharmacol* **2015**, *175*, 93–108, doi:10.1016/J.JEP.2015.09.002.

214. Leporatti, M.L.; Ivancheva, S. Preliminary Comparative Analysis of Medicinal Plants Used in the Traditional Medicine of Bulgaria and Italy. *J Ethnopharmacol* **2003**, *87*, 123–142, doi:10.1016/S0378-8741(03)00047-3.

215. Skalli, S.; Hassikou, R.; Arahou, M. An Ethnobotanical Survey of Medicinal Plants Used for Diabetes Treatment in Rabat, Morocco. *Heliyon* **2019**, *5*, doi:10.1016/J.HELIYON.2019.E01421.
216. Ghourri, M.; Zidane, L.; Douira, A. Catalogue Des Plantes Médicinales Utilisées Dans Le Traitement de La Lithiase Rénale Dans La Province de Tan-Tan (Maroc Saharien). *Int J Biol Chem Sci* **2014**, *7*, 1688, doi:10.4314/IJBCS.V7I4.24.
217. Benítez, G.; Molero-Mesa, J.; González-Tejero, M.R. Wild Edible Plants of Andalusia: Traditional Uses and Potential of Eating Wild in a Highly Diverse Region. *Plants* **2023**, *12*, doi:10.3390/PLANTS12061218/S1.
218. Khera, N.; Life, A.B.-I.J. of P.&; 2014, undefined MEDICINAL PLANTS AS NATURAL ANTI-DIABETIC AGENTS. *search.ebscohost.comN Khera, A BhatiaInternational Journal of Pharmacy & Life Sciences, 2014•search.ebscohost.com*.
219. Motti, R. Wild Plants Used as Herbs and Spices in Italy: An Ethnobotanical Review. *Plants* **2021**, *10*, 1–14, doi:10.3390/PLANTS10030563.
220. Mechchate, H.; Es-Safi, I.; Jawhari, F.Z.; Bari, A.; Grafov, A.; Bousta, D. Ethnobotanical Survey about the Management of Diabetes with Medicinal Plants Used by Diabetic Patients in Region of Fez- Meknes, Morocco. *Ethnobotany Research and Applications* **2020**, *19*, doi:10.32859/ERA.19.12.1-28.
221. Kachmar, M.R.; Naceiri Mrabti, H.; Bellahmar, M.; Ouahbi, A.; Haloui, Z.; El Badaoui, K.; Bouyahya, A.; Chakir, S. Traditional Knowledge of Medicinal Plants Used in the Northeastern Part of Morocco. *Evid Based Complement Alternat Med* **2021**, *2021*, doi:10.1155/2021/6002949.
222. Tagarelli, G.; Tagarelli, A.; Piro, A. Folk Medicine Used to Heal Malaria in Calabria (Southern Italy). *J Ethnobiol Ethnomed* **2010**, *6*, 27, doi:10.1186/1746-4269-6-27.
223. Sargin, S.A. Potential Anti-Influenza Effective Plants Used in Turkish Folk Medicine: A Review. *J Ethnopharmacol* **2021**, *265*, 113319, doi:10.1016/J.JEP.2020.113319.
224. Soković, M.; Petar, •; Marin, D.; Brkić, D.; Leo, •; Van Griensven, J.L.D. Chemical Composition and Antibacterial Activity of Essential Oils of Ten Aromatic Plants against Human Pathogenic Bacteria.
225. Sahranavard, S.; Ghafari, S.; Mosaddegh, M. Medicinal Plants Used in Iranian Traditional Medicine to Treat Epilepsy. *Seizure* **2014**, *23*, 328–332, doi:10.1016/J.SEIZURE.2014.01.013.
226. Cocco, E.; Maccioni, D.; Sanjust, E.; Falconieri, D.; Farris, E.; Maxia, A. Ethnopharmacobotany and Diversity of Mediterranean Endemic Plants in Marmilla Subregion, Sardinia, Italy. *Plants* **2022**, *11*, doi:10.3390/PLANTS11223165.
227. Ez zoubi, Y.; Bousta, D.; Farah, A. A Phytopharmacological Review of a Mediterranean Plant: Lavandula Stoechas L. *Clinical Phytoscience* **2020**, *6*, doi:10.1186/S40816-019-0142-Y.

228. Miraj, S.; Rafieian-Kopaei; Kiani, S. Melissa Officinalis L: A Review Study With an Antioxidant Prospective. *J Evid Based Complementary Altern Med* **2017**, *22*, 385, doi:10.1177/2156587216663433.
229. Alarcón, R.; Pardo-De-Santayana, M.; Priestley, C.; Morales, R.; Heinrich, M. Medicinal and Local Food Plants in the South of Alava (Basque Country, Spain). *J Ethnopharmacol* **2015**, *176*, 207, doi:10.1016/J.JEP.2015.10.022.
230. Sargin, S.A. Potential Anti-Influenza Effective Plants Used in Turkish Folk Medicine: A Review. *J Ethnopharmacol* **2021**, *265*, 113319, doi:10.1016/J.JEP.2020.113319.
231. Janaćković, P.; Gavrilović, M.; Miletić, M.; Radulović, M.; Kolašinac, S.; Stevanović, Z.D. Small Regions as Key Sources of Traditional Knowledge: A Quantitative Ethnobotanical Survey in the Central Balkans. *J Ethnobiol Ethnomed* **2022**, *18*, 70, doi:10.1186/S13002-022-00566-0.
232. Cocco, E.; Maccioni, D.; Sanjust, E.; Falconieri, D.; Farris, E.; Maxia, A. Ethnopharmacobotany and Diversity of Mediterranean Endemic Plants in Marmilla Subregion, Sardinia, Italy. *Plants* **2022**, *11*, doi:10.3390/PLANTS11223165.
233. Bounihi, A.; Hajjaj, G.; Alnamer, R.; Cherrah, Y.; Zellou, A. In Vivo Potential Anti-Inflammatory Activity of Melissa Officinalis l. Essential Oil. *Adv Pharmacol Sci* **2013**, *2013*, doi:10.1155/2013/101759.
234. PUTTE, J.A.R.GÜE.L.LO. VAN DE TOXIC, ICHTHYOTOXIC, INSECT-REPELLENT, AND BEE-ATTRACTANT PLANTS USED IN THE SERRA DO AÇOR (PORTUGAL). *https://doi.org/10.2993/0278-0771(2005)25[228:TIIABP]2.0.CO;2* **2005**, *25*, 228–239, doi:10.2993/0278-0771(2005)25.
235. Arifah, F.H.; Nugroho, A.E.; Rohman, A.; Sujarwo, W. A Review of Medicinal Plants for the Treatment of Diabetes Mellitus: The Case of Indonesia. *South African Journal of Botany* **2022**, *149*, 537–558, doi:10.1016/J.SAJB.2022.06.042.
236. Aziz, M.A.; Adnan, M.; Khan, A.H.; Rehman, A.U.; Jan, R.; Khan, J. Ethno-Medicinal Survey of Important Plants Practiced by Indigenous Community at Ladha Subdivision, South Waziristan Agency, Pakistan. *J Ethnobiol Ethnomed* **2016**, *12*, doi:10.1186/S13002-016-0126-7.
237. Balogun, F.; medica, A.A.-P.; 2019, undefined A Review of Plants Used in South African Traditional Medicine for the Management and Treatment of Hypertension. *thieme-connect.comFO Balogun, AOT AshafaPlanta medica, 2019•thieme-connect.com* **2019**, *85*, 312–334, doi:10.1055/a-0801-8771.
238. Namukobe, J.; Kasenene, J.M.; Kiremire, B.T.; Byamukama, R.; Kamatenesi-Mugisha, M.; Krief, S.; Dumontet, V.; Kabasa, J.D. Traditional Plants Used for Medicinal Purposes by Local Communities around the Northern Sector of Kibale National Park, Uganda. *J Ethnopharmacol* **2011**, *136*, 236–245, doi:10.1016/J.JEP.2011.04.044.

239. Jazani, A.M.; Hamdi, K.; Tansaz, M.; Nazemiyeh, H.; Bazargani, H.S.; Bagher Fazljou, S.M.; Doost Azgomi, R.N. Herbal Medicine for Oligomenorrhea and Amenorrhea: A Systematic Review of Ancient and Conventional Medicine. *Biomed Res Int* **2018**, *2018*, doi:10.1155/2018/3052768.
240. Belhouala, K.; Benarba, B. Medicinal Plants Used by Traditional Healers in Algeria: A Multiregional Ethnobotanical Study. *Front Pharmacol* **2021**, *12*, doi:10.3389/FPHAR.2021.760492.
241. Tahraoui, A.; El-Hilaly, J.; Israili, Z.H.; Lyoussi, B. Ethnopharmacological Survey of Plants Used in the Traditional Treatment of Hypertension and Diabetes in South-Eastern Morocco (Errachidia Province). *J Ethnopharmacol* **2007**, *110*, 105–117, doi:10.1016/J.JEP.2006.09.011.
242. Eddouks, M.; Ajebli, M.; Hebi, M. Ethnopharmacological Survey of Medicinal Plants Used in Daraa-Tafilalet Region (Province of Errachidia), Morocco. *J Ethnopharmacol* **2017**, *198*, 516–530, doi:10.1016/J.JEP.2016.12.017.
243. Ziyyat, A.; Legssyer, A.; Mekhfi, H.; Dassouli, A.; Serhrouchni, M.; Benjelloun, W. Phytotherapy of Hypertension and Diabetes in Oriental Morocco. *J Ethnopharmacol* **1997**, *58*, 45–54, doi:10.1016/S0378-8741(97)00077-9.
244. El-Gharbaoui, A.; Benítez, G.; González-Tejero, M.R.; Molero-Mesa, J.; Merzouki, A. Comparison of Lamiaceae Medicinal Uses in Eastern Morocco and Eastern Andalusia and in Ibn Al-Baytar's Compendium of Simple Medicaments (13th Century CE). *J Ethnopharmacol* **2017**, *202*, 208–224, doi:10.1016/J.JEP.2017.03.014.
245. Jouad, H.; Haloui, M.; Rhiouani, H.; El Hilaly, J.; Eddouks, M. Ethnobotanical Survey of Medicinal Plants Used for the Treatment of Diabetes, Cardiac and Renal Diseases in the North Centre Region of Morocco (Fez–Boulemane). *J Ethnopharmacol* **2001**, *77*, 175–182, doi:10.1016/S0378-8741(01)00289-6.
246. Bouyahya, A.; Abrini, J.; Et-Touys, A.; Bakri, Y.; Dakka, N. Indigenous Knowledge of the Use of Medicinal Plants in the North-West of Morocco and Their Biological Activities. *Eur J Integr Med* **2017**, *13*, 9–25, doi:10.1016/J.EUJIM.2017.06.004.
247. Lahlou, M.; Berrada, R.; Agoumi, A.; Hmamouchi, M. The Potential Effectiveness of Essential Oils in the Control of Human Head Lice in Morocco. *International Journal of Aromatherapy* **2001**, *10*, 108–123, doi:10.1016/S0962-4562(01)80005-9.
248. Teixidor-Toneu, I.; Martin, G.J.; Ouhammou, A.; Puri, R.K.; Hawkins, J.A. An Ethnomedicinal Survey of a Tashelhit-Speaking Community in the High Atlas, Morocco. *J Ethnopharmacol* **2016**, *188*, 96–110, doi:10.1016/J.JEP.2016.05.009.
249. González-Tejero, M.R.; Casares-Porcel, M.; Sánchez-Rojas, C.P.; Ramiro-Gutiérrez, J.M.; Molero-Mesa, J.; Pieroni, A.; Giusti, M.E.; Censorii, E.; de Pasquale, C.; Della, A.; et al. Medicinal Plants in the Mediterranean Area: Synthesis of the Results of the Project Rubia. *J Ethnopharmacol* **2008**, *116*, 341–357, doi:10.1016/J.JEP.2007.11.045.
250. Jamila, F.; Mostafa, E. Ethnobotanical Survey of Medicinal Plants Used by People in Oriental Morocco to Manage Various Ailments. *J Ethnopharmacol* **2014**, *154*, 76–87, doi:10.1016/J.JEP.2014.03.016.

251. El-Hilaly, J.; Hmammouchi, M.; Ethnopharmacology, B.L.-J. of; 2003, undefined Ethnobotanical Studies and Economic Evaluation of Medicinal Plants in Taounate Province (Northern Morocco). *Elsevier*.
252. Tuttolomondo, T.; Licata, M.; Leto, C.; Savo, V.; Bonsangue, G.; Letizia Gargano, M.; Venturella, G.; La Bella, S. Ethnobotanical Investigation on Wild Medicinal Plants in the Monti Sicani Regional Park (Sicily, Italy). *J Ethnopharmacol* **2014**, *153*, 568–586, doi:10.1016/J.JEP.2014.02.032.
253. Novais, M.H.; Santos, I.; Mendes, S.; Pinto-Gomes, C. Studies on Pharmaceutical Ethnobotany in Arrabida Natural Park (Portugal). *J Ethnopharmacol* **2004**, *93*, 183–195, doi:10.1016/J.JEP.2004.02.015.
254. Hanlidou, E.; Karousou, R.; Kleftoyanni, V.; Kokkini, S. The Herbal Market of Thessaloniki (N Greece) and Its Relation to the Ethnobotanical Tradition. *J Ethnopharmacol* **2004**, *91*, 281–299, doi:10.1016/J.JEP.2004.01.007.
255. Pardo De Santayana, M.; Blanco, E.; Morales, R. Plants Known as Té in Spain: An Ethno-Pharmaco-Botanical Review. *J Ethnopharmacol* **2005**, *98*, 1–19, doi:10.1016/J.JEP.2004.11.003.
256. Hadj-Said, D.; Bouazza, B. Medicinal Plants Used for the Treatment of Respiratory Diseases in Kabylia, North of Algeria: An Ethnomedicinal Survey. *J Herb Med* **2023**, *40*, 100685, doi:10.1016/J.HERMED.2023.100685.
257. Andrade-Cetto, A.; Heinrich, M. Mexican Plants with Hypoglycaemic Effect Used in the Treatment of Diabetes. *J Ethnopharmacol* **2005**, *99*, 325–348, doi:10.1016/J.JEP.2005.04.019.
258. Karousou, R.; Balta, M.; Hanlidou, E.; Kokkini, S. "Mints", Smells and Traditional Uses in Thessaloniki (Greece) and Other Mediterranean Countries. *J Ethnopharmacol* **2007**, *109*, 248–257, doi:10.1016/J.JEP.2006.07.022.
259. Vázquez, F.M.; Suarez, M.A.; Pérez, A. Medicinal Plants Used in the Barros Area, Badajoz Province (Spain). *J Ethnopharmacol* **1997**, *55*, 81–85, doi:10.1016/S0378-8741(96)01491-2.
260. Oniga, I.; Pus, C.; Silaghi-Dumitrescu, R.; Olah, N.K.; Sevastre, B.; Marica, R.; Marcus, I.; Sevastre-Berghian, A.C.; Benedec, D.; Pop, C.E.; et al. Origanum Vulgare Ssp. Vulgare: Chemical Composition and Biological Studies. *Molecules 2018, Vol. 23, Page 2077* **2018**, *23*, 2077, doi:10.3390/MOLECULES23082077.
261. Lombrea, A.; Antal, D.; Ardelean, F.; Avram, S.; Pavel, I.Z.; Vlaia, L.; Mut, A.M.; Diaconeasa, Z.; Dehelean, C.A.; Soica, C.; et al. A Recent Insight Regarding the Phytochemistry and Bioactivity of Origanum Vulgare L. Essential Oil. *International Journal of Molecular Sciences 2020, Vol. 21, Page 9653* **2020**, *21*, 9653, doi:10.3390/IJMS21249653.
262. Mahomoodally, M.F.; Mootoosamy, A.; Wambugu, S. Traditional Therapies Used to Manage Diabetes and Related Complications in Mauritius: A Comparative Ethnoreligious Study. *Evid Based Complement Alternat Med* **2016**, *2016*, doi:10.1155/2016/4523828.

263. Jiao, X.; Liu, H.; Lu, Q.; Wang, Y.; Zhao, Y.; Liu, X.; Liu, F.; Zuo, Y.; Wang, W.; Li, Y. Study on the Mechanism of Prunella Vulgaris L on Diabetes Mellitus Complicated with Hypertension Based on Network Pharmacology and Molecular Docking Analyses. *J Diabetes Res* **2021**, *2021*, doi:10.1155/2021/9949302.
264. Cheung, H.Y.; Zhang, Q.F. Enhanced Analysis of Triterpenes, Flavonoids and Phenolic Compounds in Prunella Vulgaris L. by Capillary Zone Electrophoresis with the Addition of Running Buffer Modifiers. *J Chromatogr A* **2008**, *1213*, 231–238, doi:10.1016/J.CHROMA.2008.10.033.
265. Adámková, H.; Vicar, J.; Palasová, J.; Ulrichová, J.; Simánek, V. Macleya Cordata and Prunella Vulgaris in Oral Hygiene Products - Their Efficacy in the Control of Gingivitis. *Biomed Pap Med Fac Univ Palacky Olomouc Czech Repub* **2004**, *148*, 103–105, doi:10.5507/BP.2004.019.
266. Morteza-Semnani, K.; … M.S.-J. of E.O.; 2006, undefined The Essential Oil Composition of Prunella Vulgaris L. *Taylor & FrancisK Morteza-Semnani, M Saeedi, M AkbarzadehJournal of Essential Oil Bearing Plants, 2006•Taylor & Francis* **2013**, *9*, 257–260, doi:10.1080/0972060X.2006.10643500.
267. Kojima, H.; Ogura, H. Triterpenoids from Prunella Vulgaris. *Phytochemistry* **1986**, *25*, 729–733, doi:10.1016/0031-9422(86)88033-5.
268. Yeşilada, E.; Honda, G.; Sezik, E.; Tabata, M.; … K.G.-J. of; 1993, undefined Traditional Medicine in Turkey IV. Folk Medicine in the Mediterranean Subdivision. *Elsevier* **1993**, *39*, 31–38, doi:10.1016/0378-8741(93)90048-A.
269. Balan, A.; Moga, M.A.; Dima, L.; Dinu, C.G.; Martinescu, C.C.; Panait, D.E.; Irimie, C.A.; Anastasiu, C.V. An Overview on the Conservative Management of Endometriosis from a Naturopathic Perspective: Phytochemicals and Medicinal Plants. *Plants* **2021**, *10*, 1–31, doi:10.3390/PLANTS10030587.
270. Jin, B.; Liu, Y.; Xie, J.; Luo, B.; Long, C. Ethnobotanical Survey of Plant Species for Herbal Tea in a Yao Autonomous County (Jianghua, China): Results of a 2-Year Study of Traditional Medicinal Markets on the Dragon Boat Festival. *J Ethnobiol Ethnomed* **2018**, *14*, doi:10.1186/S13002-018-0257-0.
271. Shaheen, H.; Qaseem, M.F.; Amjad, M.S.; Bruschi, P. Exploration of Ethno-Medicinal Knowledge among Rural Communities of Pearl Valley; Rawalakot, District Poonch Azad Jammu and Kashmir. *PLoS One* **2017**, *12*, doi:10.1371/JOURNAL.PONE.0183956.
272. Haq, S.M.; Waheed, M.; Khoja, A.A.; Amjad, M.S.; Bussmann, R.W.; Ali, K. A Cross-Cultural Study of High-Altitude Botanical Resources among Diverse Ethnic Groups in Kashmir Himalaya, India. *J Ethnobiol Ethnomed* **2023**, *19*, 12, doi:10.1186/S13002-023-00582-8.

273. Tariq, A.; Adnan, M.; Abdelsalam, N.M.; Fouad, H.; Hussain, K.; Ullah, R.; Ullah, A. Richness and Cover of Nontimber Economic Plants along Altitude in Temperate Himalayan Forest-Use Types. *Scientific World Journal* **2014**, *2014*, doi:10.1155/2014/748490.
274. Khan, S.M.; Page, S.; Ahmad, H.; Shaheen, H.; Ullah, Z.; Ahmad, M.; Harper, D.M. Medicinal Flora and Ethnoecological Knowledge in the Naran Valley, Western Himalaya, Pakistan. *J Ethnobiol Ethnomed* **2013**, *9*, doi:10.1186/1746-4269-9-4.
275. Orch, H.; Douira, A.; Zidane, L. Étude Ethnobotanique Des Plantes Médicinales Utilisées Dans Le Traitement Du Diabète, et Des Maladies Cardiaques Dans La Région d'Izarène (Nord Du Maroc). *J Appl Biosci* **2015**, *86*, 7940, doi:10.4314/JAB.V86I1.3.
276. Taïbi, K.; Ait Abderrahim, L.; Helal, F.; Hadji, K. Ethnopharmacological Study of Herbal Remedies Used for the Management of Thyroid Disorders in Algeria. *Saudi Pharmaceutical Journal* **2021**, *29*, 43–52, doi:10.1016/J.JSPS.2020.12.004.
277. Daoudi, A.; Bammou, M.; Zarkani, S.; Slimani, I.; Ibijbijen, J.; Nassiri, L. Étude Ethnobotanique de La Flore Médicinale Dans La Commune Rurale d'Aguelmouss Province de Khénifra (Maroc). *Phytotherapie* **2016**, *14*, 220–228, doi:10.1007/S10298-015-0953-Z.
278. Taïbi, K.; Abderrahim, L.A.; Ferhat, K.; Betta, S.; Taïbi, F.; Bouraada, F.; Boussaid, M. Ethnopharmacological Study of Natural Products Used for Traditional Cancer Therapy in Algeria. *Saudi Pharmaceutical Journal : SPJ* **2020**, *28*, 1451, doi:10.1016/J.JSPS.2020.09.011.
279. Obón, C.; Rivera, D.; Verde, A.; Fajardo, J.; Valdés, A.; Alcaraz, F.; Carvalho, A.M. Árnica: A Multivariate Analysis of the Botany and Ethnopharmacology of a Medicinal Plant Complex in the Iberian Peninsula and the Balearic Islands. *J Ethnopharmacol* **2012**, *144*, 44–56, doi:10.1016/J.JEP.2012.08.024.
280. González, J.A.; García-Barriuso, M.; Amich, F. Ethnobotanical Study of Medicinal Plants Traditionally Used in the Arribes Del Duero, Western Spain. *J Ethnopharmacol* **2010**, *131*, 343–355, doi:10.1016/J.JEP.2010.07.022.
281. Nortje, J.M.; Van Wyk, B.E. Medicinal Plants of the Kamiesberg, Namaqualand, South Africa. *J Ethnopharmacol* **2015**, *171*, 205–222, doi:10.1016/J.JEP.2015.04.049.
282. Menale, B.; De Castro, O.; Cascone, C.; Muoio, R. Ethnobotanical Investigation on Medicinal Plants in the Vesuvio National Park (Campania, Southern Italy). *J Ethnopharmacol* **2016**, *192*, 320–349, doi:10.1016/J.JEP.2016.07.049.
283. El Abbouyi, P.A.; Filali-Ansari, N.; Khyari, P.S.; Loukili, H. Inventory of Medicinal Plants Prescribed by Traditional Healers in El Jadida City and Suburbs (Morocco). *International Journal of Green Pharmacy* **2014**, *8*, 242–251, doi:10.4103/0973-8258.142681.

284. Salhi, N.; Bouyahya, A.; Fettach, S.; Zellou, A.; Cherrah, Y. Ethnopharmacological Study of Medicinal Plants Used in the Treatment of Skin Burns in Occidental Morocco (Area of Rabat). *South African Journal of Botany* **2019**, *121*, 128–142, doi:10.1016/J.SAJB.2018.10.038.
285. Slimani, I.; Najem, M.; Belaidi, R.; … L.B.-… of I. and; 2016, undefined Étude Ethnobotanique Des Plantes Médicinales Utilisées Dans La Région de Zerhoun-Maroc-[Ethnobotanical Survey of Medicinal Plants Used in Zerhoun Region. *search.proquest.comI Slimani, M Najem, R Belaidi, L Bachiri, EH Bouiamrine, L Nassiri, J IbijbijenInternational Journal of Innovation and Applied Studies, 2016•search.proquest.com*.
286. Barros, L.; Heleno, S.A.; Carvalho, A.M.; Ferreira, I.C.F.R. Lamiaceae Often Used in Portuguese Folk Medicine as a Source of Powerful Antioxidants: Vitamins and Phenolics. *LWT* **2010**, *43*, 544–550, doi:10.1016/J.LWT.2009.09.024.
287. Rodrigues, M.; Lopes, A.C.; Vaz, F.; Filipe, M.; Alves, G.; Ribeiro, M.P.; Coutinho, P.; Araujo, A.R.T.S. Thymus Mastichina: Composition and Biological Properties with a Focus on Antimicrobial Activity. *Pharmaceuticals 2020, Vol. 13, Page 479* **2020**, *13*, 479, doi:10.3390/PH13120479.
288. Benítez, G.; Molero-Mesa, J.; González-Tejero, M.R. Wild Edible Plants of Andalusia: Traditional Uses and Potential of Eating Wild in a Highly Diverse Region. *Plants* **2023**, *12*, doi:10.3390/PLANTS12061218/S1.
289. Benítez, G.; González-Tejero, M.R.; Molero-Mesa, J. Pharmaceutical Ethnobotany in the Western Part of Granada Province (Southern Spain): Ethnopharmacological Synthesis. *J Ethnopharmacol* **2010**, *129*, 87–105, doi:10.1016/J.JEP.2010.02.016.
290. LEWIS, J.J. DIABETES AND THE INSULIN-ADMINISTRATION PROBLEM. *https://doi.org/10.1152/physrev.1949.29.1.75* **1949**, *29*, 75–90, doi:10.1152/PHYSREV.1949.29.1.75.
291. Gray, A.M.; Flatt, P.R. Actions of the Traditional Anti-Diabetic Plant, Agrimony Eupatoria (Agrimony): Effects on Hyperglycaemia, Cellular Glucose Metabolism and Insulin Secretion. *Br J Nutr* **1998**, *80*, 109–114, doi:10.1017/S0007114598001834.
292. PALUCH, Z.; BIRICZOVÁ, L.; PALLAG, G.; MARQUES, E.C.; VARGOVÁ, N.; KMONÍČKOVÁ, E. The Therapeutic Effects of Agrimonia Eupatoria L. *Physiol Res* **2020**, *69*, S555, doi:10.33549/PHYSIOLRES.934641.
293. Dalar, A. Plant Taxa Used in the Treatment of Diabetes in Van Province, Turkey. *International Journal of Secondary Metabolite* **2018**, *5*, 171–185, doi:10.21448/ijsm.430703.
294. Nabavi, S.F.; Habtemariam, S.; Ahmed, T.; Sureda, A.; Daglia, M.; Sobarzo-Sánchez, E.; Nabavi, S.M. Polyphenolic Composition of Crataegus Monogyna Jacq.: From Chemistry to Medical Applications. *Nutrients 2015, Vol. 7, Pages 7708-7728* **2015**, *7*, 7708–7728, doi:10.3390/NU7095361.

295. Madić, V.; Stojanović-Radić, Z.; Jušković, M.; Jugović, D.; Žabar Popović, A.; Vasiljević, P. Genotoxic and Antigenotoxic Potential of Herbal Mixture and Five Medicinal Plants Used in Ethnopharmacology. *South African Journal of Botany* **2019**, *125*, 290–297, doi:10.1016/J.SAJB.2019.07.043.
296. Kuenzle, J. Herbs and Weeds. **1996**.
297. Al-Snafi, A.E. Constituents and Pharmacology of Geum Urbanum-A Review. **2019**, *9*, 28–33.
298. Tomczyk, M.; Latté, K.P. Potentilla—A Review of Its Phytochemical and Pharmacological Profile. *J Ethnopharmacol* **2009**, *122*, 184–204, doi:10.1016/J.JEP.2008.12.022.
299. Ivancheva, S.; Stantcheva, B. Ethnobotanical Inventory of Medicinal Plants in Bulgaria. *J Ethnopharmacol* **2000**, *69*, 165–172, doi:10.1016/S0378-8741(99)00129-4.
300. Lachin, T.; Reza, H. Anti Diabetic Effect of Cherries in Alloxan Induced Diabetic Rats. *Recent Pat Endocr Metab Immune Drug Discov* **2012**, *6*, 67–72, doi:10.2174/187221412799015308.
301. Sargin, S.A.; Akçicek, E.; Selvi, S. An Ethnobotanical Study of Medicinal Plants Used by the Local People of Alaşehir (Manisa) in Turkey. *J Ethnopharmacol* **2013**, *150*, 860–874, doi:10.1016/J.JEP.2013.09.040.
302. Vinagre, C.; Vinagre, S.; Carrilho, E. The Use of Medicinal Plants by the Population from the Protected Landscape of "Serra de Montejunto", Portugal. *J Ethnobiol Ethnomed* **2019**, *15*, 1–30, doi:10.1186/S13002-019-0309-0/TABLES/6.
303. Idolo, M.; Motti, R.; Mazzoleni, S. Ethnobotanical and Phytomedicinal Knowledge in a Long-History Protected Area, the Abruzzo, Lazio and Molise National Park (Italian Apennines). *J Ethnopharmacol* **2010**, *127*, 379–395, doi:10.1016/J.JEP.2009.10.027.
304. Novais, M.H.; Santos, I.; Mendes, S.; Pinto-Gomes, C. Studies on Pharmaceutical Ethnobotany in Arrabida Natural Park (Portugal). *J Ethnopharmacol* **2004**, *93*, 183–195, doi:10.1016/J.JEP.2004.02.015.
305. Sargin, S.A.; Selvi, S.; López, V. Ethnomedicinal Plants of Sarigöl District (Manisa), Turkey. *J Ethnopharmacol* **2015**, *171*, 64–84, doi:10.1016/J.JEP.2015.05.031.
306. Petrakou, K.; Iatrou, G.; Lamari, F.N. Ethnopharmacological Survey of Medicinal Plants Traded in Herbal Markets in the Peloponnisos, Greece. *J Herb Med* **2020**, *19*, 100305, doi:10.1016/J.HERMED.2019.100305.
307. Yesilada, E.; Sezik, E. Traditional Medicine in Turkey IV. Folk Medicine in the Mediterranean Subdivision Article. *J Ethnopharmacol* **1993**, doi:10.1016/0378-8741(93)90048-A.
308. Egea, T.; Signorini, M.A.; Bruschi, P.; Rivera, D.; Obón, C.; Alcaraz, F.; Palazón, J.A. Spirits and Liqueurs in European Traditional Medicine: Their History and Ethnobotany in Tuscany and Bologna (Italy). *J Ethnopharmacol* **2015**, *175*, 241–255, doi:10.1016/J.JEP.2015.08.053.

309. Benítez, G.; González-Tejero, M.R.; Molero-Mesa, J. Pharmaceutical Ethnobotany in the Western Part of Granada Province (Southern Spain): Ethnopharmacological Synthesis. *J Ethnopharmacol* **2010**, *129*, 87–105, doi:10.1016/J.JEP.2010.02.016.
310. Tsioutsiou, E.E.; Cheilari, A.; Aligiannis, N. Ethnopharmacological Study of Medicinal Plants Used against Skin Ailments on Mount Pelion, Central Greece. *Front Pharmacol* **2023**, *14*, 1225580, doi:10.3389/FPHAR.2023.1225580/BIBTEX.
311. Güler, B.; Manav, E.; Uɫurlu, E. Medicinal Plants Used by Traditional Healers in Bozüyük (Bilecik–Turkey). *J Ethnopharmacol* **2015**, *173*, 39–47, doi:10.1016/J.JEP.2015.07.007.
312. Antunes, J.; Ribeiro, M.M. O Azereiro (Prunus Lusitanica L.): Uma Monografia. *Agroforum* **2007**, *18*, 33–36.
313. Chrubasik, C.; Roufogalis, B.D.; Müller-Ladner, U.; Chrubasik, S. ROSA CANINA EFFECT AND EFFICACY PROFILES 725 A Systematic Review on the Rosa Canina Effect and Efficacy Profiles. *Phytother. Res* **2008**, *22*, 725–733, doi:10.1002/ptr.
314. Kültür, Ş. Medicinal Plants Used in Kırklareli Province (Turkey). *J Ethnopharmacol* **2007**, *111*, 341–364, doi:10.1016/J.JEP.2006.11.035.
315. Khazaei, M.; Pazhouhi, M. An Overview of Therapeutic Potentials of Rosa Canina: A Traditionally Valuable Herb. *wcrj.net*.
316. Ahmad, N.; Anwar, F.; Gilani, A. ul H. Rose Hip (Rosa Canina L.) Oils. *Essential Oils in Food Preservation, Flavor and Safety* **2016**, 667–675, doi:10.1016/B978-0-12-416641-7.00076-6.
317. Živković, J.; Stojković, D.; Petrović, J.; Zdunić, G.; Glamočlija, J.; Soković, M. Rosa Canina L. – New Possibilities for an Old Medicinal Herb. *Food Funct* **2015**, *6*, 3687–3692, doi:10.1039/C5FO00820D.
318. Ethnobotany of the Caucasus. **2017**, doi:10.1007/978-3-319-49412-8.
319. Kolosova, V.; Pashkova, T.; Muslimov, M.; Sõukand, R. Historical Review of Ethnopharmacology in Karelia (1850s–2020s): Herbs and Healers. *J Ethnopharmacol* **2022**, *282*, 114565, doi:10.1016/J.JEP.2021.114565.
320. Leporatti, M.L.; Ivancheva, S. Preliminary Comparative Analysis of Medicinal Plants Used in the Traditional Medicine of Bulgaria and Italy. *J Ethnopharmacol* **2003**, *87*, 123–142, doi:10.1016/S0378-8741(03)00047-3.
321. Kültür, Ş. Medicinal Plants Used in Kırklareli Province (Turkey). *J Ethnopharmacol* **2007**, *111*, 341–364, doi:10.1016/J.JEP.2006.11.035.
322. Sak, K.; Jürisoo, K.; Raal, A. Estonian Folk Traditional Experiences on Natural Anticancer Remedies: From Past to the Future. *Pharm Biol* **2014**, *52*, 855–866, doi:10.3109/13880209.2013.871641.
323. Asadi-Samani, M.; Moradi, M.T.; Mahmoodnia, L.; Alaei, S.; Asadi-Samani, F.; Luther, T. Traditional Uses of Medicinal Plants to Prevent and Treat Diabetes; an Updated Review of Ethnobotanical Studies in Iran. *J Nephropathol* **2017**, *6*, 118, doi:10.15171/JNP.2017.20.

324. Amiri, M.S.; Joharchi, M.R. Ethnobotanical Investigation of Traditional Medicinal Plants Commercialized in the Markets of Mashhad, Iran. *Avicenna J Phytomed* **2013**, *3*, 254.
325. Jamsid Ibadullayeva, S. Ethnopharmacological Use of Wild Vegetable Plants Belonging to the Polygonaceae Juss. Family Spread in the Azerbaijan Flora. **2022**, doi:10.31396/Biodiv.Jour.2021.12.3.733.740.
326. Matejić, J.S.; Stefanović, N.; Ivković, M.; Živanović, N.; Marin, P.D.; Džamić, A.M. Traditional Uses of Autochthonous Medicinal and Ritual Plants and Other Remedies for Health in Eastern and South-Eastern Serbia. *J Ethnopharmacol* **2020**, *261*, 113186, doi:10.1016/J.JEP.2020.113186.
327. Choudhary, R.; Oh, S.; Plants, J.L.-J. of M.; 2011, undefined An Ethnomedicinal Inventory of Knotweeds of Indian Himalaya. *academia.eduRK Choudhary, S Oh, J LeeJournal of Medicinal Plants Research, 2011•academia.edu*.
328. Uddin, M.Z.; Rashid, M.M.; Hamid, S.A.; Nahar, L.; Begum, M.I.A.; Khan, A.; Rahman, M. Prevalence and Identification of Ticks in Cattle Of Rajshahi, Bangladesh. *Research in Agriculture Livestock and Fisheries* **2022**, *9*, 171–183, doi:10.3329/RALF.V9I2.61623.
329. Mollik, M.A.H.; Hossan, M.S.H.; Paul, A.K.; Taufiq-Ur-Rahman, M.; Jahan, R.; Rahmatullah, M. A Comparative Analysis of Medicinal Plants Used by Folk Medicinal Healers in Three Districts of Bangladesh and Inquiry as to Mode of Selection of Medicinal Plants. *Ethnobotany Research and Applications* **2010**, *8*, 195–218, doi:10.17348/ERA.8.0.195-218.
330. Karaman, Ö.; Cebe, G.E. Diabetes and Antidiabetic Plants Used in Turkey. *Ankara Universitesi Eczacilik Fakultesi Dergisi* 2016, *40*, 47–61.
331. Vasas, A.; Orbán-Gyapai, O.; Hohmann, J. The Genus Rumex: Review of Traditional Uses, Phytochemistry and Pharmacology. *J Ethnopharmacol* **2015**, *175*, 198–228, doi:10.1016/J.JEP.2015.09.001.
332. Bello, O.M.; Fasinu, P.S.; Bello, O.E.; Ogbesejana, A.B.; Adetunji, C.O.; Dada, A.O.; Ibitoye, O.S.; Aloko, S.; Oguntoye, O.S. Wild Vegetable Rumex Acetosa Linn.: Its Ethnobotany, Pharmacology and Phytochemistry – A Review. *South African Journal of Botany* **2019**, *125*, 149–160, doi:10.1016/J.SAJB.2019.04.018.
333. Korpelainen, H.; Pietiläinen, M. Sorrel (Rumex Acetosa L.): Not Only a Weed but a Promising Vegetable and Medicinal Plant. *Botanical Review* **2020**, *86*, 234–246, doi:10.1007/S12229-020-09225-Z/TABLES/2.
334. Alzweiri, M.; Sarhan, A. Al; Mansi, K.; Hudaib, M.; Aburjai, T. Ethnopharmacological Survey of Medicinal Herbs in Jordan, the Northern Badia Region. *J Ethnopharmacol* **2011**, *137*, 27–35, doi:10.1016/J.JEP.2011.02.007.
335. SARI, F.; KOÇYİĞİT, M. Ethnobotanical Usages of the Turkish Rumex Taxa. *Turkish Journal of Bioscience and Collections* **2021**, *5*, 123–140, doi:10.26650/tjbc.2021930272.
336. Bioactive Natural Products for Pharmaceutical Applications. **2021**, *140*, doi:10.1007/978-3-030-54027-2.

337. Guarrera, P.M.; Savo, V. Perceived Health Properties of Wild and Cultivated Food Plants in Local and Popular Traditions of Italy: A Review. *J Ethnopharmacol* **2013**, *146*, 659–680, doi:10.1016/J.JEP.2013.01.036.
338. Bulut, G.; Haznedaroğlu, M.Z.; Doğan, A.; Koyu, H.; Tuzlacı, E. An Ethnobotanical Study of Medicinal Plants in Acipayam (Denizli-Turkey). *J Herb Med* **2017**, *10*, 64–81, doi:10.1016/J.HERMED.2017.08.001.
339. Megharbi, A.; Kechairi, R. Ethnobotanical Characterization of Halophytes with Medicinal Virtues, Case of the Macta Wetland Flora: North-West Algeria. *Genetics & Biodiversity Journal* **2021**, *5*, 135–145, doi:10.46325/GABJ.V5I2.82.
340. Medicinales Aromáticas, P. Boletín Latinoamericano y Del Caribe De. *Plantas Medicinales y Aromáticas* **2012**, *11*.
341. Mrabti, H.N.; Doudach, L.; Mekkaoui, M.; Khalil, Z.; Harraqui, K.; Fozia, F.; Naceiri Mrabti, N.; El-Shazly, M.; Alotaibi, A.; Ullah, R.; et al. Profile of Medicinal Plants Traditionally Used for the Treatment of Skin Burns. *Evidence-based Complementary and Alternative Medicine* **2022**, *2022*, doi:10.1155/2022/3436665.
342. Aboukhalaf, A.; Tbatou, M.; Kalili, A.; Naciri, K.; Moujabbir, S.; Sahel, K.; Rocha, J.M.; Correspondence, R.B.; Aboukhalaf[1], A.; Tbatou[1], M.; et al. Traditional Knowledge and Use of Wild Edible Plants in Sidi Bennour Region (Central Morocco). *Ethnobotany Research and Applications* **2022**, *23*, 11, doi:10.32859/era.23.11.1-18.
343. Sargin, S.A.; Akçicek, E.; Selvi, S. An Ethnobotanical Study of Medicinal Plants Used by the Local People of Alaşehir (Manisa) in Turkey. *J Ethnopharmacol* **2013**, *150*, 860–874, doi:10.1016/J.JEP.2013.09.040.
344. Bencheikh, N.; Elbouzidi, A.; Kharchoufa, L.; Ouassou, H.; Merrouni, I.A.; Mechchate, H.; Es-Safi, I.; Hano, C.; Addi, M.; Bouhrim, M.; et al. Inventory of Medicinal Plants Used Traditionally to Manage Kidney Diseases in North-Eastern Morocco: Ethnobotanical Fieldwork and Pharmacological Evidence. *Plants 2021, Vol. 10, Page 1966* **2021**, *10*, 1966, doi:10.3390/PLANTS10091966.
345. Sarikaya, S.; Öner, H.; Şebnem HARPUT, Ü.; Üniversitesi, H.; Fakültesi, E.; Anabilim Dalı, F.; Eczanesi, H.; Özet, T. Medicinal Plants Used for the Treatment of Diabetes in Turkey. *dergipark.org.tr* **2010**, *39*, 317–342.
346. Ahmad, S.; Botany, S.J.-P.J. of; 2007, undefined Exploring the Economic Value of Underutilized Plant Species in Ayubia National Park. *pakbs.orgSS Ahmad, S JavedPakistan Journal of Botany, 2007•pakbs.org* **2007**, *39*, 1435–1442.
347. Orhan, N.; Deliorman Orhan, D.; Aslan, M.; Ükürolu, M.; Orhan, I.E. UPLC-TOF-MS Analysis of Galium Spurium towards Its Neuroprotective and Anticonvulsant Activities. *J Ethnopharmacol* **2012**, *141*, 220–227, doi:10.1016/J.JEP.2012.01.056.
348. Bokhari, J.; Khan, M.R.; Shabbir, M.; Rashid, U.; Jan, S.; Zai, J.A. Evaluation of Diverse Antioxidant Activities of Galium Aparine. *Spectrochim Acta A Mol Biomol Spectrosc* **2013**, *102*, 24–29, doi:10.1016/J.SAA.2012.09.056.

349. Atmaca, H.; Bozkurt, E.; Cittan, M.; Dilek Tepe, H. Effects of Galium Aparine Extract on the Cell Viability, Cell Cycle and Cell Death in Breast Cancer Cell Lines. *J Ethnopharmacol* **2016**, *186*, 305–310, doi:10.1016/J.JEP.2016.04.007.
350. Shi, G.; Liu, J.; Zhao, W.; Liu, Y.; Tian, X. Separation and Purification and in Vitro Anti-Proliferative Activity of Leukemia Cell K562 of Galium Aparine L. Petroleum Ether Phase. *Saudi Pharmaceutical Journal* **2016**, *24*, 241–244, doi:10.1016/J.JSPS.2016.04.005.
351. Tobyn, G.; Denham, A.; Whitelegg, M. The Western Herbal Tradition: 2000 Years of Medicinal Plant Knowledge. **2016**.
352. Bnouham, M.; Merhfour, F.Z.; Ziyyat, A.; Aziz, M.; Legssyer, A.; Mekhfi, H. Antidiabetic Effect of Some Medicinal Plants of Oriental Morocco in Neonatal Non-Insulin-Dependent Diabetes Mellitus Rats. *Hum Exp Toxicol* **2010**, *29*, 865–871, doi:10.1177/0960327110362704.
353. Merzouki, A.; Ed-derfoufi, F.; Molero Mesa, J. Contribution to the Knowledge of Rifian Traditional Medicine. II: Folk Medicine in Ksar Lakbir District (NW Morocco). *Fitoterapia* **2000**, *71*, 278–307, doi:10.1016/S0367-326X(00)00139-8.
354. El, A.; El Youbi, H.; El, L.; Sidi, M.; Ben, M.; Boukhira, S.; Ben, S.M.; Youbi, H. El; Mansouri, L. El; Daoudi, A.; et al. In Vivo Anti-Inflammatory and Analgesic Effects of Aqueous Extract of Cistus Ladanifer L. from Morocco. *journals.lww.comAEH El Youbi, L El Mansouri, S Boukhira, A Daoudi, D BoustaAmerican Journal of Therapeutics, 2016•journals.lww.com* **2016**, *23*, e1554–e1559, doi:10.1097/MJT.0000000000000419.
355. Barkaoui, M.; Katiri, A.; Boubaker, H.; Msanda, F. Ethnobotanical Survey of Medicinal Plants Used in the Traditional Treatment of Diabetes in Chtouka Ait Baha and Tiznit (Western Anti-Atlas), Morocco. *J Ethnopharmacol* **2017**, *198*, 338–350, doi:10.1016/J.JEP.2017.01.023.
356. Tuttolomondo, T.; Licata, M.; Leto, C.; … G.B.-J. of; 2014, undefined Popular Uses of Wild Plant Species for Medicinal Purposes in the Nebrodi Regional Park (North-Eastern Sicily, Italy). *Elsevier*.
357. Emre, G.; Dogan, A.; Haznedaroglu, M.Z.; Senkardes, I.; Ulger, M.; Satiroglu, A.; Can Emmez, B.; Tugay, O. An Ethnobotanical Study of Medicinal Plants in Mersin (Turkey). *Front Pharmacol* **2021**, *12*, doi:10.3389/FPHAR.2021.664500.
358. Alarcón, R.; Pardo-De-Santayana, M.; Priestley, C.; Morales, R.; Heinrich, M. Medicinal and Local Food Plants in the South of Alava (Basque Country, Spain). *J Ethnopharmacol* **2015**, *176*, 207–224, doi:10.1016/J.JEP.2015.10.022.
359. Ouhaddou, H.; Boubaker, H.; Msanda, F.; El Mousadik, A. An Ethnobotanical Study of Medicinal Plants of the Agadir Ida Ou Tanane Province (Southwest Morocco). *J Appl Biosci* **2015**, *84*, 7707, doi:10.4314/JAB.V84I1.5.
360. Ergül, A.; Correspondence, B.; Bozkurt, A.E. Folk Medicinal Plants Used for Treatment of Gynecological Disorders by Rural Population of Zorlu Village (in Turkey). *Ethnobotany Research and Applications* **2021**, *22*, 1–17, doi:10.32859/era.22.04.1-17.

361. Kim, H.; Song, M.J.; Potter, D. Medicinal Efficacy of Plants Utilized as Temple Food in Traditional Korean Buddhism. *J Ethnopharmacol* **2006**, *104*, 32–46, doi:10.1016/J.JEP.2005.08.041.
362. Lee, K.T.; Choi, J.H.; Kim, D.H.; Son, K.H.; Kim, W.B.; Kwon, S.H.; Park, H.J. Constituents and the Antitumor Principle of Allium Victorialis Var. Platyphyllum. *Arch Pharm Res* **2001**, *24*, 44–50, doi:10.1007/BF02976492.
363. Najeebullah, S.; Shinwari, Z.K.; Ahmad Jan, S.; Khan, I.; Ali, M. ETHNO-MEDICINAL AND PHYTOCHEMICAL PROPERTIES OF GENUS ALLIUM: A REVIEW OF RECENT ADVANCES. *Pak. J. Bot* **2021**, *53*, 135–144, doi:10.30848/PJB2021-1(34).
364. Shahid, F.; Shahid, R.; Waseem, T.; Hussain, S. Flora of Pakistan: An Ethnopharmacological Perspective. *Journal of Shifa Tameer-e-Millat University* **2020**, *3*, 42–48, doi:10.32593/JSTMU/VOL3.ISS1.79.
365. Kayani, S.; Ahmad, M.; Zafar, M.; Sultana, S.; Khan, M.P.Z.; Ashraf, M.A.; Hussain, J.; Yaseen, G. Ethnobotanical Uses of Medicinal Plants for Respiratory Disorders among the Inhabitants of Gallies – Abbottabad, Northern Pakistan. *J Ethnopharmacol* **2014**, *156*, 47–60, doi:10.1016/J.JEP.2014.08.005.
366. Alachkar, A.; Jaddouh, A.; Elsheikh, M.S.; Bilia, A.R.; Vincieri, F.F. Traditional Medicine in Syria: Folk Medicine in Aleppo Governorate. *Nat Prod Commun* **2011**, *6*, 79–84, doi:10.1177/1934578X1100600119.
367. Muratović, C.E.; Parić, A. Plant Ethnomedicine in Bosnia and Herzegovina, Past and Present. *Ethnobotany Research and Applications* **2023**, *26*, 1–27, doi:10.32859/era.26.61.1-27.
368. Pieroni, A.; Quave, C.L. Traditional Pharmacopoeias and Medicines among Albanians and Italians in Southern Italy: A Comparison. *J Ethnopharmacol* **2005**, *101*, 258–270, doi:10.1016/J.JEP.2005.04.028.
369. Pieroni, A.; Nebel, S.; Quave, C.; Münz, H.; Heinrich, M. Ethnopharmacology of Liakra: Traditional Weedy Vegetables of the Arbëreshë of the Vulture Area in Southern Italy. *J Ethnopharmacol* **2002**, *81*, 165–185, doi:10.1016/S0378-8741(02)00052-1.
370. Savo, V.; Caneva, G.; Maria, G.P.; David, R. Folk Phytotherapy of the Amalfi Coast (Campania, Southern Italy). *J Ethnopharmacol* **2011**, *135*, 376–392, doi:10.1016/J.JEP.2011.03.027.
371. Merrouni, I.A.; Kharchoufa, L.; Bencheikh, N.; Elachouri, M. Ethnobotanical Profile of Medicinal Plants Used by People of North-Eastern Morocco: Cross-Cultural and Historical Approach (Part I). *Ethnobotany Research and Applications* **2021**, *21*, 1–45.
372. Leonti, M.; Casu, L. Ethnopharmacology of Love. *Front Pharmacol* **2018**, *9*, 364413, doi:10.3389/FPHAR.2018.00567/BIBTEX.
373. Li, W.L.; Zheng, H.C.; Bukuru, J.; De Kimpe, N. Natural Medicines Used in the Traditional Chinese Medical System for Therapy of Diabetes Mellitus. *J Ethnopharmacol* **2004**, *92*, 1–21, doi:10.1016/J.JEP.2003.12.031.

374. Zhao, P.; Zhao, C.; Li, X.; Gao, Q.; Huang, L.; Xiao, P.; Gao, W. The Genus Polygonatum: A Review of Ethnopharmacology, Phytochemistry and Pharmacology. *J Ethnopharmacol* **2018**, *214*, 274–291, doi:10.1016/J.JEP.2017.12.006.
375. Bi, J.; Fang, H.; Zhang, J.; Lu, L.; Gu, X.; Zheng, Y. A Review on the Application, Phytochemistry and Pharmacology of Polygonatum Odoratum, an Edible Medicinal Plant. *Journal of Future Foods* **2023**, *3*, 240–251, doi:10.1016/J.JFUTFO.2023.02.006.
376. Wujisguleng, W.; Liu, Y.; Long, C. Ethnobotanical Review of Food Uses of Polygonatum (Convallariaceae) in China. *Acta Societatis Botanicorum Poloniae* **2012**, *81*, 239–244, doi:10.5586/asbp.2012.045.
377. Quave, C.L.; Pieroni, A. Traditional Health Care and | Food and Medicinal Plant Use among Historic Albanian Migrants and Italians in Lucania, Southern Italy.
378. Naceiri Mrabti, H.; Bouyahya, A.; Naceiri Mrabti, N.; Jaradat, N.; Doudach, L.; Faouzi, M.E.A. Ethnobotanical Survey of Medicinal Plants Used by Traditional Healers to Treat Diabetes in the Taza Region of Morocco. *Evidence-based Complementary and Alternative Medicine* **2021**, *2021*, doi:10.1155/2021/5515634.
379. El-Seedi, H.R.; Burman, R.; Mansour, A.; Turki, Z.; Boulos, L.; Gullbo, J.; Göransson, U. The Traditional Medical Uses and Cytotoxic Activities of Sixty-One Egyptian Plants: Discovery of an Active Cardiac Glycoside from Urginea Maritima. *J Ethnopharmacol* **2013**, *145*, 746–757, doi:10.1016/J.JEP.2012.12.007.
380. Sayed, M.D. Traditional Medicine in Health Care. *J Ethnopharmacol* **1980**, *2*, 19–22, doi:10.1016/0378-8741(80)90023-9.
381. Al-Qura'n, S. Ethnopharmacological Survey of Wild Medicinal Plants in Showbak, Jordan. *J Ethnopharmacol* **2009**, *123*, 45–50, doi:10.1016/J.JEP.2009.02.031.
382. Sabah Chermat; Rachid Gharzouli Ethnobotanical Study of Medicinal Flora in the North East of Algeria - An Empirical Knowledge in Djebel Zdimm (Setif). *J Mater Sci Eng A* **2015**, *5*, doi:10.17265/2161-6213/2015.1-2.007.
383. Zatout, F.; Benarba, B.; Bouazza, A.; Babali, B.; Bey, N.N.; Morsli, A. Ethnobotanical Investigation on Medicinal Plants Used by Local Populations in Tlemcen National Park (Extreme North West Algeria). *Mediterranean Botany* **2021**, *42*, e69396–e69396, doi:10.5209/MBOT.69396.
384. Benítez, G.; Molero-Mesa, J.; Reyes González-Tejero, M. Wild Edible Plants of Andalusia: Traditional Uses and Potential of Eating Wild in a Highly Diverse Region. **2023**, doi:10.3390/plants12061218.
385. Fortini, P.; Di Marzio, P.; Guarrera, P.M.; Iorizzi, M. Ethnobotanical Study on the Medicinal Plants in the Mainarde Mountains (Central-Southern Apennine, Italy). *J Ethnopharmacol* **2016**, *184*, 208–218, doi:10.1016/J.JEP.2016.03.010.
386. Uzun, E.; Sariyar, G.; Adsersen, A.; Karakoc, B.; Ötük, G.; Oktayoglu, E.; Pirildar, S. Traditional Medicine in Sakarya Province (Turkey) and Antimicrobial Activities of Selected Species. *J Ethnopharmacol* **2004**, *95*, 287–296, doi:10.1016/J.JEP.2004.07.013.

387. Nath, E.O.; Kultur, S. An Ethnobotanical Study of Medicinal Plants in Savaştepe (Balıkesir-Turkey). *Clin Exp Health Sci* **2022**, *12*, 954–980, doi:10.33808/clinexphealthsci.1026438.
388. González-Tejero, M.R.; Casares-Porcel, M.; Sánchez-Rojas, C.P.; Ramiro-Gutiérrez, J.M.; Molero-Mesa, J.; Pieroni, A.; Giusti, M.E.; Censorii, E.; de Pasquale, C.; Della, A.; et al. Medicinal Plants in the Mediterranean Area: Synthesis of the Results of the Project Rubia. *J Ethnopharmacol* **2008**, *116*, 341–357, doi:10.1016/J.JEP.2007.11.045.
389. Rezouki, S.; Allali, A.; Fadli, M.; Eloutassi, N.; Dahmani, J. A Contribution to the Study of Ruderal Flora of the Fez City! *J. Appl. Sci. Envir. Stud* **2018**, *1*, 8–16.
390. Orch, H.; Zidane, L.; Douira, A. Ethnobotanical Study of Plants Used in the Treatment of Respiratory Diseases in a Population Bordering the Forest of Izarène. *J Pharm Pharmacogn Res* **2020**, *8*, 392–409.
391. Gürdal, B.; Kültür, Ş. An Ethnobotanical Study of Medicinal Plants in Marmaris (Muğla, Turkey). *J Ethnopharmacol* **2013**, *146*, 113–126, doi:10.1016/J.JEP.2012.12.012.
392. Gras, A.; Parada, M.; Garnatje, T.; VallÃ¨s, J. Ethnobotany and Plants Used Against Cardiovascular Diseases in the Iberian Peninsula and the Balearic Islands. **2019**, 1–75, doi:10.2174/9789811403743119020003.
393. Ziyyat, A.; Legssyer, A.; Mekhfi, H.; Dassouli, A.; Serhrouchni, M.; Benjelloun, W. Phytotherapy of Hypertension and Diabetes in Oriental Morocco. *J Ethnopharmacol* **1997**, *58*, 45–54, doi:10.1016/S0378-8741(97)00077-9.
394. Mrabti, H.N.; Jaradat, N.; Kachmar, M.R.; Ed-Dra, A.; Ouahbi, A.; Cherrah, Y.; El Abbes Faouzi, M. Integrative Herbal Treatments of Diabetes in Beni Mellal Region of Morocco. *J Integr Med* **2019**, *17*, 93–99, doi:10.1016/J.JOIM.2019.01.001.
395. Orch, H.; Douira, A.; Zidane, L. Étude Ethnobotanique Des Plantes Médicinales Utilisées Dans Le Traitement Du Diabète, et Des Maladies Cardiaques Dans La Région d'Izarène (Nord Du Maroc). *J Appl Biosci* **2015**, *86*, 7940, doi:10.4314/JAB.V86I1.3.
396. Jouad, H.; Haloui, M.; Rhiouani, H.; El Hilaly, J.; Eddouks, M. Ethnobotanical Survey of Medicinal Plants Used for the Treatment of Diabetes, Cardiac and Renal Diseases in the North Centre Region of Morocco (Fez-Boulemane). *J Ethnopharmacol* **2001**, *77*, 175–182, doi:10.1016/S0378-8741(01)00289-6.
397. Hamel, T.; Zaafour, M.; Boumendjel, M. Ethnomedical Knowledge and Traditional Uses of Aromatic and Medicinal of the Wetlands Complex of the Guerbes-Sanhadja Plain (Wilaya of Skikda in Northeastern Algeria). *Herbal Medicine: Open Access* **2018**, *4*, 3, doi:10.21767/2472-0151.100035.
398. Gras, A.; Parada, M.; Rigat, M.; Vallès, J.; Garnatje, T. Folk Medicinal Plant Mixtures: Establishing a Protocol for Further Studies. *J Ethnopharmacol* **2018**, *214*, 244–273, doi:10.1016/J.JEP.2017.12.014.

399. Bonet, M.À.; Parada, M.; Selga, A.; Vallès, J. Studies on Pharmaceutical Ethnobotany in the Regions of L'Alt Empordà and Les Guilleries (Catalonia, Iberian Peninsula). *J Ethnopharmacol* **1999**, *68*, 145–168, doi:10.1016/S0378-8741(99)00083-5.
400. Menković, N.; Šavikin, K.; Tasić, S.; Zdunić, G.; Stešević, D.; Milosavljević, S.; Vincek, D. Ethnobotanical Study on Traditional Uses of Wild Medicinal Plants in Prokletije Mountains (Montenegro). *J Ethnopharmacol* **2011**, *133*, 97–107, doi:10.1016/J.JEP.2010.09.008.
401. Polat, R.; Cakilcioglu, U.; Kaltalioılu, K.; Ulusan, M.D.; Türkmen, Z. An Ethnobotanical Study on Medicinal Plants in Espiye and Its Surrounding (Giresun-Turkey). *J Ethnopharmacol* **2015**, *163*, 1–11, doi:10.1016/J.JEP.2015.01.008.
402. Jarić, S.; Popović, Z.; Mačukanović-Jocić, M.; Djurdjević, L.; Mijatović, M.; Karadžić, B.; Mitrović, M.; Pavlović, P. An Ethnobotanical Study on the Usage of Wild Medicinal Herbs from Kopaonik Mountain (Central Serbia). *J Ethnopharmacol* **2007**, *111*, 160–175, doi:10.1016/J.JEP.2006.11.007.
403. Abbet, C.; Mayor, R.; Roguet, D.; Spichiger, R.; Hamburger, M.; Potterat, O. Ethnobotanical Survey on Wild Alpine Food Plants in Lower and Central Valais (Switzerland). *J Ethnopharmacol* **2014**, *151*, 624–634, doi:10.1016/J.JEP.2013.11.022.
404. Vogl, S.; Picker, P.; Mihaly-Bison, J.; Fakhrudin, N.; Atanasov, A.G.; Heiss, E.H.; Wawrosch, C.; Reznicek, G.; Dirsch, V.M.; Saukel, J.; et al. Ethnopharmacological in Vitro Studies on Austria's Folk Medicine—An Unexplored Lore in Vitro Anti-Inflammatory Activities of 71 Austrian Traditional Herbal Drugs. *J Ethnopharmacol* **2013**, *149*, 750–771, doi:10.1016/J.JEP.2013.06.007.
405. Vitalini, S.; Iriti, M.; Puricelli, C.; Ciuchi, D.; Segale, A.; Fico, G. Traditional Knowledge on Medicinal and Food Plants Used in Val San Giacomo (Sondrio, Italy)—An Alpine Ethnobotanical Study. *J Ethnopharmacol* **2013**, *145*, 517–529, doi:10.1016/J.JEP.2012.11.024.
406. Emre ILKER; Hatice DEMİRAY; Oğuz BAYRAKTAR; Ali Rıza ONGUN; Berna TÜRKEKUL; İdil İPEK; Ilkay YAVAS; İsmail DEMİR; Mahmut TEPECİK; Nural KARAGÖZLÜ; et al. *Medicinal and Aromatic Plants Economics Production Agricultural Ultilization-and-Other-Aspects*; Sıdıka EKREN, Ed.; Iksad Publications.; 2021; ISBN 978-625-8007-73-2.
407. Pieroni, A.; Sõukand, R. Are Borders More Important than Geographical Distance? The Wild Food Ethnobotany of the Boykos and Its Overlap with That of the Bukovinian Hutsuls in Western Ukraine. *https://doi.org/10.2993/0278-0771-37.2.326* **2023**, *37*, 326–345, doi:10.2993/0278-0771-37.2.326.
408. Trojanowska, A. TRADITIONAL BELARUSSIAN HERBAL MEDICINE IN MICHAL FEDEROWSKI'S STUDY: "LUD BIALORUSKI NA RUSI LITEWSKIEJ ..." *Analecta. Studia i Materiały z Dziejów Nauki* **2007**, *16*, 7–53.
409. Demnati, F.; Allache, F. Effect of Verbascum Sinuatum (Scrophulariaceae) on Oviposition of Callosobruchus Maculatus (Bruchidae). *J Crop Prot* **2014**, *3*, 327–334.

410. Blanco-Salas, J.; Hortigón-Vinagre, M.P.; Morales-Jadán, D.; Ruiz-Téllez, T.; Les, F.; López, V.; Cásedas, G.; He, Z. Searching for Scientific Explanations for the Uses of Spanish Folk Medicine: A Review on the Case of Mullein (Verbascum, Scrophulariaceae). *Biology 2021, Vol. 10, Page 618* **2021**, *10*, 618, doi:10.3390/BIOLOGY10070618.
411. McCune, L.M.; Johns, T. Symptom-Specific Antioxidant Activity of Boreal Diabetes Treatments. *Pharm Biol* **2003**, *41*, 362–370, doi:10.1076/PHBI.41.5.362.15942.
412. McCune, L.M.; Johns, T. Antioxidant Activity Relates to Plant Part, Life Form and Growing Condition in Some Diabetes Remedies. *J Ethnopharmacol* **2007**, *112*, 461–469, doi:10.1016/J.JEP.2007.04.006.
413. McCune, L.M.; Johns, T. Antioxidant Activity in Medicinal Plants Associated with the Symptoms of Diabetes Mellitus Used by the Indigenous Peoples of the North American Boreal Forest. *J Ethnopharmacol* **2002**, *82*, 197–205, doi:10.1016/S0378-8741(02)00180-0.
414. Benítez, G.; González-Tejero, M.R.; Molero-Mesa, J. Knowledge of Ethnoveterinary Medicine in the Province of Granada, Andalusia, Spain. *J Ethnopharmacol* **2012**, *139*, 429–439, doi:10.1016/J.JEP.2011.11.029.
415. Ullah, M.; Khan, M.U.; Mahmood, A.; Malik, R.N.; Hussain, M.; Wazir, S.M.; Daud, M.; Shinwari, Z.K. An Ethnobotanical Survey of Indigenous Medicinal Plants in Wana District South Waziristan Agency, Pakistan. *J Ethnopharmacol* **2013**, *150*, 918–924, doi:10.1016/J.JEP.2013.09.032.
416. Miara, M.D.; Bendif, H.; Rebbas, K.; Rabah, B.; Hammou, M.A.; Maggi, F. Medicinal Plants and Their Traditional Uses in the Highland Region of Bordj Bou Arreridj (Northeast Algeria). *J Herb Med* **2019**, *16*, 100262, doi:10.1016/J.HERMED.2019.100262.
417. de Rus Jacquet, A.; Timmers, M.; Ma, S.Y.; Thieme, A.; McCabe, G.P.; Vest, J.H.C.; Lila, M.A.; Rochet, J.C. Lumbee Traditional Medicine: Neuroprotective Activities of Medicinal Plants Used to Treat Parkinson's Disease-Related Symptoms. *J Ethnopharmacol* **2017**, *206*, 408–425, doi:10.1016/J.JEP.2017.02.021.
418. Shah, A.; Bharati, K.A.; Ahmad, J.; Sharma, M.P. New Ethnomedicinal Claims from Gujjar and Bakerwals Tribes of Rajouri and Poonch Districts of Jammu and Kashmir, India. *J Ethnopharmacol* **2015**, *166*, 119–128, doi:10.1016/J.JEP.2015.01.056.
419. Murad, W.; Ahmad, A.; Gilani, S.A.; Khan, M.A. Indigenous Knowledge and Folk Use of Medicinal Plants by the Tribal Communities of Hazar Nao Forest, Malakand District, North Pakistan. *Journal of Medicinal Plants Research* **2011**, *5*, 1072–1086.
420. Graça, V.C.; Ferreira, I.C.F.R.; Santos, P.F. Phytochemical Composition and Biological Activities of Geranium Robertianum L.: A Review. *Ind Crops Prod* **2016**, *87*, 363–378, doi:10.1016/J.INDCROP.2016.04.058.
421. Hamza, N.; Berke, B.; Umar, A.; Cheze, C.; Gin, H.; Moore, N. A Review of Algerian Medicinal Plants Used in the Treatment of Diabetes. *J Ethnopharmacol* **2019**, *238*, 111841, doi:10.1016/J.JEP.2019.111841.

422. Zielinska, S.; Jezierska-Domaradzka, A.; Wójciak-Kosior, M.; Sowa, I.; Junka, A.; Matkowski, A.M. Greater Celandine's Ups and Downs-21 Centuries of Medicinal Uses of Chelidonium Majus From the Viewpoint of Today's Pharmacology. *Front Pharmacol* **2018**, *9*, doi:10.3389/FPHAR.2018.00299.
423. Zare, G.; Diker, N.; Arıtuluk, Z.; Tatlı Çankaya, İ. Chelidonium Majus L. (Papaveraceae) Morphology, Anatomy and Traditional Medicinal Uses in Turkey. *İstanbul Journal of Pharmacy* **2021**, *51*, 123–132, doi:10.26650/ISTANBULJPHARM.2020.0074.
424. Grauso, L.; de Falco, B.; Motti, R.; Lanzotti, V. Corn Poppy, Papaver Rhoeas L.: A Critical Review of Its Botany, Phytochemistry and Pharmacology. *Phytochemistry Reviews* **2021**, *20*, 227–248, doi:10.1007/S11101-020-09676-7/TABLES/2.
425. Caprioli, G.; Iannarelli, R.; Cianfaglione, K.; Fiorini, D.; Giuliani, C.; Lucarini, D.; Papa, F.; Sagratini, G.; Vittori, S.; Maggi, F. Volatile Profile, Nutritional Value and Secretory Structures of the Berry-like Fruits of Hypericum Androsaemum L. *Food Research International* **2016**, *79*, 1–10, doi:10.1016/J.FOODRES.2015.11.021.
426. Guedes, A.P.; Amorim, L.R.; Vicente, A.M.S.; Ramos, G.; Fernandes-Ferreira, M. Essential Oils from Plants and in Vitro Shoots of Hypericum Androsaemum L. *J Agric Food Chem* **2003**, *51*, 1399–1404, doi:10.1021/JF020872F.
427. Zhang, R.; Ji, Y.; Zhang, X.; Kennelly, E.J.; Long, C. Ethnopharmacology of Hypericum Species in China: A Comprehensive Review on Ethnobotany, Phytochemistry and Pharmacology. *J Ethnopharmacol* **2020**, *254*, 112686, doi:10.1016/J.JEP.2020.112686.
428. González-Tejero, M.R.; Casares-Porcel, M.; Sánchez-Rojas, C.P.; Ramiro-Gutiérrez, J.M.; Molero-Mesa, J.; Pieroni, A.; Giusti, M.E.; Censorii, E.; de Pasquale, C.; Della, A.; et al. Medicinal Plants in the Mediterranean Area: Synthesis of the Results of the Project Rubia. *J Ethnopharmacol* **2008**, *116*, 341–357, doi:10.1016/J.JEP.2007.11.045.
429. Silva, A.R.; Taofiq, O.; Ferreira, I.C.F.R.; Barros, L. Hypericum Genus Cosmeceutical Application – A Decade Comprehensive Review on Its Multifunctional Biological Properties. *Ind Crops Prod* **2021**, *159*, 113053, doi:10.1016/J.INDCROP.2020.113053.
430. EMA/HMPC *Community Herbal Monograph on Hypericum Perforatum L., Herba (Well Established Medicinal Use) EMA/HMPC/101304/2008.*; 2009;
431. Vokoua, D.; Katradi, K.; Kokkinib, S. Ethnobotanical Survey of Zagori (Epirus, Greece), a Renowned Centre of Folk Medicine in the Past. *J Ethnopharmacol* **1993**, *39*, 87–196.
432. Neves, J.M.; Cunha, S. Castanea Sativa Mill A Brief Review. In *Recent Advances in Biological Research.*; 2019; Vol. 4 ISBN 978-93-89246-50-6.

433. Pieroni, A. Medicinal Plants and Food Medicines in the Folk Traditions of the Upper Lucca Province, Italy. *J Ethnopharmacol* **2000**, *70*, 235–273, doi:10.1016/S0378-8741(99)00207-X.
434. Benítez, G.; González-Tejero, M.R.; Molero-Mesa, J. Pharmaceutical Ethnobotany in the Western Part of Granada Province (Southern Spain): Ethnopharmacological Synthesis. *J Ethnopharmacol* **2010**, *129*, 87–105, doi:10.1016/J.JEP.2010.02.016.
435. El-Hilaly, J.; Hmammouchi, M.; Lyoussi, B. Ethnobotanical Studies and Economic Evaluation of Medicinal Plants in Taounate Province (Northern Morocco). *J Ethnopharmacol* **2003**, *86*, 149–158, doi:10.1016/S0378-8741(03)00012-6.
436. Ansari, R.; Ekhlasi-Kazaj, K. *Adiantum Capillus-Veneris. L: Phytochemical Constituents, Traditional Uses and Pharmacological Properties: A Review*; 2012; Vol. 3;.
437. Rastogi, S.; Pandey, M.M.; Rawat, A.K.S. Ethnopharmacological Uses, Phytochemistry and Pharmacology of Genus Adiantum: A Comprehensive Review. *J Ethnopharmacol* **2018**, *215*, 101–119, doi:10.1016/J.JEP.2017.12.034.
438. Carrió, E.; Rigat, M.; Garnatje, T.; Mayans, M.; Parada, M.; Vallès, J. Plant Ethnoveterinary Practices in Two Pyrenean Territories of Catalonia (Iberian Peninsula) and in Two Areas of the Balearic Islands and Comparison with Ethnobotanical Uses in Human Medicine. *Evidence-based Complementary and Alternative Medicine* **2012**, *2012*, doi:10.1155/2012/896295.
439. Waswa, E.N.; Li, J.; Mkala, E.M.; Wanga, V.O.; Mutinda, E.S.; Nanjala, C.; Odago, W.O.; Katumo, D.M.; Gichua, M.K.; Gituru, R.W.; et al. Ethnobotany, Phytochemistry, Pharmacology, and Toxicology of the Genus Sambucus L. (Viburnaceae). *J Ethnopharmacol* **2022**, *292*, 115102, doi:10.1016/J.JEP.2022.115102.
440. Marković, M.; Pljevljakušić, D.; Menković, N.; Matejić, J.; Papović, O.; Stankov, J.V. Traditional Knowledge on the Medicinal Use of Plants from Genus Gentiana in the Pirot County (Serbia). *Lekovite sirovine* **2021**, 46–53, doi:10.5937/LEKSIR2141046M.
441. Parada, M.; Vallès, J.; Parada, M.; Carrió, E.; Vallès, J. Ethnobotany of Food Plants in the Alt Emporda Region (Catalonia, Iberian Peninsula) Ethnobotany of Food Plants in the Alt Empordà Region (Catalonia, Iberian Peninsula). *Journal of Applied Botany and Food Quality* **2011**, *84*, 11–25.
442. Zain-Ul-Abidin, S.; Khan, R.; Ahmad, M.; Zeeshan Bhatti, M.; Zafar, M.; Saeed, A.; Khan, N. *Ethnobotanical Survey of Highly Effective Medicinal Plants and Phytotherapies to Treat Diabetes Mellitus II in South-West Pakistan*; 2018; Vol. 17;.
443. Alarcón, R.; Pardo-de-Santayana, M.; Priestley, C.; Morales, R.; Heinrich, M. Medicinal and Local Food Plants in the South of Alava (Basque Country, Spain). *J Ethnopharmacol* **2015**, *176*, 207–224, doi:10.1016/j.jep.2015.10.022.
444. Passalacqua, N.G.; Guarrera, P.M.; De Fine, G. Contribution to the Knowledge of the Folk Plant Medicine in Calabria Region (Southern Italy). *Fitoterapia* **2007**, *78*, 52–68, doi:10.1016/J.FITOTE.2006.07.005.

445. Leonti, M.; Casu, L.; Sanna, F.; Bonsignore, L. A Comparison of Medicinal Plant Use in Sardinia and Sicily—De Materia Medica Revisited? *J Ethnopharmacol* **2009**, *121*, 255–267, doi:10.1016/J.JEP.2008.10.027.
446. Akbulut, S.; Zeynep, Z. Ethnobotanical Survey of Wild Plants Used in Gümüşhane Province (Turkey). *Bol Latinoam Caribe Plantas Med Aromat* **2023**, *22*, 237–254, doi:10.37360/blacpma.23.22.2.18.
447. Iranshahy, M.; Javadi, B.; Iranshahi, M.; Jahanbakhsh, S.P.; Mahyari, S.; Hassani, F.V.; Karimi, G. A Review of Traditional Uses, Phytochemistry and Pharmacology of Portulaca Oleracea L. *J Ethnopharmacol* **2017**, *205*, 158–172, doi:10.1016/J.JEP.2017.05.004.
448. Gürbüz, İ.; Özatkan, G.; Akaydin, G.; Günbatan, T. Ethnopharmacobotanical Findings of Medicinal Plants in the Kızılcahamam District of Ankara, Turkey. *Turk J Pharm Sci* **2021**, *18*, 667–682, doi:10.4274/TJPS.GALENOS.2021.12574.
449. Younessi-Hamzekhanlu, M.; Ozturk, M.; Altay, V.; Nojadeh, M.S.; Alakbarli, F. Ethnopharmacological Study of Medicinal Plants from Khoy City of West Azerbaijan- Iran. *Indian Journal of Traditional Knowledge* **2020**, *19*, 251–267.
450. El Amrani, F.; Rhallab, A.; Alaoui, T.; El Badaoui, K.; Chakir, S. Étude Ethnopharmacologique de Quelques Plantes Utilisées Dans Le Traitement Du Diabète Dans La Région de Meknès- Tafilalet (Maroc). *Phytotherapie* **2010**, *8*, 161–165, doi:10.1007/S10298-010-0552-Y/METRICS.
451. Hashmi, M.A.; Khan, A.; Hanif, M.; Farooq, U.; Perveen, S. Traditional Uses, Phytochemistry, and Pharmacology of Olea Europaea (Olive). *Evidence-based Complementary and Alternative Medicine* **2015**, *2015*, doi:10.1155/2015/541591.
452. Chen, X.; Dai, X.; Liu, Y.; Yang, Y.; Yuan, L.; He, X.; Gong, G. Solanum Nigrum Linn.: An Insight into Current Research on Traditional Uses, Phytochemistry, and Pharmacology. *Front Pharmacol* **2022**, *13*, 918071, doi:10.3389/FPHAR.2022.918071/BIBTEX.
453. Aburjai, T.A.; Oun, I.M.; Auzi, A.A.; Hudaib, M.M. Volatile Oil Constituents of Fruits and Leaves of Solanum Nigrum L. Growing in Libya. *Journal of Essential Oil Bearing Plants* **2014**, *17*, 397–404, doi:10.1080/0972060X.2014.895194.
454. El Amrani, F.; Rhallab, A.; Alaoui, T.; El Badaoui, K.; Chakir, S. Étude Ethnopharmacologique de Quelques Plantes Utilisées Dans Le Traitement Du Diabète Dans La Région de Meknès- Tafilalet (Maroc). *Phytotherapie* **2010**, *8*, 161–165, doi:10.1007/S10298-010-0552-Y.
455. Cruz, E.C.; Andrade-Cetto, A. Ethnopharmacological Field Study of the Plants Used to Treat Type 2 Diabetes among the Cakchiquels in Guatemala. *J Ethnopharmacol* **2015**, *159*, 238–244, doi:10.1016/J.JEP.2014.11.021.
456. Masunda, A.T.; Liyongo Inkoto, C.; Ngiala Bongo, G.; Desiré, J.; Oloko, O.W.; Ngbolua, K.-T.-N.; Tshibangu, D.S.; Dinangayi Tshilanda, D.; Tshimankinda Mpiana, P.; Gédéon, N.; et al. Ethnobotanical and Ecological Studies of Plants Used in

the Treatment of Diabetes in Kwango, Kongo Central and Kinshasa in the Democratic Republic of the Congo. *International Journal of Diabetes and Endocrinology* **2019**, *4*, 18–25, doi:10.11648/j.ijde.20190401.14.

457. Kamdem, B.P.; Le Doux Kamto, E.; Paumo, H.K.; Katata-Seru, L.M.; Pegnyemb, D.E.; Igne, F.E. Chemical Constituents, Ethnomedicinal Uses, Pharmacology, and Toxicity of Dysphania Ambrosioides (L.) Mosyakin & Clemants, Formerly Chenopodium Ambrosioides L. *Nat Prod J* **2021**, *12*, doi:10.2174/2210315511666210920144526.

458. Bais, S.; Gill, N.S.; Rana, N.; Shandil, S. A Phytopharmacological Review on a Medicinal Plant: Juniperus Communis. **2014**, doi:10.1155/2014/634723.

459. Živković, J.; Ilić, M.; Šavikin, K.; Zdunić, G.; Ilić, A.; Stojković, D. Traditional Use of Medicinal Plants in South-Eastern Serbia (Pčinja District): Ethnopharmacological Investigation on the Current Status and Comparison With Half a Century Old Data. *Front Pharmacol* **2020**, *11*, 565141, doi:10.3389/FPHAR.2020.01020/BIBTEX.

460. Rana, C.S.; Sharma, A.; Kumar, N.; Dangwal, L.R.; Tiwari, J.K. *Ethnopharmacology of Some Important Medicinal Plants of Nanda Devi National Park (NDNP) Uttarakhand, India*; 2010; Vol. 8;.

461. Leduc, C.; Coonishish, J.; Haddad, P.; Cuerrier, A. Plants Used by the Cree Nation of Eeyou Istchee (Quebec, Canada) for the Treatment of Diabetes: A Novel Approach in Quantitative Ethnobotany. *J Ethnopharmacol* **2006**, *105*, 55–63, doi:10.1016/J.JEP.2005.09.038.

462. Wang, B.; Guan, C.; Fu, Q. The Traditional Uses, Secondary Metabolites, and Pharmacology of Lycopodium Species. *Phytochemistry Reviews 2021 21:1* **2021**, *21*, 1–79, doi:10.1007/S11101-021-09746-4.

463. Namsa, N.D.; Tag, H.; Mandal, M.; Kalita, P.; Das, A.K. An Ethnobotanical Study of Traditional Anti-Inflammatory Plants Used by the Lohit Community of Arunachal Pradesh, India. *J Ethnopharmacol* **2009**, *125*, 234–245, doi:10.1016/J.JEP.2009.07.004.

464. Pieroni, A.; Quave, C.L.; Villanelli, M.L.; Mangino, P.; Sabbatini, G.; Santini, L.; Boccetti, T.; Profili, M.; Ciccioli, T.; Rampa, L.G.; et al. Ethnopharmacognostic Survey on the Natural Ingredients Used in Folk Cosmetics, Cosmeceuticals and Remedies for Healing Skin Diseases in the Inland Marches, Central-Eastern Italy. *J Ethnopharmacol* **2004**, *91*, 331–344, doi:10.1016/J.JEP.2004.01.015.

465. Taïbi, K.; Aït Abderrahim, L.; Boussaid, M.; Taibi, F.; Achir, M.; Souana, K.; Benaissa, T.; Farhi, K.H.; Naamani, F.Z.; Nait Said, K. Unraveling the Ethnopharmacological Potential of Medicinal Plants Used in Algerian Traditional Medicine for Urinary Diseases. *Eur J Integr Med* **2021**, *44*, 101339, doi:10.1016/J.EUJIM.2021.101339.

466. Piwowarski, J.P.; Granica, S.; Kiss, A.K. Lythrum Salicaria L.—Underestimated Medicinal Plant from European Traditional Medicine. A Review. *J Ethnopharmacol* **2015**, *170*, 226–250, doi:10.1016/J.JEP.2015.05.017.

467. Mehrnia, M.; Akaberi, M.; Amiri, M.S.; Nadaf, M.; Emami, S.A. Ethnopharmacological Studies of Medicinal Plants in Central Zagros, Lorestan Province, Iran. *J Ethnopharmacol* **2021**, *280*, 114080, doi:10.1016/J.JEP.2021.114080.

468. Senouci, F.; Ababou, A.; Chouieb, M. Ethnobotanical Survey of the Medicinal Plants Used in the Southern Mediterranean. Case Study: The Region of Bissa (Northeastern Dahra Mountains, Algeria). *Pharmacognosy Journal* **2019**, *11*, 647–659, doi:10.5530/pj.2019.11.103.

469. Zucca, P.; Pintus, M.; Manzo, G.; Nieddu, M.; Steri, D.; Rinaldi, A.C. Antimicrobial, Antioxidant and Anti-Tyrosinase Properties of Extracts of the Mediterranean Parasitic Plant Cytinus Hypocistis. *BMC Res Notes* **2015**, *8*, 1–9, doi:10.1186/S13104-015-1546-5/TABLES/3.

470. Cocco, E.; Maccioni, D.; Sanjust, E.; Falconieri, D.; Farris, E.; Maxia, A. Ethnopharmacobotany and Diversity of Mediterranean Endemic Plants in Marmilla Subregion, Sardinia, Italy. *Plants 2022, Vol. 11, Page 3165* **2022**, *11*, 3165, doi:10.3390/PLANTS11223165.

471. Papp, N.; Bartha, S.; Boris, G.; Balogh, L. Traditional Uses of Medicinal Plants for Respiratory Diseases in Transylvania. *NPC Natural Product Communications* **2011**, *6*, 1459–1460, doi:10.1186/1746-4269-6-10.

472. Sak, K.; Jürisoo, K.; Raal, A.; Jü Risoo, K. Pharmaceutical Biology Estonian Folk Traditional Experiences on Natural Anticancer Remedies: From Past to the Future. *John M. Pezzuto Pharm Biol* **2014**, *52*, 855–866, doi:10.3109/13880209.2013.871641.

473. Kızılarslan, Ç.; Sevg, E. Ethnobotanical Uses of Genus Pinus L. (Pinaceae) in Turkey. *Indian Journal of Traditional Knowledge* **2013**, *12*, 209–220.

474. Khouchlaa, A.; El Menyiy, N.; Guaouguaou, F.E.; El Baaboua, A.; Charfi, S.; Lakhdar, F.; El Omari, N.; Taha, D.; Shariati, M.A.; Rebezov, M.; et al. Ethnomedicinal Use, Phytochemistry, Pharmacology, and Toxicology of Daphne Gnidium: A Review. *J Ethnopharmacol* **2021**, *275*, 114124, doi:10.1016/J.JEP.2021.114124.

475. Agelet, A.; Vallès, J. Studies on Pharmaceutical Ethnobotany in the Region of Pallars (Pyrenees, Catalonia, Iberian Peninsula). Part II. New or Very Rare Uses of Previously Known Medicinal Plants. *J Ethnopharmacol* **2003**, *84*, 211–227, doi:10.1016/S0378-8741(02)00319-7.

476. Zayneb, A.; Hayat, A.; Bouchra, A.; Youssef, H.; Hanane, L. Herbal Medicines Use among Diabetic Patients in Oriental Morocco. *Journal of Pharmacognosy Phytotherapy* **2015**, *7*, 9, doi:https://doi.org/10.5897/JPP2014.0338.

477. M., L.; M., L.O.; L., Z.; A., E.H.; A., O.; A., M. Ethnopharmacological Survey of Plants Used for the Treatment of Diabetes in the Town of Sidi Slimane (Morocco). *Journal of Pharmacognosy and Phytotherapy* **2017**, *9*, 101–110, doi:10.5897/JPP2016.0437.

478. Iatrou G.; Kokkalou E. Rarity, Conservation, Importance and Ethnopharmacological Knowledge of the Greek Flora. In *Identification of wild food and non-food plants of the Mediterranean region.*; Heywood V.H., Skoula M., Eds.; Identification of wild food and non-food plants of the Mediterranean region., 1997; pp. 65–75.
479. Labiad, H.; Et-tahir, A.; Ghanmi, M.; Satrani, B.; Aljaiyash, A.; Chaouch, A.; Fadli, M. Ethnopharmacological Survey of Aromatic and Medicinal Plants of the Pharmacopoeia of Northern Morocco. *Ethnobotany Research and Applications* **2020**, *19*, 1–16.
480. Gray, A.M.; Flatt, P.R. Nature's Own Pharmacy: The Diabetes Perspective. *Proceedings of the Nutrition Society* **1997**, *56*, 507–517, doi:10.1079/PNS19970051.
481. Zanoli, P.; Zavatti, M. Pharmacognostic and Pharmacological Profile of Humulus Lupulus L. *J Ethnopharmacol* **2008**, *116*, 383–396, doi:10.1016/J.JEP.2008.01.011.
482. Korpelainen, H.; Pietiläinen, M. Hop (Humulus Lupulus L.): Traditional and Present Use, and Future Potential. *Econ Bot* **2021**, *75*, 302–322, doi:10.1007/S12231-021-09528-1/TABLES/2.
483. Kumhar, I.P.; Salim, M.; Prajapati, P. *Ethnobotanical Studies on Plants Used in Folk Medicines to Cure of Diabetes in Gondwana of Vindhyan Region of Madhya Pradesh India*; 2017; Vol. 5;.
484. Rehman, K.; Mashwani, Z.U.R.; Khan, M.A.; Ullah, Z.; Chaudhary, H.J. An Ethno Botanical Perspective of Traditional Medicinal Plants from the Khattak Tribe of Chonthra Karak, Pakistan. *J Ethnopharmacol* **2015**, *165*, 251–259, doi:10.1016/J.JEP.2015.02.035.
485. Tariq, A.; Adnan, M.; Iqbal, A.; Sadia, S.; Fan, Y.; Nazar, A.; Mussarat, S.; Ahmad, M.; Olatunji, O.A.; Begum, S.; et al. Ethnopharmacology and Toxicology of Pakistani Medicinal Plants Used to Treat Gynecological Complaints and Sexually Transmitted Infections. *South African Journal of Botany* **2018**, *114*, 132–149, doi:10.1016/J.SAJB.2017.11.004.
486. Sadat-Hosseini, M.; Farajpour, M.; Boroomand, N.; Solaimani-Sardou, F. Ethnopharmacological Studies of Indigenous Medicinal Plants in the South of Kerman, Iran. *J Ethnopharmacol* **2017**, *199*, 194–204, doi:10.1016/J.JEP.2017.02.006.
487. Benarba, B. Bryonia Dioica, an Antibreast Cancer Plant: Ethnobotanical Study. *Journal of Medicinal Herbs and Ethnomedicine* **2015**, 1, doi:10.5455/jmhe.2015.09.023.
488. Bourhia, M.; Abdelaziz Shahat, A.; Mohammed Almarfadi, O.; Ali Naser, F.; Mostafa Abdelmageed, W.; Ait Haj Said, A.; El Gueddari, F.; Naamane, A.; Benbacer, L.; Khlil, N. Ethnopharmacological Survey of Herbal Remedies Used for the Treatment of Cancer in the Greater Casablanca-Morocco. *Evidence-based Complementary and Alternative Medicine* **2019**, *2019*, doi:10.1155/2019/1613457.

489. Jarić, S.; Popović, Z.; Mačukanović-Jocić, M.; Djurdjević, L.; Mijatović, M.; Karadžić, B.; Mitrović, M.; Pavlović, P. An Ethnobotanical Study on the Usage of Wild Medicinal Herbs from Kopaonik Mountain (Central Serbia). *J Ethnopharmacol* **2007**, *111*, 160–175, doi:10.1016/J.JEP.2006.11.007.
490. Marc, E.B.; Nelly, A.; Annick, D.D.; Frederic, D. Plants Used as Remedies Antirheumatic and Antineuralgic in the Traditional Medicine of Lebanon. *J Ethnopharmacol* **2008**, *120*, 315–334, doi:10.1016/J.JEP.2008.08.024.
491. Mukherjee, P.K.; Singha, S.; Kar, A.; Chanda, J.; Banerjee, S.; Dasgupta, B.; Haldar, P.K.; Sharma, N. Therapeutic Importance of Cucurbitaceae: A Medicinally Important Family. *J Ethnopharmacol* **2022**, *282*, 114599, doi:10.1016/J.JEP.2021.114599.
492. Bruni, A.; Ballero, M.; Poli, F. Quantitative Ethnopharmacological Study of the Campidano Valley and Urzulei District, Sardinia, Italy. *J Ethnopharmacol* **1997**, *57*, 97–124, doi:10.1016/S0378-8741(97)00055-X.
493. Guarrera, P.M.; Forti, G.; Marignoli, S. Ethnobotanical and Ethnomedicinal Uses of Plants in the District of Acquapendente (Latium, Central Italy). *J Ethnopharmacol* **2005**, *96*, 429–444, doi:10.1016/J.JEP.2004.09.014.
494. Khare, C. *Indian Medicinal Plants: An Illustrated Dictionary*; 2008;
495. Hajam, T.A.; H, S. Phytochemistry, Biological Activities, Industrial and Traditional Uses of Fig (Ficus Carica): A Review. *Chem Biol Interact* **2022**, *368*, 110237, doi:10.1016/J.CBI.2022.110237.
496. Mawa, S.; Husain, K.; Jantan, I. Ficus Carica L. (Moraceae): Phytochemistry, Traditional Uses and Biological Activities. *Evid Based Complement Alternat Med* **2013**, *2013*, doi:10.1155/2013/974256.
497. Gras, A.; Serrasolses, G.; Vallès, J.; Garnatje, T. Traditional Knowledge in Semi-Rural Close to Industrial Areas: Ethnobotanical Studies in Western Gironès (Catalonia, Iberian Peninsula). *J Ethnobiol Ethnomed* **2019**, *15*, 1–37, doi:10.1186/S13002-019-0295-2/FIGURES/4.
498. van Wyk, B.E. A Review of Khoi-San and Cape Dutch Medical Ethnobotany. *J Ethnopharmacol* **2008**, *119*, 331–341, doi:10.1016/J.JEP.2008.07.021.
499. Mechaala, S.; Bouatrous, Y.; Adouane, S. Traditional Knowledge and Diversity of Wild Medicinal Plants in El Kantara's Area (Algerian Sahara Gate): An Ethnobotany Survey. *Acta Ecologica Sinica* **2022**, *42*, 33–45, doi:10.1016/J.CHNAES.2021.01.007.
500. Cavender, A. Folk Medical Uses of Plant Foods in Southern Appalachia, United States. *J Ethnopharmacol* **2006**, *108*, 74–84, doi:10.1016/J.JEP.2006.04.008.
501. Guarrera, P.M. Traditional Antihelmintic, Antiparasitic and Repellent Uses of Plants in Central Italy. *J Ethnopharmacol* **1999**, *68*, 183–192, doi:10.1016/S0378-8741(99)00089-6.

502. Alonso-Castro, A.J.; Domínguez, F.; Zapata-Morales, J.R.; Carranza-Álvarez, C. Plants Used in the Traditional Medicine of Mesoamerica (Mexico and Central America) and the Caribbean for the Treatment of Obesity. *J Ethnopharmacol* **2015**, *175*, 335–345, doi:10.1016/J.JEP.2015.09.029.
503. Afolayan, A.J.; Grierson, D.S.; Mbeng, W.O. Ethnobotanical Survey of Medicinal Plants Used in the Management of Skin Disorders among the Xhosa Communities of the Amathole District, Eastern Cape, South Africa. *J Ethnopharmacol* **2014**, *153*, 220–232, doi:10.1016/J.JEP.2014.02.023.
504. Uncini Manganelli, R.E.; Tomei, P.E. Ethnopharmacobotanical Studies of the Tuscan Archipelago. *J Ethnopharmacol* **1999**, *65*, 181–202, doi:10.1016/S0378-8741(98)00177-9.
505. Inta, A.; Shengji, P.; Balslev, H.; Wangpakapattanawong, P.; Trisonthi, C. A Comparative Study on Medicinal Plants Used in Akha's Traditional Medicine in China and Thailand, Cultural Coherence or Ecological Divergence? *J Ethnopharmacol* **2008**, *116*, 508–517, doi:10.1016/J.JEP.2007.12.015.
506. Iwalewa, E.O.; McGaw, L.J.; Naidoo, V.; Eloff, J.N. Inflammation: The Foundation of Diseases and Disorders. A Review of Phytomedicines of South African Origin Used to Treat Pain and Inflammatory Conditions. *Afr J Biotechnol* **2007**, *6*, 2868–2885, doi:10.5897/AJB2007.000-2457.
507. Amala, S. *Traditional Herbal Medicines for Modern Times Traditional*; Amala, S., Ed.; CRC Press Taylor & Francis Group , 2006; ISBN 0-415-33464-0.
508. Sher, H.; Aldosari, A.; Ali, A.; De Boer, H.J. Indigenous Knowledge of Folk Medicines among Tribal Minorities in Khyber Pakhtunkhwa, Northwestern Pakistan. *J Ethnopharmacol* **2015**, *166*, 157–167, doi:10.1016/J.JEP.2015.03.022.
509. Güneş, S.; Savran, A.; Paksoy, M.Y.; Koşar, M.; Çakılcıoğlu, U. Ethnopharmacological Survey of Medicinal Plants in Karaisalı and Its Surrounding (Adana-Turkey). *J Herb Med* **2017**, *8*, 68–75, doi:10.1016/J.HERMED.2017.04.002.
510. Li, X.; Li, Y.; Ma, S.; Zhao, Q.; Wu, J.; Duan, L.; Xie, Y.; Wang, S. Traditional Uses, Phytochemistry, and Pharmacology of Ailanthus Altissima (Mill.) Swingle Bark: A Comprehensive Review. *J Ethnopharmacol* **2021**, *275*, 114121, doi:10.1016/J.JEP.2021.114121.
511. Khajuria, A.K.; Manhas, R.K.; Kumar, H.; Bisht, N.S. Ethnobotanical Study of Traditionally Used Medicinal Plants of Pauri District of Uttarakhand, India. *J Ethnopharmacol* **2021**, *276*, 114204, doi:10.1016/J.JEP.2021.114204.
512. Naz, A.; Amjad, M.S.; Umair, M.; Altaf, M.; Ni, J. Medicinal Plants Used as Therapeutic Medicine in the Himalayan Region of Azad Jammu and Kashmir. *South African Journal of Botany* **2022**, *150*, 194–216, doi:10.1016/J.SAJB.2022.07.007.

513. Negi, V.S.; Maikhuri, R.K.; Maletha, A.; Phondani, P.C. Ethnobotanical Knowledge and Population Density of Threatened Medicinal Plants of Nanda Devi Biosphere Reserve, Western Himalaya, India. *Iran J Sci Technol Trans A Sci* **2019**, *43*, 63–73, doi:10.1007/S40995-018-0545-5/FIGURES/3.
514. Cavero, R.Y.; Calvo, M.I. Medicinal Plants Used for Musculoskeletal Disorders in Navarra and Their Pharmacological Validation. *J Ethnopharmacol* **2015**, *168*, 255–259, doi:10.1016/J.JEP.2015.03.078.
515. Ghourri, M.; Zidane, L.; Douira, A. Catalogue Des Plantes Médicinales Utilisées Dans Le Traitement de La Lithiase Rénale Dans La Province de Tan-Tan (Maroc Saharien). *Int J Biol Chem Sci* **2014**, *7*, 1688, doi:10.4314/IJBCS.V7I4.24.
516. Tahraoui, A.; El-Hilaly, J.; Israili, Z.H.; Lyoussi, B. Ethnopharmacological Survey of Plants Used in the Traditional Treatment of Hypertension and Diabetes in South-Eastern Morocco (Errachidia Province). *J Ethnopharmacol* **2007**, *110*, 105–117, doi:10.1016/J.JEP.2006.09.011.
517. Pieroni, A.; Quave, C.; Nebel, S.; Heinrich, M. Ethnopharmacy of the Ethnic Albanians (Arbëreshë) of Northern Basilicata, Italy. *Fitoterapia* **2002**, *73*, 217–241, doi:10.1016/S0367-326X(02)00063-1.
518. Muhammad, P.Z.K.; Ahmad, M.; Zafar, M.; Sultana, S.; Ali, M.I.; Sun, H. Ethnomedicinal Uses of Edible Wild Fruits (EWFs) in Swat Valley, Northern Pakistan. *J Ethnopharmacol* **2015**, *173*, 191–203, doi:10.1016/J.JEP.2015.07.029.
519. Ginko, E.; Alajmovic Demirović, E.; Šarić-Kundalić, B. Ethnobotanical Study of Traditionally Used Plants in the Municipality of Zavidovići, BiH. *J Ethnopharmacol* **2023**, *302*, 115888, doi:10.1016/J.JEP.2022.115888.
520. Akerreta, S.; Calvo, M.I.; Cavero, R.Y. Ethnoveterinary Knowledge in Navarra (Iberian Peninsula). *J Ethnopharmacol* **2010**, *130*, 369–378, doi:10.1016/J.JEP.2010.05.023.
521. Di Novella, R.; Di Novella, N.; De Martino, L.; Mancini, E.; De Feo, V. Traditional Plant Use in the National Park of Cilento and Vallo Di Diano, Campania, Southern, Italy. *J Ethnopharmacol* **2013**, *145*, 328–342, doi:10.1016/J.JEP.2012.10.065.
