# Supplementary material for: Overview of Ethnobotanical–Pharmacological Studies Carried Out on Medicinal Plants from the Serra da Estrela Natural Park: Focus on Their Antidiabetic Potential
Source: Pharmaceutics. 2024 Mar 25;16(4):454. doi: 10.3390/pharmaceutics16040454 (PMC11054966; doi:10.3390/pharmaceutics16040454)
Supplement: Supplementary file 1 [file pharmaceutics-16-00454-s001.zip › Suplementery Tables S1 (1).pdf]

Supplementary Tables S1: Description of botanical data and search methodology for Serra da Estrela Natural Park plants in the Flora-on database.

Search results Flora-On; "893 taxa".

Consultation date: "21/04/2023".

Territory: "Mainland Portugal"

USE OF THIS DATA;" IMPLIES REFERENCE TO FLORA-ON."

Link to this search: "https://flora-on.pt/#1serra_da_estrela

Search carried out: "Site of Community Interest: Serra da Estrela"; "Site of Community Interest: Serra da Estrela".

Collaborators who provided data: "A.Silva;P.V.Araújo; D.T.Holyoak; J.D.Almeida; E.Portela-Pereira; J.Jansen; F.Clamote; M.Porto; A.Carapeto; P.Silveira; J.T.Tavares; P.Beja; P.Pereira; S.Ribeiro; P.Ministro; A.Matos; C.Meireles; E.Marabuto; A.J.Pereira; P.Guillaumeau; J.Pessoa; F.Covelo; AOSP; J.Costa; C.Aguiar; C.Vila-Viçosa; H.Engels; R.Lima; B.Moreira; A.Clemente; A.Francisco; P.R.González; D.Frade; J.M.R.P.†; A.Amado; R.Faria; A.T.Blasi; U.Schwarzer; M.Geraldes; P.Lemos; C.P.Gomes; M.Raposo; C.T.Gomes; J.Farminhão; J.Diamantino; D.Silva; J.Brehm D.Pinto"

Taxa corresponding to the research:

Family; "Taxon"; "Degree of endemism"; "Naturalness"; "Annexes DL 49/2005"; "IUCN category of extinction risk"; "No. Of squares"; "Observers in the site".

1	"Alismataceae;""Baldellia alpestris"";""Autóctone"";""Endémica da Península Ibérica"";"""";"""";43;""A.Silva J.Jansen "
2	"Amaranthaceae;""Chenopodium ambrosioides"";""Introduzida"";"""";"""";"""";85;""A.Silva"""
3	"Amaranthaceae;""Chenopodium botrys"";""Autóctone"";"""";"""";"""";14;""A.Silva"""
4	"Amaryllidaceae;""Allium massaessylum"";""Autóctone"";"""";"""";"""";51;""F.Clamote"
5	"Amaryllidaceae;""Allium scorzonerifolium"";""Autóctone"";"""";"""";"""";24;""A.Silva J.Jansen F.Clamote J.D.Almeida"""

6	"Amaryllidaceae;""Allium sphaerocephalon"";""Autóctone"";"""";"""";"""";133;""E.Portela-Pereira F.Clamote A.Silva D.T.Holyoak P.V.Araújo"""
7	"Amaryllidaceae;""Allium victorialis"";""Autóctone"";"""";"""";""NT"";6;""A.Matos A.Silva J.Brehm J.Jansen E.Portela-Pereira M.Porto"""
8	"Amaryllidaceae;""Leucojum autumnale"";""Autóctone"";"""";"""";""LC"";253;""A.Silva"""
9	"Amaryllidaceae;""Narcissus asturiensis"";""Autóctone"";""Endémica da Península Ibérica"";""Anexo IIAnexo IV"";""NT"";17;""J.Jansen A.Silva P.Silveira A.Carapeto D.T.Holyoak S.Ribeiro M.Porto"""
10	"Amaryllidaceae;""Narcissus bulbocodium subsp. obesus"";""Autóctone"";""Endémica da Península Ibérica"";""Anexo V"";"""";68;""S.Ribeiro"""
11	"Amaryllidaceae;""Narcissus bulbocodium subsp. bulbocodium"";""Autóctone"";"""";""Anexo V"";"""";240;""F.Clamote A.Silva J.Jansen J.D.Almeida"""
12	"Amaryllidaceae;""Narcissus bulbocodium"";"""";"""";""Anexo V"";""LC"";469;""J.T.Tavares A.Carapeto J.Jansen A.Silva D.T.Holyoak A.T.Blasi B.Moreira"""
13	"Amaryllidaceae;""Narcissus calcicola"";""Endémica"";""Endémica de Portugal continental"";""Anexo IIAnexo IV"";""LC"";34;""F.Clamote"""
14	"Amaryllidaceae;""Narcissus pseudonarcissus subsp. nobilis"";""Autóctone"";""Endémica da Península Ibérica"";""Anexo IIAnexo IV"";""LC"";19;""D.Silva J.Jansen A.Carapeto"""
15	"Amaryllidaceae;""Narcissus pseudonarcissus"";"""";"""";"""";"""";52;""A.Silva"""
16	"Amaryllidaceae;""Narcissus rupicola"";""Autóctone"";""Endémica da Península Ibérica"";"""";"""";19;""A.Carapeto M.Porto A.Silva F.Clamote D.T.Holyoak P.V.Araújo"""
17	"Amaryllidaceae;""Narcissus triandrus subsp. pallidulus"";""Autóctone"";""Endémica da Península Ibérica"";""Anexo IV"";"""";77;""A.Silva P.Silveira A.Carapeto D.T.Holyoak A.Francisco"""
18	"Amaryllidaceae;""Narcissus triandrus subsp. triandrus"";""Autóctone"";""Endémica da Península Ibérica"";""Anexo IV"";"""";120;""E.Portela-Pereira S.Ribeiro"""
19	"Amaryllidaceae;""Narcissus triandrus"";"""";"""";""Anexo IV"";""LC"";324;""J.Jansen J.T.Tavares M.Porto A.Silva P.Beja A.T.Blasi B.Moreira"""
20	"Apiaceae;""Angelica major"";""Autóctone"";""Endémica da Península Ibérica"";"""";"""";14;""J.Jansen E.Portela-Pereira A.Silva J.D.Almeida F.Clamote M.Porto P.Pereira C.Aguiar A.Carapeto D.T.Holyoak"""
21	"Apiaceae;""Angelica sylvestris"";""Autóctone"";"""";"""";"""";134;""A.Silva A.Carapeto F.Clamote"""
22	"Apiaceae;""Anthriscus caucalis"";""Autóctone"";"""";"""";"""";95;""J.Jansen"""
23	"Apiaceae;""Anthriscus sylvestris"";""Autóctone"";"""";"""";"""";39;""A.Silva E.Portela-Pereira P.Pereira F.Clamote"""
24	"Apiaceae;""Apium nodiflorum"";""Autóctone"";"""";"""";"""";396;""A.Silva F.Clamote E.Portela-Pereira"""
25	"Apiaceae;""Carum verticillatum"";""Autóctone"";"""";"""";"""";167;""A.Silva J.Jansen"""

26	"Apiaceae;""Chaerophyllum temulum"";""Autóctone"";"""";"""";"""";154;""F.Clamote A.Silva E.Portela-Pereira"""
27	"Apiaceae;""Conopodium majus subsp. marizianum"";""Autóctone"";""Endémica da Península Ibérica"";"""";"""";171;""E.Portela-Pereira A.Silva F.Clamote J.Jansen D.T.Holyoak"""
28	"Apiaceae;""Conopodium pyrenaeum"";""Autóctone"";"""";"""";"""";10;""A.Silva"""
29	"Apiaceae;""Conopodium subcarneum"";""Autóctone"";""Endémica da Península Ibérica"";"""";"""";25;""A.Silva"""
30	"Apiaceae;""Daucus carota subsp. carota"";""Autóctone"";"""";"""";"""";375;""E.Portela-Pereira A.Silva"""
31	"Apiaceae;""Daucus carota"";"""";"""";"""";"""";702;""A.Silva J.Jansen"""
32	"Apiaceae;""Daucus carota subsp. maximus"";""Autóctone"";"""";"""";"""";70;""F.Clamote"""
33	"Apiaceae;""Daucus durieua"";""Autóctone"";"""";"""";"""";33;""A.Silva"""
34	"Apiaceae;""Eryngium campestre"";""Autóctone"";"""";"""";"""";324;""F.Clamote"""
35	"Apiaceae;""Eryngium duriaei"";""Autóctone"";""Endémica da Península Ibérica"";"""";""LC"";20;""J.Jansen P.V.Araújo F.Clamote A.Silva M.Porto A.Carapeto J.D.Almeida E.Marabuto P.Guillaumeau"""
36	"Apiaceae;""Eryngium tenue"";""Autóctone"";"""";"""";"""";125;""P.V.Araújo F.Clamote J.T.Tavares"""
37	"Apiaceae;""Ferula communis subsp. catalaunica"";""Autóctone"";""Endémica da Península Ibérica"";"""";"""";187;""J.Jansen F.Clamote"""
38	"Apiaceae;""Ferulago capillaris"";""Autóctone"";""Endémica da Península Ibérica"";"""";"""";15;""J.D.Almeida"""
39	"Apiaceae;""Foeniculum vulgare"";""Autóctone"";"""";"""";"""";582;""F.Clamote E.Portela-Pereira"""
40	"Apiaceae;""Heracleum sphondylium"";"""";"""";"""";"""";206;""A.Silva E.Portela-Pereira F.Clamote"""
41	"Apiaceae;""Oenanthe crocata"";""Autóctone"";"""";"""";"""";562;""A.Silva F.Clamote E.Portela-Pereira"""
42	"Apiaceae;""Physospermum cornubiense"";""Autóctone"";"""";"""";"""";82;""A.Silva E.Portela-Pereira"""
43	"Apiaceae;""Thapsia minor"";""Autóctone"";""Endémica da Península Ibérica"";"""";"""";155;""A.Silva P.Silveira F.Clamote"""
44	"Apiaceae;""Thapsia villosa"";"""";"""";"""";"""";436;""F.Clamote A.Silva"""
45	"Apiaceae;""Tordylium maximum"";""Autóctone"";"""";"""";"""";85;""E.Portela-Pereira"""
46	"Apiaceae;""Torilis arvensis subsp. purpurea"";""Autóctone"";"""";"""";"""";99;""D.T.Holyoak"""
47	"Apiaceae;""Torilis arvensis subsp. recta"";""Autóctone"";"""";"""";"""";19;""E.Portela-Pereira"""
48	"Apiaceae;""Torilis japonica"";""Autóctone"";"""";"""";"""";18;""E.Portela-Pereira"""

49	"Apocynaceae;""Vinca difformis subsp. difformis"";""Autóctone"";"""";"""";"""";216;""A.Silva E.Portela-Pereira"""
50	"Apocynaceae;""Vincetoxicum nigrum"";""Autóctone"";"""";"""";"""";69;""A.Silva E.Portela-Pereira"""
51	"Aquifoliaceae;""Ilex aquifolium"";""Autóctone"";"""";"""";""LC"";102;""E.Portela-Pereira A.Silva A.Carapeto J.Pessoa P.R.González F.Clamote J.Costa J.Jansen"""
52	"Araceae;""Arum italicum subsp. italicum"";""Autóctone"";"""";"""";""LC"";353;""A.Silva F.Clamote"""
53	"Araliaceae;""Hedera hibernica"";""Autóctone"";"""";"""";"""";270;""A.Silva E.Portela-Pereira"""
54	"Araliaceae;""Hedera maderensis subsp. iberica"";""Autóctone"";""Endémica da Península Ibérica"";"""";"""";45;""A.Silva"""
55	"Aristolochiaceae;""Aristolochia paucinervis"";""Autóctone"";"""";"""";"""";380;""F.Clamote J.Jansen P.V.Araújo A.Silva E.Portela-Pereira A.Carapeto"""
56	"Asparagaceae;""Dipcadi serotinum subsp. serotinum"";""Autóctone"";"""";"""";"""";170;""E.Portela-Pereira"""
57	"Asparagaceae;""Hyacinthoides hispanica"";""Autóctone"";"""";"""";"""";223;""E.Portela-Pereira A.Silva F.Clamote P.V.Araújo D.T.Holyoak A.Carapeto"""
58	"Asparagaceae;""Muscari comosum"";""Autóctone"";"""";"""";"""";320;""A.Silva"""
59	"Asparagaceae;""Muscari neglectum"";""Autóctone"";"""";"""";"""";92;""A.Silva"""
60	"Asparagaceae;""Ornithogalum concinnum"";""Autóctone"";""Endémica da Península Ibérica"";"""";"""";104;""D.T.Holyoak F.Clamote S.Ribeiro A.Silva J.Jansen P.Silveira P.V.Araújo J.T.Tavares"""
61	"Asparagaceae;""Ornithogalum pyrenaicum"";""Autóctone"";"""";"""";"""";125;""A.Silva E.Portela-Pereira F.Clamote A.Carapeto P.Pereira E.Marabuto"""
62	"Asparagaceae;""Paradisea lusitanica"";""Autóctone"";""Endémica da Península Ibérica"";"""";"""";46;""A.Silva"""
63	"Asparagaceae;""Polygonatum odoratum"";""Autóctone"";"""";"""";"""";109;""A.Silva A.Carapeto F.Clamote"""
64	"Asparagaceae;""Ruscus aculeatus"";""Autóctone"";"""";""Anexo V"";""LC"";534;""A.Carapeto A.Silva F.Clamote E.Portela-Pereira M.Porto P.R.González"""
65	"Asparagaceae;""Scilla autumnalis"";""Autóctone"";"""";"""";"""";268;""A.Silva"""
66	"Asparagaceae;""Scilla monophyllos"";""Autóctone"";"""";"""";"""";309;""A.Silva M.Porto P.Beja J.Jansen"""
67	"Asparagaceae;""Scilla ramburei"";""Autóctone"";"""";"""";""LC"";69;""A.Silva J.Jansen"""
68	"Asparagaceae;""Urginea maritima"";""Autóctone"";"""";"""";"""";461;""A.Silva F.Clamote"""
69	"Aspleniaceae;""Asplenium adiantum-nigrum var. adiantum-nigrum"";""Autóctone"";"""";"""";"""";59;""E.Portela-Pereira P.V.Araújo"""
70	"Aspleniaceae;""Asplenium adiantum-nigrum"";""Inexistente"";"""";"""";"""";116;""A.Silva"""

71	"Aspleniaceae;""Asplenium billotii"";""Autóctone"";"""";"""";"""";305;""F.Clamote J.Jansen P.V.Araújo A.Silva J.T.Tavares"""
72	"Aspleniaceae;""Asplenium onopteris"";""Autóctone"";"""";"""";"""";338;""P.V.Araújo A.Silva D.T.Holyoak J.T.Tavares F.Clamote J.Jansen J.D.Almeida A.Carapeto"""
73	"Aspleniaceae;""Asplenium septentrionale subsp. septentrionale"";""Autóctone"";"""";"""";""EN"";3;""J.Jansen J.D.Almeida A.Matos P.V.Araújo"""
74	"Aspleniaceae;""Asplenium trichomanes subsp. quadrivalens"";""Autóctone"";"""";"""";"""";343;""A.Silva P.V.Araújo E.Portela-Pereira A.Carapeto J.Jansen J.D.Almeida F.Clamote"""
75	"Asteraceae;""Achillea millefolium"";""Autóctone"";"""";"""";"""";148;""E.Portela-Pereira C.Meireles F.Clamote A.Silva R.Faria D.T.Holyoak P.V.Araújo J.T.Tavares"""
76	"Asteraceae;""Andryala integrifolia"";""Autóctone"";"""";"""";"""";630;""F.Clamote A.Silva J.Jansen P.Silveira J.D.Almeida P.V.Araújo M.Porto"""
77	"Asteraceae;""Anthemis arvensis subsp. arvensis"";""Autóctone"";"""";"""";"""";58;""D.T.Holyoak J.D.Almeida"""
78	"Asteraceae;""Anthemis arvensis"";"""";"""";"""";"""";197;""A.Silva J.Jansen"""
79	"Asteraceae;""Anthemis canescens"";""Autóctone"";"""";"""";""EN"";8;""A.Silva A.Matos"""
80	"Asteraceae;""Arnica montana subsp. atlantica"";""Autóctone"";"""";""Anexo V"";""NT"";42;""AOSP A.Silva J.Jansen"""
81	"Asteraceae;""Arnoseris minima"";""Autóctone"";"""";"""";"""";131;""J.Jansen M.Porto A.Silva D.T.Holyoak A.Carapeto F.Clamote P.V.Araújo"""
82	"Asteraceae;""Bellis perennis"";""Autóctone"";"""";"""";"""";148;""J.Jansen A.Silva"""
83	"Asteraceae;""Bellis sylvestris"";""Autóctone"";"""";"""";"""";206;""F.Clamote J.D.Almeida P.Beja"""
84	"Asteraceae;""Bidens aurea"";""Introduzida"";""Exótica"";"""";"""";96;""A.Silva"""
85	"Asteraceae;""Bidens frondosa"";""Introduzida"";""Exótica"";"""";"""";273;""E.Portela-Pereira F.Clamote J.T.Tavares"""
86	"Asteraceae;""Calendula arvensis"";""Autóctone"";"""";"""";"""";532;""J.Jansen A.Silva F.Clamote"""
87	"Asteraceae;""Carduus carpetanus"";""Autóctone"";""Endémica da Península Ibérica"";"""";"""";76;""F.Clamote A.Silva D.T.Holyoak P.V.Araújo J.Jansen P.Silveira"""
88	"Asteraceae;""Carduus platypus subsp. platypus"";""Autóctone"";""Endémica da Península Ibérica"";"""";"""";36;""P.V.Araújo A.Silva F.Clamote E.Portela-Pereira"""
89	"Asteraceae;""Carduus pycnocephalus"";""Introduzida"";"""";"""";"""";63;""F.Clamote"""
90	"Asteraceae;""Carduus tenuiflorus"";""Autóctone"";"""";"""";"""";384;""F.Clamote J.Jansen A.Silva E.Portela-Pereira D.T.Holyoak"""
91	"Asteraceae;""Carlina hispanica"";""Autóctone"";"""";"""";"""";450;""A.Silva F.Clamote J.T.Tavares"""

92	"Asteraceae;""Carlina racemosa"";""Autóctone"";"""";"""";"""";230;""F.Clamote"""
93	"Asteraceae;""Centaurea coutinhoi"";""Autóctone"";""Endémica da Península Ibérica"";"""";""LC"";12;""C.Meireles"""
94	"Asteraceae;""Centaurea herminii"";""Endémica"";""Endémica de Portugal continental"";""Anexo IIAnexo IV"";"""";18;""J.D.Almeida"""
95	"Asteraceae;""Centaurea langeana subsp. langeana"";""Autóctone"";""Endémica da Península Ibérica"";"""";""LC"";51;""P.Silveira A.Amado"""
96	"Asteraceae;""Centaurea nigra subsp. rivularis"";""Autóctone"";""Endémica da Península Ibérica"";"""";"""";98;""F.Clamote"""
97	"Asteraceae;""Centaurea ornata"";"""";""Endémica da Península Ibérica"";"""";"""";107;""A.Silva F.Clamote"""
98	"Asteraceae;""Centaurea paniculata"";"""";"""";"""";"""";63;""A.Silva E.Portela-Pereira"""
99	"Asteraceae;""Chamaemelum mixtum"";""Autóctone"";"""";"""";"""";367;""E.Portela-Pereira P.V.Araújo"""
100	"Asteraceae;""Chamaemelum nobile"";""Autóctone"";"""";"""";"""";195;""P.Silveira F.Clamote A.Silva J.Jansen"""
101	"Asteraceae;""Chondrilla juncea"";""Autóctone"";"""";"""";"""";321;""F.Clamote P.Ministro J.Jansen A.Silva J.T.Tavares"""
102	"Asteraceae;""Chrysanthemum segetum"";""Introduzida"";""Exótica"";"""";"""";221;""F.Clamote J.Jansen"""
103	"Asteraceae;""Cichorium intybus"";""Autóctone"";"""";"""";"""";276;""F.Clamote"""
104	"Asteraceae;""Cirsium palustre"";""Autóctone"";"""";"""";"""";155;""M.Porto A.Silva P.V.Araújo J.T.Tavares P.Silveira F.Clamote E.Portela-Pereira A.Carapeto"""
105	"Asteraceae;""Cirsium vulgare"";""Autóctone"";"""";"""";"""";269;""J.T.Tavares J.Jansen J.D.Almeida A.Silva F.Clamote D.T.Holyoak P.Beja M.Porto"""
106	"Asteraceae;""Cnicus benedictus"";""Autóctone"";"""";"""";"""";74;""F.Clamote J.D.Almeida"""
107	"Asteraceae;""Coleostephus myconis"";""Autóctone"";"""";"""";"""";494;""F.Clamote"""
108	"Asteraceae;""Conyza canadensis"";""Introduzida"";""Exótica"";"""";"""";79;""A.Silva"""
109	"Asteraceae;""Conyza sumatrensis"";""Introduzida"";""Exótica"";"""";"""";180;""E.Portela-Pereira F.Clamote"""
110	"Asteraceae;""Crepis capillaris var. capillaris"";""Autóctone"";"""";"""";"""";62;""E.Portela-Pereira"""
111	"Asteraceae;""Crepis capillaris"";"""";"""";"""";"""";453;""A.Silva D.T.Holyoak J.Jansen F.Clamote"""
112	"Asteraceae;""Crepis lampsanoides"";""Autóctone"";"""";"""";"""";91;""E.Portela-Pereira A.Silva F.Clamote J.D.Almeida D.T.Holyoak M.Porto"""
113	"Asteraceae;""Crepis vesicaria subsp. taraxacifolia"";""Autóctone"";"""";"""";"""";399;""A.Silva F.Clamote J.Jansen"""

114	"Asteraceae;""Crupina vulgaris"";""Autóctone"";"""";"""";"""";54;""A.Silva J.D.Almeida"""
115	"Asteraceae;""Dittrichia viscosa subsp. viscosa"";""Autóctone"";"""";"""";"""";392;""E.Portela-Pereira F.Clamote M.Porto"""
116	"Asteraceae;""Doronicum carpetanum subsp. pubescens"";""Autóctone"";""Endémica da Península Ibérica"";"""";"""";3;""A.Silva J.Jansen"""
117	"Asteraceae;""Doronicum carpetanum"";"""";"""";"""";""VU"";6;""E.Portela-Pereira"""
118	"Asteraceae;""Erigeron karvinskianus"";""Introduzida"";""Exótica"";"""";"""";141;""A.Silva F.Clamote J.T.Tavares M.Porto P.Beja"""
119	"Asteraceae;""Eupatorium cannabinum subsp. cannabinum"";""Autóctone"";"""";"""";"""";184;""A.Silva"""
120	"Asteraceae;""Filago lutescens"";"""";"""";"""";"""";20;""J.D.Almeida"""
121	"Asteraceae;""Galactites tomentosus"";""Autóctone"";"""";"""";"""";513;""F.Clamote J.D.Almeida J.Jansen"""
122	"Asteraceae;""Galinsoga parviflora"";""Introduzida"";""Exótica"";"""";"""";165;""J.T.Tavares F.Clamote"""
123	"Asteraceae;""Helichrysum stoechas subsp. stoechas"";""Autóctone"";"""";"""";"""";375;""J.D.Almeida A.Silva P.V.Araújo P.Silveira F.Clamote P.Ministro"""
124	"Asteraceae;""Hieracium lachenalii"";""Autóctone"";"""";"""";"""";1;""J.D.Almeida"""
125	"Asteraceae;""Hieracium laevigatum"";""Autóctone"";"""";"""";"""";3;""A.Silva"""
126	"Asteraceae;""Hieracium sabaudum"";""Autóctone"";"""";"""";"""";48;""A.Silva E.Portela-Pereira"""
127	"Asteraceae;""Hieracium schmidtii"";""Autóctone"";"""";"""";"""";2;""E.Portela-Pereira A.Silva"""
128	"Asteraceae;""Hispidella hispanica"";""Autóctone"";""Endémica da Península Ibérica"";"""";"""";102;""E.Portela-Pereira F.Clamote J.T.Tavares P.V.Araújo J.Jansen A.Silva J.D.Almeida D.T.Holyoak M.Porto A.J.Pereira"""
129	"Asteraceae;""Hypochaeris glabra"";""Autóctone"";"""";"""";"""";358;""J.Jansen J.D.Almeida A.Silva D.T.Holyoak F.Clamote P.Silveira"""
130	"Asteraceae;""Hypochaeris radicata"";""Autóctone"";"""";"""";"""";498;""J.Jansen E.Portela-Pereira A.Silva F.Clamote J.D.Almeida D.T.Holyoak M.Porto P.Silveira J.T.Tavares"""
131	"Asteraceae;""Jurinea humilis"";""Autóctone"";"""";"""";""VU"";7;""P.Silveira A.Silva"""
132	"Asteraceae;""Lactuca saligna"";""Autóctone"";"""";"""";"""";32;""A.Silva"""
133	"Asteraceae;""Lactuca serriola"";""Autóctone"";"""";"""";"""";358;""F.Clamote A.Silva E.Portela-Pereira"""
134	"Asteraceae;""Lactuca viminea subsp. chondrilliflora"";""Autóctone"";"""";"""";"""";21;""F.Clamote D.T.Holyoak"""

135	"Asteraceae;""Lactuca viminea subsp. viminea"";""Autóctone"";"""";"""";"""";10;""E.Portela-Pereira J.Jansen"""
136	"Asteraceae;""Lactuca viminea"";"""";"""";"""";"""";178;""A.Silva F.Clamote J.T.Tavares M.Porto"""
137	"Asteraceae;""Lactuca virosa"";""Autóctone"";"""";"""";"""";139;""A.Silva F.Clamote E.Portela-Pereira"""
138	"Asteraceae;""Lapsana communis subsp. communis"";""Autóctone"";"""";"""";"""";206;""A.Silva J.D.Almeida E.Portela-Pereira"""
139	"Asteraceae;""Leontodon hispidus subsp. bourgaeanus"";""Autóctone"";""Endémica da Península Ibérica"";"""";""NT"";2;""J.Jansen C.Meireles M.Porto A.Silva P.Pereira D.T.Holyoak J.D.Almeida"""
140	"Asteraceae;""Leontodon hispidus subsp. hispidus"";""Autóctone"";"""";"""";"""";6;""E.Portela-Pereira"""
141	"Asteraceae;""Leontodon hispidus"";"""";"""";"""";"""";10;""A.Silva F.Clamote"""
142	"Asteraceae;""Leontodon pyrenaicus subsp. herminicus"";""Endémica"";""Endémica de Portugal continental"";"""";"""";1;""J.D.Almeida"""
143	"Asteraceae;""Leontodon pyrenaicus subsp. cantabricus"";""Autóctone"";""Endémica da Península Ibérica"";"""";""NT"";2;""J.Jansen A.Silva"""
144	"Asteraceae;""Leontodon pyrenaicus"";"""";"""";"""";"""";4;""P.Pereira A.Silva J.Jansen M.Porto"""
145	"Asteraceae;""Leontodon taraxacoides subsp. longirostris"";""Autóctone"";"""";"""";"""";253;""A.Silva"""
146	"Asteraceae;""Leontodon taraxacoides subsp. taraxacoides"";""Autóctone"";"""";"""";"""";75;""F.Clamote"""
147	"Asteraceae;""Leontodon taraxacoides"";"""";"""";"""";"""";559;""A.Silva J.D.Almeida"""
148	"Asteraceae;""Lepidophorum repandum"";""Autóctone"";""Endémica da Península Ibérica"";"""";"""";111;""J.D.Almeida"""
149	"Asteraceae;""Leucanthemopsis flaveola subsp. flaveola"";""Autóctone"";""Endémica da Península Ibérica"";"""";"""";15;""A.Silva J.D.Almeida"""
150	"Asteraceae;""Leucanthemopsis flaveola"";"""";"""";"""";"""";38;""P.V.Araújo A.Carapeto"""
151	"Asteraceae;""Leucanthemopsis pulverulenta subsp. pulverulenta"";""Autóctone"";""Endémica da Península Ibérica"";"""";"""";22;""A.Silva"""
152	"Asteraceae;""Leucanthemum sylvaticum"";""Endémica"";""Endémica de Portugal continental"";"""";"""";15;""A.Silva"""
153	"Asteraceae;""Logfia gallica"";""Autóctone"";"""";"""";"""";257;""A.Silva J.D.Almeida"""
154	"Asteraceae;""Logfia minima"";""Autóctone"";"""";"""";"""";189;""D.T.Holyoak E.Portela-Pereira F.Clamote J.Jansen J.D.Almeida A.Silva"""

155	"Asteraceae;""Onopordum acanthium subsp. acanthium"";""Autóctone"";"""";"""";"""";52;""F.Clamote"""
156	"Asteraceae;""Phagnalon saxatile"";""Autóctone"";"""";"""";"""";431;""F.Clamote A.Silva J.D.Almeida"""
157	"Asteraceae;""Phalacrocarpum oppositifolium"";""Autóctone"";""Endémica da Península Ibérica"";"""";"""";36;""J.Jansen E.Portela-Pereira A.Carapeto F.Clamote P.V.Araújo D.T.Holyoak A.Silva E.Marabuto J.D.Almeida P.Pereira M.Porto A.J.Pereira"""
158	"Asteraceae;""Picris hieracioides subsp. longifolia"";""Autóctone"";""Endémica da Península Ibérica"";"""";"""";132;""A.Silva E.Portela-Pereira F.Clamote M.Porto P.Beja J.D.Almeida"""
159	"Asteraceae;""Pilosella castellana"";""Autóctone"";"""";"""";"""";25;""M.Porto D.T.Holyoak F.Clamote A.Silva J.Jansen P.V.Araújo E.Portela-Pereira"""
160	"Asteraceae;""Pilosella galiciana"";""Autóctone"";""Endémica da Península Ibérica"";"""";""DD"";1;""F.Clamote"""
161	"Asteraceae;""Pilosella pseudopilosella"";""Autóctone"";"""";"""";"""";40;""A.Silva"""
162	"Asteraceae;""Pseudognaphalium luteo-album"";""Autóctone"";"""";"""";"""";239;""F.Clamote"""
163	"Asteraceae;""Pulicaria odora"";""Autóctone"";"""";"""";"""";278;""A.Silva"""
164	"Asteraceae;""Pulicaria paludosa"";""Autóctone"";"""";"""";"""";305;""F.Clamote"""
165	"Asteraceae;""Rhagadiolus edulis"";""Autóctone"";"""";"""";"""";119;""E.Portela-Pereira"""
166	"Asteraceae;""Rhagadiolus stellatus"";""Autóctone"";"""";"""";"""";73;""J.Jansen"""
167	"Asteraceae;""Scorzonera angustifolia var. angustifolia"";""Autóctone"";"""";"""";"""";92;""F.Clamote"""
168	"Asteraceae;""Senecio jacobaea"";""Autóctone"";"""";"""";"""";400;""F.Clamote"""
169	"Asteraceae;""Senecio lividus"";""Autóctone"";"""";"""";"""";222;""A.Silva M.Porto"""
170	"Asteraceae;""Senecio pyrenaicus subsp. caespitosus"";""Endémica"";""Endémica de Portugal continental"";""Anexo IV"";""VU"";2;""J.D.Almeida J.Jansen A.Silva M.Porto F.Clamote P.Pereira P.V.Araújo A.Matos"""
171	"Asteraceae;""Senecio sylvaticus"";""Autóctone"";"""";"""";"""";218;""J.D.Almeida D.T.Holyoak E.Portela-Pereira M.Porto F.Clamote P.Beja P.V.Araújo A.Silva J.Jansen P.Silveira"""
172	"Asteraceae;""Senecio vulgaris"";""Autóctone"";"""";"""";"""";508;""A.Silva D.T.Holyoak F.Clamote J.D.Almeida P.Beja"""
173	"Asteraceae;""Solidago virgaurea subsp. fallit-tirones"";""Autóctone"";""Endémica da Península Ibérica"";"""";"""";1;""A.Silva F.Clamote J.D.Almeida J.Jansen"""
174	"Asteraceae;""Solidago virgaurea"";"""";"""";"""";"""";83;""M.Porto P.V.Araújo J.Jansen J.D.Almeida A.Silva E.Portela-Pereira F.Clamote J.T.Tavares"""
175	"Asteraceae;""Sonchus asper"";"""";"""";"""";"""";321;""F.Clamote A.Silva"""
176	"Asteraceae;""Sonchus oleraceus"";""Autóctone"";"""";"""";"""";557;""A.Silva J.D.Almeida E.Portela-Pereira F.Clamote"""

177	"Asteraceae;""Tanacetum corymbosum subsp. corymbosum"";""Autóctone"";"""";"""";"""";25;""P.V.Araújo A.Silva"""
178	"Asteraceae;""Tanacetum parthenium"";""Introduzida"";""Exótica"";"""";"""";14;""A.Silva"""
179	"Asteraceae;""Tanacetum vulgare"";""Introduzida"";""Exótica"";"""";"""";8;""A.Silva F.Clamote"""
180	"Asteraceae;""Taraxacum pinto-silvae"";""Autóctone"";""Endémica da Península Ibérica"";"""";"""";6;""A.Silva"""
181	"Asteraceae;""Tolpis barbata-(2)"";""Autóctone"";"""";"""";"""";406;""F.Clamote A.Silva J.D.Almeida"""
182	"Betulaceae;""Alnus glutinosa"";""Autóctone"";"""";"""";"""";442;""E.Portela-Pereira F.Clamote A.Silva A.Carapeto J.T.Tavares J.Jansen"""
183	"Betulaceae;""Betula pubescens subsp. celtiberica"";""Autóctone"";"""";"""";"""";60;""E.Portela-Pereira P.Pereira A.Silva"""
184	"Betulaceae;""Betula pubescens"";"""";"""";"""";"""";92;""A.Silva J.T.Tavares D.T.Holyoak"""
185	"Betulaceae;""Corylus avellana"";""Autóctone"";"""";"""";"""";94;""A.Silva H.Engels J.T.Tavares"""
186	"Blechnaceae;""Blechnum spicant subsp. spicant"";""Autóctone"";"""";"""";"""";220;""J.T.Tavares A.Silva M.Porto P.Beja E.Portela-Pereira F.Clamote J.Jansen J.D.Almeida P.V.Araújo"""
187	"Boraginaceae;""Anchusa undulata"";"""";"""";"""";"""";170;""P.V.Araújo"""
188	"Boraginaceae;""Echium lusitanicum"";""Autóctone"";""Endémica da Península Ibérica"";"""";"""";102;""A.Silva E.Portela-Pereira P.Pereira S.Ribeiro F.Clamote"""
189	"Boraginaceae;""Echium plantagineum"";""Autóctone"";"""";"""";"""";451;""A.Silva J.Jansen"""
190	"Boraginaceae;""Echium rosulatum subsp. rosulatum"";""Autóctone"";""Endémica da Península Ibérica"";"""";"""";94;""E.Portela-Pereira F.Clamote P.V.Araújo J.Jansen"""
191	"Boraginaceae;""Echium rosulatum"";"""";"""";"""";"""";180;""F.Clamote J.T.Tavares A.Silva"""
192	"Boraginaceae;""Lithodora prostrata"";"""";"""";"""";"""";570;""A.Silva P.V.Araújo M.Porto P.Beja A.Francisco F.Clamote"""
193	"Boraginaceae;""Myosotis discolor"";"""";"""";"""";"""";157;""A.Silva"""
194	"Boraginaceae;""Myosotis persoonii"";""Autóctone"";"""";"""";"""";16;""A.Silva D.T.Holyoak J.Jansen"""
195	"Boraginaceae;""Myosotis ramosissima"";"""";"""";"""";"""";52;""F.Clamote A.Silva"""
196	"Boraginaceae;""Myosotis secunda"";""Autóctone"";"""";"""";"""";53;""P.Silveira E.Portela-Pereira A.Silva"""
197	"Boraginaceae;""Myosotis stolonifera"";""Autóctone"";""Endémica da Península Ibérica"";"""";"""";23;""M.Porto A.Silva D.T.Holyoak J.Jansen E.Portela-Pereira P.V.Araújo"""
198	"Boraginaceae;""Myosotis welwitschii"";""Autóctone"";"""";"""";"""";87;""F.Clamote"""
199	"Boraginaceae;""Omphalodes nitida"";""Autóctone"";""Endémica da Península Ibérica"";"""";"""";186;""J.D.Almeida A.Silva"""

200	"Boraginaceae;""Pentaglottis sempervirens"";""Autóctone"";"""";"""";"""";122;""P.V.Araújo F.Clamote A.Silva E.Portela-Pereira"""
201	"Brassicaceae;""Alliaria petiolata"";""Autóctone"";"""";"""";"""";66;""E.Portela-Pereira F.Clamote A.Silva"""
202	"Brassicaceae;""Arabidopsis thaliana var. thaliana"";""Autóctone"";"""";"""";"""";140;""A.Silva M.Porto J.Jansen P.Silveira P.Beja"""
203	"Brassicaceae;""Arabis glabra"";""Autóctone"";"""";"""";"""";10;""A.Silva"""
204	"Brassicaceae;""Arabis juressi"";""Autóctone"";""Endémica da Península Ibérica"";"""";"""";14;""A.Silva"""
205	"Brassicaceae;""Arabis stenocarpa"";""Autóctone"";""Endémica da Península Ibérica"";"""";"""";16;""A.Silva"""
206	"Brassicaceae;""Barbarea intermedia"";""Autóctone"";"""";"""";"""";17;""A.Silva F.Clamote"""
207	"Brassicaceae;""Biscutella valentina subsp. valentina"";""Autóctone"";"""";"""";"""";65;""J.Jansen"""
208	"Brassicaceae;""Brassica barrelieri"";""Autóctone"";"""";"""";"""";226;""A.Silva F.Clamote P.Beja"""
209	"Brassicaceae;""Capsella bursa-pastoris"";""Autóctone"";"""";"""";"""";359;""A.Silva D.T.Holyoak P.Beja J.D.Almeida F.Clamote"""
210	"Brassicaceae;""Cardamine castellana"";""Autóctone"";""Endémica da Península Ibérica"";"""";""EN"";1;""A.Silva"""
211	"Brassicaceae;""Cardamine flexuosa"";""Autóctone"";"""";"""";"""";50;""F.Clamote A.Silva J.Jansen"""
212	"Brassicaceae;""Cardamine hirsuta"";""Autóctone"";"""";"""";"""";352;""A.Silva F.Clamote J.D.Almeida E.Portela-Pereira P.Beja"""
213	"Brassicaceae;""Cardaria draba subsp. draba"";""Autóctone"";"""";"""";"""";17;""D.T.Holyoak"""
214	"Brassicaceae;""Coincya monensis subsp. cheiranthos"";""Autóctone"";"""";"""";"""";95;""J.D.Almeida"""
215	"Brassicaceae;""Coincya monensis"";"""";"""";"""";"""";166;""A.Silva F.Clamote A.Carapeto A.Francisco"""
216	"Brassicaceae;""Coincya monensis subsp. orophila"";""Autóctone"";"""";"""";"""";6;""J.Jansen D.T.Holyoak P.Pereira S.Ribeiro E.Portela-Pereira F.Clamote J.D.Almeida A.Silva"""
217	"Brassicaceae;""Diplotaxis catholica"";""Autóctone"";"""";"""";"""";128;""A.Silva"""
218	"Brassicaceae;""Draba muralis"";""Autóctone"";"""";"""";"""";59;""A.Silva"""
219	"Brassicaceae;""Erophila verna"";""Autóctone"";"""";"""";"""";52;""D.T.Holyoak F.Clamote M.Porto"""
220	"Brassicaceae;""Erysimum lagascae"";""Autóctone"";""Endémica da Península Ibérica"";"""";""VU"";7;""J.Jansen A.Silva C.Meireles F.Clamote C.Vila-Viçosa"""

221	"Brassicaceae;""Erysimum linifolium"";""Autóctone"";""Endémica da Península Ibérica"";"""";"""";78;""P.V.Araújo A.Silva"""
222	"Brassicaceae;""Erysimum merxmuelleri"";""Autóctone"";""Endémica da Península Ibérica"";"""";"""";5;""J.Jansen A.Silva P.V.Araújo F.Clamote J.D.Almeida E.Portela-Pereira M.Porto C.Vila-Viçosa"""
223	"Brassicaceae;""Hirschfeldia incana"";""Autóctone"";"""";"""";"""";151;""E.Portela-Pereira"""
224	"Brassicaceae;""Lepidium heterophyllum"";""Autóctone"";"""";"""";"""";143;""A.Silva F.Clamote D.T.Holyoak"""
225	"Brassicaceae;""Lunaria annua"";""Introduzida"";""Exótica"";"""";"""";24;""F.Clamote"""
226	"Brassicaceae;""Murbeckiella boryi"";""Autóctone"";"""";""Anexo V"";""NT"";7;""P.Pereira J.Jansen P.Silveira F.Clamote A.Silva P.V.Araújo D.T.Holyoak J.D.Almeida S.Ribeiro A.Carapeto"""
227	"Brassicaceae;""Murbeckiella sousae"";""Endémica"";""Endémica de Portugal continental"";""Anexo IV"";""LC"";19;""J.Jansen P.Silveira A.Silva A.Carapeto"""
228	"Brassicaceae;""Raphanus raphanistrum subsp. raphanistrum"";""Autóctone"";"""";"""";"""";451;""A.Silva J.D.Almeida"""
229	"Brassicaceae;""Rorippa nasturtium-aquaticum"";""Autóctone"";"""";"""";"""";205;""A.Silva"""
230	"Brassicaceae;""Sisymbrium officinale"";""Autóctone"";"""";"""";"""";188;""A.Silva F.Clamote"""
231	"Brassicaceae;""Teesdalia coronopifolia"";""Autóctone"";"""";"""";"""";141;""A.Silva S.Ribeiro"""
232	"Brassicaceae;""Teesdalia nudicaulis"";""Autóctone"";"""";"""";"""";237;""P.Beja F.Clamote A.Silva P.Silveira J.Jansen D.T.Holyoak"""
233	"Brassicaceae;""Teesdaliopsis conferta"";""Autóctone"";""Endémica da Península Ibérica"";"""";""NT"";3;""J.Jansen A.Silva J.Diamantino C.Meireles P.Ministro J.D.Almeida M.Porto A.Matos"""
234	"Buxaceae;""Buxus sempervirens"";""Autóctone"";"""";"""";""EN"";25;""A.Carapeto"""
235	"Campanulaceae;""Campanula herminii"";""Autóctone"";""Endémica da Península Ibérica"";"""";""VU"";2;""F.Clamote A.Carapeto J.Jansen C.Meireles D.T.Holyoak A.Silva M.Porto P.Pereira J.D.Almeida P.V.Araújo E.Portela-Pereira"""
236	"Campanulaceae;""Campanula lusitanica subsp. lusitanica"";""Autóctone"";"""";"""";"""";398;""A.Silva D.T.Holyoak P.V.Araújo E.Portela-Pereira F.Clamote M.Porto J.D.Almeida J.T.Tavares"""
237	"Campanulaceae;""Campanula rapunculus"";""Autóctone"";"""";"""";"""";342;""J.Jansen P.V.Araújo A.Silva F.Clamote E.Portela-Pereira J.D.Almeida"""
238	"Campanulaceae;""Jasione crispa subsp. crispa"";""Autóctone"";""Endémica da Península Ibérica"";"""";""NT"";3;""J.Jansen A.Carapeto A.Silva P.Pereira F.Clamote S.Ribeiro"""
239	"Campanulaceae;""Jasione laevis subsp. gredensis"";""Autóctone"";""Endémica da Península Ibérica"";"""";"""";1;""J.Jansen"""
240	"Campanulaceae;""Jasione montana var. gracilis"";""Autóctone"";"""";"""";"""";6;""P.Ministro"""

241	"Campanulaceae;""Jasione montana"";""Inexistente"";"""";"""";"""";649;""E.Portela-Pereira A.Silva F.Clamote P.V.Araújo J.Jansen"""
242	"Campanulaceae;""Jasione montana var. montana"";""Autóctone"";"""";"""";"""";157;""P.Silveira P.Ministro J.D.Almeida"""
243	"Campanulaceae;""Jasione sessiliflora"";""Autóctone"";""Endémica da Península Ibérica"";"""";""LC"";39;""J.Jansen A.Silva P.Pereira D.T.Holyoak J.D.Almeida A.Carapeto"""
244	"Campanulaceae;""Legousia scabra"";""Autóctone"";"""";"""";"""";20;""J.Jansen"""
245	"Campanulaceae;""Wahlenbergia hederacea"";""Autóctone"";"""";"""";"""";161;""J.Jansen E.Portela-Pereira P.V.Araújo F.Clamote M.Porto A.Silva J.T.Tavares P.Beja D.T.Holyoak P.Silveira A.J.Pereira"""
246	"Cannabaceae;""Humulus lupulus"";""Autóctone"";"""";"""";"""";126;""F.Clamote J.Jansen"""
247	"Caprifoliaceae;""Lonicera periclymenum subsp. hispanica"";""Autóctone"";"""";"""";"""";253;""A.Silva E.Portela-Pereira F.Clamote"""
248	"Caprifoliaceae;""Lonicera periclymenum subsp. periclymenum"";""Autóctone"";"""";"""";"""";127;""A.Silva E.Portela-Pereira F.Clamote"""
249	"Caprifoliaceae;""Lonicera periclymenum"";"""";"""";"""";"""";582;""A.Silva F.Clamote P.V.Araújo M.Porto"""
250	"Caprifoliaceae;""Sambucus nigra"";""Autóctone"";"""";"""";"""";315;""F.Clamote J.T.Tavares A.Silva E.Portela-Pereira"""
251	"Caprifoliaceae;""Viburnum tinus"";""Autóctone"";"""";"""";"""";192;""P.Beja A.Silva J.T.Tavares M.Porto A.Carapeto"""
252	"Caryophyllaceae;""Agrostemma githago"";""Introduzida"";""Exótica"";"""";"""";74;""A.Silva J.Jansen"""
253	"Caryophyllaceae;""Arenaria montana subsp. montana"";""Autóctone"";"""";"""";"""";270;""E.Portela-Pereira P.V.Araújo J.D.Almeida A.Silva F.Clamote D.T.Holyoak J.Jansen P.Silveira"""
254	"Caryophyllaceae;""Arenaria querioides subsp. querioides"";""Autóctone"";""Endémica da Península Ibérica"";"""";"""";17;""F.Clamote P.V.Araújo A.Silva P.Pereira A.Carapeto J.D.Almeida S.Ribeiro"""
255	"Caryophyllaceae;""Arenaria querioides"";"""";"""";"""";"""";40;""A.Silva P.Silveira D.T.Holyoak J.M.R.P.† A.Carapeto J.Jansen"""
256	"Caryophyllaceae;""Cerastium brachypetalum subsp. brachypetalum"";""Autóctone"";"""";"""";"""";48;""J.Jansen"""
257	"Caryophyllaceae;""Cerastium diffusum subsp. diffusum"";""Autóctone"";"""";"""";"""";37;""F.Clamote"""
258	"Caryophyllaceae;""Cerastium fontanum subsp. vulgare"";""Autóctone"";"""";"""";"""";131;""A.Silva D.T.Holyoak J.D.Almeida"""

259	"Caryophyllaceae;""Cerastium glomeratum"";""Autóctone"";"""";"""";"""";441;""A.Silva J.D.Almeida M.Porto"""
260	"Caryophyllaceae;""Cerastium ramosissimum"";""Autóctone"";"""";"""";"""";13;""F.Clamote P.V.Araújo J.Jansen A.Silva D.T.Holyoak A.Carapeto M.Porto P.Beja P.Pereira"""
261	"Caryophyllaceae;""Chaetonychia cymosa"";""Autóctone"";"""";"""";"""";97;""A.Silva"""
262	"Caryophyllaceae;""Corrigiola litoralis subsp. perez-larae"";""Autóctone"";"""";"""";"""";8;""E.Portela-Pereira"""
263	"Caryophyllaceae;""Corrigiola litoralis"";"""";"""";"""";"""";312;""F.Clamote"""
264	"Caryophyllaceae;""Corrigiola telephiifolia"";"""";"""";"""";"""";149;""A.Silva P.Silveira F.Clamote"""
265	"Caryophyllaceae;""Cucubalus baccifer"";""Autóctone"";"""";"""";"""";46;""F.Clamote J.Jansen A.Silva"""
266	"Caryophyllaceae;""Dianthus langeanus"";""Autóctone"";""Endémica da Península Ibérica"";"""";"""";27;""F.Clamote"""
267	"Caryophyllaceae;""Dianthus laricifolius"";"""";"""";"""";"""";74;""A.Silva"""
268	"Caryophyllaceae;""Dianthus laricifolius subsp. laricifolius"";""Autóctone"";""Endémica da Península Ibérica"";"""";"""";37;""A.Silva P.Silveira D.T.Holyoak"""
269	"Caryophyllaceae;""Dianthus lusitanus"";""Autóctone"";"""";"""";"""";170;""F.Clamote A.Carapeto P.V.Araújo D.T.Holyoak A.Silva P.Ministro E.Portela-Pereira J.Jansen J.D.Almeida C.T.Gomes P.Beja P.Silveira"""
270	"Caryophyllaceae;""Herniaria lusitanica subsp. lusitanica"";""Autóctone"";""Endémica da Península Ibérica"";"""";"""";22;""D.T.Holyoak"""
271	"Caryophyllaceae;""Herniaria scabrida subsp. scabrida"";""Autóctone"";""Endémica da Península Ibérica"";"""";"""";67;""J.D.Almeida D.T.Holyoak"""
272	"Caryophyllaceae;""Herniaria scabrida"";"""";"""";"""";"""";86;""A.Silva P.V.Araújo"""
273	"Caryophyllaceae;""Illecebrum verticillatum"";""Autóctone"";"""";"""";"""";277;""M.Porto P.Silveira D.T.Holyoak F.Clamote P.Guillaumeau"""
274	"Caryophyllaceae;""Minuartia recurva"";""Autóctone"";"""";"""";"""";5;""J.Jansen A.Silva A.Carapeto J.D.Almeida D.T.Holyoak S.Ribeiro P.V.Araújo M.Porto F.Clamote P.Pereira J.T.Tavares A.J.Pereira P.Beja"""
275	"Caryophyllaceae;""Moehringia pentandra"";""Autóctone"";"""";"""";"""";34;""J.Jansen"""
276	"Caryophyllaceae;""Moehringia trinervia"";""Autóctone"";"""";"""";"""";16;""J.Jansen A.Silva"""
277	"Caryophyllaceae;""Moenchia erecta subsp. erecta"";""Autóctone"";"""";"""";"""";18;""D.T.Holyoak"""
278	"Caryophyllaceae;""Paronychia argentea"";"""";"""";"""";"""";293;""J.D.Almeida"""
279	"Caryophyllaceae;""Paronychia polygonifolia var. velucensis"";""Autóctone"";"""";"""";""NT"";5;""J.Jansen C.Meireles P.V.Araújo A.Silva P.Pereira M.Porto F.Clamote P.Guillaumeau A.Matos"""

280	"Caryophyllaceae;""Petrorhagia nanteuilii"";""Autóctone"";"""";"""";"""";362;""F.Clamote M.Porto A.Silva D.T.Holyoak J.D.Almeida"""
281	"Caryophyllaceae;""Polycarpon tetraphyllum subsp. tetraphyllum"";""Autóctone"";"""";"""";"""";192;""F.Clamote"""
282	"Caryophyllaceae;""Polycarpon tetraphyllum"";"""";"""";"""";"""";384;""J.D.Almeida"""
283	"Caryophyllaceae;""Sagina apetala"";""Autóctone"";"""";"""";"""";136;""D.T.Holyoak P.Ministro"""
284	"Caryophyllaceae;""Sagina procumbens"";""Autóctone"";"""";"""";"""";51;""F.Clamote D.T.Holyoak J.D.Almeida A.Silva"""
285	"Caryophyllaceae;""Sagina saginoides"";""Autóctone"";"""";"""";""EN"";4;""J.Jansen M.Porto C.Meireles A.Silva A.Matos F.Clamote D.T.Holyoak A.Carapeto"""
286	"Caryophyllaceae;""Saponaria officinalis"";""Autóctone"";"""";"""";"""";247;""A.Clemente A.Silva F.Clamote P.V.Araújo E.Portela-Pereira J.T.Tavares"""
287	"Caryophyllaceae;""Scleranthus annuus"";""Autóctone"";"""";"""";"""";65;""A.Silva D.T.Holyoak"""
288	"Caryophyllaceae;""Scleranthus perennis"";""Autóctone"";"""";"""";""CR"";1;""C.Meireles"""
289	"Caryophyllaceae;""Scleranthus polycarpos"";""Autóctone"";"""";"""";"""";45;""A.Silva D.T.Holyoak F.Clamote F.Covelo J.D.Almeida"""
290	"Caryophyllaceae;""Silene acutifolia"";""Autóctone"";""Endémica da Península Ibérica"";"""";"""";75;""J.Jansen A.Silva J.T.Tavares J.D.Almeida P.V.Araújo P.Silveira F.Clamote A.Carapeto E.Portela-Pereira M.Porto"""
291	"Caryophyllaceae;""Silene ciliata"";""Autóctone"";"""";"""";""EN"";1;""J.Jansen A.Silva P.Pereira A.Carapeto F.Clamote J.D.Almeida M.Porto A.J.Pereira D.T.Holyoak A.Matos P.V.Araújo"""
292	"Caryophyllaceae;""Silene coutinhoi"";""Autóctone"";""Endémica da Península Ibérica"";"""";"""";43;""J.Jansen"""
293	"Caryophyllaceae;""Silene foetida subsp. foetida"";""Endémica"";""Endémica de Portugal continental"";"""";""EN"";1;""J.Jansen D.T.Holyoak A.Matos P.Pereira P.V.Araújo A.Silva S.Ribeiro J.D.Almeida H.Engels F.Clamote M.Porto A.Carapeto"""
294	"Caryophyllaceae;""Silene gallica"";""Autóctone"";"""";"""";"""";486;""A.Silva F.Clamote J.D.Almeida"""
295	"Caryophyllaceae;""Silene inaperta subsp. inaperta"";""Autóctone"";"""";"""";"""";61;""A.Silva"""
296	"Caryophyllaceae;""Silene latifolia"";""Autóctone"";"""";"""";"""";418;""F.Clamote E.Portela-Pereira A.Silva J.Jansen J.D.Almeida A.Carapeto D.T.Holyoak M.Porto P.V.Araújo"""
297	"Caryophyllaceae;""Silene nutans subsp. nutans"";""Autóctone"";"""";"""";"""";141;""E.Portela-Pereira A.Carapeto J.Jansen P.V.Araújo A.Silva"""
298	"Caryophyllaceae;""Silene psammitis subsp. psammitis"";""Autóctone"";""Endémica da Península Ibérica"";"""";"""";26;""A.Silva"""
299	"Caryophyllaceae;""Silene scabriflora subsp. scabriflora"";""Autóctone"";"""";"""";"""";81;""F.Clamote P.V.Araújo"""

300	"Caryophyllaceae;""Silene scabriflora"";"""";"""";"""";"""";260;""A.Silva"""
301	"Caryophyllaceae;""Silene vulgaris subsp. vulgaris"";""Autóctone"";"""";"""";"""";198;""P.V.Araújo A.Silva"""
302	"Caryophyllaceae;""Spergula arvensis"";""Autóctone"";"""";"""";"""";325;""A.Silva F.Clamote"""
303	"Caryophyllaceae;""Spergula morisonii"";""Autóctone"";"""";"""";"""";88;""F.Clamote A.Silva J.Jansen J.D.Almeida P.Silveira D.T.Holyoak F.Covelo"""
304	"Caryophyllaceae;""Spergularia capillacea"";""Autóctone"";""Endémica da Península Ibérica"";"""";"""";42;""A.Silva F.Clamote"""
305	"Caryophyllaceae;""Spergularia media"";""Autóctone"";"""";"""";"""";33;""P.Beja"""
306	"Caryophyllaceae;""Spergularia purpurea"";""Autóctone"";"""";"""";"""";284;""F.Clamote A.Silva D.T.Holyoak"""
307	"Caryophyllaceae;""Spergularia rubra"";""Autóctone"";"""";"""";"""";28;""F.Clamote"""
308	"Caryophyllaceae;""Stellaria alsine"";""Autóctone"";"""";"""";"""";72;""F.Clamote J.Jansen M.Porto"""
309	"Caryophyllaceae;""Stellaria graminea"";""Autóctone"";"""";"""";"""";96;""A.Silva"""
310	"Caryophyllaceae;""Stellaria holostea"";""Autóctone"";"""";"""";"""";133;""A.Silva A.Carapeto J.D.Almeida"""
311	"Caryophyllaceae;""Stellaria media"";""Autóctone"";"""";"""";"""";465;""M.Porto P.Beja F.Clamote A.Silva J.D.Almeida D.T.Holyoak P.Silveira"""
312	"Cistaceae;""Cistus ladanifer subsp. ladanifer"";""Autóctone"";"""";"""";""LC"";444;""F.Clamote"""
313	"Cistaceae;""Cistus ladanifer"";"""";"""";"""";"""";666;""A.Silva"""
314	"Cistaceae;""Cistus populifolius subsp. populifolius"";""Autóctone"";"""";"""";"""";31;""E.Portela-Pereira"""
315	"Cistaceae;""Cistus populifolius"";"""";"""";"""";"""";211;""A.Silva M.Porto J.Jansen"""
316	"Cistaceae;""Cistus psilosepalus"";""Autóctone"";"""";"""";""LC"";468;""J.D.Almeida A.Silva F.Clamote M.Porto P.Beja P.V.Araújo J.Jansen A.Carapeto"""
317	"Cistaceae;""Cistus salviifolius"";""Autóctone"";"""";"""";"""";562;""A.Silva M.Porto P.Beja J.Jansen"""
318	"Cistaceae;""Halimium lasianthum subsp. alyssoides"";""Autóctone"";"""";"""";"""";208;""A.Silva E.Portela-Pereira F.Clamote J.Jansen P.V.Araújo J.D.Almeida P.Silveira D.T.Holyoak A.Carapeto"""
319	"Cistaceae;""Halimium lasianthum subsp. lasianthum"";""Autóctone"";"""";"""";"""";19;""P.Silveira F.Clamote"""
320	"Cistaceae;""Halimium lasianthum"";"""";"""";"""";"""";286;""A.Carapeto P.V.Araújo J.T.Tavares A.Silva P.Beja M.Porto F.Clamote"""
321	"Cistaceae;""Halimium ocymoides"";""Autóctone"";"""";"""";"""";135;""F.Clamote A.Silva P.Silveira"""
322	"Cistaceae;""Halimium umbellatum var. umbellatum"";""Autóctone"";"""";"""";"""";40;""A.Silva"""

323	"Cistaceae;""Halimium umbellatum"";"""";"""";"""";"""";216;""P.V.Araújo A.Silva"""
324	"Cistaceae;""Halimium umbellatum var. viscosum"";""Autóctone"";"""";"""";"""";28;""J.Jansen"""
325	"Cistaceae;""Helianthemum nummularium"";""Autóctone"";"""";"""";"""";50;""P.V.Araújo A.Silva"""
326	"Cistaceae;""Tuberaria guttata"";""Autóctone"";"""";"""";"""";528;""D.T.Holyoak F.Clamote A.Silva J.Jansen P.V.Araújo J.D.Almeida"""
327	"Cistaceae;""Tuberaria lignosa"";""Autóctone"";"""";"""";"""";194;""A.Silva J.Jansen"""
328	"Colchicaceae;""Merendera montana"";""Autóctone"";"""";"""";"""";118;""F.Clamote M.Porto A.Carapeto J.Jansen A.Silva P.V.Araújo"""
329	"Commelinaceae;""Tradescantia fluminensis"";""Introduzida"";""Exótica"";"""";"""";167;""A.Silva"""
330	"Convolvulaceae;""Convolvulus arvensis"";""Autóctone"";"""";"""";"""";461;""P.V.Araújo F.Clamote A.Silva D.T.Holyoak"""
331	"Crassulaceae;""Crassula tillaea"";""Autóctone"";"""";"""";"""";293;""A.Silva J.Jansen"""
332	"Crassulaceae;""Sedum amplexicaule"";""Autóctone"";"""";"""";"""";93;""F.Clamote E.Portela-Pereira"""
333	"Crassulaceae;""Sedum andegavense"";""Autóctone"";"""";"""";"""";77;""J.Jansen"""
334	"Crassulaceae;""Sedum anglicum"";""Autóctone"";"""";"""";"""";70;""A.Silva A.Carapeto J.T.Tavares D.Frade J.Jansen E.Portela-Pereira P.V.Araújo"""
335	"Crassulaceae;""Sedum arenarium"";""Autóctone"";""Endémica da Península Ibérica"";"""";"""";236;""J.Jansen A.Silva J.D.Almeida A.Carapeto D.T.Holyoak F.Clamote P.Silveira F.Covelo P.V.Araújo"""
336	"Crassulaceae;""Sedum brevifolium"";""Autóctone"";"""";"""";"""";282;""A.Silva A.Carapeto J.Jansen D.T.Holyoak M.Porto J.D.Almeida F.Clamote E.Portela-Pereira P.Silveira P.Ministro J.T.Tavares P.Beja S.Ribeiro P.Pereira P.V.Araújo"""
337	"Crassulaceae;""Sedum forsterianum"";""Autóctone"";"""";"""";"""";301;""A.Silva P.V.Araújo F.Clamote E.Portela-Pereira J.D.Almeida"""
338	"Crassulaceae;""Sedum hirsutum subsp. hirsutum"";""Autóctone"";"""";"""";"""";259;""P.V.Araújo M.Porto A.Carapeto E.Portela-Pereira J.D.Almeida F.Clamote P.Ministro"""
339	"Crassulaceae;""Sedum hirsutum"";"""";"""";"""";"""";404;""A.Silva D.T.Holyoak F.Clamote M.Porto J.T.Tavares A.Carapeto E.Portela-Pereira J.Jansen"""
340	"Crassulaceae;""Sedum maireanum"";""Autóctone"";"""";"""";"""";19;""A.Silva J.Jansen S.Ribeiro"""
341	"Crassulaceae;""Sedum pedicellatum subsp. lusitanicum"";""Autóctone"";""Endémica da Península Ibérica"";"""";"""";6;""C.Meireles A.Matos J.Jansen M.Porto"""
342	"Crassulaceae;""Sedum pedicellatum"";"""";"""";"""";""LC"";20;""D.T.Holyoak J.Jansen"""
343	"Crassulaceae;""Sedum pruinatum"";""Autóctone"";""Endémica da Península Ibérica"";"""";"""";37;""A.Silva M.Porto E.Portela-Pereira F.Clamote"""

344	"Crassulaceae;""Umbilicus rupestris"";""Autóctone"";"""";"""";"""";582;""F.Clamote D.T.Holyoak E.Portela-Pereira P.V.Araújo A.Silva J.D.Almeida A.Carapeto J.Jansen P.Ministro"""
345	"Cucurbitaceae;""Bryonia dioica"";""Autóctone"";"""";"""";"""";458;""J.Jansen J.D.Almeida A.Silva E.Portela-Pereira F.Clamote"""
346	"Cupressaceae;""Juniperus communis subsp. alpina"";""Autóctone"";"""";"""";"""";1;""J.Jansen J.D.Almeida"""
347	"Cupressaceae;""Juniperus communis"";"""";"""";"""";"""";8;""A.Carapeto D.T.Holyoak A.Silva P.V.Araújo F.Clamote E.Portela-Pereira P.Pereira S.Ribeiro P.Beja R.Lima"""
348	"Cyperaceae;""Carex binervis"";""Autóctone"";"""";"""";"""";35;""A.Silva F.Covelo"""
349	"Cyperaceae;""Carex demissa"";""Autóctone"";"""";"""";"""";32;""J.Jansen"""
350	"Cyperaceae;""Carex depauperata"";""Autóctone"";"""";"""";"""";2;""J.Jansen"""
351	"Cyperaceae;""Carex distachya"";""Autóctone"";"""";"""";"""";34;""A.Silva"""
352	"Cyperaceae;""Carex echinata"";""Autóctone"";"""";"""";"""";36;""J.Jansen A.Silva P.V.Araújo"""
353	"Cyperaceae;""Carex elata subsp. reuteriana"";""Autóctone"";""Endémica da Península Ibérica"";"""";"""";95;""D.T.Holyoak E.Portela-Pereira P.V.Araújo A.Silva J.T.Tavares"""
354	"Cyperaceae;""Carex furva"";""Autóctone"";""Endémica da Península Ibérica"";"""";""CR"";1;""P.Pereira A.Silva J.Jansen"""
355	"Cyperaceae;""Carex laevigata"";""Autóctone"";"""";"""";"""";79;""A.Silva J.Jansen E.Portela-Pereira"""
356	"Cyperaceae;""Carex leporina"";""Autóctone"";"""";"""";"""";77;""A.Carapeto P.Pereira J.Jansen M.Porto A.Silva D.T.Holyoak F.Clamote"""
357	"Cyperaceae;""Carex muricata subsp. pairae"";""Autóctone"";"""";"""";"""";66;""A.Silva J.Jansen E.Portela-Pereira"""
358	"Cyperaceae;""Carex nigra"";""Autóctone"";"""";"""";"""";3;""A.Silva F.Clamote P.Ministro"""
359	"Cyperaceae;""Carex remota"";""Autóctone"";"""";"""";"""";16;""A.Silva"""
360	"Cyperaceae;""Cyperus eragrostis"";""Introduzida"";""Exótica"";"""";"""";309;""F.Clamote"""
361	"Cyperaceae;""Cyperus longus"";""Autóctone"";"""";"""";"""";319;""A.Silva E.Portela-Pereira F.Clamote"""
362	"Cyperaceae;""Eleocharis multicaulis"";""Autóctone"";"""";"""";"""";68;""J.Jansen"""
363	"Cyperaceae;""Eleocharis palustris subsp. palustris"";""Autóctone"";"""";"""";"""";38;""A.Silva"""
364	"Cyperaceae;""Eriophorum angustifolium"";""Autóctone"";"""";"""";""VU"";13;""J.Jansen"""
365	"Cyperaceae;""Isolepis setacea"";""Autóctone"";"""";"""";"""";54;""A.Silva"""
366	"Cyperaceae;""Scirpoides holoschoenus"";""Autóctone"";"""";"""";"""";444;""A.Silva"""
367	"Cytinaceae;""Cytinus hypocistis subsp. hypocistis"";""Autóctone"";"""";"""";"""";5;""D.T.Holyoak"""
368	"Cytinaceae;""Cytinus hypocistis"";"""";"""";"""";"""";127;""A.Silva M.Porto"""

369	"Dennstaedtiaceae;""Pteridium aquilinum subsp. aquilinum"";""Autóctone"";"""";"""";"""";581;""E.Portela-Pereira A.Silva P.V.Araújo A.Carapeto P.Beja F.Clamote J.Jansen J.D.Almeida D.T.Holyoak J.T.Tavares M.Porto"""
370	"Dioscoreaceae;""Tamus communis"";""Autóctone"";"""";"""";"""";465;""E.Portela-Pereira F.Clamote J.Jansen A.Silva J.D.Almeida"""
371	"Droseraceae;""Drosera rotundifolia"";""Autóctone"";"""";"""";"""";37;""A.Silva P.Pereira P.Ministro H.Engels J.Jansen M.Porto P.V.Araújo J.D.Almeida"""
372	"Dryopteridaceae;""Dryopteris affinis subsp. affinis"";""Autóctone"";"""";"""";"""";81;""E.Portela-Pereira P.Ministro"""
373	"Dryopteridaceae;""Dryopteris affinis"";"""";"""";"""";"""";227;""P.V.Araújo P.Pereira J.T.Tavares J.Jansen F.Clamote"""
374	"Dryopteridaceae;""Dryopteris dilatata"";""Autóctone"";"""";"""";"""";36;""A.Silva E.Portela-Pereira"""
375	"Dryopteridaceae;""Dryopteris expansa"";""Autóctone"";"""";"""";""CR"";2;""A.Matos J.Jansen A.Silva P.V.Araújo"""
376	"Dryopteridaceae;""Dryopteris filix-mas"";""Autóctone"";"""";"""";"""";61;""E.Portela-Pereira A.Silva F.Clamote J.Jansen J.D.Almeida P.Beja P.V.Araújo"""
377	"Dryopteridaceae;""Dryopteris oreades"";""Autóctone"";"""";"""";""VU"";2;""A.Silva J.Jansen D.T.Holyoak P.Pereira F.Clamote A.Matos P.Ministro E.Portela-Pereira P.V.Araújo A.Carapeto"""
378	"Dryopteridaceae;""Polystichum setiferum"";""Autóctone"";"""";"""";"""";207;""A.Silva E.Portela-Pereira A.Carapeto F.Clamote J.T.Tavares J.Jansen J.D.Almeida P.V.Araújo P.Ministro"""
379	"Elatinaceae;""Elatine brochonii"";""Autóctone"";"""";"""";""NT"";29;""F.Clamote D.Pinto"""
380	"Ericaceae;""Arbutus unedo"";""Autóctone"";"""";"""";"""";451;""A.Silva F.Clamote J.Jansen M.Porto"""
381	"Ericaceae;""Calluna vulgaris"";""Autóctone"";"""";"""";"""";467;""J.Jansen A.Carapeto A.Silva M.Porto P.V.Araújo D.T.Holyoak P.Beja E.Portela-Pereira R.Lima F.Clamote J.T.Tavares"""
382	"Ericaceae;""Erica arborea"";""Autóctone"";"""";"""";"""";410;""A.Silva P.V.Araújo J.Jansen A.Carapeto E.Portela-Pereira D.T.Holyoak J.D.Almeida F.Clamote M.Porto"""
383	"Ericaceae;""Erica australis subsp. australis"";""Autóctone"";"""";"""";"""";257;""A.Carapeto A.Silva F.Clamote D.T.Holyoak M.Porto P.Beja E.Portela-Pereira J.Jansen E.Marabuto P.Silveira P.V.Araújo P.Ministro"""
384	"Ericaceae;""Erica ciliaris"";""Autóctone"";"""";"""";"""";183;""A.Silva"""
385	"Ericaceae;""Erica cinerea"";""Autóctone"";"""";"""";"""";230;""A.Silva A.Carapeto"""
386	"Ericaceae;""Erica lusitanica"";""Autóctone"";"""";"""";"""";146;""A.Silva P.Beja E.Portela-Pereira"""
387	"Ericaceae;""Erica scoparia subsp. scoparia"";""Autóctone"";"""";"""";"""";220;""A.Silva"""
388	"Ericaceae;""Erica tetralix"";""Autóctone"";"""";"""";"""";70;""M.Porto E.Portela-Pereira J.D.Almeida J.Jansen"""

389	"Ericaceae;""Erica umbellata"";"""";"""";"""";"""";385;""D.T.Holyoak M.Porto A.Silva F.Clamote P.Silveira"""
390	"Ericaceae;""Monotropa hypopitys"";""Autóctone"";"""";"""";""VU"";17;""P.V.Araújo A.Silva J.Pessoa J.Jansen J.Farminhão"""
391	"Ericaceae;""Vaccinium myrtillus"";""Autóctone"";"""";"""";"""";23;""A.Silva"""
392	"Ericaceae;""Vaccinium uliginosum"";""Autóctone"";"""";"""";""VU"";1;""A.Silva P.Pereira M.Porto P.V.Araújo P.Ministro"""
393	"Euphorbiaceae;""Euphorbia amygdaloides subsp. amygdaloides"";""Autóctone"";"""";"""";"""";151;""F.Clamote A.Silva"""
394	"Euphorbiaceae;""Euphorbia characias subsp. characias"";""Autóctone"";"""";"""";"""";136;""P.Silveira"""
395	"Euphorbiaceae;""Euphorbia dulcis"";""Autóctone"";"""";"""";"""";73;""J.Jansen"""
396	"Euphorbiaceae;""Euphorbia helioscopia subsp. helioscopia"";""Autóctone"";"""";"""";"""";254;""P.Beja"""
397	"Euphorbiaceae;""Euphorbia oxyphylla"";""Autóctone"";""Endémica da Península Ibérica"";"""";"""";81;""F.Clamote J.T.Tavares P.Silveira"""
398	"Euphorbiaceae;""Euphorbia peplus"";"""";"""";"""";"""";272;""P.Beja"""
399	"Euphorbiaceae;""Mercurialis ambigua"";""Autóctone"";"""";"""";"""";402;""A.Silva F.Clamote"""
400	"Fabaceae;""Acacia dealbata"";""Introduzida"";""Exótica"";"""";"""";309;""A.Silva E.Portela-Pereira F.Clamote A.Carapeto"""
401	"Fabaceae;""Acacia melanoxylon"";""Introduzida"";""Exótica"";"""";"""";144;""A.Silva"""
402	"Fabaceae;""Adenocarpus complicatus"";""Autóctone"";"""";"""";"""";148;""C.Meireles P.Pereira S.Ribeiro F.Clamote J.Jansen J.T.Tavares M.Porto"""
403	"Fabaceae;""Adenocarpus hispanicus subsp. gredensis"";""Autóctone"";""Endémica da Península Ibérica"";"""";""EN"";3;""C.P.Gomes C.Meireles S.Ribeiro A.Silva A.Carapeto J.Jansen J.D.Almeida"""
404	"Fabaceae;""Adenocarpus lainzii"";""Autóctone"";""Endémica da Península Ibérica"";"""";"""";152;""S.Ribeiro"""
405	"Fabaceae;""Anthyllis vulneraria subsp. gandogeri"";""Autóctone"";"""";"""";"""";24;""P.V.Araújo"""
406	"Fabaceae;""Astragalus glycyphyllos"";""Autóctone"";"""";"""";""EN"";3;""A.Silva J.D.Almeida A.Matos"""
407	"Fabaceae;""Astragalus pelecinus subsp. pelecinus"";""Autóctone"";"""";"""";"""";150;""J.Jansen"""
408	"Fabaceae;""Coronilla repanda subsp. dura"";""Autóctone"";"""";"""";"""";85;""J.Jansen"""
409	"Fabaceae;""Cytisus grandiflorus subsp. grandiflorus"";""Autóctone"";"""";"""";"""";68;""E.Portela-Pereira A.Silva D.T.Holyoak"""

410	"Fabaceae;""Cytisus grandiflorus"";"""";"""";"""";"""";136;""A.Silva P.V.Araújo J.Jansen P.Silveira"""
411	"Fabaceae;""Cytisus multiflorus"";""Autóctone"";""Endémica da Península Ibérica"";"""";"""";301;""F.Clamote D.T.Holyoak A.Silva P.V.Araújo E.Portela-Pereira M.Porto J.Jansen A.Carapeto"""
412	"Fabaceae;""Cytisus oromediterraneus"";""Autóctone"";"""";"""";"""";4;""J.Jansen J.D.Almeida A.Silva E.Portela-Pereira F.Clamote A.Carapeto D.T.Holyoak"""
413	"Fabaceae;""Cytisus scoparius subsp. scoparius"";""Autóctone"";"""";"""";"""";149;""A.Silva"""
414	"Fabaceae;""Cytisus striatus"";""Autóctone"";"""";"""";"""";396;""P.V.Araújo A.Silva J.T.Tavares E.Portela-Pereira J.Jansen A.Carapeto J.D.Almeida D.T.Holyoak"""
415	"Fabaceae;""Echinospartum ibericum"";""Autóctone"";""Endémica da Península Ibérica"";"""";"""";36;""P.Guillaumeau E.Portela-Pereira J.D.Almeida A.Silva P.Pereira A.Carapeto F.Clamote P.Silveira J.T.Tavares D.T.Holyoak M.Porto J.Jansen P.V.Araújo C.Aguiar"""
416	"Fabaceae;""Erophaca baetica subsp. baetica"";""Autóctone"";"""";"""";"""";226;""J.Costa"""
417	"Fabaceae;""Genista anglica"";""Autóctone"";"""";"""";"""";23;""E.Portela-Pereira M.Porto S.Ribeiro A.Silva A.Carapeto J.Jansen D.T.Holyoak J.D.Almeida M.Geraldes F.Clamote P.V.Araújo"""
418	"Fabaceae;""Genista cinerascens"";""Autóctone"";""Endémica da Península Ibérica"";"""";"""";7;""J.Jansen J.D.Almeida A.Silva E.Portela-Pereira A.Carapeto"""
419	"Fabaceae;""Genista falcata"";""Autóctone"";""Endémica da Península Ibérica"";"""";"""";143;""F.Clamote A.Silva J.Jansen P.V.Araújo J.D.Almeida A.Carapeto P.Beja E.Portela-Pereira M.Porto"""
420	"Fabaceae;""Genista florida"";""Autóctone"";"""";"""";"""";75;""P.Silveira J.D.Almeida E.Portela-Pereira A.Silva D.T.Holyoak A.Carapeto F.Clamote M.Porto P.V.Araújo"""
421	"Fabaceae;""Genista triacanthos"";""Autóctone"";"""";"""";"""";298;""P.V.Araújo A.Silva"""
422	"Fabaceae;""Hymenocarpos lotoides"";""Autóctone"";"""";"""";"""";268;""A.Silva"""
423	"Fabaceae;""Lathyrus angulatus"";""Autóctone"";"""";"""";"""";252;""F.Clamote"""
424	"Fabaceae;""Lathyrus aphaca"";""Autóctone"";"""";"""";"""";79;""A.Silva"""
425	"Fabaceae;""Lathyrus cicera"";""Autóctone"";"""";"""";"""";133;""A.Silva"""
426	"Fabaceae;""Lathyrus latifolius"";""Autóctone"";"""";"""";"""";56;""J.Jansen"""
427	"Fabaceae;""Lathyrus niger"";""Autóctone"";"""";"""";"""";25;""P.V.Araújo A.Silva"""
428	"Fabaceae;""Lathyrus sphaericus"";""Autóctone"";"""";"""";"""";96;""J.D.Almeida A.Silva"""
429	"Fabaceae;""Lathyrus sylvestris"";""Autóctone"";"""";"""";"""";47;""F.Clamote"""
430	"Fabaceae;""Lotus castellanus"";""Autóctone"";"""";"""";"""";67;""J.Jansen"""
431	"Fabaceae;""Lotus corniculatus subsp. carpetanus"";""Autóctone"";""Endémica da Península Ibérica"";"""";"""";127;""F.Clamote A.Silva P.Silveira D.T.Holyoak J.D.Almeida"""
432	"Fabaceae;""Lotus corniculatus"";"""";"""";"""";"""";216;""F.Clamote A.Silva"""
433	"Fabaceae;""Lotus glaber"";""Autóctone"";"""";"""";"""";16;""F.Clamote"""

434	"Fabaceae;""Lotus pedunculatus"";""Autóctone"";"""";"""";"""";368;""E.Portela-Pereira P.Silveira F.Clamote P.V.Araújo A.Silva J.T.Tavares J.Jansen J.D.Almeida D.T.Holyoak"""
435	"Fabaceae;""Lupinus angustifolius"";""Autóctone"";"""";"""";"""";284;""F.Clamote J.D.Almeida"""
436	"Fabaceae;""Lupinus gredensis"";""Autóctone"";""Endémica da Península Ibérica"";"""";"""";102;""J.Jansen P.V.Araújo A.Silva A.Carapeto F.Clamote J.D.Almeida"""
437	"Fabaceae;""Lupinus luteus"";""Autóctone"";"""";"""";"""";188;""P.V.Araújo J.D.Almeida"""
438	"Fabaceae;""Medicago arabica"";""Autóctone"";"""";"""";"""";112;""F.Clamote"""
439	"Fabaceae;""Medicago lupulina"";""Autóctone"";"""";"""";"""";131;""J.D.Almeida A.Silva"""
440	"Fabaceae;""Melilotus albus"";""Autóctone"";"""";"""";"""";40;""A.Silva"""
441	"Fabaceae;""Ornithopus compressus"";""Autóctone"";"""";"""";"""";482;""F.Clamote A.Silva D.T.Holyoak J.D.Almeida E.Portela-Pereira J.Jansen"""
442	"Fabaceae;""Ornithopus perpusillus"";""Autóctone"";"""";"""";"""";174;""D.T.Holyoak F.Clamote P.V.Araújo J.Jansen A.Silva"""
443	"Fabaceae;""Ornithopus pinnatus"";""Autóctone"";"""";"""";"""";231;""A.Silva F.Clamote"""
444	"Fabaceae;""Ornithopus sativus"";"""";"""";"""";"""";123;""F.Clamote"""
445	"Fabaceae;""Pisum sativum subsp. elatius"";""Autóctone"";"""";"""";"""";24;""J.Jansen"""
446	"Fabaceae;""Pisum sativum"";"""";"""";"""";"""";54;""A.Silva"""
447	"Fabaceae;""Pterospartum tridentatum subsp. cantabricum"";""Autóctone"";"""";"""";"""";99;""A.Silva D.T.Holyoak"""
448	"Fabaceae;""Pterospartum tridentatum"";"""";"""";"""";"""";464;""A.Carapeto F.Clamote P.Beja A.Silva M.Porto"""
449	"Fabaceae;""Retama sphaerocarpa"";""Autóctone"";"""";"""";"""";126;""A.Carapeto"""
450	"Fabaceae;""Robinia pseudoacacia"";""Introduzida"";""Exótica"";"""";"""";56;""E.Portela-Pereira J.D.Almeida"""
451	"Fabaceae;""Trifolium angustifolium"";""Autóctone"";"""";"""";"""";514;""F.Clamote A.Silva E.Portela-Pereira P.V.Araújo"""
452	"Fabaceae;""Trifolium arvense"";""Inexistente"";"""";"""";"""";424;""F.Clamote A.Silva D.T.Holyoak J.D.Almeida"""
453	"Fabaceae;""Trifolium campestre"";""Autóctone"";"""";"""";""LC"";387;""P.V.Araújo"""
454	"Fabaceae;""Trifolium dubium"";""Autóctone"";"""";"""";"""";222;""P.V.Araújo D.T.Holyoak F.Clamote J.D.Almeida A.Silva"""
455	"Fabaceae;""Trifolium glomeratum"";""Autóctone"";"""";"""";"""";245;""J.D.Almeida"""
456	"Fabaceae;""Trifolium incarnatum"";""Introduzida"";""Exótica"";"""";"""";40;""P.V.Araújo"""
457	"Fabaceae;""Trifolium ochroleucon"";""Autóctone"";"""";"""";"""";9;""J.D.Almeida"""
458	"Fabaceae;""Trifolium pratense subsp. pratense"";""Autóctone"";"""";"""";"""";216;""A.Silva F.Clamote J.D.Almeida"""
459	"Fabaceae;""Trifolium repens"";""Inexistente"";"""";"""";"""";498;""A.Silva F.Clamote D.T.Holyoak J.T.Tavares P.V.Araújo J.Jansen J.D.Almeida"""

460	"Fabaceae;""Trifolium repens var. repens"";""Autóctone"";"""";"""";"""";120;""J.D.Almeida"""
461	"Fabaceae;""Trifolium subterraneum subsp. subterraneum"";""Autóctone"";"""";"""";"""";26;""E.Portela-Pereira"""
462	"Fabaceae;""Trifolium subterraneum"";""Inexistente"";"""";"""";"""";214;""F.Clamote"""
463	"Fabaceae;""Trifolium vesiculosum"";""Autóctone"";"""";"""";"""";40;""E.Portela-Pereira"""
464	"Fabaceae;""Ulex europaeus subsp. latebracteatus"";""Autóctone"";""Endémica da Península Ibérica"";"""";"""";124;""C.Vila-Viçosa"""
465	"Fabaceae;""Ulex europaeus"";"""";"""";"""";"""";209;""A.Silva"""
466	"Fabaceae;""Ulex minor"";""Autóctone"";"""";"""";"""";321;""M.Porto J.D.Almeida A.Silva J.T.Tavares"""
467	"Fabaceae;""Vicia angustifolia"";""Autóctone"";"""";"""";"""";261;""E.Marabuto A.Silva F.Clamote D.T.Holyoak"""
468	"Fabaceae;""Vicia cordata"";""Autóctone"";"""";"""";"""";17;""E.Portela-Pereira"""
469	"Fabaceae;""Vicia dasycarpa"";""Autóctone"";"""";"""";"""";72;""F.Clamote"""
470	"Fabaceae;""Vicia disperma"";""Autóctone"";"""";"""";"""";232;""F.Clamote D.T.Holyoak A.Silva"""
471	"Fabaceae;""Vicia hirsuta"";""Autóctone"";"""";"""";"""";37;""A.Silva"""
472	"Fabaceae;""Vicia lutea subsp. lutea"";""Autóctone"";"""";"""";"""";293;""P.V.Araújo F.Clamote"""
473	"Fabaceae;""Vicia lutea"";"""";"""";"""";"""";469;""A.Silva"""
474	"Fabaceae;""Vicia sativa subsp. sativa"";""Autóctone"";"""";"""";"""";337;""A.Silva J.D.Almeida"""
475	"Fabaceae;""Vicia tenuifolia"";""Autóctone"";"""";"""";"""";12;""A.Silva"""
476	"Fagaceae;""Castanea sativa"";""Introduzida"";""Exótica"";"""";"""";296;""F.Clamote A.Carapeto A.Silva J.Pessoa J.Jansen E.Portela-Pereira J.D.Almeida J.T.Tavares P.Pereira"""
477	"Fagaceae;""Quercus faginea"";"""";"""";"""";"""";416;""A.Silva"""
478	"Fagaceae;""Quercus pyrenaica"";""Autóctone"";"""";"""";"""";314;""F.Clamote E.Portela-Pereira A.Silva A.Carapeto J.D.Almeida P.V.Araújo P.Ministro J.Jansen"""
479	"Fagaceae;""Quercus robur"";""Autóctone"";"""";"""";"""";256;""A.Silva E.Portela-Pereira A.Carapeto J.T.Tavares C.Vila-Viçosa P.Pereira"""
480	"Fagaceae;""Quercus rotundifolia"";""Autóctone"";"""";"""";"""";488;""A.Silva E.Portela-Pereira D.T.Holyoak F.Clamote J.T.Tavares"""
481	"Fagaceae;""Quercus rubra"";""Introduzida"";""Exótica"";"""";"""";22;""A.Carapeto"""
482	"Fagaceae;""Quercus suber"";""Autóctone"";"""";"""";"""";631;""A.Silva J.D.Almeida A.Carapeto"""
483	"Gentianaceae;""Centaurium erythraea-(1)"";"""";"""";"""";"""";224;""P.V.Araújo A.Silva"""
484	"Gentianaceae;""Cicendia filiformis"";""Autóctone"";"""";"""";"""";86;""J.Jansen"""
485	"Gentianaceae;""Gentiana lutea subsp. lutea"";""Autóctone"";"""";""Anexo V"";""CR"";2;""F.Clamote J.Jansen A.Silva C.Meireles P.V.Araújo M.Porto A.Matos J.D.Almeida D.T.Holyoak"""

486	"Gentianaceae;""Gentiana pneumonanthe"";""Autóctone"";"""";"""";"""";55;""A.J.Pereira F.Clamote A.Silva M.Porto P.V.Araújo J.T.Tavares P.Beja R.Lima P.Guillaumeau J.D.Almeida P.Ministro"""
487	"Geraniaceae;""Erodium cicutarium"";"""";"""";"""";"""";433;""A.Silva D.T.Holyoak M.Porto F.Clamote"""
488	"Geraniaceae;""Erodium moschatum"";""Autóctone"";"""";"""";"""";393;""J.T.Tavares P.Beja"""
489	"Geraniaceae;""Geranium columbinum"";""Autóctone"";"""";"""";"""";150;""A.Silva"""
490	"Geraniaceae;""Geranium dissectum"";""Autóctone"";"""";"""";"""";316;""F.Clamote"""
491	"Geraniaceae;""Geranium lucidum"";""Autóctone"";"""";"""";"""";313;""A.Silva F.Clamote E.Portela-Pereira J.T.Tavares P.V.Araújo A.Carapeto"""
492	"Geraniaceae;""Geranium molle"";""Autóctone"";"""";"""";"""";545;""A.Silva P.Beja F.Clamote J.D.Almeida J.Jansen"""
493	"Geraniaceae;""Geranium purpureum"";""Autóctone"";"""";"""";"""";517;""A.Silva F.Clamote J.Jansen E.Portela-Pereira P.Pereira P.V.Araújo"""
494	"Geraniaceae;""Geranium pyrenaicum subsp. lusitanicum"";""Autóctone"";""Endémica da Península Ibérica"";"""";"""";70;""F.Clamote"""
495	"Geraniaceae;""Geranium robertianum"";""Autóctone"";"""";"""";"""";134;""A.Silva E.Portela-Pereira F.Clamote J.Jansen P.V.Araújo"""
496	"Geraniaceae;""Geranium rotundifolium"";""Autóctone"";"""";"""";"""";303;""E.Portela-Pereira A.Silva F.Clamote J.D.Almeida"""
497	"Haloragaceae;""Myriophyllum alterniflorum"";""Autóctone"";"""";"""";"""";43;""U.Schwarzer"""
498	"Hypericaceae;""Hypericum androsaemum"";""Autóctone"";"""";"""";"""";94;""A.Silva E.Portela-Pereira P.Pereira M.Porto J.T.Tavares"""
499	"Hypericaceae;""Hypericum elodes"";""Autóctone"";"""";"""";"""";100;""J.Jansen A.Silva"""
500	"Hypericaceae;""Hypericum humifusum"";""Autóctone"";"""";"""";"""";278;""J.Jansen P.V.Araújo A.Silva P.Silveira D.T.Holyoak J.T.Tavares F.Clamote"""
501	"Hypericaceae;""Hypericum linariifolium var. parviflorum"";""Autóctone"";""Endémica da Península Ibérica"";"""";"""";38;""J.D.Almeida"""
502	"Hypericaceae;""Hypericum linariifolium"";""Inexistente"";"""";"""";"""";298;""A.Silva D.T.Holyoak M.Porto P.V.Araújo J.Jansen J.D.Almeida P.Silveira F.Clamote"""
503	"Hypericaceae;""Hypericum perforatum"";""Inexistente"";"""";"""";"""";514;""J.Jansen J.D.Almeida F.Clamote"""
504	"Hypericaceae;""Hypericum pulchrum"";""Autóctone"";"""";"""";"""";48;""A.Silva"""
505	"Hypericaceae;""Hypericum undulatum"";""Autóctone"";"""";"""";"""";312;""F.Clamote A.Silva P.Silveira E.Portela-Pereira J.Jansen A.Carapeto P.Ministro"""
506	"Iridaceae;""Crocus carpetanus"";""Autóctone"";""Endémica da Península Ibérica"";"""";"""";41;""A.Silva D.T.Holyoak A.Carapeto J.T.Tavares"""
507	"Iridaceae;""Crocus serotinus"";"""";"""";"""";"""";171;""A.Silva J.T.Tavares"""
508	"Iridaceae;""Gladiolus illyricus"";"""";"""";"""";"""";259;""A.Silva P.V.Araújo J.Jansen"""

509	"Iridaceae;""Romulea bulbocodium"";""Autóctone"";"""";"""";"""";225;""P.Silveira A.Silva J.Jansen"""
510	"Juncaceae;""Juncus acutiflorus"";""Autóctone"";"""";"""";"""";146;""A.Silva J.Jansen P.Silveira"""
511	"Juncaceae;""Juncus acutus"";""Autóctone"";"""";"""";"""";106;""E.Portela-Pereira"""
512	"Juncaceae;""Juncus articulatus subsp. articulatus"";""Autóctone"";"""";"""";"""";109;""P.Silveira J.Jansen"""
513	"Juncaceae;""Juncus bufonius"";""Autóctone"";"""";"""";"""";287;""A.Silva J.Jansen E.Portela-Pereira"""
514	"Juncaceae;""Juncus bulbosus"";""Autóctone"";"""";"""";"""";109;""F.Clamote J.Jansen A.Silva D.T.Holyoak P.Guillaumeau"""
515	"Juncaceae;""Juncus capitatus"";""Autóctone"";"""";"""";"""";177;""J.Jansen"""
516	"Juncaceae;""Juncus conglomeratus"";""Autóctone"";"""";"""";"""";50;""E.Portela-Pereira"""
517	"Juncaceae;""Juncus effusus subsp. effusus"";""Autóctone"";"""";"""";"""";245;""A.Silva F.Clamote P.V.Araújo J.Jansen P.Silveira"""
518	"Juncaceae;""Juncus pygmaeus"";""Autóctone"";"""";"""";"""";101;""F.Clamote"""
519	"Juncaceae;""Juncus squarrosus"";""Autóctone"";"""";"""";"""";57;""A.Carapeto D.T.Holyoak P.Pereira J.Jansen A.Silva E.Portela-Pereira M.Porto F.Clamote P.V.Araújo J.D.Almeida"""
520	"Juncaceae;""Juncus tenageia subsp. perpusillus"";""Autóctone"";"""";"""";"""";1;""A.Silva"""
521	"Juncaceae;""Juncus tenageia"";"""";"""";"""";"""";94;""F.Clamote D.T.Holyoak A.Carapeto"""
522	"Juncaceae;""Luzula caespitosa"";""Autóctone"";""Endémica da Península Ibérica"";"""";""NT"";2;""J.Jansen A.Silva M.Porto D.T.Holyoak C.Meireles P.Pereira S.Ribeiro A.Matos"""
523	"Juncaceae;""Luzula campestris"";""Autóctone"";"""";"""";"""";81;""A.Silva J.D.Almeida"""
524	"Juncaceae;""Luzula forsteri"";""Autóctone"";"""";"""";"""";139;""A.Silva F.Clamote J.D.Almeida J.Jansen"""
525	"Juncaceae;""Luzula lactea"";""Autóctone"";""Endémica da Península Ibérica"";"""";"""";43;""F.Clamote A.Silva P.Pereira A.Carapeto P.V.Araújo A.J.Pereira D.T.Holyoak"""
526	"Juncaceae;""Luzula multiflora subsp. multiflora"";""Autóctone"";"""";"""";"""";40;""A.Silva E.Portela-Pereira"""
527	"Juncaceae;""Luzula sylvatica subsp. henriquesii"";""Autóctone"";""Endémica da Península Ibérica"";"""";"""";76;""E.Portela-Pereira A.Silva A.Carapeto F.Clamote J.Jansen J.D.Almeida"""
528	"Lamiaceae;""Acinos alpinus"";""Autóctone"";"""";"""";"""";17;""P.V.Araújo A.Silva J.D.Almeida J.Jansen"""
529	"Lamiaceae;""Ajuga pyramidalis subsp. meonantha"";""Autóctone"";"""";"""";"""";28;""F.Clamote A.Silva J.Jansen"""
530	"Lamiaceae;""Ajuga pyramidalis"";"""";"""";"""";"""";69;""F.Clamote A.Silva"""

531	"Lamiaceae;""Calamintha nepeta subsp. nepeta"";""Autóctone"";"""";"""";"""";380;""A.Silva M.Porto J.D.Almeida"""
532	"Lamiaceae;""Clinopodium vulgare"";""Autóctone"";"""";"""";"""";368;""F.Clamote A.Silva J.D.Almeida E.Portela-Pereira P.V.Araújo J.Jansen A.Carapeto"""
533	"Lamiaceae;""Glechoma hederacea"";""Autóctone"";"""";"""";"""";29;""A.Silva M.Porto P.Beja"""
534	"Lamiaceae;""Lamium amplexicaule"";""Autóctone"";"""";"""";"""";299;""A.Silva F.Clamote P.Beja"""
535	"Lamiaceae;""Lamium bifidum"";""Autóctone"";"""";"""";"""";18;""M.Porto A.Silva"""
536	"Lamiaceae;""Lamium hybridum"";""Autóctone"";"""";"""";"""";34;""P.Beja"""
537	"Lamiaceae;""Lamium maculatum"";""Autóctone"";"""";"""";"""";222;""J.D.Almeida D.T.Holyoak E.Portela-Pereira A.Silva M.Porto F.Clamote J.T.Tavares J.Jansen P.V.Araújo P.Beja"""
538	"Lamiaceae;""Lamium purpureum"";""Autóctone"";"""";"""";"""";288;""F.Clamote J.D.Almeida A.Silva M.Porto P.Beja"""
539	"Lamiaceae;""Lavandula pedunculata subsp. pedunculata"";""Autóctone"";"""";"""";"""";123;""J.Jansen J.D.Almeida"""
540	"Lamiaceae;""Lavandula stoechas"";"""";"""";"""";"""";389;""A.Silva F.Clamote P.Silveira M.Porto"""
541	"Lamiaceae;""Lycopus europaeus"";""Autóctone"";"""";"""";"""";297;""F.Clamote A.Silva"""
542	"Lamiaceae;""Melissa officinalis"";""Autóctone"";"""";"""";"""";98;""P.Pereira"""
543	"Lamiaceae;""Melittis melissophyllum"";""Autóctone"";"""";"""";"""";41;""A.Silva A.Carapeto"""
544	"Lamiaceae;""Mentha aquatica"";""Autóctone"";"""";"""";"""";112;""P.Ministro"""
545	"Lamiaceae;""Mentha pulegium"";""Autóctone"";"""";"""";"""";392;""A.Silva F.Clamote P.Silveira"""
546	"Lamiaceae;""Mentha suaveolens"";""Autóctone"";"""";"""";"""";598;""F.Clamote A.Silva D.T.Holyoak A.Carapeto P.Silveira E.Portela-Pereira R.Faria J.D.Almeida A.Clemente"""
547	"Lamiaceae;""Origanum vulgare subsp. virens"";""Autóctone"";"""";"""";"""";287;""A.Silva F.Clamote J.T.Tavares J.D.Almeida J.Jansen"""
548	"Lamiaceae;""Prunella grandiflora"";""Autóctone"";"""";"""";"""";48;""A.Silva E.Portela-Pereira P.V.Araújo"""
549	"Lamiaceae;""Prunella vulgaris"";""Autóctone"";"""";"""";"""";404;""E.Portela-Pereira F.Clamote A.Silva J.T.Tavares P.V.Araújo P.Silveira"""
550	"Lamiaceae;""Salvia verbenaca"";""Autóctone"";"""";"""";"""";174;""A.Silva"""
551	"Lamiaceae;""Scutellaria minor"";""Autóctone"";"""";"""";"""";108;""J.Jansen E.Portela-Pereira A.Silva"""
552	"Lamiaceae;""Stachys arvensis"";""Autóctone"";"""";"""";"""";428;""A.Silva P.Beja F.Clamote"""
553	"Lamiaceae;""Teucrium salviastrum"";""Endémica"";""Endémica de Portugal continental"";""Anexo V"";""LC"";30;""D.T.Holyoak A.Silva J.Jansen F.Clamote J.D.Almeida E.Marabuto M.Porto E.Portela-

	Pereira P.Pereira P.V.Araújo C.Meireles A.Carapeto J.T.Tavares C.Aguiar A.J.Pereira P.Silveira P.Ministro"""
554	"Lamiaceae;""Teucrium scorodonia"";""Autóctone"";"""";"""";"""";429;""F.Clamote A.Silva M.Porto J.T.Tavares J.D.Almeida A.Carapeto E.Portela-Pereira P.V.Araújo"""
555	"Lamiaceae;""Thymus mastichina subsp. mastichina"";""Autóctone"";""Endémica da Península Ibérica"";"""";"""";119;""P.V.Araújo E.Portela-Pereira J.D.Almeida P.Ministro"""
556	"Lamiaceae;""Thymus mastichina"";"""";"""";"""";"""";296;""F.Clamote A.Silva"""
557	"Lauraceae;""Laurus nobilis"";""Autóctone"";"""";"""";"""";237;""A.Silva J.Jansen"""
558	"Liliaceae;""Erythronium dens-canis"";""Autóctone"";"""";"""";"""";25;""A.Silva H.Engels J.Jansen"""
559	"Liliaceae;""Fritillaria nervosa subsp. nervosa"";""Autóctone"";"""";"""";""NT"";20;""E.Portela-Pereira A.Carapeto J.Jansen A.Matos P.V.Araújo J.D.Almeida P.Lemos A.Silva S.Ribeiro F.Clamote P.Ministro D.T.Holyoak A.J.Pereira P.Pereira"""
560	"Liliaceae;""Gagea foliosa subsp. ellyptica"";""Autóctone"";"""";"""";"""";36;""S.Ribeiro"""
561	"Liliaceae;""Gagea soleirolii"";""Autóctone"";"""";"""";"""";87;""A.Carapeto E.Portela-Pereira F.Clamote A.Silva M.Porto J.Jansen D.T.Holyoak S.Ribeiro"""
562	"Liliaceae;""Lilium martagon"";""Autóctone"";"""";"""";""VU"";18;""A.Silva AOSP J.D.Almeida"""
563	"Linaceae;""Linum bienne"";""Autóctone"";"""";"""";"""";400;""A.Silva"""
564	"Linaceae;""Linum trigynum subsp. trigynum"";""Autóctone"";"""";"""";"""";59;""P.V.Araújo"""
565	"Linaceae;""Radiola linoides"";""Autóctone"";"""";"""";"""";127;""A.Silva"""
566	"Lycopodiaceae;""Lycopodiella inundata"";""Autóctone"";"""";""Anexo V"";""EN"";9;""A.Silva F.Covelo"""
567	"Lycopodiaceae;""Lycopodium clavatum"";""Autóctone"";"""";""Anexo V"";""CR"";1;""A.Silva P.V.Araújo A.Matos"""
568	"Lythraceae;""Lythrum portula"";""Autóctone"";"""";"""";"""";113;""A.Silva P.Silveira F.Clamote"""
569	"Lythraceae;""Lythrum salicaria"";""Autóctone"";"""";"""";"""";402;""E.Portela-Pereira F.Clamote A.Clemente"""
570	"Malvaceae;""Malva neglecta"";""Autóctone"";"""";"""";"""";54;""A.Silva"""
571	"Malvaceae;""Malva sylvestris"";""Autóctone"";"""";"""";"""";104;""F.Clamote"""
572	"Malvaceae;""Malva tournefortiana"";""Autóctone"";"""";"""";"""";198;""A.Silva F.Clamote E.Portela-Pereira J.D.Almeida E.Marabuto P.V.Araújo J.Jansen"""
573	"Melanthiaceae;""Veratrum album"";""Autóctone"";"""";"""";""CR"";3;""A.Silva J.Jansen P.V.Araújo A.Matos AOSP"""
574	"Menyanthaceae;""Menyanthes trifoliata"";""Autóctone"";"""";"""";""EN"";3;""J.Jansen A.Silva A.Matos C.Meireles"""
575	"Moraceae;""Ficus carica"";""De espontaneidade incerta"";"""";"""";"""";273;""E.Portela-Pereira"""

576	"Myrtaceae;""Eucalyptus globulus"";""Introduzida"";""Exótica"";"""";"""";343;""A.Silva P.Pereira"""
577	"Oleaceae;""Fraxinus angustifolia subsp. angustifolia"";""Autóctone"";"""";"""";"""";605;""F.Clamote A.Silva J.T.Tavares J.Jansen E.Portela-Pereira"""
578	"Oleaceae;""Olea europaea var. europaea"";""Introduzida"";""Exótica"";"""";"""";236;""A.Silva"""
579	"Oleaceae;""Phillyrea angustifolia"";""Autóctone"";"""";"""";"""";404;""A.Silva P.Silveira P.Beja M.Porto"""
580	"Onagraceae;""Circaea lutetiana subsp. lutetiana"";""Autóctone"";"""";"""";"""";40;""A.Silva"""
581	"Onagraceae;""Epilobium lanceolatum"";""Autóctone"";"""";"""";"""";20;""A.Silva E.Portela-Pereira"""
582	"Onagraceae;""Epilobium obscurum"";""Autóctone"";"""";"""";"""";52;""E.Portela-Pereira A.Silva J.Jansen M.Porto D.T.Holyoak P.Silveira"""
583	"Onagraceae;""Epilobium palustre"";""Autóctone"";"""";"""";"""";3;""A.Silva J.Jansen"""
584	"Onagraceae;""Epilobium tetragonum subsp. tetragonum"";""Autóctone"";"""";"""";"""";12;""P.Ministro"""
585	"Orchidaceae;""Dactylorhiza maculata"";""Autóctone"";"""";"""";"""";77;""J.Jansen P.V.Araújo A.Amado A.Silva E.Portela-Pereira AOSP F.Clamote M.Porto"""
586	"Orchidaceae;""Epipactis fageticola"";""Autóctone"";"""";"""";""EN"";6;""J.Pessoa P.V.Araújo AOSP"""
587	"Orchidaceae;""Epipactis helleborine subsp. helleborine"";""Autóctone"";"""";"""";"""";19;""A.Silva AOSP P.V.Araújo J.Pessoa"""
588	"Orchidaceae;""Neottia nidus-avis"";""Autóctone"";"""";"""";""VU"";9;""J.Pessoa"""
589	"Orchidaceae;""Orchis langei"";""Autóctone"";"""";"""";"""";42;""A.Silva P.V.Araújo"""
590	"Orchidaceae;""Orchis mascula"";""Autóctone"";"""";"""";"""";79;""A.Silva P.Silveira"""
591	"Orchidaceae;""Serapias cordigera subsp. cordigera"";""Autóctone"";"""";"""";"""";94;""J.Jansen E.Marabuto A.Silva"""
592	"Orchidaceae;""Serapias lingua"";""Autóctone"";"""";"""";"""";227;""A.Silva"""
593	"Orobanchaceae;""Bartsia trixago"";""Autóctone"";"""";"""";"""";352;""F.Clamote J.Jansen"""
594	"Orobanchaceae;""Orobanche gracilis"";""Autóctone"";"""";"""";"""";78;""E.Portela-Pereira A.Silva"""
595	"Orobanchaceae;""Orobanche minor"";""Autóctone"";"""";"""";"""";79;""P.V.Araújo"""
596	"Orobanchaceae;""Orobanche rapum-genistae"";""Autóctone"";"""";"""";"""";126;""F.Clamote P.V.Araújo A.Silva D.T.Holyoak J.Jansen"""
597	"Orobanchaceae;""Parentucellia latifolia"";""Autóctone"";"""";"""";"""";146;""A.Silva"""
598	"Orobanchaceae;""Parentucellia viscosa"";""Autóctone"";"""";"""";"""";338;""F.Clamote"""

599	"Orobanchaceae;""Pedicularis sylvatica subsp. lusitanica"";""Autóctone"";"""";"""";"""";118;""F.Clamote A.Silva M.Porto J.Jansen A.Carapeto D.T.Holyoak E.Portela-Pereira S.Ribeiro P.V.Araújo"""
600	"Orobanchaceae;""Rhinanthus minor"";""Autóctone"";"""";"""";"""";65;""A.Silva E.Marabuto"""
601	"Osmundaceae;""Osmunda regalis"";""Autóctone"";"""";"""";"""";301;""A.Silva E.Portela-Pereira A.Carapeto M.Porto J.T.Tavares F.Clamote"""
602	"Oxalidaceae;""Oxalis corniculata"";""Autóctone"";"""";"""";"""";222;""P.Beja"""
603	"Oxalidaceae;""Oxalis pes-caprae"";""Introduzida"";""Exótica"";"""";"""";379;""M.Porto"""
604	"Paeoniaceae;""Paeonia broteri"";""Autóctone"";"""";"""";""LC"";104;""A.Silva A.Carapeto J.D.Almeida J.Pessoa"""
605	"Paeoniaceae;""Paeonia officinalis subsp. microcarpa"";""Autóctone"";"""";"""";"""";12;""J.D.Almeida A.Silva"""
606	"Papaveraceae;""Ceratocapnos claviculata subsp. picta"";""Endémica"";""Endémica de Portugal continental"";"""";""LC"";22;""P.Pereira A.Silva"""
607	"Papaveraceae;""Ceratocapnos claviculata subsp. claviculata"";""Autóctone"";"""";"""";"""";98;""A.Silva E.Portela-Pereira"""
608	"Papaveraceae;""Ceratocapnos claviculata"";"""";"""";"""";"""";146;""A.Carapeto"""
609	"Papaveraceae;""Chelidonium majus"";""Autóctone"";"""";"""";"""";310;""F.Clamote A.Silva E.Portela-Pereira J.Jansen P.Beja J.T.Tavares"""
610	"Papaveraceae;""Fumaria bastardii"";""Autóctone"";"""";"""";"""";22;""A.Silva"""
611	"Papaveraceae;""Fumaria muralis"";""Autóctone"";"""";"""";"""";154;""A.Silva E.Portela-Pereira"""
612	"Papaveraceae;""Papaver argemone"";""Autóctone"";"""";"""";"""";38;""A.Silva"""
613	"Papaveraceae;""Papaver dubium"";""Autóctone"";"""";"""";"""";159;""A.Silva F.Clamote"""
614	"Papaveraceae;""Papaver rhoeas"";"""";"""";"""";"""";375;""A.Silva"""
615	"Phytolaccaceae;""Phytolacca americana"";""Introduzida"";""Exótica"";"""";"""";272;""A.Silva E.Portela-Pereira F.Clamote"""
616	"Pinaceae;""Pinus pinaster"";""Autóctone"";"""";"""";"""";581;""A.Silva F.Clamote E.Portela-Pereira D.T.Holyoak P.Pereira"""
617	"Pinaceae;""Pinus sylvestris"";""Autóctone"";"""";"""";""EN"";30;""E.Portela-Pereira J.T.Tavares D.Frade A.Carapeto D.T.Holyoak"""
618	"Plantaginaceae;""Anarrhinum bellidifolium"";""Autóctone"";"""";"""";"""";516;""F.Clamote A.Silva M.Porto P.V.Araújo J.Jansen E.Marabuto J.D.Almeida E.Portela-Pereira J.T.Tavares"""
619	"Plantaginaceae;""Antirrhinum graniticum"";""Autóctone"";""Endémica da Península Ibérica"";"""";"""";88;""F.Clamote A.Silva J.Jansen"""
620	"Plantaginaceae;""Antirrhinum meonanthum"";""Autóctone"";""Endémica da Península Ibérica"";"""";"""";18;""A.Silva E.Portela-Pereira J.D.Almeida P.V.Araújo E.Marabuto"""

621	"Plantaginaceae;""Cymbalaria muralis subsp. muralis"";""Introduzida"";""Endémica da Península Ibérica"";"""";"""";168;""A.Silva J.T.Tavares"""
622	"Plantaginaceae;""Digitalis purpurea subsp. purpurea"";""Autóctone"";"""";"""";"""";399;""D.T.Holyoak A.Carapeto M.Porto P.V.Araújo F.Clamote J.Jansen A.Silva J.D.Almeida E.Marabuto"""
623	"Plantaginaceae;""Digitalis purpurea"";"""";"""";"""";"""";589;""J.T.Tavares F.Clamote A.Silva J.Jansen J.D.Almeida E.Portela-Pereira"""
624	"Plantaginaceae;""Digitalis thapsi"";""Autóctone"";""Endémica da Península Ibérica"";"""";"""";184;""A.Silva D.T.Holyoak F.Clamote M.Porto J.Jansen P.V.Araújo"""
625	"Plantaginaceae;""Linaria diffusa"";""Endémica"";""Endémica de Portugal continental"";"""";""DD"";8;""A.Silva"""
626	"Plantaginaceae;""Linaria elegans"";""Autóctone"";""Endémica da Península Ibérica"";"""";"""";63;""J.T.Tavares M.Porto P.V.Araújo F.Clamote A.J.Pereira A.Silva S.Ribeiro A.Carapeto F.Covelo P.Beja J.Jansen"""
627	"Plantaginaceae;""Linaria saxatilis"";""Autóctone"";""Endémica da Península Ibérica"";"""";"""";160;""A.Silva J.Jansen J.D.Almeida D.T.Holyoak E.Portela-Pereira P.V.Araújo P.Pereira F.Clamote M.Porto A.J.Pereira P.Silveira"""
628	"Plantaginaceae;""Linaria spartea"";""Autóctone"";"""";"""";"""";320;""P.V.Araújo"""
629	"Plantaginaceae;""Linaria triornithophora"";""Autóctone"";""Endémica da Península Ibérica"";"""";"""";182;""F.Clamote E.Portela-Pereira P.V.Araújo A.Silva J.D.Almeida"""
630	"Plantaginaceae;""Misopates orontium"";""Autóctone"";"""";"""";"""";380;""P.V.Araújo A.Silva E.Portela-Pereira"""
631	"Plantaginaceae;""Plantago alpina"";""Autóctone"";"""";"""";""NT"";1;""J.Jansen A.Silva F.Clamote P.Pereira A.Matos S.Ribeiro"""
632	"Plantaginaceae;""Plantago bellardii"";""Autóctone"";"""";"""";"""";146;""A.Silva"""
633	"Plantaginaceae;""Plantago coronopus"";""Autóctone"";"""";"""";"""";621;""M.Porto A.Silva D.T.Holyoak J.D.Almeida"""
634	"Plantaginaceae;""Plantago holosteum"";""Autóctone"";"""";"""";"""";40;""A.Silva P.Pereira P.V.Araújo J.D.Almeida P.Ministro J.Jansen"""
635	"Plantaginaceae;""Plantago lanceolata"";""Autóctone"";"""";"""";"""";545;""A.Silva F.Clamote E.Portela-Pereira J.D.Almeida D.T.Holyoak P.V.Araújo J.Jansen"""
636	"Plantaginaceae;""Plantago major subsp. intermedia"";""Autóctone"";"""";"""";"""";299;""A.Silva F.Clamote J.D.Almeida"""
637	"Plantaginaceae;""Sibthorpia europaea"";""Autóctone"";"""";"""";"""";149;""A.Silva M.Porto J.T.Tavares J.Jansen P.V.Araújo F.Clamote"""
638	"Plantaginaceae;""Veronica arvensis"";""Autóctone"";"""";"""";"""";250;""J.D.Almeida D.T.Holyoak F.Clamote P.Beja J.Jansen"""
639	"Plantaginaceae;""Veronica hederifolia"";""Autóctone"";"""";"""";"""";72;""A.Silva"""

640	"Plantaginaceae;""Veronica micrantha"";""Autóctone"";""Endémica da Península Ibérica"";""Anexo IIAnexo IV"";""NT"";41;""A.Silva J.Jansen E.Portela-Pereira"""
641	"Plantaginaceae;""Veronica nevadensis"";""Autóctone"";""Endémica da Península Ibérica"";"""";""VU"";3;""J.Jansen P.Pereira F.Clamote P.V.Araújo A.Silva D.T.Holyoak"""
642	"Plantaginaceae;""Veronica officinalis"";""Autóctone"";"""";"""";"""";87;""A.Silva F.Clamote J.D.Almeida E.Portela-Pereira P.V.Araújo"""
643	"Plantaginaceae;""Veronica persica"";""Autóctone"";"""";"""";"""";165;""A.Silva P.Beja"""
644	"Plantaginaceae;""Veronica scutellata"";""Autóctone"";"""";"""";"""";27;""A.Silva J.Jansen"""
645	"Plumbaginaceae;""Armeria beirana"";"""";"""";"""";"""";41;""S.Ribeiro"""
646	"Plumbaginaceae;""Armeria beirana subsp. beirana"";""Autóctone"";""Endémica da Península Ibérica"";"""";"""";33;""E.Portela-Pereira A.Silva D.T.Holyoak J.D.Almeida P.Silveira"""
647	"Plumbaginaceae;""Armeria sampaioi"";""Endémica"";""Endémica de Portugal continental"";""Anexo V"";""VU"";10;""J.Jansen P.Pereira C.Meireles A.Silva F.Clamote S.Ribeiro A.Carapeto M.Porto"""
648	"Plumbaginaceae;""Armeria transmontana"";""Autóctone"";""Endémica da Península Ibérica"";"""";"""";73;""E.Portela-Pereira A.Carapeto F.Clamote"""
649	"Poaceae;""Agrostis canina subsp. canina"";""Autóctone"";"""";"""";"""";2;""J.Jansen"""
650	"Poaceae;""Agrostis capillaris"";""Autóctone"";"""";"""";"""";11;""E.Portela-Pereira"""
651	"Poaceae;""Agrostis castellana"";""Autóctone"";"""";"""";"""";255;""P.Silveira D.T.Holyoak E.Portela-Pereira A.Carapeto P.Ministro J.Jansen A.Silva J.D.Almeida"""
652	"Poaceae;""Agrostis curtisii"";""Autóctone"";"""";"""";"""";186;""A.Silva P.Silveira J.D.Almeida"""
653	"Poaceae;""Agrostis hesperica"";""Autóctone"";"""";"""";"""";10;""E.Portela-Pereira"""
654	"Poaceae;""Agrostis pourretii"";""Autóctone"";"""";"""";"""";72;""A.Silva"""
655	"Poaceae;""Agrostis truncatula subsp. commista"";""Autóctone"";"""";"""";"""";60;""A.Silva E.Portela-Pereira"""
656	"Poaceae;""Agrostis truncatula subsp. truncatula"";""Autóctone"";"""";"""";"""";19;""P.Silveira A.Silva D.T.Holyoak"""
657	"Poaceae;""Agrostis truncatula"";"""";"""";"""";"""";144;""A.Silva P.Ministro J.Jansen A.Carapeto"""
658	"Poaceae;""Aira caryophyllea"";"""";"""";"""";"""";164;""D.T.Holyoak A.Silva"""
659	"Poaceae;""Aira praecox"";""Autóctone"";"""";"""";"""";60;""D.T.Holyoak J.Jansen A.Silva"""
660	"Poaceae;""Anthoxanthum amarum"";""Autóctone"";""Endémica da Península Ibérica"";"""";"""";40;""J.Jansen"""
661	"Poaceae;""Anthoxanthum aristatum subsp. aristatum"";""Autóctone"";"""";"""";"""";156;""A.Silva J.Jansen D.T.Holyoak"""
662	"Poaceae;""Anthoxanthum odoratum"";""Autóctone"";"""";"""";"""";87;""F.Clamote A.Silva J.Jansen"""

663	"Poaceae;""Antinoria agrostidea"";""Autóctone"";"""";"""";"""";56;""A.Silva D.T.Holyoak A.Carapeto P.V.Araújo J.Jansen"""
664	"Poaceae;""Arrhenatherum album"";"""";"""";"""";"""";94;""D.T.Holyoak"""
665	"Poaceae;""Arrhenatherum elatius subsp. elatius"";""Autóctone"";"""";"""";"""";14;""E.Portela-Pereira"""
666	"Poaceae;""Arrhenatherum elatius subsp. bulbosum"";""Autóctone"";"""";"""";"""";150;""J.D.Almeida A.Silva D.T.Holyoak E.Portela-Pereira"""
667	"Poaceae;""Arrhenatherum elatius"";"""";"""";"""";"""";225;""A.Silva E.Portela-Pereira J.Jansen"""
668	"Poaceae;""Avena barbata subsp. barbata"";""Autóctone"";"""";"""";"""";107;""E.Portela-Pereira"""
669	"Poaceae;""Avena barbata subsp. lusitanica"";""Autóctone"";"""";"""";"""";44;""E.Portela-Pereira"""
670	"Poaceae;""Avena barbata"";""Inexistente"";"""";"""";"""";477;""E.Portela-Pereira J.D.Almeida"""
671	"Poaceae;""Avena sativa subsp. macrantha"";""Introduzida"";""Exótica"";"""";"""";16;""J.Jansen"""
672	"Poaceae;""Avenula sulcata"";"""";"""";"""";"""";114;""A.Silva A.Carapeto J.Jansen P.Pereira"""
673	"Poaceae;""Avenula sulcata subsp. sulcata"";""Autóctone"";"""";"""";"""";24;""D.T.Holyoak E.Portela-Pereira"""
674	"Poaceae;""Brachypodium phoenicoides"";"""";"""";"""";"""";215;""A.Silva"""
675	"Poaceae;""Brachypodium sylvaticum"";""Autóctone"";"""";"""";"""";265;""E.Portela-Pereira J.Jansen A.Silva"""
676	"Poaceae;""Briza maxima"";""Autóctone"";"""";"""";"""";683;""F.Clamote M.Porto J.Jansen A.Silva E.Portela-Pereira A.Carapeto J.D.Almeida"""
677	"Poaceae;""Briza minor"";""Autóctone"";"""";"""";"""";320;""E.Portela-Pereira J.Jansen F.Clamote"""
678	"Poaceae;""Bromus diandrus"";""Autóctone"";"""";"""";"""";282;""A.Silva D.T.Holyoak J.Jansen"""
679	"Poaceae;""Bromus hordeaceus"";""Autóctone"";"""";"""";"""";313;""A.Silva D.T.Holyoak E.Portela-Pereira J.Jansen P.Beja"""
680	"Poaceae;""Bromus rigidus"";""Autóctone"";"""";"""";"""";62;""E.Portela-Pereira"""
681	"Poaceae;""Bromus sterilis"";""Autóctone"";"""";"""";"""";73;""F.Clamote"""
682	"Poaceae;""Bromus tectorum"";""Autóctone"";"""";"""";"""";95;""A.Silva D.T.Holyoak M.Porto"""
683	"Poaceae;""Cortaderia selloana"";""Introduzida"";""Exótica"";"""";"""";143;""A.Silva"""
684	"Poaceae;""Corynephorus canescens"";""Autóctone"";"""";"""";"""";108;""D.T.Holyoak A.Silva"""
685	"Poaceae;""Cynosurus echinatus"";""Autóctone"";"""";"""";"""";416;""E.Portela-Pereira F.Clamote A.Silva D.T.Holyoak M.Porto J.Jansen"""
686	"Poaceae;""Cynosurus effusus"";""Autóctone"";"""";"""";"""";55;""E.Portela-Pereira"""

687	"Poaceae;""Dactylis glomerata subsp. lusitanica"";""Autóctone"";"""";"""";"""";126;""E.Portela-Pereira A.Silva"""
688	"Poaceae;""Dactylis glomerata"";"""";"""";"""";"""";779;""A.Silva D.T.Holyoak F.Clamote J.Jansen"""
689	"Poaceae;""Danthonia decumbens"";""Autóctone"";"""";"""";"""";136;""J.Jansen A.Silva"""
690	"Poaceae;""Deschampsia flexuosa"";""Autóctone"";"""";"""";"""";18;""P.Pereira M.Porto A.Silva J.Jansen D.T.Holyoak E.Portela-Pereira"""
691	"Poaceae;""Deschampsia stricta"";""Endémica"";""Endémica de Portugal continental"";"""";"""";23;""M.Porto"""
692	"Poaceae;""Festuca ampla subsp. ampla"";""Autóctone"";"""";"""";"""";26;""J.Jansen E.Portela-Pereira"""
693	"Poaceae;""Festuca durandoi subsp. livida"";""Autóctone"";""Endémica da Península Ibérica"";"""";"""";15;""J.Jansen"""
694	"Poaceae;""Festuca elegans"";""Autóctone"";"""";""Anexo IIAnexo IV"";""LC"";44;""P.Pereira S.Ribeiro J.Jansen E.Portela-Pereira A.Silva A.Carapeto D.T.Holyoak"""
695	"Poaceae;""Festuca henriquesii"";""Endémica"";""Endémica de Portugal continental"";""Anexo IIAnexo IV"";""VU"";2;""A.Silva S.Ribeiro A.Carapeto J.Jansen D.T.Holyoak E.Portela-Pereira"""
696	"Poaceae;""Festuca indigesta"";""Autóctone"";"""";"""";"""";6;""A.Silva"""
697	"Poaceae;""Festuca nigrescens subsp. microphylla"";""Autóctone"";"""";"""";"""";17;""E.Portela-Pereira"""
698	"Poaceae;""Festuca paniculata subsp. multispiculata"";""Autóctone"";"""";"""";"""";27;""A.Silva A.Carapeto"""
699	"Poaceae;""Festuca pseudotrichophylla"";""Autóctone"";"""";"""";"""";4;""E.Portela-Pereira"""
700	"Poaceae;""Festuca rivularis"";""Autóctone"";"""";"""";"""";9;""J.Jansen A.Silva"""
701	"Poaceae;""Festuca summilusitana"";""Autóctone"";""Endémica da Península Ibérica"";""Anexo IIAnexo IV"";""LC"";32;""P.Pereira D.T.Holyoak A.Silva E.Portela-Pereira J.Jansen P.Silveira A.Carapeto"""
702	"Poaceae;""Festuca trichophylla"";""Autóctone"";"""";"""";"""";4;""E.Portela-Pereira"""
703	"Poaceae;""Holcus gayanus"";""Autóctone"";""Endémica da Península Ibérica"";"""";"""";8;""J.Jansen A.Silva D.T.Holyoak"""
704	"Poaceae;""Holcus lanatus"";"""";"""";"""";"""";480;""D.T.Holyoak E.Portela-Pereira F.Clamote A.Silva J.T.Tavares J.Jansen P.Silveira"""
705	"Poaceae;""Holcus mollis"";""Autóctone"";"""";"""";"""";135;""E.Portela-Pereira A.Silva J.Jansen A.Carapeto"""
706	"Poaceae;""Hordeum murinum subsp. murinum"";""Autóctone"";"""";"""";"""";8;""D.T.Holyoak"""
707	"Poaceae;""Hordeum murinum subsp. leporinum"";""Autóctone"";"""";"""";"""";171;""D.T.Holyoak E.Portela-Pereira"""
708	"Poaceae;""Hordeum murinum"";"""";"""";"""";"""";341;""J.Jansen"""

709	"Poaceae;""Koeleria caudata"";""Autóctone"";""Endémica da Península Ibérica"";"""";"""";13;""A.Silva"""
710	"Poaceae;""Koeleria crassipes"";""Autóctone"";""Endémica da Península Ibérica"";"""";"""";16;""D.T.Holyoak P.Ministro"""
711	"Poaceae;""Lolium perenne"";""Autóctone"";"""";"""";"""";64;""D.T.Holyoak A.Silva"""
712	"Poaceae;""Melica uniflora"";""Autóctone"";"""";"""";"""";21;""A.Silva"""
713	"Poaceae;""Mibora minima"";""Autóctone"";"""";"""";"""";89;""A.Silva"""
714	"Poaceae;""Micropyrum patens"";""Autóctone"";"""";"""";"""";31;""J.Jansen"""
715	"Poaceae;""Micropyrum tenellum"";""Autóctone"";"""";"""";"""";160;""J.Jansen A.Silva P.Silveira D.T.Holyoak E.Portela-Pereira M.Porto"""
716	"Poaceae;""Molineriella laevis"";""Autóctone"";"""";"""";"""";62;""J.Jansen"""
717	"Poaceae;""Molinia caerulea"";""Autóctone"";"""";"""";"""";155;""A.Silva E.Portela-Pereira"""
718	"Poaceae;""Nardus stricta"";""Autóctone"";"""";"""";"""";46;""P.V.Araújo A.Carapeto A.Silva A.J.Pereira J.Jansen D.T.Holyoak E.Portela-Pereira P.Pereira"""
719	"Poaceae;""Periballia involucrata"";""Autóctone"";""Endémica da Península Ibérica"";"""";"""";30;""A.Silva"""
720	"Poaceae;""Poa annua"";""Autóctone"";"""";"""";"""";228;""A.Silva D.T.Holyoak P.Silveira"""
721	"Poaceae;""Poa bulbosa"";""Autóctone"";"""";"""";"""";204;""P.Silveira D.T.Holyoak F.Clamote J.Jansen A.Silva"""
722	"Poaceae;""Poa trivialis subsp. trivialis"";""Autóctone"";"""";"""";"""";64;""E.Portela-Pereira"""
723	"Poaceae;""Poa trivialis"";"""";"""";"""";"""";117;""A.Silva D.T.Holyoak"""
724	"Poaceae;""Polypogon monspeliensis"";""Autóctone"";"""";"""";"""";104;""A.Silva"""
725	"Poaceae;""Setaria pumila"";""Autóctone"";"""";"""";"""";35;""A.Silva"""
726	"Poaceae;""Stipa gigantea"";""Autóctone"";"""";"""";"""";214;""A.Carapeto F.Clamote J.Costa A.Silva E.Portela-Pereira J.Jansen D.T.Holyoak"""
727	"Poaceae;""Trisetaria hispida"";""Autóctone"";""Endémica da Península Ibérica"";"""";""NT"";5;""J.Jansen M.Porto E.Portela-Pereira A.Silva A.Matos"""
728	"Poaceae;""Trisetaria ovata"";""Autóctone"";""Endémica da Península Ibérica"";"""";"""";26;""D.T.Holyoak A.Silva"""
729	"Poaceae;""Vulpia bromoides"";""Autóctone"";"""";"""";"""";69;""D.T.Holyoak"""
730	"Poaceae;""Vulpia myuros"";""Autóctone"";"""";"""";"""";136;""P.Silveira D.T.Holyoak"""
731	"Polygalaceae;""Polygala microphylla"";""Autóctone"";"""";"""";""LC"";65;""A.Silva P.Silveira J.Jansen M.Porto"""
732	"Polygalaceae;""Polygala serpyllifolia"";""Autóctone"";"""";"""";"""";37;""M.Porto F.Clamote A.Silva D.T.Holyoak"""
733	"Polygalaceae;""Polygala vulgaris"";""Autóctone"";"""";"""";"""";182;""A.Silva F.Clamote M.Porto J.Jansen J.D.Almeida P.Beja"""

734	"Polygonaceae;""Fallopia convolvulus"";""Autóctone"";"""";"""";"""";115;""F.Clamote A.Silva"""
735	"Polygonaceae;""Polygonum arenastrum"";""Autóctone"";"""";"""";"""";40;""J.D.Almeida"""
736	"Polygonaceae;""Polygonum aviculare"";""Autóctone"";"""";"""";"""";234;""F.Clamote D.T.Holyoak"""
737	"Polygonaceae;""Polygonum hydropiper"";""Autóctone"";"""";"""";"""";146;""F.Clamote"""
738	"Polygonaceae;""Polygonum lapathifolium"";""Autóctone"";"""";"""";"""";139;""F.Clamote"""
739	"Polygonaceae;""Polygonum persicaria"";""Autóctone"";"""";"""";"""";283;""A.Silva E.Portela-Pereira F.Clamote J.D.Almeida"""
740	"Polygonaceae;""Rumex acetosa subsp. acetosa"";""Autóctone"";"""";"""";"""";206;""J.D.Almeida A.Silva E.Portela-Pereira F.Clamote"""
741	"Polygonaceae;""Rumex acetosella subsp. angiocarpus"";""Autóctone"";"""";"""";"""";341;""E.Portela-Pereira P.Silveira F.Clamote J.Jansen A.Silva J.D.Almeida D.T.Holyoak P.V.Araújo"""
742	"Polygonaceae;""Rumex bucephalophorus"";"""";"""";"""";"""";443;""A.Silva A.Carapeto J.Jansen"""
743	"Polygonaceae;""Rumex conglomeratus"";""Autóctone"";"""";"""";"""";234;""E.Portela-Pereira"""
744	"Polygonaceae;""Rumex crispus"";""Autóctone"";"""";"""";"""";197;""A.Silva"""
745	"Polygonaceae;""Rumex induratus"";""Autóctone"";"""";"""";"""";330;""F.Clamote J.D.Almeida A.Silva P.V.Araújo"""
746	"Polygonaceae;""Rumex obtusifolius"";""Autóctone"";"""";"""";"""";107;""J.T.Tavares A.Silva"""
747	"Polygonaceae;""Rumex suffruticosus"";""Autóctone"";""Endémica da Península Ibérica"";"""";""VU"";2;""J.Jansen P.Pereira A.Silva M.Porto A.Carapeto F.Clamote A.Matos"""
748	"Polypodiaceae;""Polypodium cambricum subsp. cambricum"";""Autóctone"";"""";"""";"""";265;""A.Silva F.Clamote"""
749	"Polypodiaceae;""Polypodium interjectum"";""Autóctone"";"""";"""";"""";132;""A.Carapeto E.Portela-Pereira"""
750	"Polypodiaceae;""Polypodium vulgare"";""Autóctone"";"""";"""";"""";82;""A.Silva"""
751	"Portulacaceae;""Montia fontana subsp. amporitana"";""Autóctone"";"""";"""";"""";16;""A.Silva P.Silveira D.T.Holyoak"""
752	"Portulacaceae;""Montia fontana"";"""";"""";"""";"""";143;""F.Clamote A.Silva M.Porto P.Beja P.V.Araújo"""
753	"Portulacaceae;""Portulaca oleracea"";"""";"""";"""";"""";260;""J.D.Almeida"""
754	"Potamogetonaceae;""Potamogeton polygonifolius"";""Autóctone"";"""";"""";"""";65;""A.Silva"""
755	"Primulaceae;""Anagallis arvensis"";""Autóctone"";"""";"""";"""";580;""A.Silva J.D.Almeida"""
756	"Primulaceae;""Anagallis tenella"";""Autóctone"";"""";"""";"""";165;""A.Silva F.Clamote J.Jansen P.V.Araújo M.Porto"""
757	"Primulaceae;""Asterolinon linum-stellatum"";""Autóctone"";"""";"""";"""";283;""F.Clamote J.D.Almeida J.Jansen"""

758	"Primulaceae;""Primula acaulis subsp. acaulis"";""Autóctone"";"""";"""";"""";140;""A.Silva F.Clamote J.D.Almeida M.Porto"""
759	"Proteaceae;""Hakea sericea"";""Introduzida"";""Exótica"";"""";"""";64;""A.Silva"""
760	"Pteridaceae;""Adiantum capillus-veneris"";""Autóctone"";"""";"""";"""";101;""F.Clamote"""
761	"Pteridaceae;""Anogramma leptophylla"";""Autóctone"";"""";"""";"""";412;""A.Silva A.Carapeto F.Clamote P.V.Araújo J.Jansen"""
762	"Pteridaceae;""Cheilanthes hispanica"";""Autóctone"";"""";"""";"""";70;""A.Silva"""
763	"Pteridaceae;""Cheilanthes tinaei"";""Autóctone"";"""";"""";"""";86;""P.Silveira"""
764	"Pteridaceae;""Cryptogramma crispa"";""Autóctone"";"""";"""";""VU"";1;""J.Jansen P.V.Araújo A.Silva A.Matos F.Covelo"""
765	"Ranunculaceae;""Aquilegia vulgaris subsp. dichroa"";""Autóctone"";""Endémica da Península Ibérica"";"""";"""";113;""A.Silva E.Portela-Pereira P.V.Araújo"""
766	"Ranunculaceae;""Aquilegia vulgaris"";"""";"""";"""";"""";168;""F.Clamote A.Carapeto"""
767	"Ranunculaceae;""Caltha palustris"";""Autóctone"";"""";"""";"""";32;""J.Jansen A.Silva"""
768	"Ranunculaceae;""Delphinium halteratum subsp. halteratum"";""Autóctone"";"""";"""";"""";2;""P.V.Araújo"""
769	"Ranunculaceae;""Delphinium halteratum subsp. verdunense"";""Autóctone"";"""";"""";"""";16;""E.Portela-Pereira"""
770	"Ranunculaceae;""Ranunculus abnormis"";""Autóctone"";""Endémica da Península Ibérica"";"""";""VU"";6;""A.Silva J.Jansen S.Ribeiro"""
771	"Ranunculaceae;""Ranunculus bulbosus subsp. aleae"";""Autóctone"";"""";"""";"""";96;""J.Jansen A.Silva E.Portela-Pereira D.T.Holyoak F.Clamote"""
772	"Ranunculaceae;""Ranunculus bulbosus"";"""";"""";"""";"""";189;""A.Silva"""
773	"Ranunculaceae;""Ranunculus bupleuroides"";""Autóctone"";""Endémica da Península Ibérica"";"""";"""";59;""A.Silva J.Jansen"""
774	"Ranunculaceae;""Ranunculus ficaria subsp. ficaria"";""Autóctone"";"""";"""";"""";235;""A.Silva P.Beja"""
775	"Ranunculaceae;""Ranunculus flammula"";""Autóctone"";"""";"""";"""";84;""A.Silva F.Clamote J.Jansen"""
776	"Ranunculaceae;""Ranunculus hederaceus"";""Autóctone"";"""";"""";"""";11;""J.Jansen"""
777	"Ranunculaceae;""Ranunculus henriquesii"";""Endémica"";""Endémica de Portugal continental"";"""";""VU"";18;""A.Silva F.Clamote"""
778	"Ranunculaceae;""Ranunculus nigrescens"";""Autóctone"";""Endémica da Península Ibérica"";"""";"""";53;""J.Jansen A.Silva J.D.Almeida D.T.Holyoak P.V.Araújo E.Portela-Pereira S.Ribeiro F.Clamote M.Porto P.Silveira A.Carapeto J.T.Tavares"""
779	"Ranunculaceae;""Ranunculus ollissiponensis subsp. ollissiponensis"";""Autóctone"";""Endémica da Península Ibérica"";"""";"""";167;""J.Jansen P.V.Araújo A.Silva F.Clamote J.D.Almeida P.Silveira"""

780	"Ranunculaceae;""Ranunculus ololeucos"";""Autóctone"";"""";"""";"""";13;""S.Ribeiro F.Clamote M.Porto A.Silva D.T.Holyoak J.Jansen"""
781	"Ranunculaceae;""Ranunculus omiophyllus"";""Autóctone"";"""";"""";"""";73;""A.Silva J.Jansen P.Silveira"""
782	"Ranunculaceae;""Ranunculus paludosus"";""Autóctone"";"""";"""";"""";123;""B.Moreira"""
783	"Ranunculaceae;""Ranunculus pseudofluitans"";""Autóctone"";"""";"""";"""";9;""E.Portela-Pereira"""
784	"Ranunculaceae;""Ranunculus repens"";""Autóctone"";"""";"""";"""";227;""A.Silva E.Portela-Pereira F.Clamote J.Jansen"""
785	"Ranunculaceae;""Thalictrum speciosissimum"";""Autóctone"";"""";"""";"""";166;""A.Silva J.Jansen"""
786	"Resedaceae;""Reseda gredensis"";""Autóctone"";""Endémica da Península Ibérica"";"""";""EN"";1;""A.Silva A.Matos J.Jansen M.Porto"""
787	"Resedaceae;""Reseda luteola"";""Autóctone"";"""";"""";"""";216;""A.Silva F.Clamote"""
788	"Resedaceae;""Reseda media"";""Autóctone"";"""";"""";"""";213;""A.Silva M.Porto P.Beja"""
789	"Resedaceae;""Sesamoides purpurascens"";""Autóctone"";"""";"""";"""";241;""D.T.Holyoak J.D.Almeida A.Silva P.Silveira J.Jansen"""
790	"Resedaceae;""Sesamoides suffruticosa"";"""";"""";"""";"""";102;""P.Silveira D.T.Holyoak F.Clamote M.Porto A.Silva J.Jansen"""
791	"Rhamnaceae;""Frangula alnus"";""Autóctone"";"""";"""";"""";324;""E.Portela-Pereira J.Jansen F.Clamote A.Silva P.Silveira M.Porto J.T.Tavares"""
792	"Rosaceae;""Agrimonia eupatoria"";"""";"""";"""";"""";158;""A.Silva"""
793	"Rosaceae;""Alchemilla transiens"";""Autóctone"";"""";"""";""CR"";1;""A.Carapeto A.Matos C.Aguiar C.Meireles A.Silva J.Jansen F.Clamote M.Porto J.D.Almeida"""
794	"Rosaceae;""Aphanes australis"";""Autóctone"";"""";"""";"""";133;""J.Jansen"""
795	"Rosaceae;""Crataegus monogyna"";""Autóctone"";"""";"""";"""";539;""E.Portela-Pereira F.Clamote A.Silva J.T.Tavares J.Jansen P.V.Araújo P.Beja"""
796	"Rosaceae;""Fragaria vesca subsp. vesca"";""Autóctone"";"""";"""";"""";115;""A.Silva P.Beja J.D.Almeida M.Porto"""
797	"Rosaceae;""Geum sylvaticum"";""Autóctone"";"""";"""";"""";93;""P.Beja A.Silva M.Porto"""
798	"Rosaceae;""Geum urbanum"";""Autóctone"";"""";"""";"""";72;""A.Silva E.Portela-Pereira"""
799	"Rosaceae;""Potentilla erecta"";""Autóctone"";"""";"""";"""";203;""D.T.Holyoak M.Porto F.Clamote A.Silva P.V.Araújo P.Beja J.Jansen E.Portela-Pereira"""
800	"Rosaceae;""Prunus avium"";""Autóctone"";"""";"""";"""";120;""P.V.Araújo A.Silva E.Portela-Pereira J.Jansen P.Ministro"""
801	"Rosaceae;""Prunus insititia"";""Autóctone"";"""";"""";"""";36;""E.Portela-Pereira E.Marabuto"""
802	"Rosaceae;""Prunus lusitanica subsp. lusitanica"";""Autóctone"";"""";"""";""NT"";29;""A.Silva M.Raposo J.Costa M.Porto J.Jansen"""

803	"Rosaceae;""Prunus spinosa"";""Autóctone"";"""";"""";"""";115;""A.Silva J.Jansen"""
804	"Rosaceae;""Rosa canina"";""Autóctone"";"""";"""";"""";125;""A.Silva E.Portela-Pereira"""
805	"Rosaceae;""Rosa corymbifera"";""Autóctone"";"""";"""";"""";35;""A.Silva"""
806	"Rosaceae;""Rosa pouzinii"";""Autóctone"";"""";"""";"""";59;""A.Silva E.Portela-Pereira"""
807	"Rosaceae;""Rosa rubiginosa"";""Autóctone"";"""";"""";""CR"";1;""A.Silva"""
808	"Rosaceae;""Rosa squarrosa"";""Autóctone"";"""";"""";"""";41;""E.Portela-Pereira"""
809	"Rosaceae;""Rubus genevieri"";""Autóctone"";"""";""Anexo V"";""VU"";11;""P.R.González"""
810	"Rosaceae;""Rubus henriquesii"";""Autóctone"";""Endémica da Península Ibérica"";"""";"""";28;""A.Silva J.D.Almeida"""
811	"Rosaceae;""Rubus peratticus"";""Autóctone"";""Endémica da Península Ibérica"";"""";"""";9;""E.Portela-Pereira"""
812	"Rosaceae;""Rubus radula"";""Autóctone"";"""";"""";"""";7;""E.Portela-Pereira"""
813	"Rosaceae;""Rubus ulmifolius var. ulmifolius"";""Autóctone"";"""";"""";"""";652;""E.Portela-Pereira A.Silva J.D.Almeida M.Porto"""
814	"Rosaceae;""Rubus vagabundus"";""Autóctone"";""Endémica da Península Ibérica"";"""";"""";7;""E.Portela-Pereira"""
815	"Rosaceae;""Sanguisorba verrucosa"";""Autóctone"";"""";"""";"""";443;""A.Silva J.Jansen F.Clamote"""
816	"Rosaceae;""Sorbus aria"";""Autóctone"";"""";"""";""CR"";4;""E.Portela-Pereira"""
817	"Rosaceae;""Sorbus aucuparia"";""Autóctone"";"""";"""";"""";29;""E.Portela-Pereira A.Silva S.Ribeiro F.Clamote P.V.Araújo J.T.Tavares D.T.Holyoak J.D.Almeida A.Carapeto"""
818	"Rosaceae;""Sorbus latifolia"";""Autóctone"";"""";"""";""VU"";20;""A.Silva A.Matos J.Jansen J.Costa"""
819	"Rosaceae;""Sorbus torminalis"";""Autóctone"";"""";"""";""VU"";10;""A.Silva"""
820	"Rubiaceae;""Crucianella angustifolia"";""Autóctone"";"""";"""";"""";225;""M.Porto A.Silva F.Clamote D.T.Holyoak"""
821	"Rubiaceae;""Galium aparine subsp. aparine"";""Autóctone"";"""";"""";"""";195;""E.Portela-Pereira J.D.Almeida"""
822	"Rubiaceae;""Galium aparine"";"""";"""";"""";"""";609;""A.Silva F.Clamote"""
823	"Rubiaceae;""Galium broterianum"";""Autóctone"";""Endémica da Península Ibérica"";"""";"""";150;""E.Portela-Pereira J.Jansen A.Silva F.Clamote P.V.Araújo"""
824	"Rubiaceae;""Galium lucidum subsp. lucidum"";""Autóctone"";"""";"""";"""";35;""P.V.Araújo F.Clamote"""
825	"Rubiaceae;""Galium mollugo subsp. erectum"";""Autóctone"";"""";"""";"""";19;""E.Portela-Pereira F.Clamote"""
826	"Rubiaceae;""Galium mollugo subsp. mollugo"";""Autóctone"";"""";"""";"""";30;""E.Portela-Pereira"""
827	"Rubiaceae;""Galium mollugo"";"""";"""";"""";"""";122;""A.Silva D.T.Holyoak"""

828	"Rubiaceae;""Galium palustre var. palustre"";""Autóctone"";"""";"""";"""";15;""J.Jansen"""
829	"Rubiaceae;""Galium palustre"";"""";"""";"""";"""";157;""A.Silva"""
830	"Rubiaceae;""Galium rotundifolium"";""Autóctone"";"""";"""";"""";25;""P.Pereira A.Silva E.Portela-Pereira P.Guillaumeau P.V.Araújo"""
831	"Rubiaceae;""Galium saxatile var. saxatile"";""Autóctone"";"""";"""";"""";7;""J.Jansen A.Silva"""
832	"Rubiaceae;""Galium saxatile var. vivianum"";""Autóctone"";"""";"""";"""";11;""J.Jansen P.V.Araújo A.Silva"""
833	"Rubiaceae;""Galium saxatile"";"""";"""";"""";"""";81;""A.Silva P.V.Araújo F.Clamote D.T.Holyoak P.Pereira"""
834	"Rubiaceae;""Galium verum subsp. verum"";""Autóctone"";"""";"""";"""";45;""A.Silva"""
835	"Rubiaceae;""Rubia peregrina"";""Autóctone"";"""";"""";"""";467;""M.Porto J.Jansen A.Silva J.D.Almeida F.Clamote E.Portela-Pereira"""
836	"Rubiaceae;""Sherardia arvensis"";""Autóctone"";"""";"""";"""";470;""F.Clamote A.Silva P.Beja"""
837	"Salicaceae;""Populus nigra var. nigra"";""Introduzida"";""Exótica"";"""";"""";30;""E.Portela-Pereira"""
838	"Salicaceae;""Populus nigra"";""Introduzida"";"""";"""";"""";158;""A.Silva E.Portela-Pereira"""
839	"Salicaceae;""Salix atrocinerea"";""Autóctone"";"""";"""";"""";503;""E.Portela-Pereira D.T.Holyoak A.Silva P.Silveira P.V.Araújo J.Jansen J.D.Almeida"""
840	"Salicaceae;""Salix fragilis"";""Introduzida"";""Exótica"";"""";"""";31;""E.Portela-Pereira"""
841	"Salicaceae;""Salix neotricha"";""Autóctone"";"""";"""";"""";30;""E.Portela-Pereira"""
842	"Salicaceae;""Salix salviifolia subsp. salviifolia"";""Autóctone"";""Endémica da Península Ibérica"";"""";"""";147;""E.Portela-Pereira P.V.Araújo"""
843	"Salicaceae;""Salix salviifolia"";"""";"""";"""";"""";399;""D.T.Holyoak J.D.Almeida P.V.Araújo A.Silva A.Carapeto P.Silveira"""
844	"Santalaceae;""Osyris alba"";""Autóctone"";"""";"""";"""";340;""A.Silva M.Porto J.Jansen J.D.Almeida"""
845	"Santalaceae;""Thesium humifusum"";""Autóctone"";"""";"""";"""";34;""A.Silva J.D.Almeida"""
846	"Santalaceae;""Thesium pyrenaicum subsp. pyrenaicum"";""Autóctone"";"""";"""";"""";18;""J.D.Almeida"""
847	"Sapindaceae;""Acer pseudoplatanus"";""Autóctone"";"""";"""";"""";90;""F.Clamote E.Portela-Pereira J.T.Tavares A.Silva C.Meireles A.Carapeto P.Pereira P.V.Araújo"""
848	"Saxifragaceae;""Chrysosplenium oppositifolium"";""Autóctone"";"""";"""";"""";42;""J.Jansen A.Silva P.Pereira F.Clamote M.Porto P.V.Araújo A.Carapeto"""
849	"Saxifragaceae;""Saxifraga fragosoi"";""Autóctone"";"""";"""";"""";70;""A.Silva F.Clamote P.Pereira M.Porto P.V.Araújo J.D.Almeida E.Portela-Pereira"""
850	"Saxifragaceae;""Saxifraga granulata"";""Autóctone"";"""";"""";"""";298;""A.Silva D.T.Holyoak P.V.Araújo P.Beja F.Clamote"""

851	"Saxifragaceae;""Saxifraga spathularis"";""Autóctone"";"""";"""";"""";66;""J.D.Almeida A.Carapeto A.Silva F.Clamote P.V.Araújo D.T.Holyoak M.Porto J.T.Tavares E.Portela-Pereira J.Jansen P.Pereira P.Ministro P.Guillaumeau"""
852	"Saxifragaceae;""Saxifraga stellaris"";""Autóctone"";"""";"""";""VU"";1;""J.Jansen A.Silva J.D.Almeida D.T.Holyoak A.Matos A.Carapeto F.Clamote P.V.Araújo"""
853	"Scrophulariaceae;""Scrophularia auriculata subsp. auriculata"";""Autóctone"";"""";"""";"""";181;""A.Silva"""
854	"Scrophulariaceae;""Scrophularia canina subsp. canina"";""Autóctone"";"""";"""";"""";157;""F.Clamote A.Silva"""
855	"Scrophulariaceae;""Scrophularia herminii"";""Autóctone"";""Endémica da Península Ibérica"";""Anexo V"";""LC"";26;""E.Portela-Pereira J.Jansen A.Silva M.Porto P.Pereira F.Clamote J.D.Almeida"""
856	"Scrophulariaceae;""Scrophularia schousboei"";""Autóctone"";""Endémica da Península Ibérica"";""Anexo V"";"""";18;""A.Silva J.Jansen"""
857	"Scrophulariaceae;""Scrophularia scorodonia"";"""";"""";"""";"""";299;""F.Clamote A.Silva P.V.Araújo E.Portela-Pereira"""
858	"Scrophulariaceae;""Verbascum pulverulentum"";""Autóctone"";"""";"""";"""";112;""E.Portela-Pereira F.Clamote A.Silva E.Marabuto"""
859	"Scrophulariaceae;""Verbascum simplex"";""Autóctone"";"""";"""";"""";38;""E.Portela-Pereira"""
860	"Scrophulariaceae;""Verbascum sinuatum"";""Autóctone"";"""";"""";"""";231;""A.Silva"""
861	"Scrophulariaceae;""Verbascum thapsus"";""Autóctone"";"""";"""";"""";61;""A.Silva F.Clamote"""
862	"Scrophulariaceae;""Verbascum virgatum"";""Autóctone"";"""";"""";"""";141;""F.Clamote"""
863	"Simaroubaceae;""Ailanthus altissima"";""Introduzida"";""Exótica"";"""";"""";156;""F.Clamote A.Carapeto A.Silva"""
864	"Solanaceae;""Solanum chenopodioides"";""Introduzida"";""Exótica"";"""";"""";166;""A.Silva"""
865	"Solanaceae;""Solanum dulcamara"";""Autóctone"";"""";"""";"""";167;""P.V.Araújo F.Clamote E.Portela-Pereira"""
866	"Solanaceae;""Solanum nigrum"";""Autóctone"";"""";"""";"""";398;""A.Silva J.D.Almeida F.Clamote"""
867	"Taxaceae;""Taxus baccata"";""Autóctone"";"""";"""";""EN"";12;""E.Portela-Pereira A.Silva A.Carapeto F.Clamote J.Jansen S.Ribeiro D.T.Holyoak J.M.R.P.† M.Porto"""
868	"Thymelaeaceae;""Daphne gnidium"";""Autóctone"";"""";"""";"""";635;""A.Silva F.Clamote M.Porto P.Beja P.V.Araújo"""
869	"Thymelaeaceae;""Thymelaea coridifolia subsp. dendrobryum"";""Autóctone"";""Endémica da Península Ibérica"";"""";""EN"";1;""C.Meireles J.Jansen A.Silva D.T.Holyoak A.Matos P.Pereira P.V.Araújo"""
870	"Typhaceae;""Sparganium angustifolium"";""Autóctone"";"""";"""";""CR"";1;""A.Silva J.Jansen A.Matos"""

871	"Typhaceae;""Typha latifolia"";""Autóctone"";"""";"""";"""";117;""A.Silva"""
872	"Ulmaceae;""Ulmus glabra"";""Introduzida"";""Exótica"";"""";"""";18;""A.Silva"""
873	"Ulmaceae;""Ulmus minor"";""Autóctone"";"""";"""";"""";172;""A.Silva E.Portela-Pereira P.V.Araújo"""
874	"Urticaceae;""Parietaria judaica"";""Autóctone"";"""";"""";"""";306;""A.Silva M.Porto"""
875	"Urticaceae;""Urtica dioica"";""Autóctone"";"""";"""";"""";308;""D.T.Holyoak A.Silva E.Portela-Pereira F.Clamote J.T.Tavares J.Jansen"""
876	"Urticaceae;""Urtica membranacea"";""Autóctone"";"""";"""";"""";294;""E.Portela-Pereira J.D.Almeida"""
877	"Urticaceae;""Urtica urens"";""Autóctone"";"""";"""";"""";105;""P.Beja"""
878	"Valerianaceae;""Centranthus calcitrapae var. calcitrapae"";""Autóctone"";"""";"""";"""";498;""A.Silva F.Clamote D.T.Holyoak J.Jansen P.V.Araújo"""
879	"Valerianaceae;""Centranthus ruber subsp. ruber"";""Autóctone"";"""";"""";"""";55;""A.Silva"""
880	"Verbenaceae;""Verbena officinalis"";""Autóctone"";"""";"""";"""";312;""F.Clamote A.Silva J.T.Tavares"""
881	"Violaceae;""Viola arvensis"";""Autóctone"";"""";"""";"""";11;""J.Jansen"""
882	"Violaceae;""Viola kitaibeliana"";""Autóctone"";"""";"""";"""";190;""P.Beja A.Silva"""
883	"Violaceae;""Viola langeana"";""Autóctone"";""Endémica da Península Ibérica"";"""";""LC"";16;""J.Jansen M.Porto F.Clamote P.Pereira A.Silva A.Carapeto P.Silveira D.T.Holyoak P.V.Araújo P.Beja"""
884	"Violaceae;""Viola palustris subsp. palustris"";""Autóctone"";"""";"""";"""";79;""A.Silva E.Portela-Pereira J.Jansen S.Ribeiro"""
885	"Violaceae;""Viola riviniana"";""Autóctone"";"""";"""";"""";301;""P.Beja E.Portela-Pereira A.Silva F.Clamote J.Jansen M.Porto A.Carapeto P.V.Araújo"""
886	"Vitaceae;""Vitis vinifera subsp. sylvestris"";""Autóctone"";"""";"""";"""";55;""J.T.Tavares"""
887	"Woodsiaceae;""Athyrium filix-femina"";""Autóctone"";"""";"""";"""";256;""E.Portela-Pereira F.Clamote A.Silva J.T.Tavares D.T.Holyoak P.V.Araújo J.Jansen P.Ministro"""
888	"Woodsiaceae;""Cystopteris dickieana"";""Autóctone"";"""";"""";"""";6;""D.T.Holyoak P.Ministro F.Clamote"""
889	"Woodsiaceae;""Cystopteris fragilis subsp. fragilis"";""Autóctone"";"""";"""";"""";7;""A.Silva D.T.Holyoak J.Jansen"""
890	"Woodsiaceae;""Cystopteris viridula"";""Autóctone"";"""";"""";"""";83;""F.Clamote A.Silva"""
891	"Xanthorrhoeaceae;""Asphodelus albus subsp. villarsii"";""Autóctone"";"""";"""";"""";8;""A.Silva P.Pereira"""
892	"Xanthorrhoeaceae;""Asphodelus lusitanicus"";"""";"""";"""";"""";70;""F.Clamote"""
893	"Xanthorrhoeaceae;""Simethis mattiazzi"";""Autóctone"";"""";"""";"""";223;""J.D.Almeida P.Silveira M.Porto J.Jansen A.Silva"""
