# Supplementary material for: Overview of Ethnobotanical–Pharmacological Studies Carried Out on Medicinal Plants from the Serra da Estrela Natural Park: Focus on Their Antidiabetic Potential
Source: Pharmaceutics. 2024 Mar 25;16(4):454. doi: 10.3390/pharmaceutics16040454 (PMC11054966; doi:10.3390/pharmaceutics16040454)
Supplement: Supplementary file 1 [file pharmaceutics-16-00454-s001.zip › Suplementery Tables S2 (1).pdf]

Supplementary Tables S2: Analysis of the plant number with anti-diabetic potential in Serra da Estrela Natural Park. (Group A, B, C and D).

	Family	Total number of species	Number of species with anti-diabetic potential
**1**	Asteraceae	108	21
**2**	Lamiaceae	29	12
**3**	Fabaceae	74	9
**4**	Rosaceae	28	8
**5**	Caryophyllaceae	60	6
**6**	Polygalaceae	22	5
**7**	Apiaceae	29	4
**8**	Asparagaceae	13	4
**9**	Ericaceae	13	4
**10**	Boraginaceae	13	3
**11**	Geraniaceae	9	3
**12**	Hypericaceae	8	3
**13**	Fagaceae	7	3

Group A. Number of families with more than three plants with anti-diabetic potential (13 families/85 species).

Group B. Number of families with only two plants with anti-diabetic potential (12 families/24 species).

	Family	Total number of species	Number of species with anti-diabetic potential
**1**	Brassicaceae	33	2
**2**	Cistaceae	16	2
**3**	Amaryllidaceae	15	2
**4**	Scrophulariaceae	10	2
**5**	Papaveraceae	9	2
**6**	Caprifoliaceae	5	2
**7**	Gentianaceae	5	2
**8**	Urticaceae	3	2
**9**	Malvaceae	3	2
**10**	Cupressaceae	2	2
**11**	Cytinaceae	2	2
**12**	Pinaceae	2	2

Group C. Number of families with only one plant with anti-diabetic potential (30 families/30 species).

	Family	Total number of species	Number of species with anti-diabetic potential
**1**	Pteridaceae	6	1
**2**	Poaceae	81	1
**3**	Juncaceae	18	1
**4**	Rubiaceae	17	1

**5**	Campanulaceae	11	1
**6**	Dryopteridaceae	7	1
**7**	Betulaceae	4	1
**8**	Primulaceae	4	1
**9**	Portulacaceae	3	1
**10**	Oleaceae	3	1
**11**	Solanaceae	3	1
**12**	Amaranthaceae	2	1
**13**	Lycopodiaceae	2	1
**14**	Lythraceae	2	1
**15**	Thymelaeaceae	2	1
**16**	Ulmaceae	2	1
**17**	Lauraceae	1	1
**18**	Cannabaceae	1	1
**19**	Convolvulaceae	1	1
**20**	Cucurbitaceae	1	1
**21**	Dioscoreaceae	1	1
**22**	Moraceae	1	1
**23**	Myrtaceae	1	1
**24**	Oxalidaceae	1	1
**25**	Phytolaccaceae	1	1
**26**	Simaroubaceae	1	1
**27**	Taxaceae	1	1
**28**	Verbenaceae	1	1
**29**	Vitaceae	1	1
**30**	Buxaceae	1	1

Group D. Number of families without plants with anti-diabetic potential (42 families).

	Family	Total number of species	Number of species with anti-diabetic potential
**1**	Plantaginaceae	27	0
**2**	Ranunculaceae	21	0
**3**	Cyperaceae	19	0
**4**	Crassulaceae	14	0
**5**	Onagraceae	7	0
**6**	Orchidaceae	6	0
**7**	Orobanchaceae	8	0
**8**	Liliaceae	8	0
**9**	Salicaceae	7	0
**10**	Aspleniaceae	6	0
**11**	Euphorbiaceae	5	0
**12**	Plumbaginaceae	5	0
**13**	Resedaceae	5	0
**14**	Saxifragaceae	5	0
**15**	Violaceae	5	0
**16**	Iridaceae	4	0
**17**	Woodsiaceae	4	0
**18**	Santalaceae	3	0
**19**	Apocynaceae	2	0
**20**	Araliaceae	2	0
**21**	Paeoniaceae	2	0
**22**	Typhaceae	2	0
**23**	Valerianaceae	2	0
**24**	Xanthorrhoeaceae	2	0
**25**	Colchicaceae	1	0
**26**	Commelinaceae	1	0
**27**	Dennstaedtiaceae	1	0
**28**	Droseraceae	1	0

**29**	Elatinaceae	1	0
**30**	Haloragaceae	1	0
**31**	Melanthiaceae	1	0
**32**	Menyanthaceae	1	0
**33**	Potamogetonaceae	1	0
**34**	Proteaceae	1	0
**35**	Rhamnaceae	1	0
**36**	Sapindaceae	1	0
**37**	Osmundaceae	1	0
**38**	Alismataceae	1	0
**39**	Aquifoliaceae	1	0
**40**	Araceae	1	0
**41**	Aristolochiaceae	1	0
**42**	Blechnaceae	1	0
