# Supplementary material for: Overview of Ethnobotanical–Pharmacological Studies Carried Out on Medicinal Plants from the Serra da Estrela Natural Park: Focus on Their Antidiabetic Potential
Source: Pharmaceutics. 2024 Mar 25;16(4):454. doi: 10.3390/pharmaceutics16040454 (PMC11054966; doi:10.3390/pharmaceutics16040454)
Supplement: Supplementary file 1 [file pharmaceutics-16-00454-s001.zip › Suplementery Tables S4.pdf]

Supplementary Table S4: Species of plants in Serra da Estrela Natural Park reported to have anti-diabetic potential.

Category (A): Plant species in NPSE are reportedly used traditionally to treat diabetes. Category (B): Plant species in NPSE with no evidence of traditional use against diabetes but with scientific validation of claimed anti-diabetic effects.

**Category A**	
	**Asteraceae**
**1**	*Arctium minus (Hill) Bernh*
**2**	*Achillea millefolium*
**3**	*Arnica montana subsp. atlantica*
**4**	*Calendula arvensis*
**5**	*Chamaemelum nobile (syn. Anthemis nobilis L. or Chamomilla nobilis (L.) Godr.)*
**6**	*Cichorium intybus*
**7**	*Dittrichia viscoso subsp. Viscosa (Syn. Inula viscosa)*
**8**	*Galinsoga parviflora (Syn. Galinsoga quadriradiata*
**9**	*Hypochaeris radicata*
**10**	*Solidago virgaurea*
**11**	*Sonchus asper*
**12**	*Sonchus oleraceus*
**13**	*Tanacetum vulgare*
	**Fabaceae**
**14**	*Pisum sativum*
**15**	*Pterospartum tridentatum*
**16**	*Retama sphaerocarpa*
**17**	*Trifolium pratense subsp. pratense*
	**Caryophyllaceae**
**18**	*Corrigiola litoralis*
**19**	*Corrigiola telephiifolia*
**20**	*Paronychia argentea*
**21**	*Saponaria officinalis*
**22**	*Stellaria media*
	**Brassicaceae**
**23**	*Capsella bursa-pastoris*

**24**	*Raphanus raphanistrum subsp. raphanistrum*
	**Apiaceae**
**25**	*Daucus carota*
**26**	*Foeniculum vulgare*
**27**	*Heracleum sphondylium*
	**Lamiaceae**
**28**	*Acinos alpinus*
**29**	*Clinopodium nepeta subsp. Spruneri (Syn. Calamintha officinalis Moench)*
**30**	*Lavandula stoechas*
**31**	*Mentha aquatica*
**32**	*Mentha pulegium*
**33**	*Mentha suaveolens*
**34**	*Origanum vulgare*
**35**	*Prunella vulgaris*
**36**	*Salvia verbenaca*
	**Rosaceae**
**37**	*Agrimonia eupatoria*
**38**	*Crataegus monogyna*
**39**	*Geum urbanum*
**40**	*Potentilla erecta*
**41**	*Prunus avium*
**42**	*Rosa canina*
**43**	*Sorbus aucuparia*
	**Polygalaceae**
**44**	*Polygonum aviculare*
**45**	*Rumex acetosa subsp. acetosa*
**46**	*Rumex crispus*
	**Rubiaceae**
**47**	*Galium aparine*
**Cistaceae**	
**48**	*Cistus ladanifer*
**49**	*Cistus salviifolius*
	**Asparagaceae**

**50**	*Polygonatum odoratum*	
**51**	*Urginea maritima*	
	**Boraginaceae**	
**52**	*Anchusa undulata*	
	**Ericaceae**	
**53**	*Arbutus unedo*	
**54**	*Vaccinium myrtillus*	
	**Scrophulariaceae**	
**55**	*Verbascum thapsus*	
	**Geraniaceae**	
**56**	*Geranium robertianum*	
	**Papaveraceae**	
**57**	*Chelidonium majus*	
	**Hypericaceae**	
**58**	*Hypericum perforatum*	
	**Pteridaceae**	
**59**	*Adiantum capillus-veneris L.*	
	**Gentianaceae**	
**60**	*Centaurium erythraea*	
**61**	*Gentiana lutea subsp. lutea*	
	**Primulaceae**	
**62**	*Anagallis arvensis*	
	**Urticaceae**	
**63**	*Urtica dioica*	
	**Portulacaceae**	
**64**	*Portulaca oleracea*	
	**Malvaceae**	
**65**	*Malva neglecta*	
	**Oleaceae**	
**66**	*Olea europaea var. europaea*	
	**Solanaceae**	
**67**	*Solanum nigrum*	
	**Amaranthaceae**	

**68**	*Chenopodium ambrosioides*
	**Cupressaceae**
**69**	*Juniperus communis*
	**Lycopodiaceae**
**70**	*Lycopodium clavatum*
	**Pinaceae**
**71**	*Pinus pinaster*
	**Thymelaeaceae**
**72**	*Daphne gnidium*
	**Lauraceae**
**73**	*Laurus nobilis*
	**Cannabaceae**
**74**	*Humulus lupulus*
	**Convolvulaceae**
**75**	*Convolvulus arvensis*
	**Cucurbitaceae**
**76**	*Bryonia dioica*
	**Moraceae**
**77**	*Ficus carica*
	**Myrtaceae**
**78**	*Eucalyptus globulus*
	**Simaroubaceae**
**79**	*Ailanthus altissima*
	**Taxaceae**
**80**	*Taxus baccata*
	**Verbenaceae**
**81**	*Verbena officinalis*
	**Vitaceae**
**82**	*Vitis vinifera subsp. sylvestris*
	**Buxaceae**
**83**	*Buxus sempervirens*

Category B

	**Asteraceae**
**1**	*Anthemis canescens var aurea (syn. Matricaria aurea)*
**2**	*Bellis perennis*
**3**	*Bidens frondosa*
**4**	*Helichrysum stoechas subsp. stoechas*
**5**	*Lactuca serriola*
**6**	*Onopordum acanthium subsp. acanthium*
**7**	*Senecio vulgaris*
**8**	*Tanacetum parthenium*
	**Poaceae**
**9**	*Avena sativa*
	**Fabaceae**
**10**	*Acacia dealbata*
**11**	*Lupinus angustifolius*
**12**	*Lupinus luteus*
**13**	*Robinia pseudoacacia*
**14**	*Trifolium repens*
	**Caryophyllaceae**
**15**	*Spergularia rubra*
	**Apiaceae**
**16**	*Eryngium campestre*
	**Lamiaceae**
**17**	*Lavandula pedunculata subsp. pedunculata*
**18**	*Melissa officinalis*
**19**	*Thymus mastichina*
	**Rosaceae**
**20**	*Prunus lusitanica subsp. lusitanica*
	**Polygalaceae**
**21**	*Polygonum hydropiper*
**22**	*Rumex obtusifolius*
	**Juncaceae**
**23**	*Juncus acutus*
	**Amaryllidaceae**

**24**	*Allium victorialis*
**25**	*Narcissus pseudonarcissus*
	**Asparagaceae**
**26**	*Muscari comosum*
**27**	*Ruscus aculeatus*
	**Boraginaceae**
**28**	*Echium plantagineum*
**29**	*Lithodora prostrata*
	**Ericaceae**
**31**	*Erica scoparia* subsp. *scoparia*
**32**	*Vaccinium uliginosum*
	**Campanulaceae**
**33**	*Jasione montana var. gracilis (syn. Jasione montana* subsp. *Montana)*
	**Scrophulariaceae**
**34**	*Verbascum sinuatum*
	**Geraniaceae**
**35**	*Geranium purpureum*
**36**	*Geranium pyrenaicum* subsp. *lusitanicum*
	**Papaveraceae**
**37**	*Papaver dubium*
	**Hypericaceae**
**38**	*Hypericum androsaemum*
**39**	*Hypericum humifusum*
	**Fagaceae**
**40**	*Castanea sativa*
**41**	*Quercus pyrenaica*
**42**	*Quercus suber*
	**Caprifoliaceae**
**43**	*Lonicera periclymenum*
**44**	*Sambucus nigra*
	**Betulaceae**
**45**	*Corylus avellana*
	**Urticaceae**

**46**	*Urtica membranacea*
	**Malvaceae**
**47**	*Malva sylvestris*
	**Lythraceae**
**48**	*Lythrum salicaria*
	**Cytinaceae**
**49**	*Cytinus hypocistis*
**50**	*Cytinus hypocistis subsp. hypocistis*
	**Pinaceae**
**51**	*Pinus sylvestris*
	**Ulmaceae**
**52**	*Ulmus glabra*
	**Dioscoreaceae**
**53**	*Tamus communis*
	**Oxalidaceae**
**54**	*Oxalis pes-caprae*
	**Phytolaccaceae**
**55**	*Phytolacca americana*
